# Reversible CO Insertion into the Si = Si Double Bond Enables a Disila-Bislactone Formation via Subsequent CO_2_ Addition

**DOI:** 10.1021/jacs.5c07040

**Published:** 2025-07-21

**Authors:** Fiona J. Kiefer, Arseni Kostenko, Richard Holzner, Shigeyoshi Inoue

**Affiliations:** TUM School of Natural Sciences, Department of Chemistry, Institute of Silicon Chemistry and Catalysis Research Center, Technische Universität München, Lichtenbergstraße 4, Garching bei München 85748, Germany

## Abstract

Carbon monoxide inserts into the Si=Si double bond of an imino­(silyl)­disilene and tetra­(silyl)­disilene affording the corresponding 1,3-disilaallene oxides, characterized by NMR spectroscopy and X-ray crystallography and supported by quantum chemical calculations. In the case of imino­(silyl)­disilene, the CO insertion is less exergonic than for tetrasilyldisilene and, thus, is fully reversible in the presence of a CO trapping agent. The reaction mechanisms and reversibility facilitated by the imino-substituent were supported by quantum chemical calculations. The imino-supported 1,3-disilaallene oxide can react with low-pressure CO_2_ to a disila-2,4-furandione; with increased CO_2_ pressure, a 6-membered ring, disila-bislactone is formed. Their formation mechanisms were investigated via DFT calculations. Reaction of CO_2_ with 1,3-disilaallene oxide was compared to the imino­(silyl)­disilene reacting with CO_2_ in a [2 + 2] cycloaddition.

## Graphical abstract

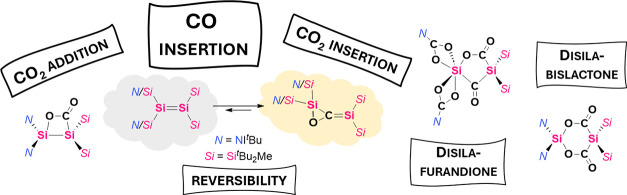

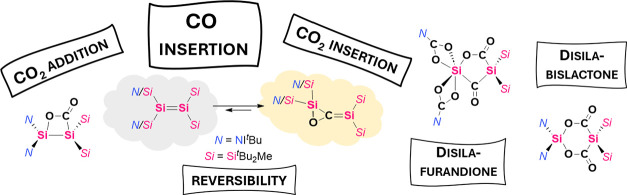

## Introduction

Carbon monoxide is commonly used as C1 feedstock in industrially important organic transformations, such as the Pauson–Khand reaction and Fischer–Tropsch, Monsanto, and Cativa processes.^1–5^ Because the C≡O bond is one of the strongest bonds in chemistry (257.3 kcal/mol),^6^ its activation and functionalization are notoriously challenging and typically require transition-metal catalysts. Nevertheless, there are several reports of CO activation by main-group-element-based compounds.^7^ The most notable examples include the CO reactivity with diboron hydride, which has long been known,^8,9^ and since then various other boron compounds have been found to react with CO.^10–16^ Additionally, CO capture by aluminum-based species^17–19^ and reversible CO insertion into an Al–C bond have been reported.^20^ Silicon compounds have also shown CO activation, with reports of silicon carbonyl complexes,^21,22^ CO homologation by silylenes and disilenides, and CO activation by silicon species.^7^ Noteworthy are the disilyl ketone **I** (Chart 1) formed from a Si–Si bond,^23^ the reversible CO insertion into a 1,2-disilylene,^24^ the CO addition to a disilyne,^25^ and very recently, a disilicon-mediated CO activation and splitting was reported.^26^ Focusing on CO reactivity with disilenes (Si=Si, Chart 1), a few examples have been reported resulting in silenes (e.g., **II**) via a bicyclobutanone intermediate,^27,28^ and the CO homologation product **III** was reported.^29^ Only a cyclotrisilene **II**, a disilenide **III**, and a very recent cyclic disilene by Driess have been reported to actually activate CO (**IV**).^26,28,29^ Reactions of disilenes with isonitriles, which are isoelectronic to CO, afforded various products,^26,30–33^ among which compounds of type **V** are formed.^34–36^ Such silenes are structurally related to the products that we describe in this article (Chart 1).

**Chart 1:**
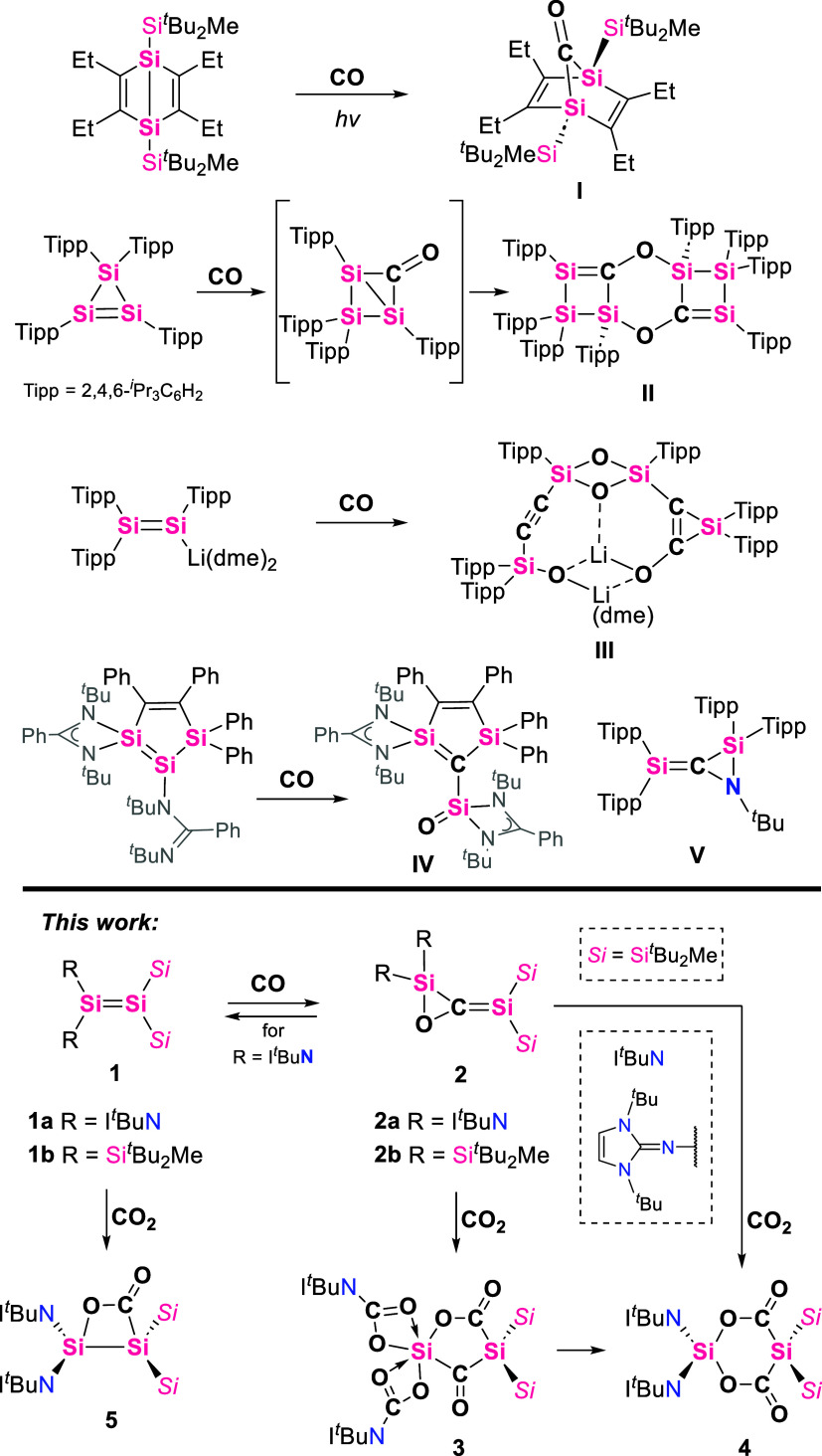
Reaction Yielding Disilyl Ketone I, Reaction Products of Disilenes with CO (II, III, IV), Reaction Product of Disilene with Isonitriles (V)

Herein, we report CO reactivity toward imino­(silyl)­disilene **1a** and tetra­(silyl)­disilene **1b**.^37,38^ CO inserts into the Si=Si bonds of these disilenes, affording compounds **2a** and **2b**, which can be described as 1,3-disilaallene oxides. DFT calculations suggest that both disilenes react via the same reaction mechanisms. The CO insertion is thermally reversible in the case of **2a**. The unique electronic structure of **2a** facilitates the reaction with CO_2_, affording disila-2,4-furandione **3** at low pressure and disila-bislactone **4** at higher pressures -a disilyl derivative of Meldrum’s acid. A plausible reaction mechanism was proposed and supported by quantum chemical calculations. For comparison, imino­(silyl)­disilene **1a** was reacted with CO_2_ to form the CO_2_-adduct **5**, whereas **5** cannot be transformed into **4**.

## Results and Discussion

To study the reactivity of R_2_Si=SiR_2_ with CO, we focused on disilenes with substituents that would ensure their enhanced lability. Substituents greatly impact the geometry, electronic structure, and, therefore, the reactivity of disilenes. Bulky silyl substituents lead to twisted geometries to minimize the steric repulsion, while π-donating substituents, e.g., N-heterocyclic imines (NHIs), result in *trans*-bent disilene structures.^38,39^ Imino­(silyl)­disilene **1a** (Scheme 1), previously reported by our group,^37^ shows *trans*-bent and twisted geometry, emphasizing the influence of silyl substituents and NHI substituents interacting in one system, which results in a polarized Si=Si double bond (*d*_Si=Si_ = 2.219(4) Å). The second disilene, whose reactivity toward CO we investigated, is the tetra­(trialkylsilyl)­disilene **1b** (Scheme 1), originally reported by Sekiguchi et al.^38^ **1b** bears four bulky *^t^* Bu_2_MeSi groups which cause an elongation of the central Si=Si bond (2.2598 Å) and a pronounced distortion around the silicon centers with a twisting angle of 54.5° and torsion angles of 48.8° and 62.9°. This leads to a small HOMO–LUMO gap and low Δ*E*_ST_.^38,40^ While the redox potential of this presumably reactive, albite sterically protected disilene **1b** has been investigated, its capability for small-molecule activation has not been tested.

**Scheme 1:**
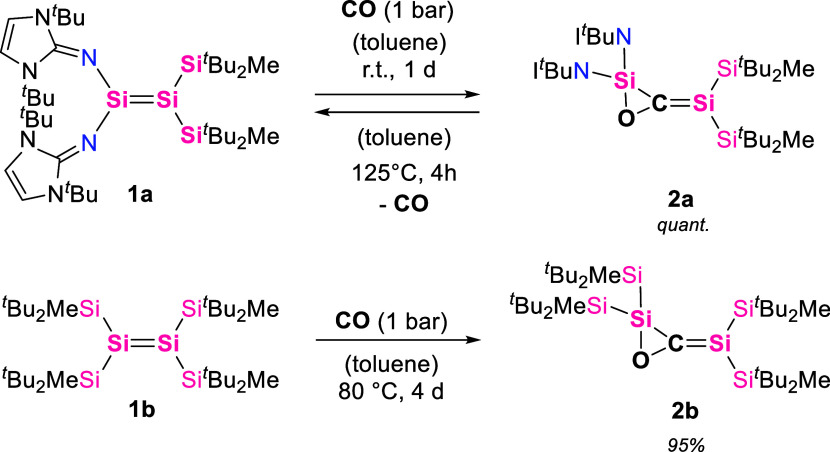
Reversible CO Insertion Reaction of Disilenes **1a** and **1b** to 1,3-Disilaallene Oxides **2a** and **2b**

When a toluene solution of **1a** was treated with 1 bar of CO at room temperature, the characteristic deep red color of **1a** faded after 1 day, giving a pale-yellow solution of a new species, which was later identified as the 1,3-disilaallene oxide **2a** (Scheme 1). ^1^H NMR spectroscopy shows two distinct sets of *^t^* Bu- and Me-signals of the silyl groups, pointing at the reduced symmetry of **2a** compared to **1a**. The Si_2_ *Si* atom in **2a** resonates at 10.1 ppm in the ^29^Si NMR spectrum and is in the range of reported resonances for related silenes (17.5 to 54.3 ppm).^41–43^ For comparison, Scheschkewitz et al. presented a cyclic silene with an oxygen substituent adjacent to the C^sp2^ center, which displays a comparable ^29^Si NMR shift at 17.5 ppm (*J* = 9.0 Hz).^43^ The N_2_Si atom resonates at −116.6 ppm (*J* = 95.4 Hz) in the ^29^Si NMR spectrum, which is high-field shifted compared to a previously reported 1-silaallene oxide (δ^29^Si_Si=C_ = −64.8 ppm).^44^ In the ^13^C NMR spectrum, the C^sp2^ (C=Si) signal can be observed at 231.1 ppm (*J*_1_ = 28.6 Hz, *J*_2_ = 47.7 Hz), which is within the typical range for silenes,^41^ but is downfield shifted from **V** (144.2 ppm)^36^ and a cyclic silene (182.8 ppm).^24^

Single-crystal X-ray diffraction (SC-XRD) of the pale-yellow crystals of **2a**, grown from a cooled *n*-hexane solution, reveals a monoclinic (*P*2_1_/c) crystal system. Structure refinement showed a whole-molecule rotational disorder and was modeled accordingly. The high R_1_ value (0.16) is due to mediocre crystal data quality. Despite numerous attempts, crystals of better quality could not be obtained. Nevertheless, the solid-state geometry of **2a** (Figure 1) was identified without a doubt and compares well to the optimized structure and **2b** (see Figure 1). The crystal structure of **2a** shows a central Si­(O)­C=Si moiety, composed of a Si–O–C three-membered ring, and the central carbon atom connected to another silicon center via a double bond. Compound **2a** is the first example of a 1,3-disilaallene oxide; silaaziridines (e.g., **V**),^34–36^ 1-silaallene oxide,^44^ and allene oxides^45^ are other comparable structures. The central Si=C bond distance of **2a** measures 1.761(13) Å, and for the optimized structure **2a**_**O**_, it is 1.754 Å (Figure 1). This is similar to a silene by Scheschkewitz (1.775(3) Å)^43^ and the Si=C bond distance (1.735(2) Å) of structurally related silaaziridine **V** (Chart 1).^36^ The Si–C=Si bond angle (Si1–C19–Si2) measures 168.9 (14)° for **2a**, while for **2a_O_**, an angle of 164.87° can be observed. Notably, both *^t^* Bu_2_MeSi groups and the three-membered silaoxirane ring are in-plane with the central Si=C atoms with bond angles of 48.0(5)° (**2a**, C19–Si1–O1) and 69.6(6)° (Si1–O1–C19). The NHI substituents are arranged perpendicular to this plane. The bond lengths and angles of the optimized structure **2a_O_** and **2a** compare well, only the twisting of the substituents has been optimized to 78.23° for **2a_O_**, which can be attributed to H-bonding of the Si*^t^* Bu_2_Me substituents to the oxygen atom.

**Figure 1:**
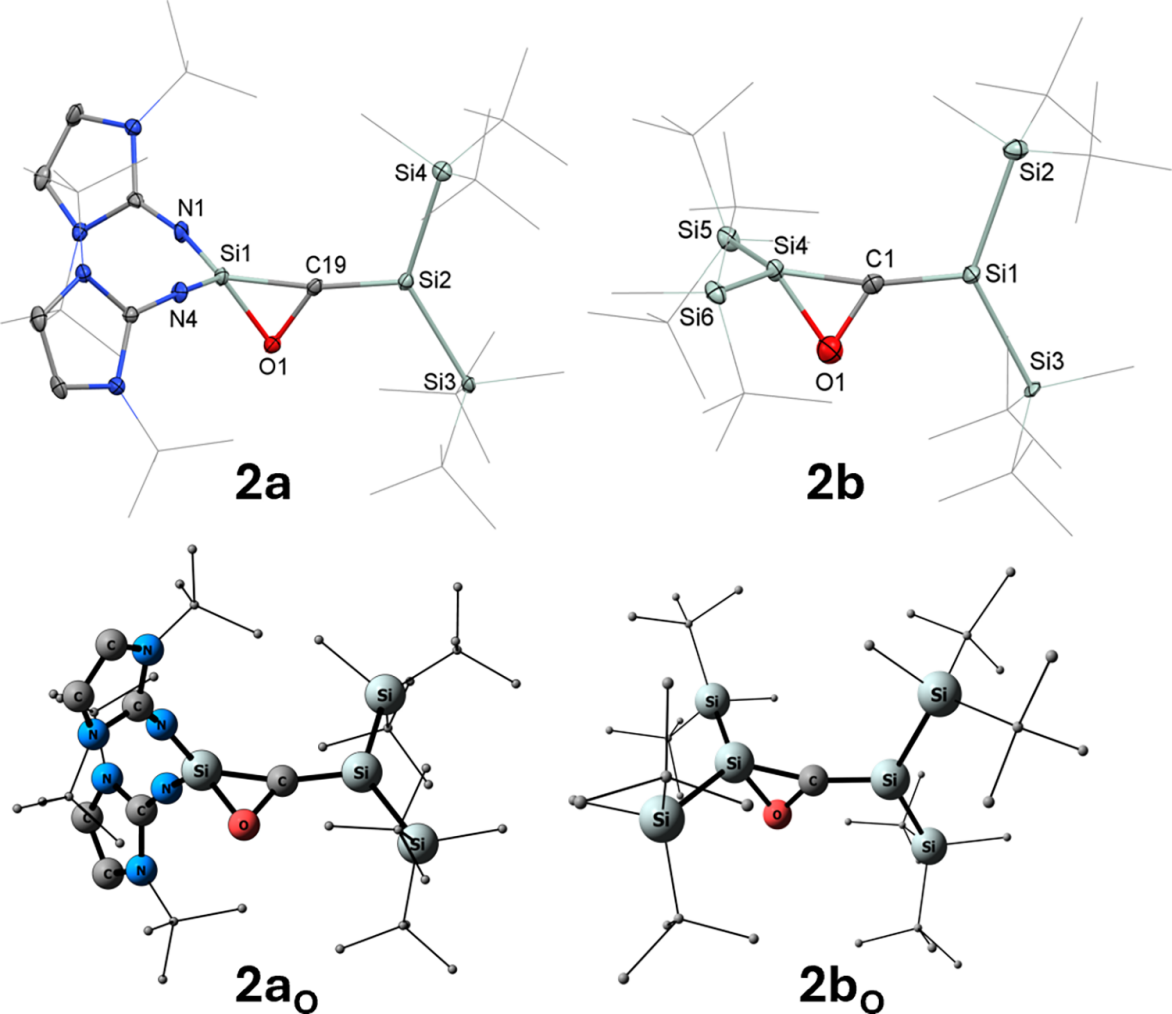
Molecular structure of **2a** (left, top) and **2b** (right, top) with thermal ellipsoids drawn at the 30% probability level. Hydrogen atoms are omitted for clarity, and *^t^* Bu- and Me-groups are simplified as wireframes. Selected bond lengths [Å] and angles [°]: **2a**: bond lengths: Si1–C19 1.799(11), C19–Si2 1.761(13), Si1–O1 1.700(10), and O1–C19 1.427(14); bond angles: O1–Si1–C19 48.0(5), O1–C19–Si1 62.3(6), Si1–O1–C19 69.6(6), Si1–C19–Si2 168.9(14); twist angle: 89.96° (N1, N4/Si3, Si4). **2b**: bond lengths: Si4–C1 1.781(10), C1–Si1 1.786(10), Si4–O1 1.727(12), and O1–C1 1.401(12); bond angles: O1–Si4–C1 47.0(4), O1–C1–Si4 64.5(6), Si4–O1–C1 68.5(6), Si4–C1–Si1 167.4(6); twist angle: 85.94° (Si5, Si6/Si2, Si3). Optimized structures **2a**_**O**_ (left, bottom) and **2b**_**O**_ (right, bottom). Selected bond lengths [Å] and bond angles [°]: **2a**_**O**_: Si1–C19 1.797, C19–Si2 1.754, O1–C19 1.432, Si1–C19–Si2 164.87°. **2b**_**O**_ (right, bottom). Selected bond lengths [Å] and bond angles [°]: **2b**_**O**_: Si4–C1 1.841°, C1–Si1 1.759°, O1–C1 1.385°, and Si4–C1–Si1 163.63°.

Quantum chemical calculations^46,47^ show that the electronic structure of **2b** is that of a 1,3-disilaallene oxide, consisting of a three-membered oxasilirane ring with an exocyclic Si­(Si*^t^* Bu_2_Me)_2_ moiety connected by a C–Si bond with pronounced double bond character (Figure 2) (WBI = 1.52, MBO = 1.76). In **2b**, NBO analysis (Figure 3) shows that in addition to the two lone pairs on the oxygen atom (see full analysis in SI, Figure S57), the central (Si–O–C)-Si unit contains a σ- and a π-bond between the carbon and the exocyclic silicon atom (NBO 73 and 74). The three single Si–O, Si–C, and O–C σ bonds form the three-membered ring (NBO 83, 84, and 91). The σ­(Si–O) bond is substantially polarized toward the oxygen atom.

**Figure 2:**
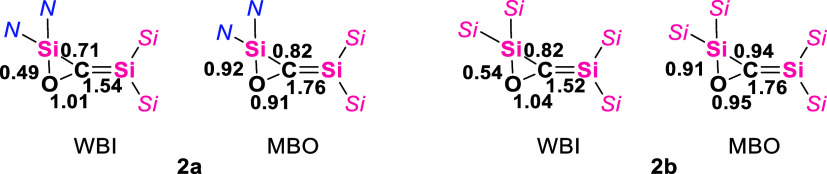
Wiberg bond indexes (WBI) and Mayer bond orders (MBO) of **2a** and **2b**.

**Figure 3:**
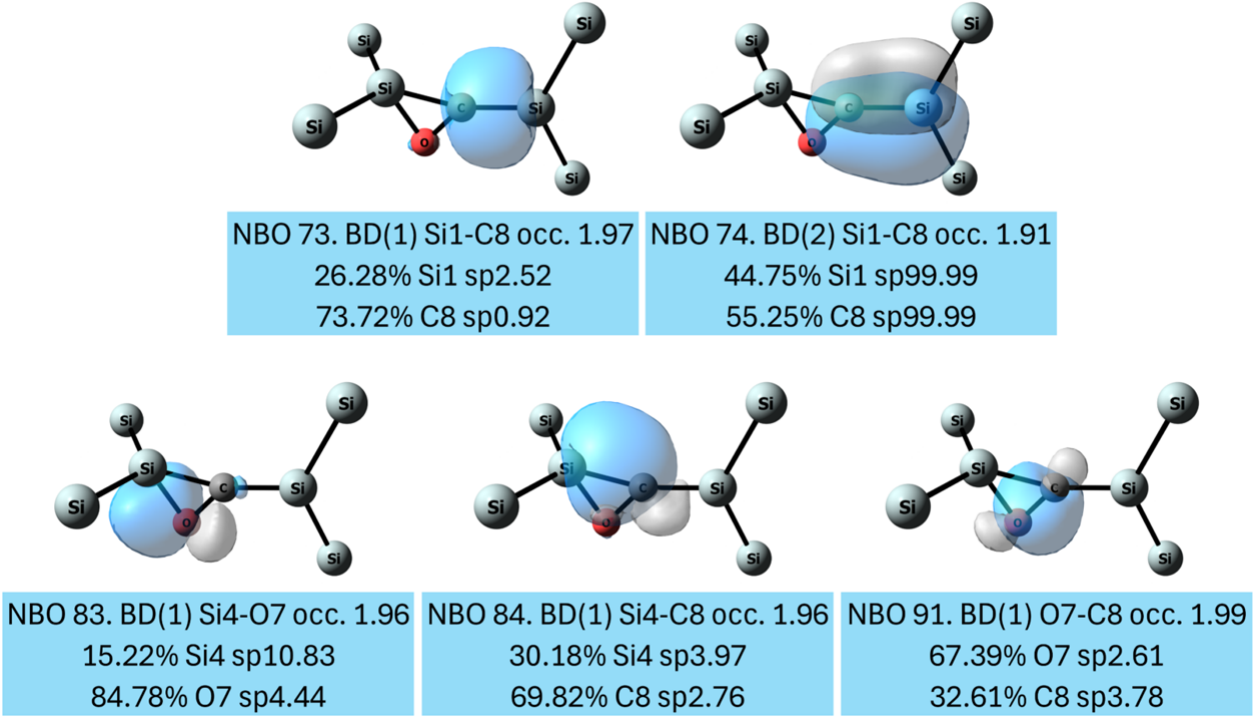
NBOs that describe the bonding within the (Si–O–C)-Si moiety of **2b**. Substituents are omitted for the sake of clarity.

The NBO depiction of the electronic situation in **2a** is slightly different (Figure 4). Besides the bonding interactions that constitute the three-membered ring (NBOs 77, 78, and 80), which are essentially similar to the NBOs 83, 84, and 91 in **2b**, there is an additional π bond between the carbon and oxygen (NBO 74). Furthermore, there is only a σ-bond between the carbon and the exocyclic silicon, and the π bond is missing. Instead, there is a very low occupancy (0.97 el) lone pair on the exocyclic silicon (NBO 69). The search for 3-center, 4-electron hyperbonds shows the ω-type bonding motif which is composed of the endocyclic O and C and the exocyclic Si centers and with occupancy of 3.85 electrons. The hyperbond exhibits a very strong (C–Si) bonding character of 83.8% and a weak O–C bonding character of 16.2%, which accounts for the low occupancy NBO of 69. Overall, similarly to **2b**, **2a** possesses a silene moiety with strong Si–C double bond character (WBI = 1.54, MBO = 1.76).

**Figure 4:**
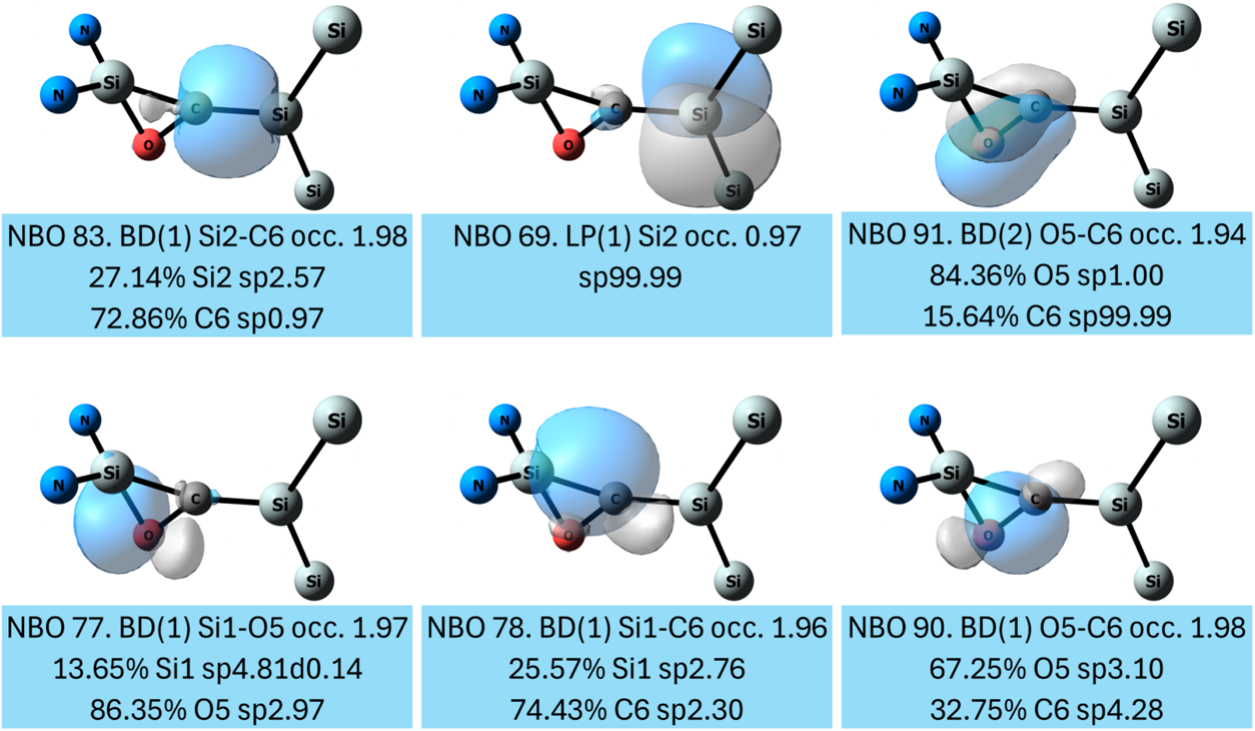
NBOs that describe the bonding within the (Si–O–C)-Si moiety of **2a**. Substituents are omitted for clarity.

To explore the mechanism of formation of **2a** and **2b**, we studied these reactions computationally (Figures 5 and 6). The addition of a CO molecule at the bis­(imino)-substituted Si center of the disilene gives **1a** as the intermediate **INT1a** (Figure 5). This type of donor–acceptor intermediate was postulated in the formation of **V**,^36^ as well as for the addition of isocyanides to Brook’s silene.^48,49^ **INT1a** rearranges to the cyclic disiliran-3-one species **INT2a** in which the carbon atom of the carbon monoxide moiety is bound to both silicon atoms of the disilene. A similar three-membered disilyl ketone was proposed as a reactive intermediate in the reactions of a cyclotrisilene with CO,^28^ and could later be trapped in the presence of a Lewis acid or base.^27^ Through a very low barrier **TS3a**, the disiliran-3-one **INT2a** rearranges to final product **2a**. The barrier for the reverse reaction is predicted to be 28.7 kcal mol^–1^, which is too high for ambient conditions, but is possible at elevated temperatures, such as in the experiment described in Scheme 2 (see below).

**Figure 5:**
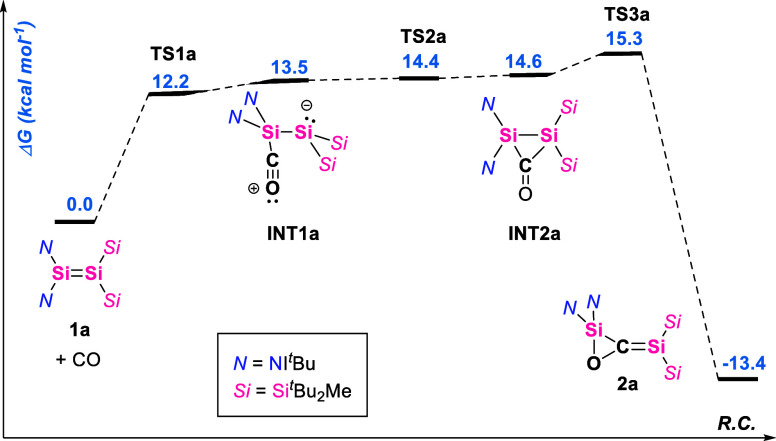
Calculated free energy diagram for the proposed reaction mechanism of **1a** with CO. Free energies at the (SMD = benzene)­PW6B95-D4/def2-QZVPP//r**^2^** SCAN-3c level of theory.

**Figure 6:**
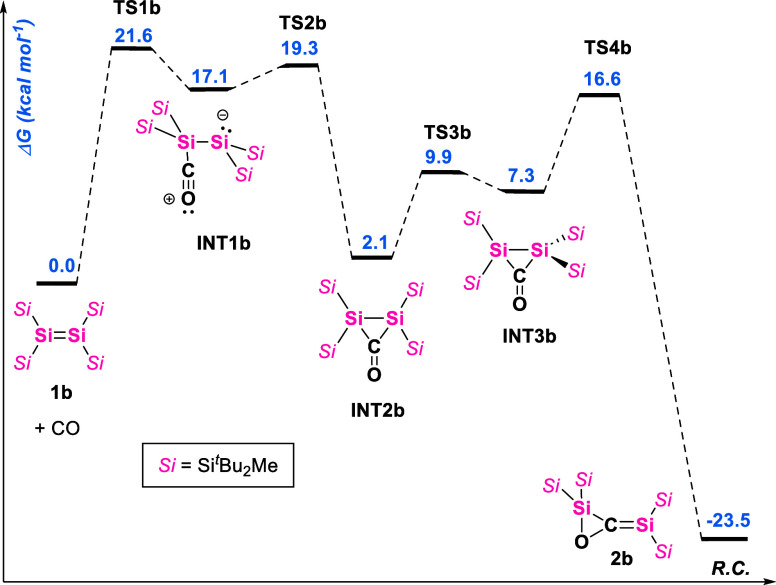
Calculated free energy diagram for the proposed reaction mechanism of reaction **1b** with CO. Free energies at the (SMD = benzene)­PW6B95-D4/def2-QZVPP//r**^2^** SCAN-3c level of theory.

**Scheme 2:**
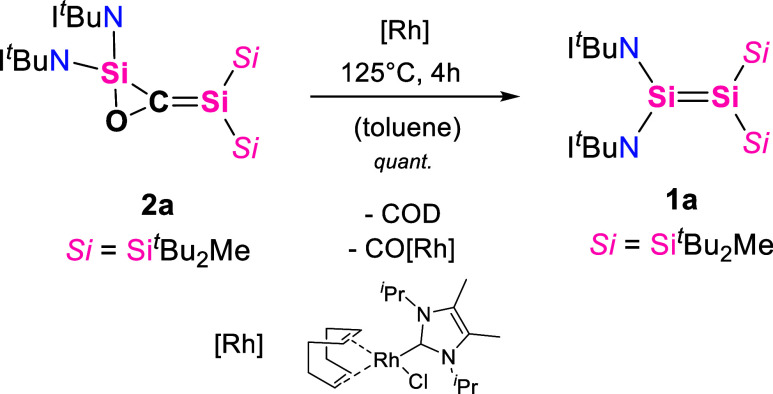
CO Release and Trapping from the Reaction of **2a** and the Regeneration of **1a**

The tetra­(trialkylsilyl)­disilene **1b** reacts with CO analogously to **1a**, forming the corresponding 1,3-disilaallene oxide **2b**, albeit at a lower rate than that of **1a**. When treating a toluene solution of tetra­(silyl)­disilene **1b** with 1 bar of CO, the characteristic deep blue color of **1b** changes to bright yellow after heating the reaction mixture at 80 °C for 4 days, yielding the **2b** product (Scheme 1). The reaction also proceeds at room temperature but requires about 6 weeks for full conversion. In the ^1^H NMR spectrum, differentiation between the two sets of *^t^* Bu- and Me-signals of the silyl groups is observable, because of the reduced symmetry of **2b** compared to **1b**. The C=*Si* atom in **2b** resonates at 14.1 ppm in the ^29^Si NMR spectrum, while the Si_2_Si­(CO) atom resonates at −50.0 ppm, comparable to **V** (δ^29^Si_Si=C_ = −80.2 ppm).^36^ In the ^13^C NMR spectrum of **2b**, the C^sp2^ signal was observed at 197.1 ppm. This is slightly upfield shifted in comparison to **2a** and downfield shifted in comparison to **V** (δ^13^C_Si=C_ = 144.2 ppm).^36^ Performing the reaction with labeled ^13^CO revealed the Si=C coupling constant (^13^C: 197.1 ppm: *J*_1_ = 17.3 Hz, *J*_2_ = 32.1 Hz and ^29^Si: -50.0 ppm: *J*_1_ = 33.8 Hz). The crystal structure of **2b** (Figure 1) shows a Si=C bond distance of 1.786(10) Å, which is comparable with the silene having a neighboring oxygen atom by Scheschkewitz and co-workers (1.775(3) Å).^43^ Notably, both *^t^* Bu_2_MeSi groups and the three-membered silaoxirane ring are in-plane with the central Si=C atoms with ring-bond angles of 47.0(4)° (C1–Si4–O1), 68.5(6)° (Si4–O1–C1), and 64.5(6)° (Si4–C1–O1). The opposing silyl substituents are arranged perpendicular to this plane. The crystal data of **2b** was analogously compared to the optimized structure **2b_O_** (Figure 1), and the data is in good agreement.

**1b** is proposed to react with CO via a similar mechanism (Figure 6). The donor–acceptor intermediate **INT1b** forms and rearranges to disiliran-3-one **INT2b**. In this case, the disiliran-3-one isomerizes to a conformation, which will allow the rearrangement to the final 1,3-disilaallene oxide product **2b**. The activation barrier for this reaction (**TS1b** at 21.6 kcal mol^–1^) is 6.3 kcal mol^–1^ higher than the **TS3a**. This is also reflected in the longer reaction times for the formation of **2b**.

To the best of our knowledge, **2a** and **2b** are the first isolated 1,3-disilaallene oxides. Other known and related functional groups are silaaziridines (e.g., **V**), derived from disilenes,^34–36^ or a silaallene oxide derived from a silylene.^44^ Interestingly, 1,3-disilaallenes (Si=C=Si) are elusive species, and while there are few examples of the related isolated 1-silaallenes (Si=C=C) reported, there are no reports of disilaallenes.^50^

The reversibility of the CO insertion in **2a** was indicated by variable-temperature NMR, showing a minor formation of **1a**, when **2a** was heated to 125 °C. To remove the released CO from the atmosphere, we introduced the previously reported rhodium complex as a CO trapping reagent.^51^ The reaction mixture was heated to 125 °C (Scheme 2), and after 4 h, a quantitative regeneration of **1a** was observed by color change and multinuclear NMR spectroscopy (^1^H, ^13^C, and ^29^Si NMR), showing that insertion of CO into the Si=Si bond of **1a** is thermally reversible. Similar studies were conducted on **2b**; however, no reversibility of the CO insertion could be observed. This is in line with the DFT calculations that show an insurmountable energy barrier of 45.1 kcal mol^–1^ for this transformation (Figure 6).

The disilaallene oxides can be viewed as an interaction between silylene and the silicon carbonyl complex (Figure 7). Calculations show that in **2a** this interaction is much weaker (Δ*E* = 36.4 kcal mol^–1^ vs 51.7 kcal mol^–1^ in **2b**), presumably due to the enhanced ability of bis­(NHI)-substituted silylene to retain the (II) oxidation state in comparison to bis­(silyl)­silene. The weaker bonding is also reflected in the smaller WBIs and MBOs corresponding to the endocyclic Si–C and C–O bonds in **2a** (Figure 2). These considerations account for the formation of **2a** being less exergonic than that of **2b** by 10.1 kcal mol^–1^ (energy decomposition analysis in TableS3, SI). The smaller energy gain in the formation of **2b** is the key to the reaction reversibility. Additionally, the weaker bonding of the silylene unit in **2a** is the reason for the ability of NHI-substituted disilaallene oxide to react with CO_2_ as we describe below.

**Figure 7:**
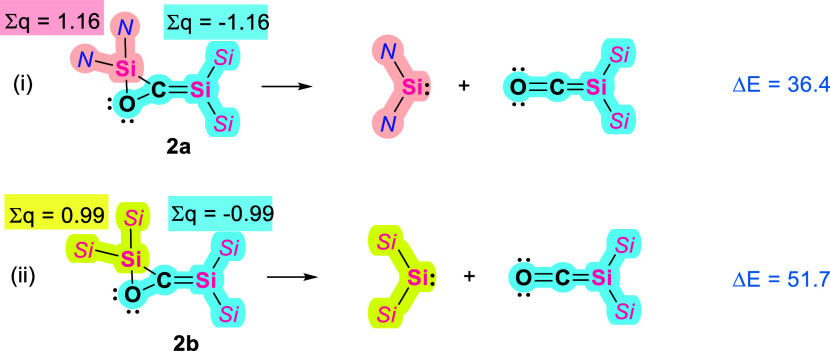
Calculated energies for the interaction between the silylene and the silicon carbonyl moieties in **2a** and **2b**.

After establishing the extraordinary reversible addition of CO to disilene **1a**, we set out to explore further the reactivity of the newfound 1,3-disilaallene oxide **2a** (Scheme 3). Our investigations revealed that pressurizing a benzene solution of **2a** with CO_2_ leads to the formation of a new compound, which was later identified as **4** (Scheme 3, path a). The ^13^C NMR spectrum of the reaction mixture exhibits a signal at 185.7 ppm (C=O). Other ^13^C NMR chemical shifts for C=O moieties of silyl carboxylates can be found at 185.9 ppm for **VI**,^52^ and 157.9 ppm for **VII** ^53^ (Scheme 3). The ^29^Si NMR shift for the central silicon atom SiSi_2_ was detected at −79.0 ppm, and the central SiN_2_ moiety resonates at −147.1 ppm. These values are expectedly slightly downfield shifted from those of **3**. When a toluene solution of **4** was cooled to −35 °C for several weeks, crystals suitable for SC-XRD could be obtained. In the X-ray structure of **4**, the two central silicon atoms are bridged by two CO_2_ moieties, featuring a slightly distorted 6-membered ring (Figure 8). The NHI and silyl substituents are in one plane perpendicular to the plane of the two CO_2_ molecules.

**Figure 8:**
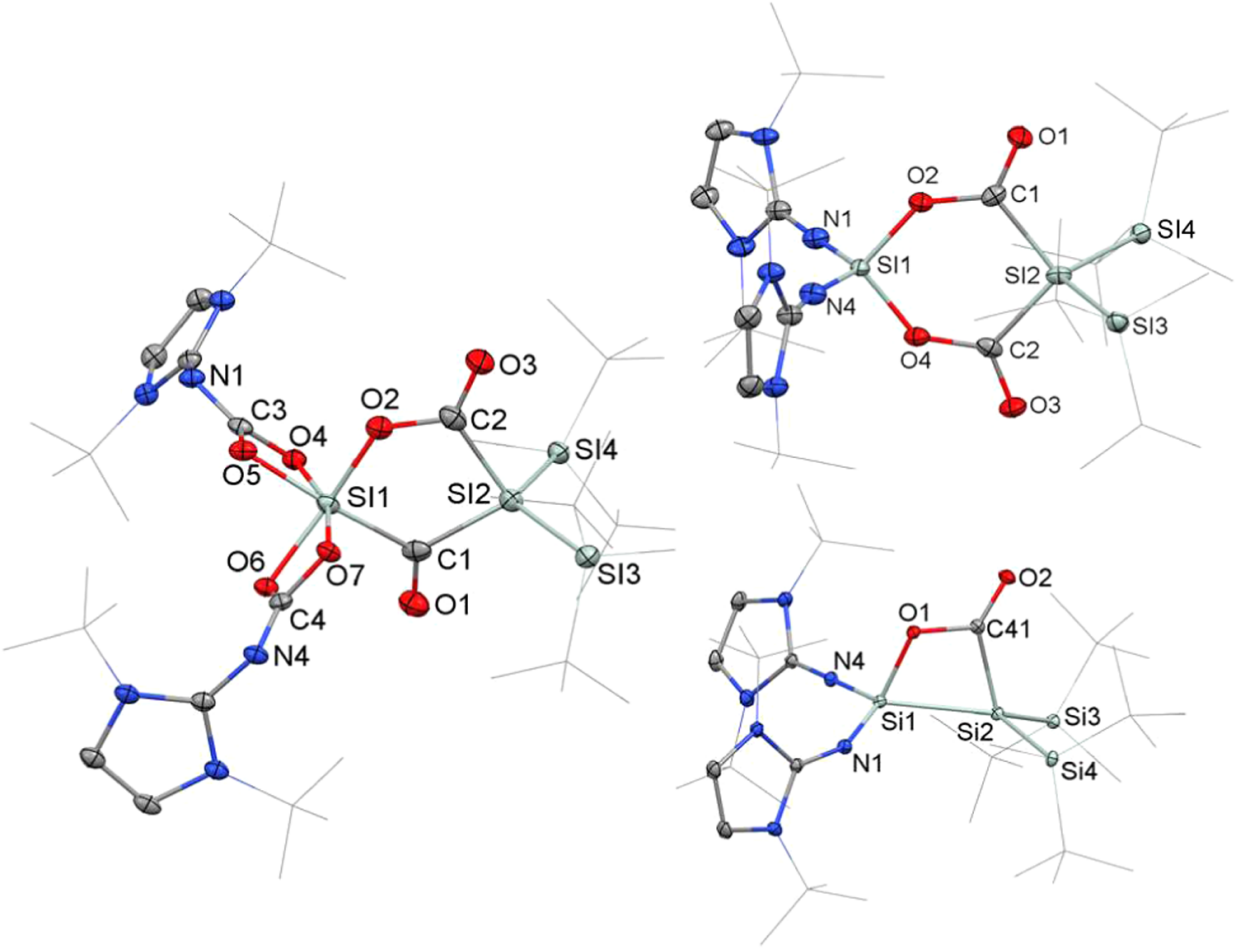
Molecular structure of **3** (left), **4** (top right), and **5** (bottom right) with thermal ellipsoids drawn at the 30% probability level. Hydrogen atoms are omitted for clarity; *^t^* Bu- and Me-groups are simplified as wireframes. Selected bond lengths [Å] and angles [°]: 3: bond lengths: C1–O1 1.203(3), C2–O2 1.313(4), C2–O3 1.217(4), Si1–C1 1.925, Si2–C1 1.992(3), Si1···Si2 3.239, Si2–Si3 2.420(3), and Si2–Si4 2.384(8). Bond angles: Si1–C1–Si2 111.56°. **4**: bond lengths: C1–O1 1.234(4), C1–O2 1.302(4), Si2–C1 1.917(3), Si1–O2 1.682(3), Si1–N1 1.609(4), Si1–N4 1.614(3), Si2–Si3 2.411(1), Si2–Si4 2.433(7), and Si1···Si2 3.547. Bond angles: O2–Si1–O4 102.7(1), C1–Si2–C2 101.9(1). **5**: Si1–Si2 2.4303(7), Si1–O1 1.744(1), Si1–N1 1.663(1), Si1–N4 1.646(1), Si2–Si3 2.4163(6), Si2–Si4 2.4248(7), Si2–C41 1.962(1), O1–C41 1.371(2), O2–C41 1.207(1). Bond angles: Si2–Si1–O1 76.53(4), Si1–Si2–C41 69.39(4), Si1–O1–C41 108.01(8), Si2–C41–O1 103.37(9), Si2–C41–O2 136.1(1), O1–C41–O2 120.5(1), N1–Si1–N4 115.28(7), and Si3–Si2–Si4 117.38(2). Twist angle (O1–C41/Si1-Si2) 13.1°, twist angle (N1–S1–N4/Si3–Si2–Si4): 45.2°.

**Scheme 3:**
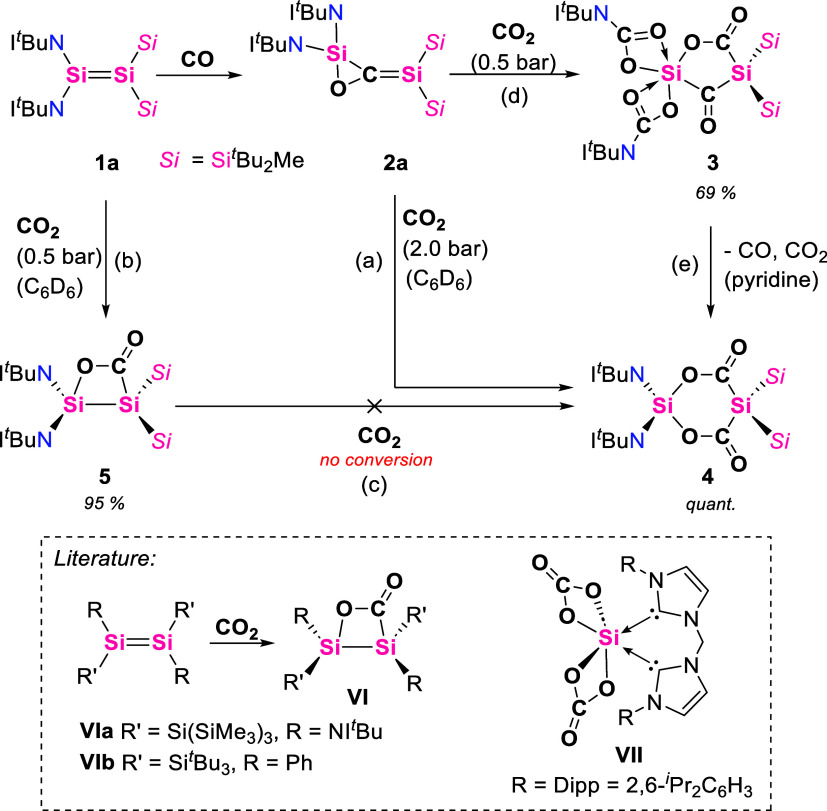
Reactivity of 1,3-Disilaallene Oxide **2a** with CO**_2_**, Forming Disila-2,4-furandione **3** (Top Right) and Disila-Bislactone **4** (Bottom Right)^a^

a Reaction of imino­(silyl)­disilene **1a** with CO_2_ to form CO_2_-adduct **5** (bottom left). Reported reactions of disilenes with CO_2_ **VI** and silicon dicarbonate **VII**.

Our initial assumption about the formation of **4** from **2a** was that it proceeds via the CO release from **2a** to reform the disilene **1a**, which consequently reacts with two molecules of CO_2_ to form the double addition product disila-bislactone **4**. However, we kept in mind that the barrier for regeneration of disilene is quite high. In any case, we tested this assumption by pressurizing a benzene solution of **1a** with 1.0 bar CO_2_. The red solution immediately loses its color, and a colorless solution is obtained. Full characterization with multinuclear NMR spectroscopy, mass spectroscopy, and SC-XRD revealed the formation of compound **5** (Scheme 3, path b), which is a product of a single [2 + 2] cycloaddition of CO_2_ across the Si=Si bond. Examples of doubly bonded silicon species such as silenes (Si=C),^54^ sila-imines (Si=N),^55^ and silanones (Si=O)^56^ have been reported to react in a [2 + 2] cycloaddition, forming structure motifs such as compound **5**. For disilenes capable of CO_2_ fixation analog to compound **5**, there is one example of an iminosilyldisilene in 2018 (**VIa**)^52^ and one alkylsilyldisilene in 2002 (**VIb**).^31^ For CO_2_-addition product **5** though, ^13^C NMR spectroscopy revealed a signal of 190.6 ppm for the C=O moiety. The ^29^Si NMR signals at −57.2 (SiSi_2_) and −87.5 ppm (N_2_ Si) could be assigned to the central silicon atoms. These signals are in line with chemical shifts reported for the disilene-CO_2_ addition reaction reported in 2018 (δ­(^29^Si): −35.6 ppm and −37.1 ppm).^52^ The SC-XRD measurement reveals a [2 + 2] cycloaddition of the CO_2_ to the Si=Si double bond, generating a slightly twisted 4-membered ring. The Si1–Si2 bond measures 2.4303(7) Å and is in line with values for **VIa** (Si–Si: 2.407(2) Å).^52^ For **VIb** reported by Wiberg et al., no SC-XRD data was provided.^31^ The C41–O1 bond distance measures 1.371(2) Å, which is elongated compared to **VIa** (*d*_C=O_ = 1.194(8) Å).^52^

The formation of **5** is calculated to be exergonic by 20.0 kcal mol^–1^ and proceeds via a concerted [2 + 2] cycloaddition with a low barrier of only 16.3 kcal mol^–1^ (Figure S49). However, conversion of **5** to the disila-bislactone **4** could not be achieved, even when the reaction mixture was heated to 60 °C for 2 days in a CO_2_ atmosphere, after which decomposition could be detected (Figure S50). This is in line with quantum chemical calculations, which suggest that the formal [2 + 2] cycloaddition **5** with CO_2_ to form **4** would be impossible under the experimental conditions due to the insurmountable barrier of 42.8 kcal mol^–1^ (Figure S49).

Thus, these results suggest that the formation of **4** from **2a** is not achieved by the release of the CO molecule and the consecutive reaction of disilene **1a** with CO_2_. This is because even if the regeneration of the disilene could be achieved, it would only react with a single CO_2_ molecule (Scheme 3, path b) since the reaction with the second CO_2_ molecule (Scheme 3, path c) is kinetically impossible. **4** cannot form from **2a** by the reversible CO release, as the barrier for this pathway is too high.

In order to understand the mechanism and isolate a possible reaction intermediate, we reacted **2a** with 0.5 bar of CO_2_ in a benzene solution, where a rapid color change from pale yellow to translucent pink could be observed. From this pink solution, **3** precipitates at room temperature as crystals suitable for SC-XRD over a period of 1 day. Once precipitated, **3** is soluble only in pyridine. Upon full characterization of **3** via multinuclear NMR spectroscopy, FT-IR, UV–vis, and SC-XRD, the formation of a 5-membered ring containing a disila-2,4-furandione was identified. ^13^C NMR spectroscopy revealed signals at 315.5 ppm for the Si_2_C=O moiety and 192.9 ppm for the O–C=O moiety in the 5-membered ring. ^13^C NMR signals for CO_2_ molecules inserted into the N–Si bond at the hexacoordinate silicon atom can be observed at 164.7 ppm. The ^29^Si NMR spectroscopy showed signals at −171.5 ppm (Si_hexacoordinate_) and −85.7 ppm (SiSi_2_) for the two central Si atoms. For comparison, reviews on hexa- and hypercoordinated silicon complexes are available.^57–59^ An experiment with labeled ^13^CO_2_ revealed the Si_2_CO moiety of **3** to originate from 1,3-disilaallene oxide **2a**. When preparing **3** with ^13^CO_2_, signals in the ^13^C NMR spectrum at 193.2 and 165.0 ppm show a strong intensity, while the Si_2_C=O signal at 315.5 ppm retains its low intensity. SC-XRD analysis revealed a carbonyl moiety with a C=O bond distance of 1.203(3) Å and a Si1–C1–Si2 bond angle of 111.56°. Both values are higher than those for disilyl ketone **I** (C=O: 1.199(2) Å, Si–C–Si: 93.26°),^23^ which is owed to the more rigid structure of **I** and its stabilization by its disila-dewar benzene structure. The silicon atom Si1, formerly bound to two NHI substituents, is now hexacoordinate surrounded by five oxygen atoms and one carbon atom with the carbon monoxide inserted into the NHI-Si bond, presenting an octahedral geometry around Si1. The group of Driess reported a related hexacoordinate bis-NHC-supported silicon dicarbonate species **VII** (Scheme 3), but this is a product of a silylone reacting with CO_2_.^53^

To understand the rearrangement reaction of **3** to **4** (pathway e, Scheme 3) and the reaction of **2a** to **4** (pathway a, Scheme 3), we performed reactions with labeled carbon dioxide (**2a** → **3** → **4**, pathways d and e) and labeled carbon monoxide (**2a** → **4**, pathway a). The formation of **3** from **2a** (pathway d) occurs only when using a low amount of pressure for CO_2_ (0.5 bar). There, three molecules of CO_2_ insert, which is calculated to be exergonic by 28.3 kcal mol^–1^. **3** precipitates after 2 days as pink crystals from the reaction mixture (benzene) in the atmosphere of 0.5 bar of CO_2_. These crystals were used to identify **3** via SC-XRD (Figure 8, left). Additional investigations with labeled ^13^CO_2_ (pathway d) lead to the conclusion that the SiO­(CO)Si originates from CO inserted into **2a**, while the remaining carboxylate groups originate from the labeled ^13^CO_2_. This can be seen in the higher intensity of the carboxylate signals (192.9 and 165.0 ppm) in the ^13^C NMR spectrum (Figure S26). Subsequent rearrangement from **3** to **4** (pathway e) can occur by exchanging the solvent with pyridine (1 day). The **3 → 4 +** CO + CO_2_ transformation is exergonic by an additional 12.9 kcal mol^–1^. In the case of the rearrangement of **3** to **4** (pathway e), we propose that the two hexacoordinate carboxylate groups dissociate as in the case of Wilson et al., where the insertion of CO_2_ into the NHI-Si bond and its subsequent dissociation of CO_2_ was shown.^60^ Then, the now liberated CO_2_ oxygenates the carbonyl moiety (Si_2_C=O), leading to the disila-bislactone species **4**.

For the selective formation of **4** from **2a** (pathway a), a CO_2_ pressure of 2.0 bar is necessary (C_6_D_6_, rt, 1 day). When decreasing the pressure to 1.6 bar of CO_2_, a mixture of **3** and **4** forms (Figure S42). This was underlined by labeled experiments ^13^CO, (pathway a), where the signal for **3**’s disilyl ketone Si_2_C=O moiety (315.5 ppm) converts to the C=O signal for **4** (192.2 ppm) (Figure S42). Thus, this proved that the carbon from the carboxylate of **4** originates from the CO inserted into **2a**.

To rationalize the formation of **3** and **4** from **2a** in the presence of CO_2_, we propose a mechanism presented in Figure 9. According to our calculations, the dissociation of **2a** into the bis­(NHI)­silylene (**INT3a**) and the silicon carbonyl complex (**INT4a**) (Figure 9, path a) is endergonic by only 15.4 kcal mol^–1^ and can take place in ambient conditions. Both intermediates have been reported before by our group, NHI-substituted germylene and stannylene,^61^ and a silyl-substituted carbonyl complex.^21^ In the presence of CO_2_, the silylene can react to form the CO_2_ complex **INT5a** (Figure 9, path b)—this type of silylene reactivity toward CO_2_ has also been previously reported.^55,62^ At this stage, the C–O bond of the CO_2_ can be cleaved via a low barrier forming a CO coordinated silanone **INT6a** (Figure 9, path c),^53,63^ which can react with the silicon carbonyl complex to form the **INT7a** in a very exergonic and irreversible step (Figure 9, path d). Consecutive insertion of two CO_2_ molecules across the Si–N^NHI^ bond will form **INT8a** and **3**, respectively (Figure 9, paths e and f). Similar reactivity of CO_2_ insertion into E-NHI bonds has been previously reported.^60^ The key in this process is the relative stability and abundance of **INT6a**, which can easily lose the coordinating CO to form free silanone **INT9a** (Figure 9, path g). The silanone can react with the silicon carbonyl complex forming **INT10a** (path h), which can further react with CO_2_ forming the final product **4** (path h). Due to low barriers, **INT6a** + **INT4a ⇌ INT9a** + **INT4a ⇌ INT10a** are expected to be in thermal equilibrium. The competing irreversible steps are path (d) (at Δ*G* = 6.0 kcal mol^–1^), which ultimately leads to the formation of **3** and path (i) (at Δ*G* = 7.2 kcal mol^–1^). The small ΔΔ*G* ^‡^ of 1.2 kcal mol^–1^ implies that higher CO_2_ pressure may facilitate the reaction with **INT10a** (path i), to form the final product **4**. This mechanism also explains the retention of the carbon that originates from the initial reaction of **1a** with carbon monoxide (marked in bold in Figure 9), as has been shown by the isotope labeling experiments. The disila-bislactone **4** can be viewed as a disilyl derivative of Meldrum’s acid depicted in Figure 10. Meldrum’s acid derivatives form ketenes and acetone via pyrolysis, during which CO_2_ is released. Interestingly, this is exactly the reverse process of formation of **4** (Figure 9), which forms from silicon carbonyl complex—the analogue of ketene, silanone—the analogue of ketone, and CO_2_.

**Figure 9:**
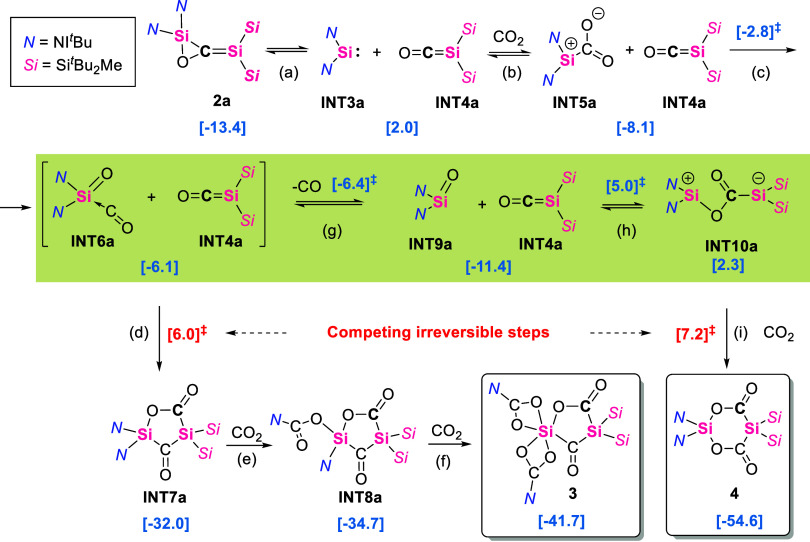
Proposed mechanism for the formation of **3** and **4** from **2a** in the presence of CO**_2_**. Free energies relative to **1a** at the (SMD = Benzene)­PW6B95-D4/def2-QZVPP//r**^2^** SCAN-3c level of theory are shown in brackets.

**Figure 10:**
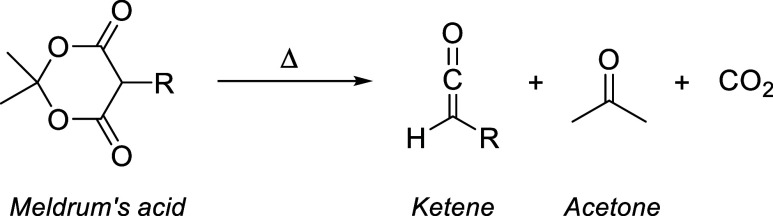
Literature-known pyrolysis of Meldrum’s acid.^64^

The reactivity of disilene **1b** and 1,3-disilaallene oxide **2b** toward CO_2_ was also tested. However, even with increased pressure (2 bar) and elevated temperatures (up to 140 °C), no reaction could be observed in either case. According to the proposed mechanism in Figure 9, the key step of the reactivity of the 1,3-disilaallene oxide **2a** with CO_2_ is its dissociation to the bis­(NHI)­silylene **INT3a** and the silicon carbonyl complex **INT4a**. While for **2a** this step is calculated to be endergonic by only 15.4 kcal mol^–1^, in the case of **2b**, this would require 31.5 kcal mol^–1^. Additionally, other reactivity pathways such as the insertion of CO_2_ into the Si-R bond are not taking place in **2b**, as it would involve the insertion of CO_2_ molecules into the Si–Si bond, which is unprecedented, unlike the insertion of CO_2_ into the tetrel-NHI bond.^60^ We also calculated that although the reaction of disilene **1b** with CO_2_ to form the analogue of **5** is exergonic (by 9.2 kcal mol^–1^), the barrier for this reaction would be 37.5 kcal mol^–1^, which is unachievable under the experimental conditions.

## Conclusions

In summary, we isolated and fully characterized hereto unknown 1,3-disilaallene oxides **2a** and **2b**, which result from rare CO insertion into the Si=Si bonds of disilenes. In the case of imino­(silyl)­disilene **1a**, the CO insertion is only exergonic by 13.4 kcal mol^–1^ and, thus, remarkably, is fully reversible in the presence of a CO trapping agent. Further reactivity of **2a** with CO_2_ was proven to occur via disila-2,4-furandione formation **3**, which rearranges to the disila-bislactone **4**. The products of CO_2_ insertions were compared to those of the single CO_2_-addition **5**. We exemplify novel reactivity patterns facilitated by reactive intermediates to produce silicon scaffolds. In this case, initial reaction with CO opened the door to further reactivity. In general, it might be worthwhile to look at the possibility of carrying out low-valent main-group reactions under CO atmosphere. This article exemplifies how the initial reaction with CO may form a reactive intermediate, which can react further with small molecules differently from the initial low-valent compounds, giving access to novel reactivity patterns.

## Supplementary Material



## Notes

Full experimental procedures; NMR spectra; SC-XRD structures; LIFDI-MS spectra; UV–vis spectra; and FT-IR spectra (PDF)
