# Supplementary material for: Reversible CO Insertion into the Si = Si Double Bond Enables a Disila-Bislactone Formation via Subsequent CO2 Addition
Source: J Am Chem Soc. 2025 Jul 21;147(30):26663–73. doi: 10.1021/jacs.5c07040 (PMC12314906; doi:10.1021/jacs.5c07040)
Supplement: Supplementary file 1 [file ja5c07040_si_001.pdf]

Supporting Information

# Reversible CO Insertion into the Si=Si Double Bond Enables A Disila-bislactone Formation via Subsequent $CO_2$ Addition

*Fiona J. Kiefer,[1] Arseni Kostenko,[1] Richard Holzner,[1] and Shigeyoshi Inoue[1*]*

[1]TUM School of Natural Sciences, Department of Chemistry, Institute of Silicon Chemistry and Catalysis Research Center, Technische Universität München, Lichtenbergstraße 4, 85748 Garching bei München, Germany. *E-mail: s.inoue@tum.de

## Table of Contents


1. Experimental Section 3

1.1. General Methods and Instrumentation ....................3

1.2. 1,3-disilaallene oxide 2a ....................5

Synthesis of silene 2a with labelled $^{13}CO$ ....................5

1.3 CO Trapping of 2a ....................11

1.4 1,3-disilaallene oxide 2b ....................14

Synthesis of 2b with labeled $^{13}CO$ ....................15

Explanation for the difference in energy profiles for the formation of 2a and 2b: ....................22

1.5 Hexacoordinate disilyl ketone intermediate: disila-2,4-furandione 3 ....................24

Synthesis of 3 using labeled $^{13}CO_2$ gas ....................25

1.6 Double $CO_2$ insertion product: disila-bislactone 4 ....................31

1.7 Proposed mechanism for the formation of 4 ....................35

1.8 Disilene $CO_2$ adduct 5 ....................37

2. X-ray Crystallographic Data 42

2.1. General Information ....................42

2.2 SC-XRD structures ....................44

2.3 Crystal data and structural refinement parameters ....................47

3 Computational Details 50

4 References 86

# 1. Experimental Section

## 1.1. General Methods and Instrumentation

All manipulations were carried out under exclusion of water and oxygen under an atmosphere of argon 4.6 (≥99.996%) using standard Schlenk and glovebox techniques. The glassware used was heat dried under high vacuum prior to use. All solvents were refluxed over sodium/benzophenone, freshly distilled under argon and deoxygenated prior to use. PTFE-based grease (*Triboflon III* from *Freudenberg & Co. KG*) was used as sealant. Deuterated benzene ($C_6D_6$) was obtained from Sigma-Aldrich, dried over Na/K alloy, flask-to-flask condensed, deoxygenated by three freeze-pump-thaw cycles and stored over 4 Å molecular sieves in a glovebox. All NMR samples were prepared under argon in *J. Young* PTFE valve NMR tubes. The nuclear magnetic resonance spectra (NMR) were recorded on a *Bruker DRX400* ($^1$H: 400.13 MHz, $^{13}$C: 100.62 MHz, $^{29}$Si: 79.49 MHz), AV500 ($^1$H: 500.13 MHz) or *AV500C* ($^1$H: 500.36 MHz, $^{13}$C: 125.83 MHz, $^{29}$Si: 99.41 MHz) spectrometer at ambient temperature (300 K), unless otherwise stated. The $^1$H, $^{13}$C{$^1$H} and $^{29}$Si{$^1$H} NMR spectroscopic chemical shifts δ are reported in ppm relative to tetramethylsilane. $^1$H and $^{13}$C{$^1$H} NMR spectra are calibrated against the residual proton and natural abundance carbon resonances of the respective deuterated solvent as internal standard ($C_6D_6$: δ($^1$H) = 7.16 ppm and δ($^{13}$C) = 128.1 ppm, Tol-$d_8$: δ($^1$H) = 2.08 ppm and δ($^{13}$C) = 20.4 ppm).[1] The following abbreviations are used to describe signal multiplicities: s = singlet, d = doublet, dd = doublet of doublets, dt = doublet of triplets, t = triplet, sept = septet, m = multiplet, br = broad. In some NMR spectra, signals from silicone grease ($C_6D_6$: δ($^1$H) = 0.29 ppm, δ($^{13}$C) = 1.4 ppm and δ($^{29}$Si) = -21.8 ppm), originating from the cannulas used (*B. Braun Melsungen AG Sterican®)*, can be observed. UV/VIS spectra were recorded on a *Varian, Inc. Cary 50* spectrophotometer with a Schlenk quartz cuvette. Quantitative elemental analyses (EA) were measured with a *EURO EA (HEKAtech)* instrument equipped with a CHNS combustion analyzer at the *Laboratory for Microanalysis* at the *TUM Catalysis Research Center*. Liquid Injection Field Desorption Ionization Mass Spectroscopy (LIFDI-MS) was measured directly from an inert atmosphere glovebox with a Thermo Fisher Scientific Exactive Plus Orbitrap equipped with an ion source from Linden CMS.[2] Melting Points (m.p.) were determined in sealed glass capillaries under inert gas by a *Büchi M-565* melting point apparatus. Infrared (IR) spectra were recorded on a Perkin Elmer FT-IR spectrometer (diamond ATR, Spectrum Two) in the range of 400–4000 cm$^{-1}$ at room temperature under an argon atmosphere. IR intensity bands are abbreviated as s = strong, m = medium, and w = weak. Unless otherwise stated, all commercially available chemicals were purchased from *abcr GmbH* or *Sigma-Aldrich* and used without further

purification. Carbon monoxide (CO) 4.7 (≥99.997%) was purchased from *Westfalen AG* and used as received. The compounds $(I^{t}BuN)_2Si{=}Si(Si^{t}Bu_2Me)_2$ [3] and $(^{t}Bu_2MeSi)_2Si{=}Si(Si^{t}Bu_2Me)_2$ [4] were prepared as described in the corresponding references.

## 1.2. 1,3-disilaallene oxide 2a

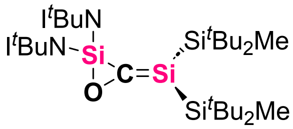

**2a**

$Si_4C_{41}H_{82}N_6O$

787.49 g/mol

A solution of disilene **1a** (40.0 mg, 52.2 µmol) in toluene (4 mL) in a pressurizable Schlenk flask was degassed in one freeze-pump-thaw cycle. After exposure to carbon monoxide (1.3 bar) at room temperature, the red color vanished within 1 hour to give a clear, colorless solution. All volatiles were removed in vacuo, and compound **2a** was obtained as a colorless solid (41.4 mg, 52.2 µmol, quant.). Crystals, suitable for SC-XRD analysis were obtained from a cooled (-35 °C) *n*-hexane solution of **2a**.

**$^1$H NMR (500 MHz, $C_6D_6$, 300 K):** δ [ppm] = 5.99 (s, 4H, C<u>*H*</u>-N), 1.55 (s, 36H, <u>*$^t$Bu*</u>N), 1.41 (s, 18H, <u>*$^t$Bu$_2$*</u>MeSi), 1.34 (s, 18H, <u>*$^t$Bu$_2$*</u>MeSi), 0.52 (s, 3H, $^t$Bu$_2$<u>*Me*</u>Si), 0.42 (s, 3H, $^t$Bu$_2$<u>*Me*</u>Si).

**$^{13}$C{$^1$H} NMR (126 MHz, $C_6D_6$, 300 K):** δ [ppm] = 231.4 (<u>*C*</u>O), 139.6 (<u>*C*</u>=N), 107.5 (<u>*C*</u>H-N), 55.1 (N<u>*C*</u>(CH$_3$)$_3$), 30.4 (Si(C(<u>*C*</u>H$_3$)$_3$)$_2$(CH$_3$)), 30.3 (Si(C(<u>*C*</u>H$_3$)$_3$)$_2$(CH$_3$)), 28.9 (NC(<u>*C*</u>H$_3$)$_3$), 22.0 (Si(<u>*C*</u>(CH$_3$)$_3$)$_2$(CH$_3$)), 21.3 (Si(<u>*C*</u>(CH$_3$)$_3$)$_2$(CH$_3$)), -4.8 (Si(C(CH$_3$)$_3$)$_2$(<u>*C*</u>H$_3$)), -4.8 (Si(C(CH$_3$)$_3$)$_2$(<u>*C*</u>H$_3$)).

**$^{29}$Si{$^1$H} NMR (99 MHz, $C_6D_6$, 300 K):** δ [ppm] = 16.8 ($^t$Bu$_2$Me<u>*Si*</u>), 13.9 ($^t$Bu$_2$Me<u>*Si*</u>), 10.1 (C=<u>*Si*</u>Si$_2$), -116.6 (<u>*Si*</u>N$_2$).

**FT-IR** [cm$^{-1}$]: 3170 (w, $^t$Bu), 2972 (w, $^t$Bu), 2931 (w, $^t$Bu), 2853 (w, $^t$Bu), 2080 (w, Si=C), 1647 (w), 1363 (s), 1223 (s), 970 (s).

**m.p:** 106°C

**EA:** $Si_4C_{40}H_{82}N_6$ Calculated: C (62.53), H (10.50), N (10.67)

Experimental: C (63.12), H (10.93), N (11.05)

**LIFDI-MS:** Only fragmented compound **2a** was found during LIFDI-MS measurements. This is due to **2a** fragmenting at temperatures used for LIFDI-MS.

### Synthesis of silene 2a with labelled $^{13}$CO

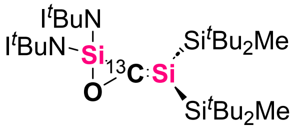

**2a**

$Si_4C_{41}H_{82}O$

788.49 g/mol

For additional analytical proof, the $^{13}$C-labeled isotopomer of silene **2a** was synthesized. For this, a sample of disilene **1a** (30.0 mg, 39.5 µmol) was dissolved in $C_6D_6$ in a J-Young NMR tube. The solution was degassed and gaseous $^{13}$CO (1.0 bar) was added. The spectroscopic data is given with difference to the $^{12}$C analogue.

**$^{13}$C{$^1$H} NMR (126 MHz, $C_6D_6$, 300 K):** δ [ppm] = 231.4 (dt, $J_1$ = 28.6 Hz, $J_2$ = 47.7 Hz, <u>*C*</u>O), 184.5 (s, $^{13}$CO$_{free}$).

**$^{29}$Si{$^1$H} NMR (99 MHz, $C_6D_6$, 300 K):** δ [ppm] = 16.8 (d, $J$ = 8.9 Hz, N$_2$<u>*Si*</u>), -116.6 (d, $J$ = 95.4 Hz, <u>*Si*</u>=C).

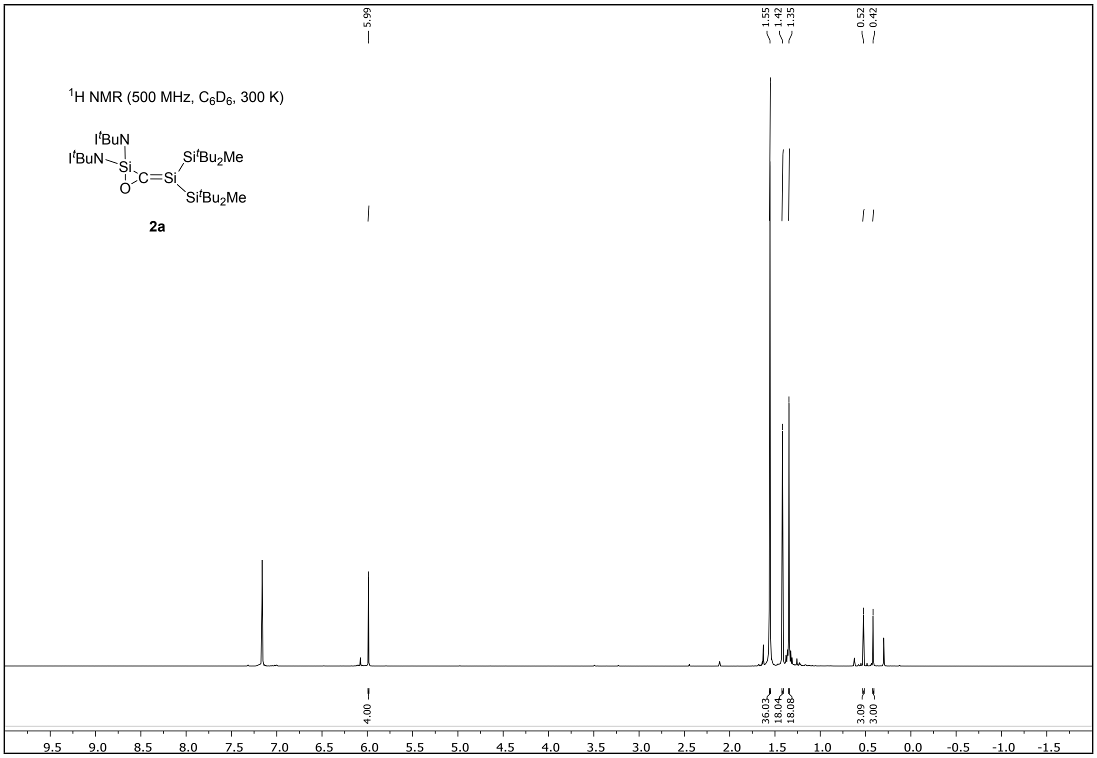


**Figure S1** $^1$H NMR spectrum (500 MHz) of compound **2a** in $C_6D_6$ at 300 K.

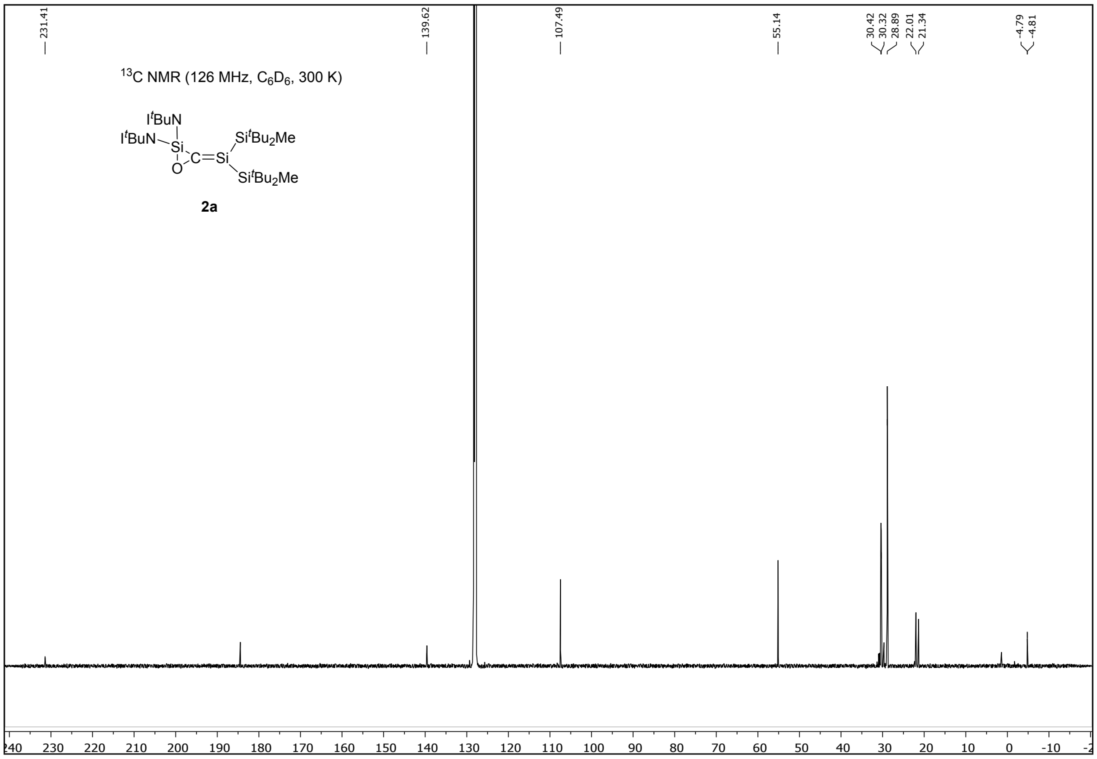


**Figure S2** $^{13}$C NMR spectrum (126 MHz) of compound **2a** in $C_6D_6$ at 300 K.

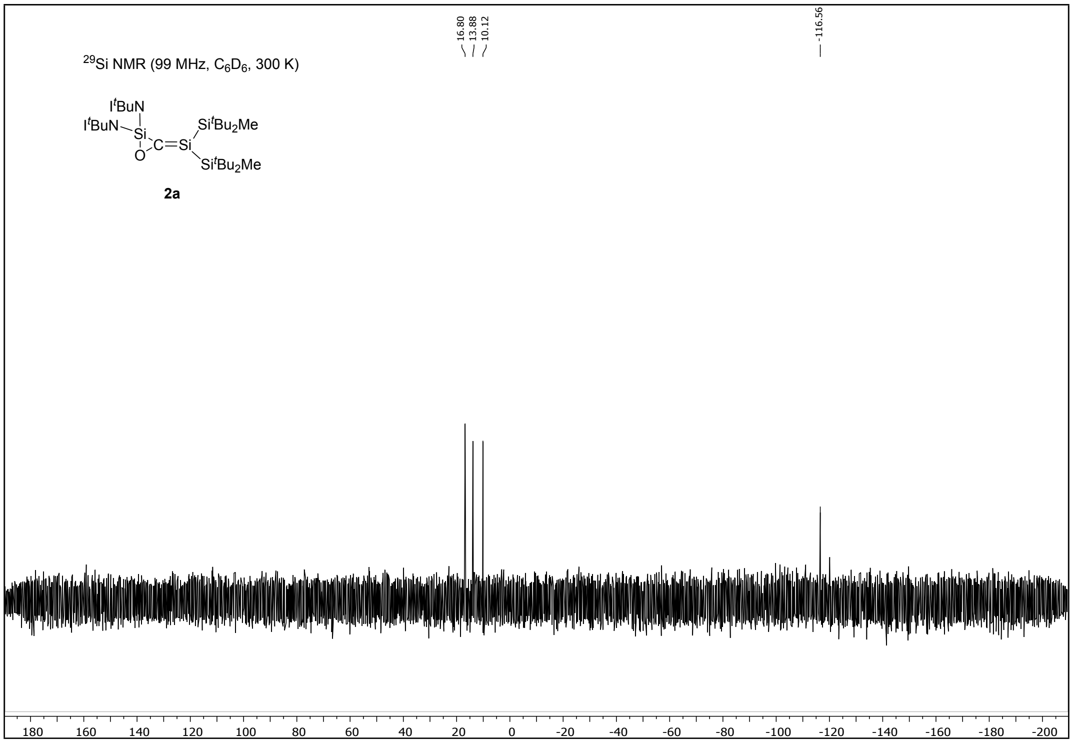


**Figure S3** $^{29}$Si NMR spectrum (99 MHz) of compound **2a** in $C_6D_6$ at 300 K.

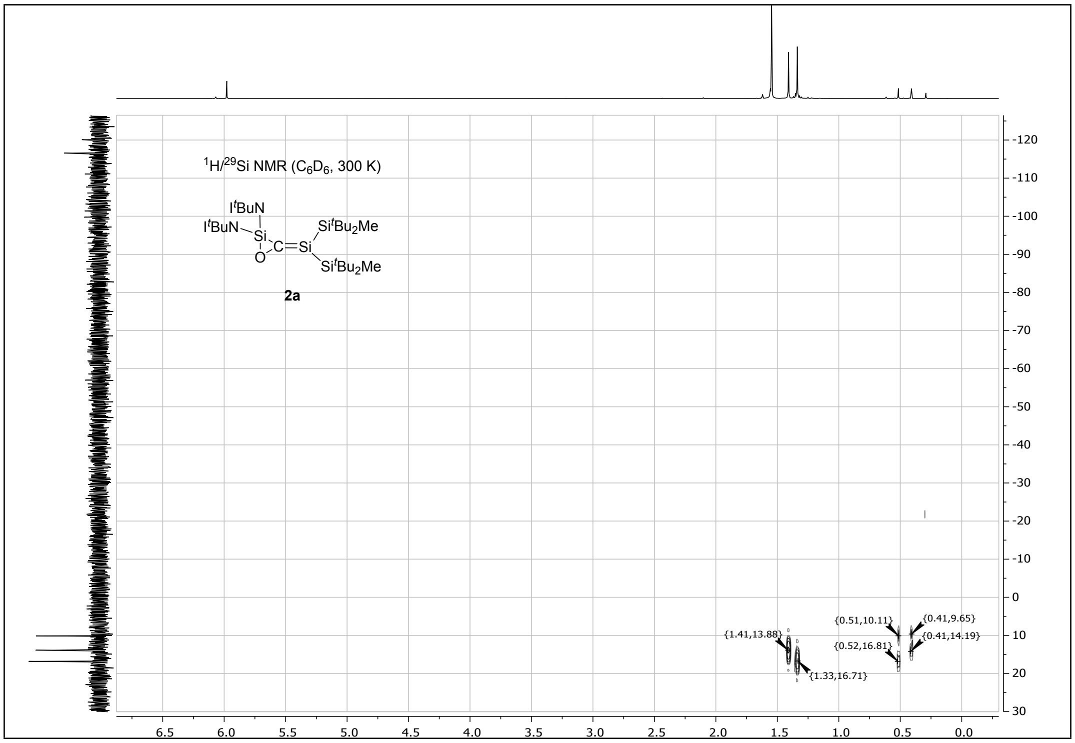


**Figure S4** $^{1}$H/$^{29}$Si HMBC NMR spectrum of **2a** in $C_6D_6$ at 300 K.

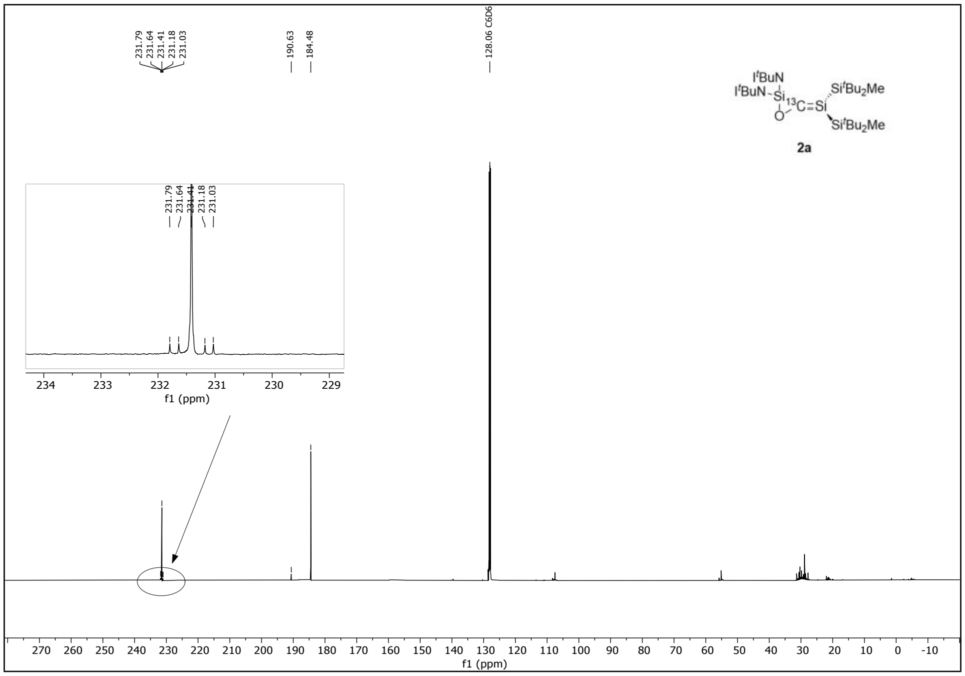

**Figure S5** $^{13}C$ NMR spectrum (126 MHz) of compound **2a** performed with labelled $^{13}CO$ in $C_6D_6$ at 300 K. Coupling constants are determined as $J_1$ = 28.6 Hz and $J_2$ = 47.7 Hz, free CO is visible at 184.5 ppm.

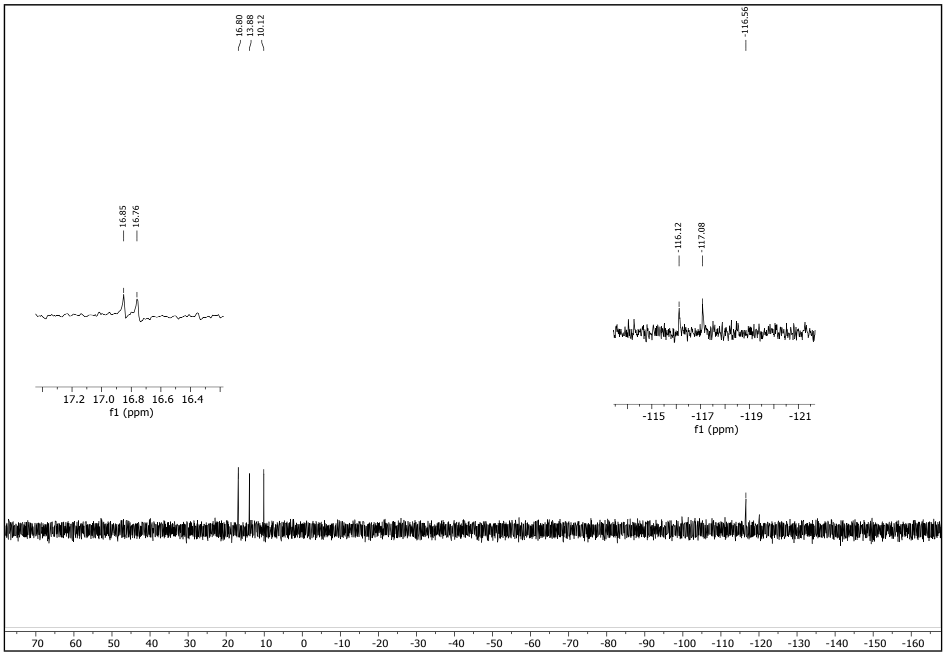

**Figure S6** $^{29}Si$ NMR spectrum (99 MHz) of compound **2a** performed with labeled $^{13}CO$ in $C_6D_6$ at 300 K. Coupling constants are determined as δ = 16.8 ppm (d, $J_1$ = 8.9 Hz, $N_2$<u>*Si*</u>) and δ = -116.6 ppm (d, $J_1$ = 95.4 Hz, <u>*Si*</u>=C).

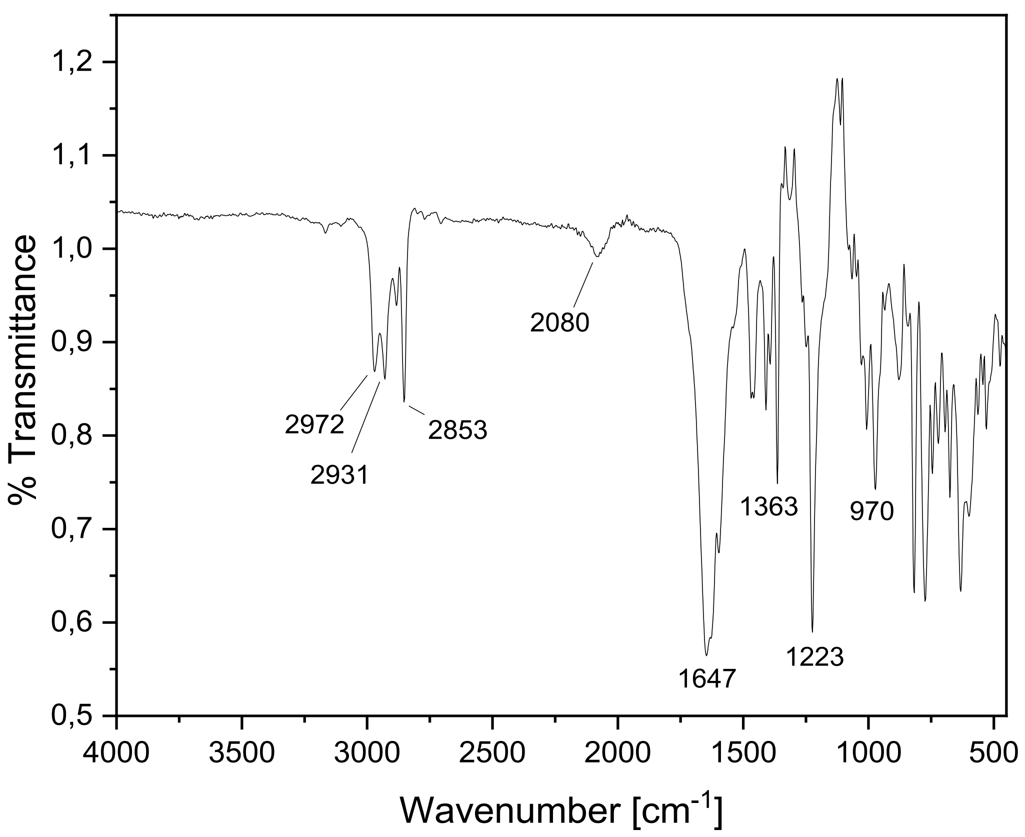


**Figure S7** Solid-state FT-IR spectrum of compound **2a** at 298 K.

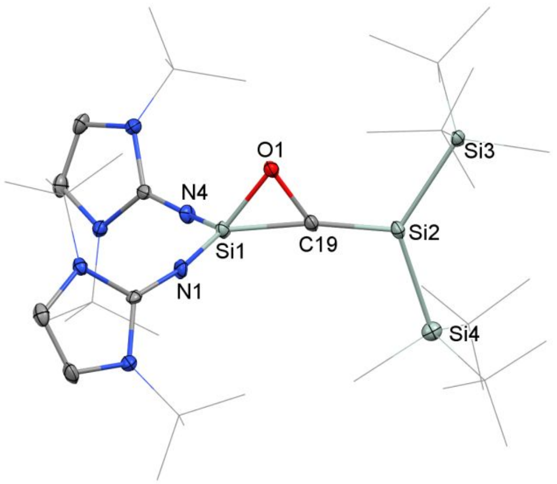


**Figure S8** SC-XRD structure of compound **2a**, with thermal ellipsoids drawn at the 50% probability level. Hydrogen atoms are omitted for clarity, *t*Bu- and Me-groups are simplified as wireframes. Selected bond lengths [Å] and angles [°]: bond legths: Si1-C19 1.799(11), C19-Si2 1.761(13), Si1-O1 1.700(10), O1-C19

1.427(14); bond angles: O1-Si1-C19 48.0(5), O1-C19-Si1 62.3(6), Si1-O1-C19 69.6(6), Si1-C19-Si2 168.9(14); twist angle: 89.96° (N1, N4 / Si3, Si4).

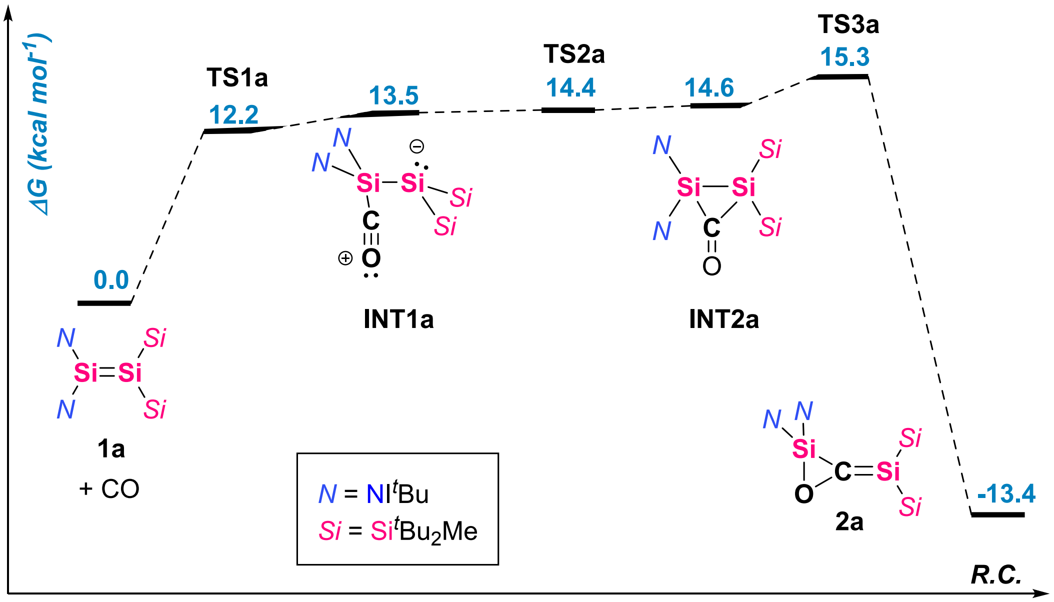


**Figure S9** Free energy diagram calculated at PW6B95-D4(CPCM=Benzene)/def2-QZVPP/r²SCAN-3c level of theory for the proposed reaction mechanism of **1a** with CO to give **2a**.

### 1.3 CO Trapping of 2a

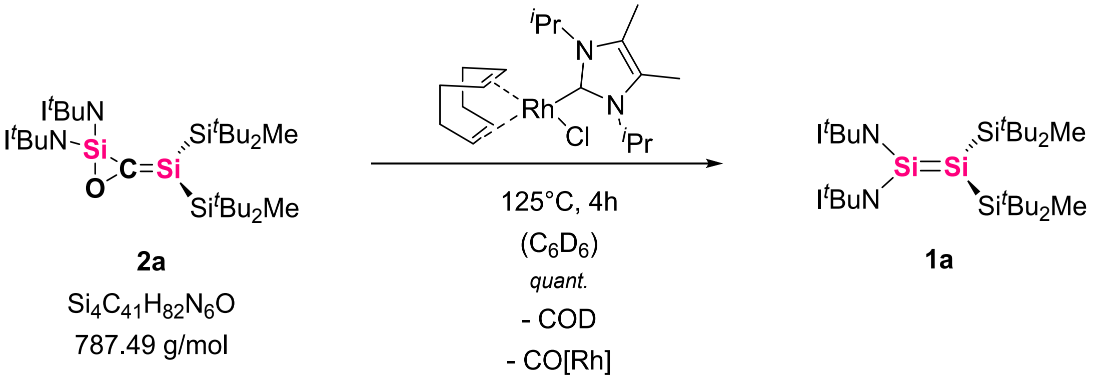


**2a**

$Si_4C_{41}H_{82}N_6O$

787.49 g/mol

**1a**

To a solution of 1,3-disilaallene oxide **2a** (15.0 mg, 19.1 µmol, 1.0 eq.) in $C_6D_6$ (0.4 mL), CODRhCl$^{iPrMe}$NHC (8.15 mg, 19.1 µmol, 1.0 eq.) was added. The reaction mixture was heated to 125 °C and after 4 hours of heating, the bright yellow solution turned dark red, indicating the capture of CO by the Rh CO scavenger.[5] Multinuclear NMR spectroscopy ($^1$H, $^{13}$C, $^{29}$Si) confirmed full conversion. All volatiles were removed in vacuo and a mixture of disilene **1a** and CORhCl$^{iPrMe}$NHC was obtained as dark red solid (20.9 mg, 19.1 µmol, quant.).

**$^1$H NMR (500 MHz, $C_6D_6$, 300 K):** δ [ppm] = 6.00 (s, 4H, C*H*-N), 2.11 ($^{free}$COD), 1.75 ($^{free}$COD) 1.53(s, 36H, *$^t$Bu*N), 1.50 (s, 36H, *$^t$Bu$_2$*MeSi), 0.36 (s, 6H, $^t$Bu$_2$*Me*Si).

**$^{13}$C{$^1$H} NMR (126 MHz, $C_6D_6$, 300 K):** δ [ppm] = 139.2 (*C*=N), 131.2 ($^{free}$COD), 126.3 ($^{free}$COD), 108.3 (*C*H-N), 55.9 (N*C*(CH$_3$)$_3$), 31.4 (Si(C(*C*H$_3$)$_3$)$_2$(CH$_3$)), 29.5 (NC(*C*H$_3$)$_3$), 22.6 (Si(*C*(CH$_3$)$_3$)$_2$(CH$_3$)), -3.0 (Si(C(CH$_3$)$_3$)$_2$(*C*H$_3$)).

**$^{29}$Si{$^1$H} NMR (99 MHz, $C_6D_6$, 300 K):** δ [ppm] = 30.3 (*Si*N$_2$), 23.0 ($^t$Bu$_2$Me*Si*), -176.1 (Si=*Si*Si$_2$).

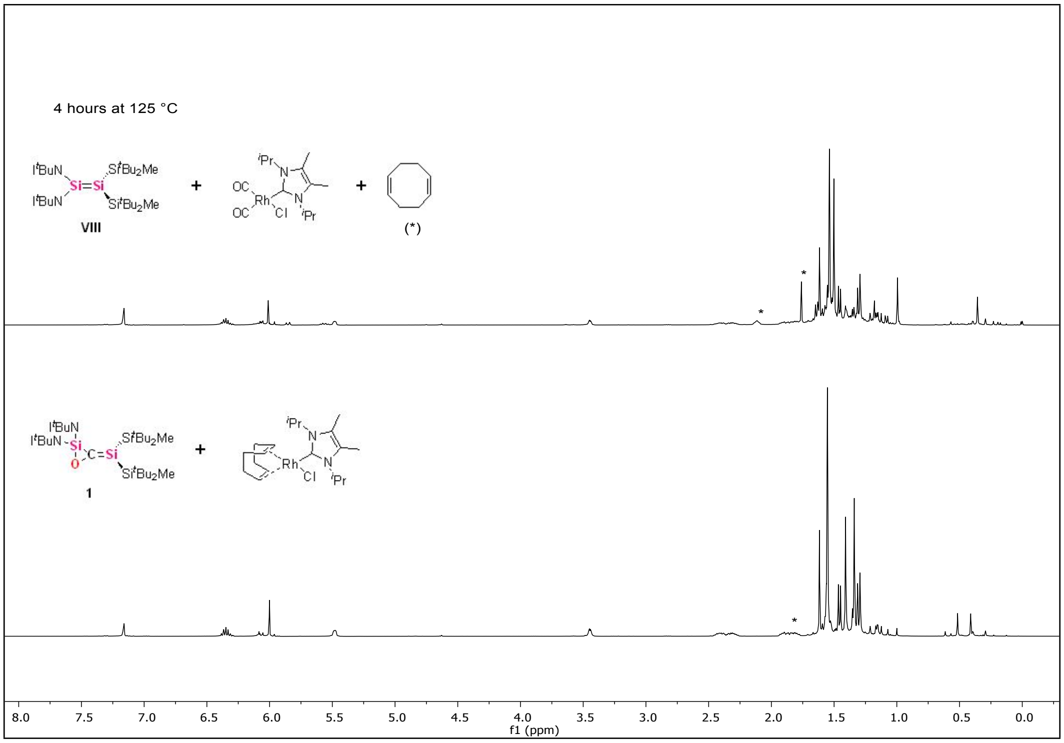


**Figure S10** $^{1}H$ NMR spectra (400 MHz) in $C_6D_6$ at 300 K of compound **2a** with (COD)RhCl$^{iPrMe}$NHC[5] (bottom) and regeneration of disilene **1a** (top) after 4 hours at 125 °C.

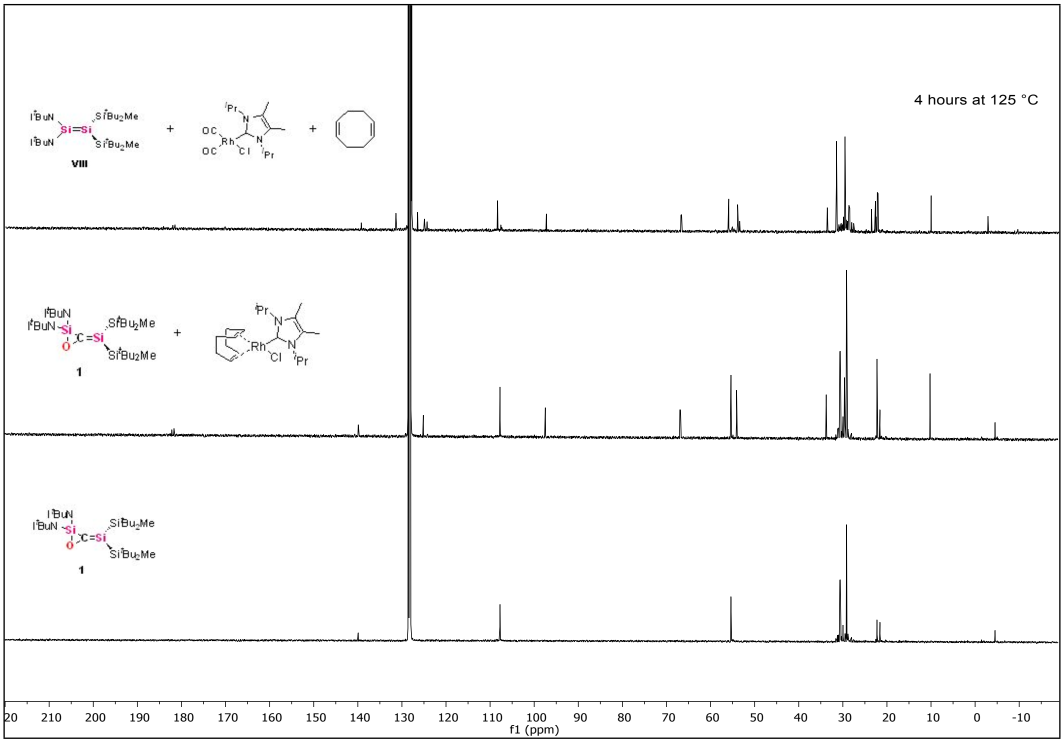


**Figure S11** $^{13}C$ NMR spectra (126 MHz) in $C_6D_6$ at 300 K of compound **2a** (bottom), compound **2a** with (COD)RhCl$^{iPrMe}$NHC[5] (middle), and regeneration of disilene **1a** (top) after 4 hours at 125 °C.

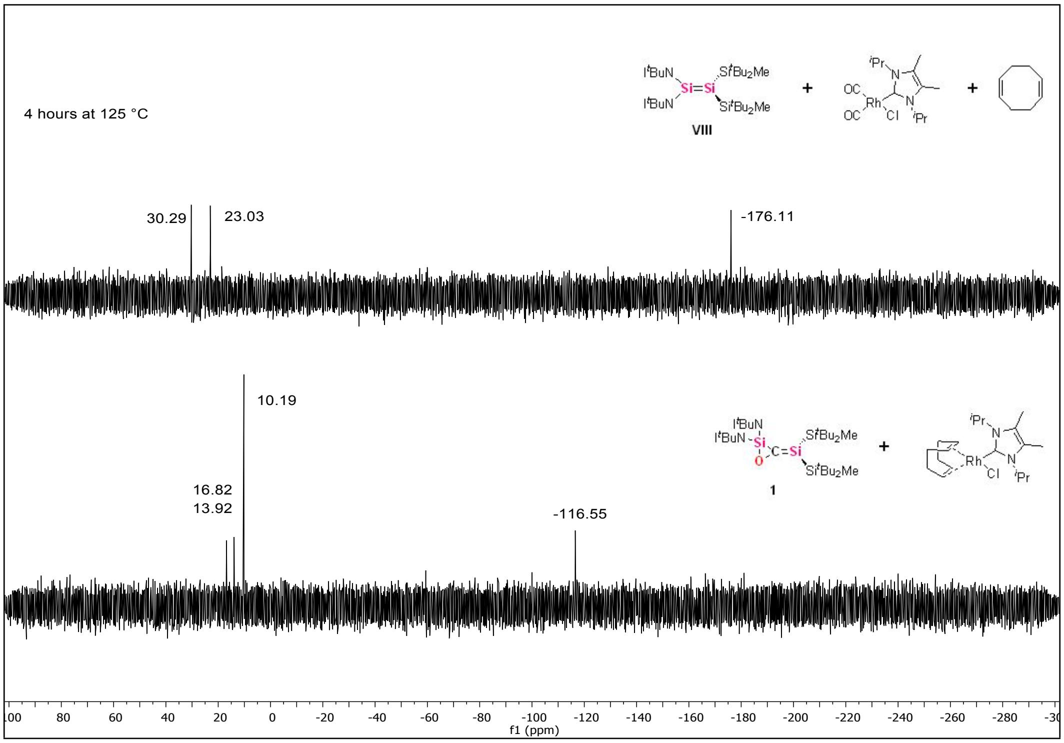


**Figure S12** $^{29}$Si NMR spectra (99 MHz) in $C_6D_6$ at 300 K of compound **2a** with (COD)RhCl$^{iPrMe}$NHC[5] (bottom) and regeneration of disilene **1a** (top) after 4 hours at 125 °C.

## 1.4 1,3-disilaallene oxide 2b

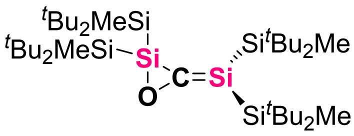


**2b**
$Si_6C_{37}H_{84}O$
713.59 g/mol

A solution of disilene ($^t$Bu$_2$MeSi)$_2$Si=Si(Si$^t$Bu$_2$Me)$_2$ (**1b**) (200 mg, 333 µmol) in toluene (10 mL) in a pressurizable Schlenk flask was degassed and exposed to carbon monoxide (1.3 bar). Upon heating the mixture to 80 °C for 4 days, the deep blue color vanished slowly to give a pale yellow solution. After evaporation of all volatiles under reduced pressure, compound **2b** was obtained as a yellow, crystalline solid (242 mg, 316 µmol, 95% yield). Crystals of **2b** suitable for SC-XRD can be obtained from a cooled (-35 °C) solution in toluene or *n*-hexane.

**$^1$H NMR (500 MHz, $C_6D_6$, 300 K):** δ [ppm] = 1.32 (s, 18H, $^t$<u>*Bu*</u>$_2$MeSi$^{3/4}$), 1.26 (s, 18H, $^t$<u>*Bu*</u>$_2$MeSi$^{3/4}$), 1.16 (s, 18H, $^t$<u>*Bu*</u>$_2$MeSi$^{5/6}$), 1.15 (s, 18H, $^t$<u>*Bu*</u>$_2$MeSi$^{5/6}$), 0.54 (s, 3H, $^t$Bu$_2$<u>*Me*</u>Si$^{3/4}$), 0.40 (s, 3H, $^t$Bu$_2$<u>*Me*</u>Si$^{3/4}$), 0.32 (s, 3H, $^t$Bu$_2$<u>*Me*</u>Si$^{5,6}$).
**$^{13}$C{$^1$H} NMR (126 MHz, $C_6D_6$, 300 K):** δ [ppm] = 197.1 (<u>*C*</u>O), 30.5 (Si(C(<u>*C*</u>H$_3$)$_3$)$_2$(CH$_3$)), 30.4 (Si(C(<u>*C*</u>H$_3$)$_3$)$_2$(CH$_3$)), 30.2 (Si(C(<u>*C*</u>H$_3$)$_3$)$_2$(CH$_3$)), 29.8 (Si(C(<u>*C*</u>H$_3$)$_3$)$_2$(CH$_3$)), 22.6 (Si(<u>*C*</u>(CH$_3$)$_3$)$_2$(CH$_3$)), 22.4 (Si(<u>*C*</u>(CH$_3$)$_3$)$_2$(CH$_3$)), 21.7 (Si(<u>*C*</u>(CH$_3$)$_3$)$_2$(CH$_3$)), 21.4 (Si(<u>*C*</u>(CH$_3$)$_3$)$_2$(CH$_3$)), –4.5 (Si(C(CH$_3$)$_3$)$_2$(<u>*C*</u>H$_3$)), –4.6 (Si(C(CH$_3$)$_3$)$_2$(<u>*C*</u>H$_3$)), –5.7 (Si(C(CH$_3$)$_3$)$_2$(<u>*C*</u>H$_3$)).
**$^{29}$Si{$^1$H} NMR (99 MHz, $C_6D_6$, 300 K):** δ [ppm] = 19.1 (<u>*Si*</u> $^{3/4}$), 14.6 (<u>*Si*</u> $^{3/4}$), 14.1 (<u>*Si*</u> $^2$), 9.2 (<u>*Si*</u> $^{5,6}$), -50.0 (<u>*Si*</u> $^1$).
**FT-IR [cm$^{-1}$]:** 2929 (w), 2855 (w), 2080 (Si=C), 1466 (s), 1363 (s), 1248 (s), 1180 (w), 1452 (s), 1544 (s), 768 (s).

**m.p.**: 221°C

**EA:** $Si_6C_{37}H_{84}O$ Calculated: C (62.28), H (11.87), N (-)
Experimental: C (61.10), H (11.94), N (-)

Note: The carbon value was reproducibly low, presumably due to the formation of incombustible silicon carbides.

**LIFDI-MS:** $Si_6C_{37}H_{84}O$ calculated (found): 712.5138 (712.5102 m/z)

**Synthesis of 2b with labeled $^{13}CO$**

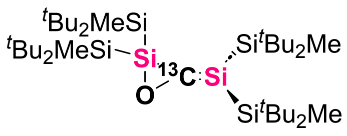

**2b**

$Si_6C_{37}H_{84}O$

714.59 g/mol

For additional analytical proof, the $^{13}C$-labeled isotopomer of silene 2b was synthesized. For this, a sample of disilene 1b (30.0 mg, 43.8 µmol) was dissolved in $C_6D_6$ in a J-Young NMR tube. The solution was degassed and gaseous $^{13}CO$ (0.8 bar) was added. The spectroscopic data is given with difference to the $^{12}C$ analogue.

**$^{13}C\{^1H\}$ NMR (126 MHz, $C_6D_6$, 300 K):** δ [ppm] = 197.1 (t, $J_1$ = 17.3 Hz, $J_2$ = 32.1 Hz, C̲O).

**$^{29}Si\{^1H\}$ NMR (99 MHz, $C_6D_6$, 300 K):** δ [ppm] = 19.1 (d, $J$ = 8.1 Hz, *S̲i̲*$^t$Bu$_2$Me), 14.6 (d, $J$ = 14.0 Hz, *S̲i̲*$^t$Bu$_2$Me), -49.9 (d, $J$ = 33.8 Hz, *S̲i̲*=C).

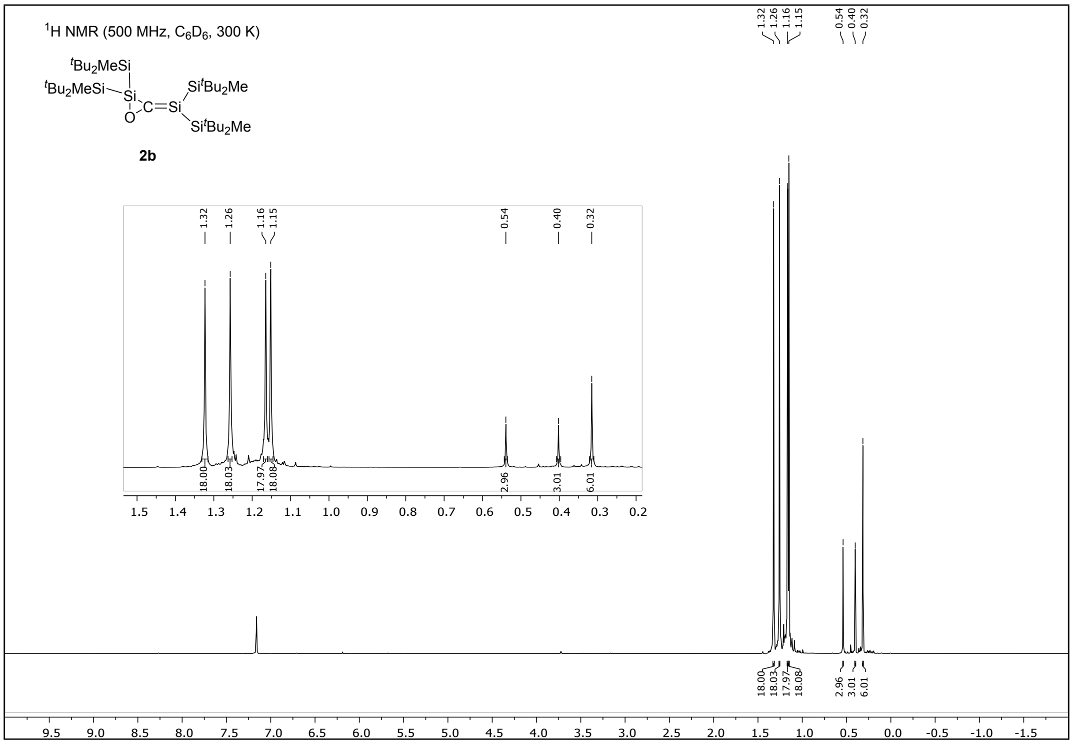


**Figure S13** $^1H$ NMR spectrum (500 MHz) of compound **2b** in $C_6D_6$ at 300 K.

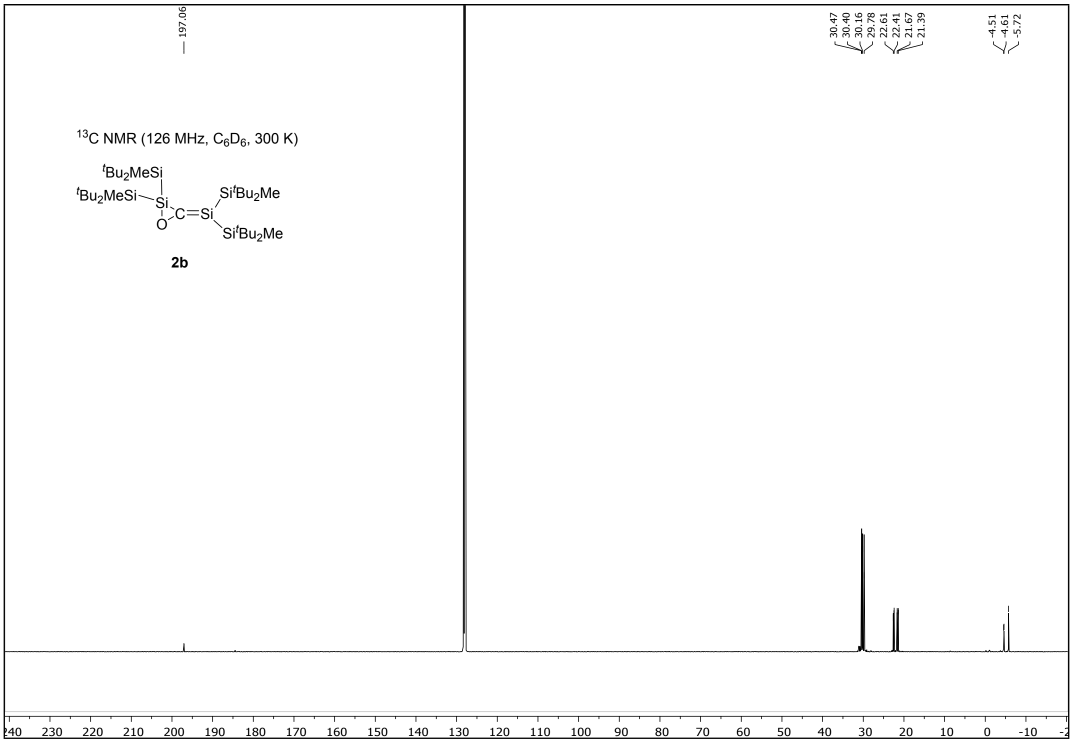


**Figure S14** $^{13}$C NMR spectrum (126 MHz) of compound **2b** in $C_6D_6$ at 300 K.

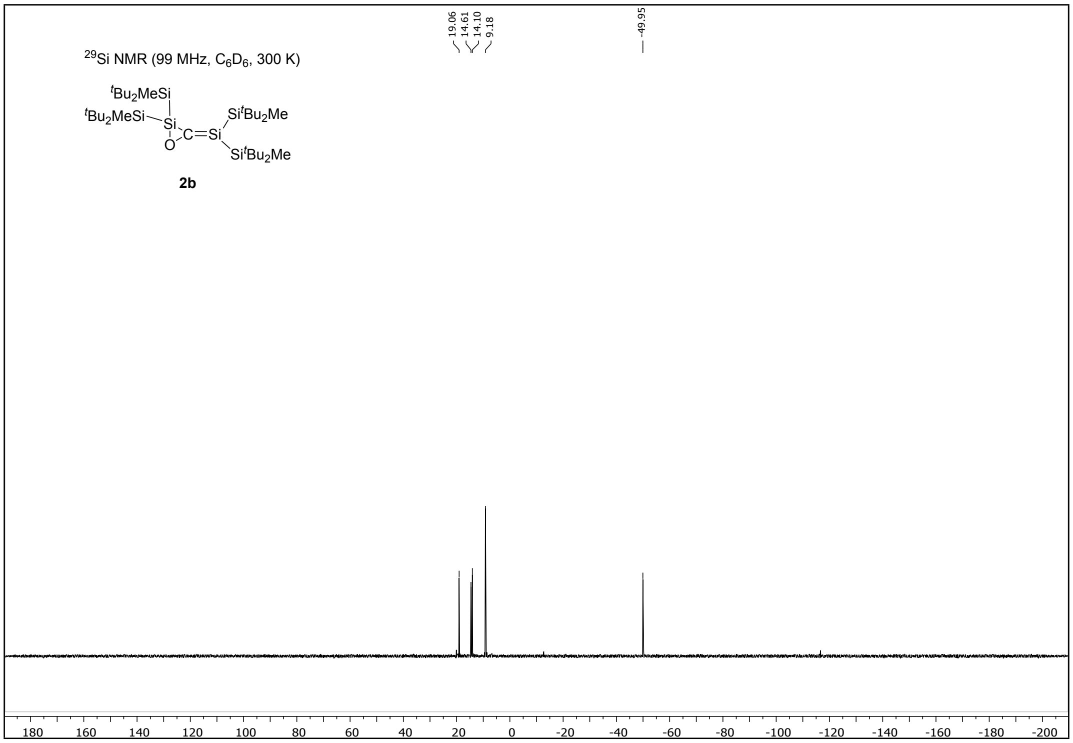


**Figure S15** $^{29}$Si NMR spectrum (99 MHz) of compound **2b** in $C_6D_6$ at 300 K.

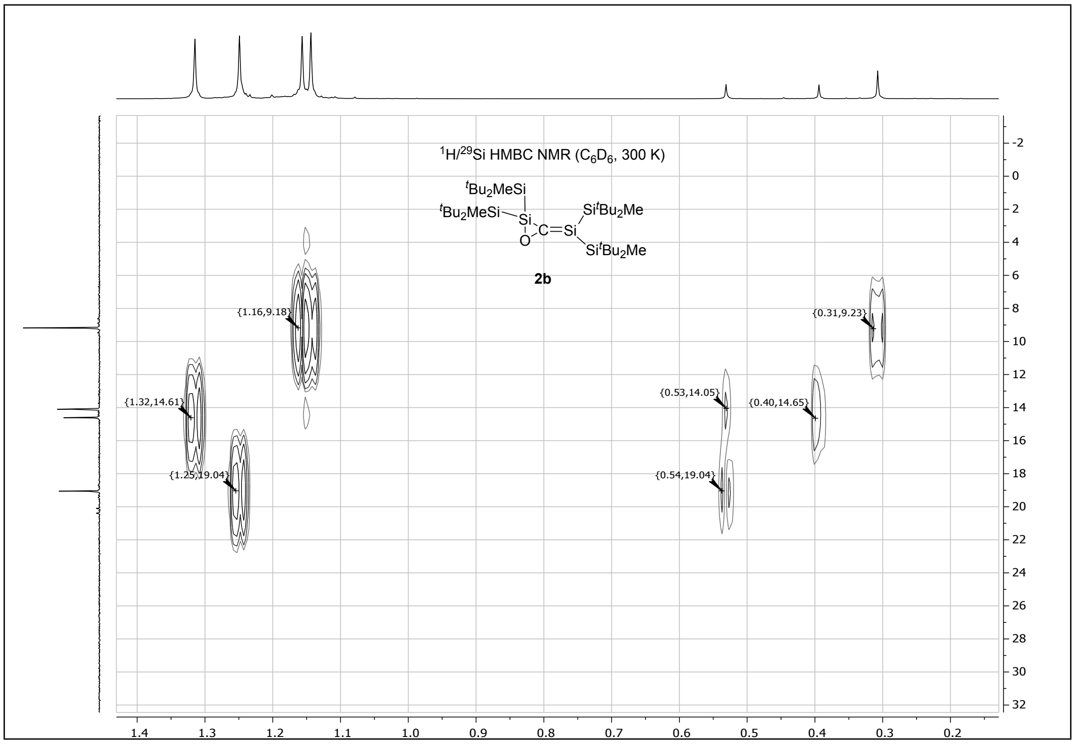


**Figure S16** $^1$H/$^{29}$Si HMBC NMR spectrum of compound **2b** in $C_6D_6$ at 300 K.

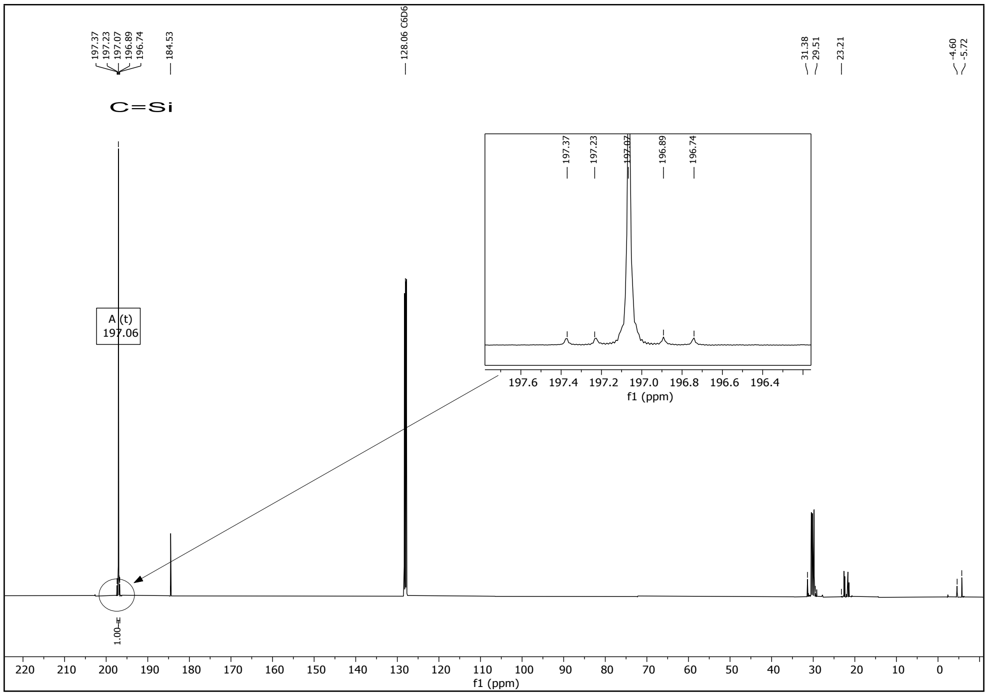


**Figure S17** $^{13}C$ NMR spectrum (100 MHz) of compound **2b** performed with labelled $^{13}CO$ in $C_6D_6$ at 300 K. Free CO is visible at 184.5 ppm and the $^{13}C$=Si signal is visible at 197.0 ppm. Corresponding coupling constants are identified as $J_1$ = 17.2 Hz and $J_2$ = 32.1 Hz.

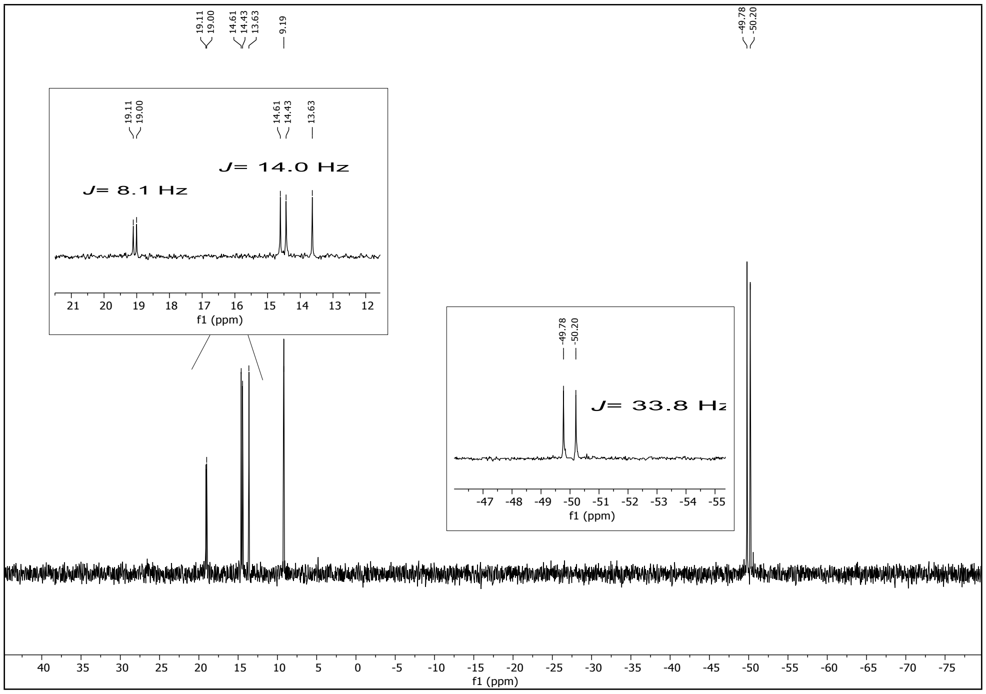


**Figure S18**$^{29}$Si NMR spectrum (79.5 MHz) of compound **2b** performed with labelled $^{13}$CO in $C_6D_6$ at 300 K.

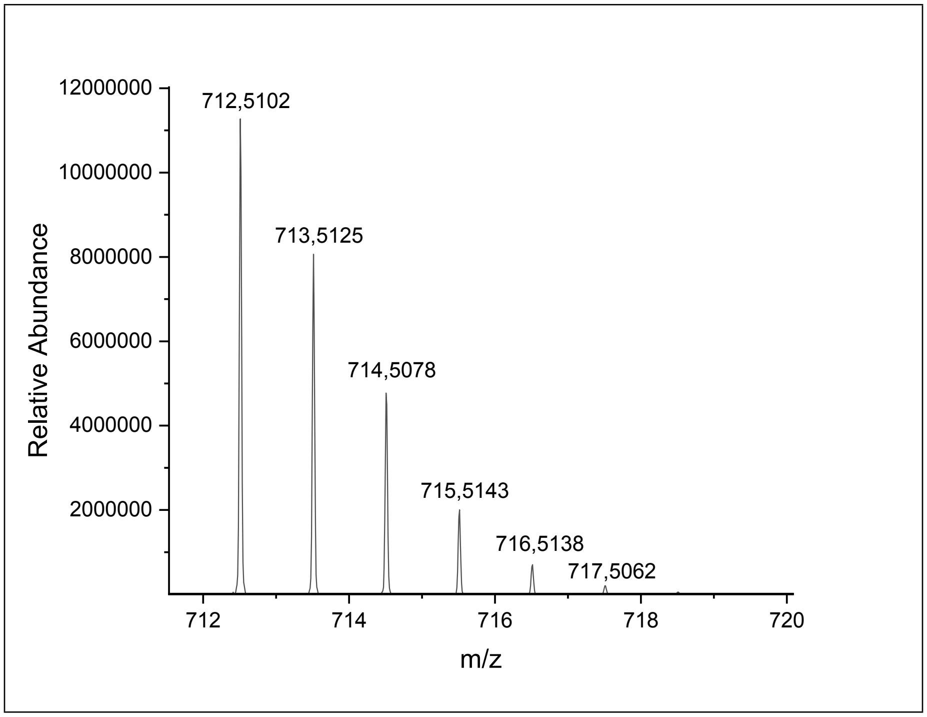


**Figure S19** Measured LIFDI-MS spectrum of **2b** in toluene.

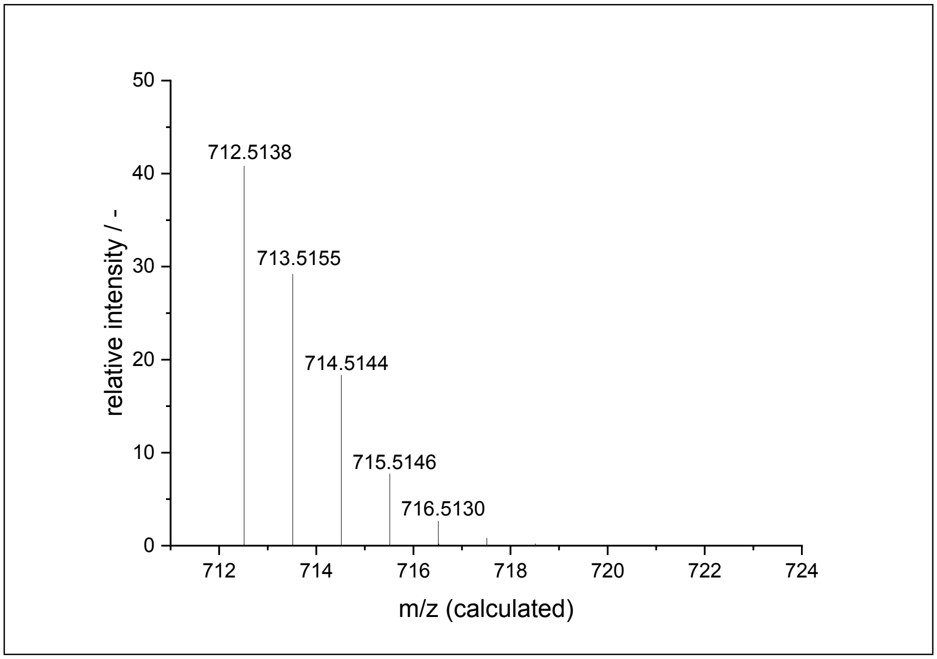


**Figure S20** Calculated mass spectrum of **2b**.[6]

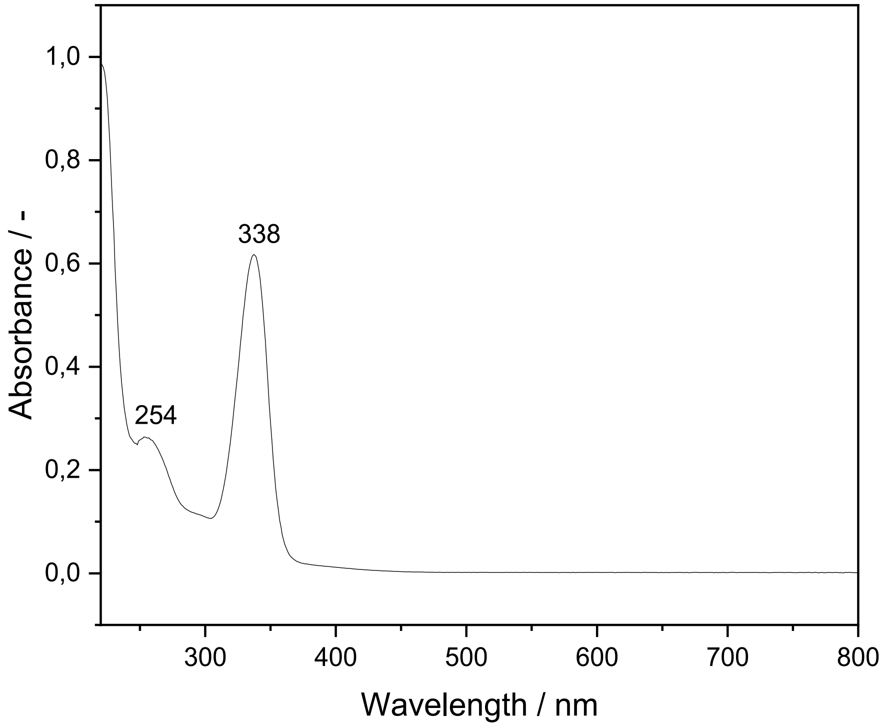


**Figure S21** UV-Vis spectrum of compound **2b** in *n*-hexane (1.44×10-4 M), measured at room temperature (λmax = 254 nm, 338 nm).

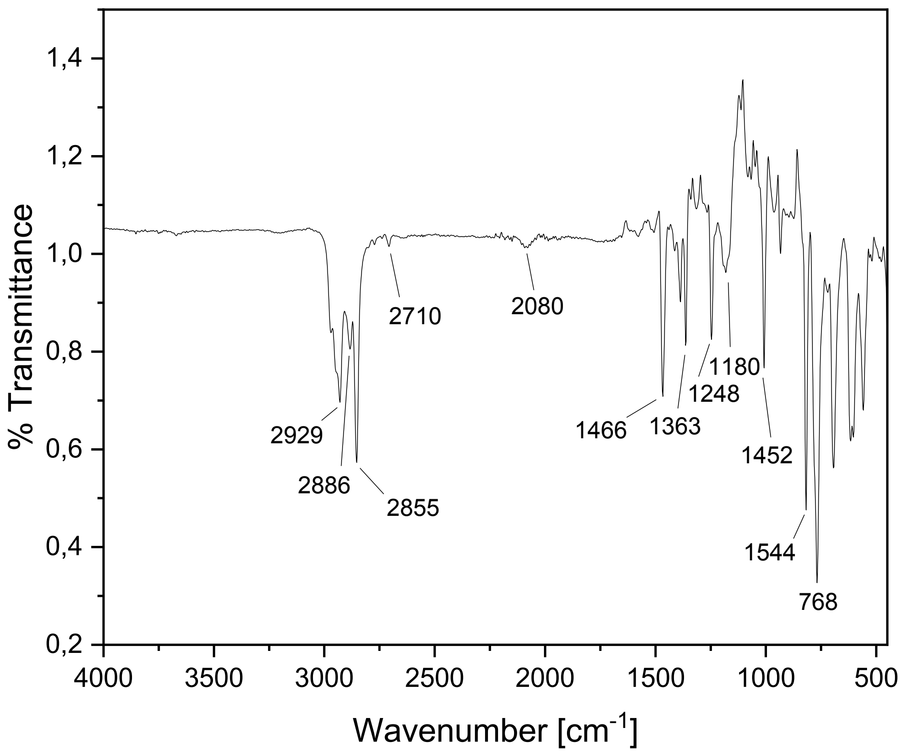


**Figure S22** Solid-state FT-IR spectrum of compound **2b** at 298 K.

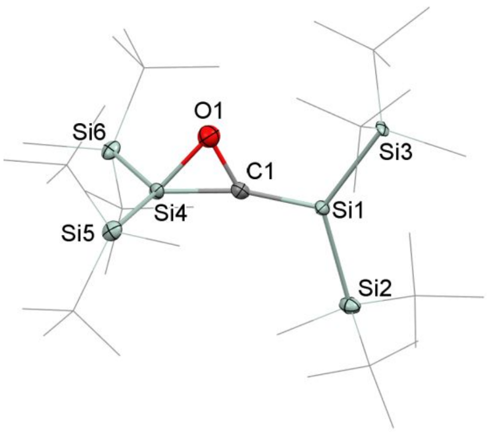


**Figure S23** SC-XRD structure of silene **2b**, with thermal ellipsoids drawn at the 50% probability level. Hydrogen atoms are omitted for clarity, *t*Bu- and Me-groups are simplified as wireframes. Selected bond lengths [Å] and angles [°]: bond lengths: Si4-C1 1.78(1), C1-Si1 1.78(1), Si4-O1 1.73(1)), O1-C1 1.40(1); bond angles: O1-Si4-C1 47.0(5), O1-C1-Si4 64.4(6), Si4-O1-C1 68.5(6), Si4-C1-Si1 167.4(7); ligand twisting: 85.94° (Si5, Si6 / Si2, Si3).

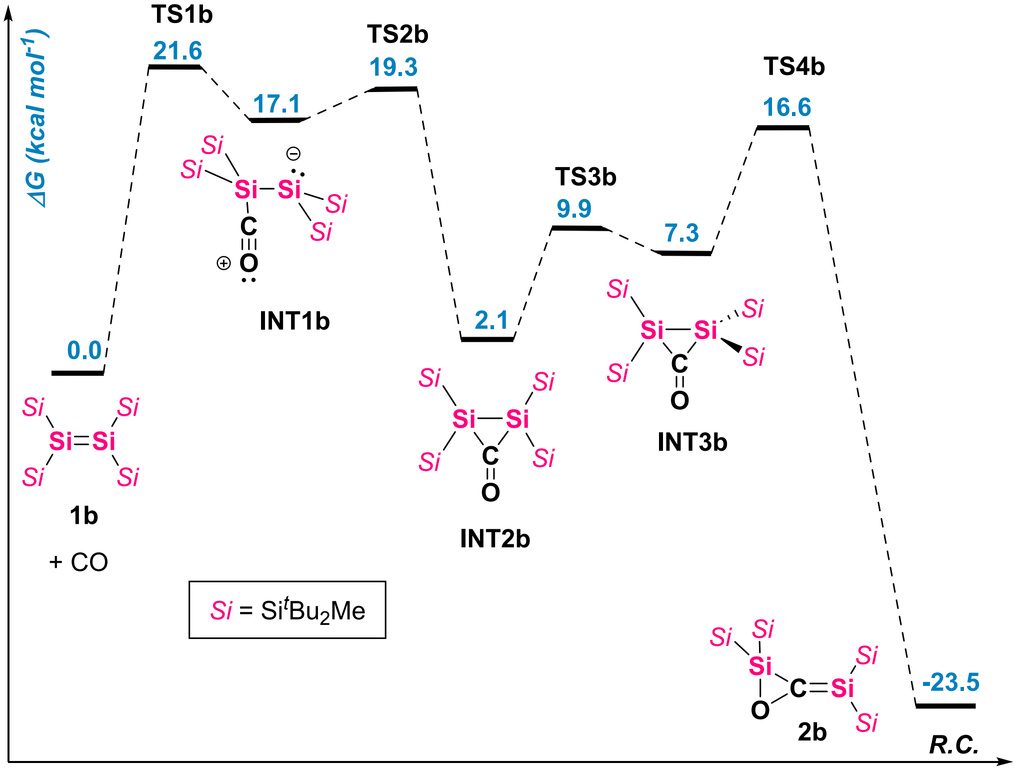


**Figure S24** Free energy diagram calculated at PW6B95-D4(CPCM=Benzene)/def2-QZVPP/r$^2$SCAN-3c level of theory for the proposed reaction mechanism of **1b** with CO to give **2b**.

**Explanation for the difference in energy profiles for the formation of 2a and 2b:**

Following a question from the reviewers regarding the difference in energy profiles when forming **2a** and **2b**, we suggest that the difference originates from the strength of the interaction between the silylene and the silicon carbonyl moieties in **2a** and **2b**. This point is mentioned in the main text using the interaction energies. The stronger interaction between the bis(silyl)silylene moiety with silicon carbonyl moiety is also reflected in the WBI and MBO (this is shown in figure 2 in the main text). Why is this interaction stronger in the case of 2b? For this, a sobEDA (second-order block-localized energy decomposition analysis) was carried out. We carried out the calculation using Gaussian and the sobEDA software at the PBE0-D3BJ/def2-TZVP level of theory using the r$^2$SCAN-3c optimized geometries. Here are the results for the interaction between the silylene and the silicon carbonyl complex in **2a** and **2b**.

| Component | **2b** | **2a** | ΔE |
|---|---|---|---|
| Total interaction energy | –122.51 | –116.38 | 6.13 |
| Electrostatic (E_els) | –287.99 | –348.40 | –60.41 |
| Exchange (E_x) | –225.05 | –262.50 | –37.45 |
| Pauli repulsion (E_rep) | 845.79 | 1031.3 | 185.55 |
| Exchange-repulsion (E_xrep = E_x + E_rep) | 620.74 | 768.83 | 148.09 |
| Orbital (E_orb) | –400.53 | –489.09 | –88.56 |
| DFT correlation (E_DFTc) | –39.90 | –36.75 | 3.15 |
| Dispersion correction (E_dc) | –14.83 | –10.97 | 3.86 |
| Coulomb correlation (E_c = E_DFTc + E_dc) | –54.73 | –47.72 | 7.01 |

Electrostatic (E_els) measures charge-based attraction or repulsion. Exchange (E_x) reflects electron cloud overlap. Pauli Repulsion (E_rep) indicates electron repulsion due to close contact. Exchange-Repulsion (E_xrep) combines exchange and Pauli terms. Orbital (E_orb) shows stabilization from orbital interactions. DFT Correlation (E_DFTc) and Dispersion correction (E_dc) account for electron correlation and van der Waals forces, respectively. Coulomb Correlation (E_c) is total correlation energy, combining DFT and dispersion effects.

The total interaction energy at this level of theory is higher in **2b** consistent with the dissociation energy results mentioned in the main text. Indicating stronger binding between bis(silyl)silylene moiety with the silicon carbonyl moiety, in comparison to bis(NHI)silylene. **2a** shows stronger electrostatic attraction (ΔE = -60.4 kcal mol$^{-1}$) and orbital interactions (ΔE = -88.6 kcal mol$^{-1}$), suggesting a more polar and covalent character. However, **2a** has higher Pauli repulsion (ΔE = 185.6 kcal mol$^{-1}$), implying closer higher steric hindrance, which reduces stability. **2b** benefits from slightly stronger dispersion forces (ΔE = 3.4). These results suggest that while **2a** benefits from electrostatic and orbital interactions **2b** benefits lower exchange-repulsion. The energy differences between these components are very large, and it all comes down to the interaction energy difference of just over 6 kcal mol$^{-1}$. The large component differences balance each other, leading to the small total energy difference. **2a** has a more polar and covalent character (stronger E_els and E_orb), while 2b (A-B) is more dispersion-driven (stronger E_dc) with less steric repulsion (lower E_rep). The small total energy difference from the complex interplay of these components makes it hard to pinpoint one dominant factor. It can be said that the nature of these interactions is fundamentally different in the two cases, and this is why it is difficult to point to what exactly causes the relatively small difference in the stability of **2a** and **2b**. This captures the essence of EDA, where individual terms can be large but cancel each other.

### 1.5 Hexacoordinate disilyl ketone intermediate: disila-2,4-furandione 3

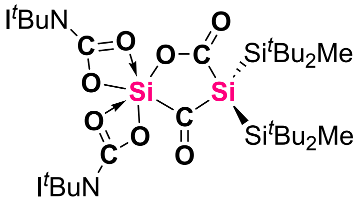

**3**

$Si_4C_{44}H_{82}N_6O_7$

M = 919.52 g/mol

In a pressurize-able J-Young NMR tube, **2a** $(I^tBuN)_2SiOC{=}Si(Si^tBu_2Me)_2$ (20.0 mg, 25.4 µmol) was dissolved in $C_6D_6$ (0.4 mL). The solution was degassed and exposed to carbon dioxide (0.5 bar). After 10 minutes, a change of color from pale-yellow to bright pink was observed. When leaving the benzene-solution in the NMR tube for 2 days, **3** precipitates as pink crystals suitable for SC-XRD. Removal of the now clear benzene-solution and drying in vacuo, compound **3** was obtained as pink powder (16.1 mg, 17.4 µmol, 68.5% yield). After precipitation, **3** is only soluble in pyridine, and not soluble in benzene, toluene, hexane, THF or acetonitrile.

**$^1$H NMR (500 MHz, Pyr-d$_5$, 300 K):** δ [ppm] = 7.37 (s, 4H, NHI), 1.65 (s, 36H, *N-$^t$Bu*), 1.33 (s, 18H, *$^t$Bu$_2$*MeSi), 1.25 (s, 18H, *$^t$Bu$_2$*MeSi), 0.52 (s, 6H, $^t$Bu$_2$*Me*Si).
**$^{13}$C{$^1$H} NMR (126 MHz, Pyr-d$_5$, 300 K):** δ [ppm] = 315.10 ($Si_2\underline{C}O$), 193.2 ($Si_2\underline{C}O_2$), 165.0 (2C, $Si(\underline{C}O_2)_2$), 155.7 (C=N), 114.61 (CH-N), 60.4 ($N\underline{C}(CH_3)_3$, 55.8 ($N\underline{C}(CH_3)_3$), 34.7 ($Si\underline{C}(CH_3)_3$), 30.6 ($NC(\underline{C}H_3)_3$), 29.7 ($NC(\underline{C}H_3)_3$), 29.2 ($NC(\underline{C}H_3)_3$), 30.7 ($NC(\underline{C}H_3)_3$), 23.2 ($SiC(\underline{C}H_3)_3$), 21.9 ($SiC(\underline{C}H_3)_3$), 2.7 ($Si\underline{C}H_3$).
**$^{29}$Si{$^1$H} NMR (99 MHz, Pyr-d$_5$, 300 K):** δ [ppm] = 13.9 ($\underline{Si}{}^tBu_2Me$), -85.7 ($\underline{Si}Si_2$), -171.5 ($\underline{Si}O_5$).

**m.p.:** 120°C

**LIFDI-MS**: $Si_4C_{44}H_{82}N_6O_7$ calculated (found experimentally): 920.5317 (921.5467 m/z).

**FT-IR [cm$^{-1}$]:** 3174 (w), 2970 (s), 2929 (s), 2883 (s), 2853 (s), 2078 (w), 1647 (s), 1619 (s), 1592(s), 1365 (s).

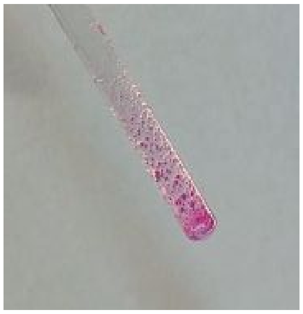

*Picture of **3**’s pink color*

### Synthesis of 3 using labeled $^{13}CO_2$ gas

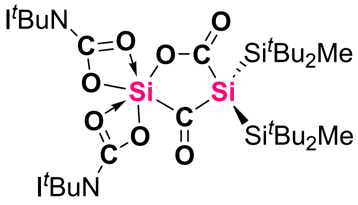

**3**

$Si_4C_{44}H_{82}N_6O_7$

M = 919.52 g/mol

For additional analytical proof, intermediate **3** was synthesized using labeled $^{13}CO_2$ gas. For this, a sample of **2a** (20.0 mg, 25.4 µmol) was dissolved in $C_6D_6$ in a J-Young NMR tube. The solution was degassed and gaseous $^{13}CO_2$ (0.5 bar) was added. The spectroscopic data is given with difference to the $^{12}C$ analogue.

**$^{13}C\{^1H\}$ NMR (126 MHz, Pyr-d$_5$, 300 K):** δ [ppm] = 192.5 (t, *J* = 28.1 Hz, $Si_2\underline{C}O_2$), 164.4 (2C, $Si(\underline{C}O_2)_2$).

**$^{29}Si\{^1H\}$ NMR (99 MHz, Pyr-d$_5$, 300 K):** δ [ppm] = -86.2 (d, *J* = 55.4 Hz, $\underline{Si}Si_2$), -171.5 ($\underline{Si}O_5$).

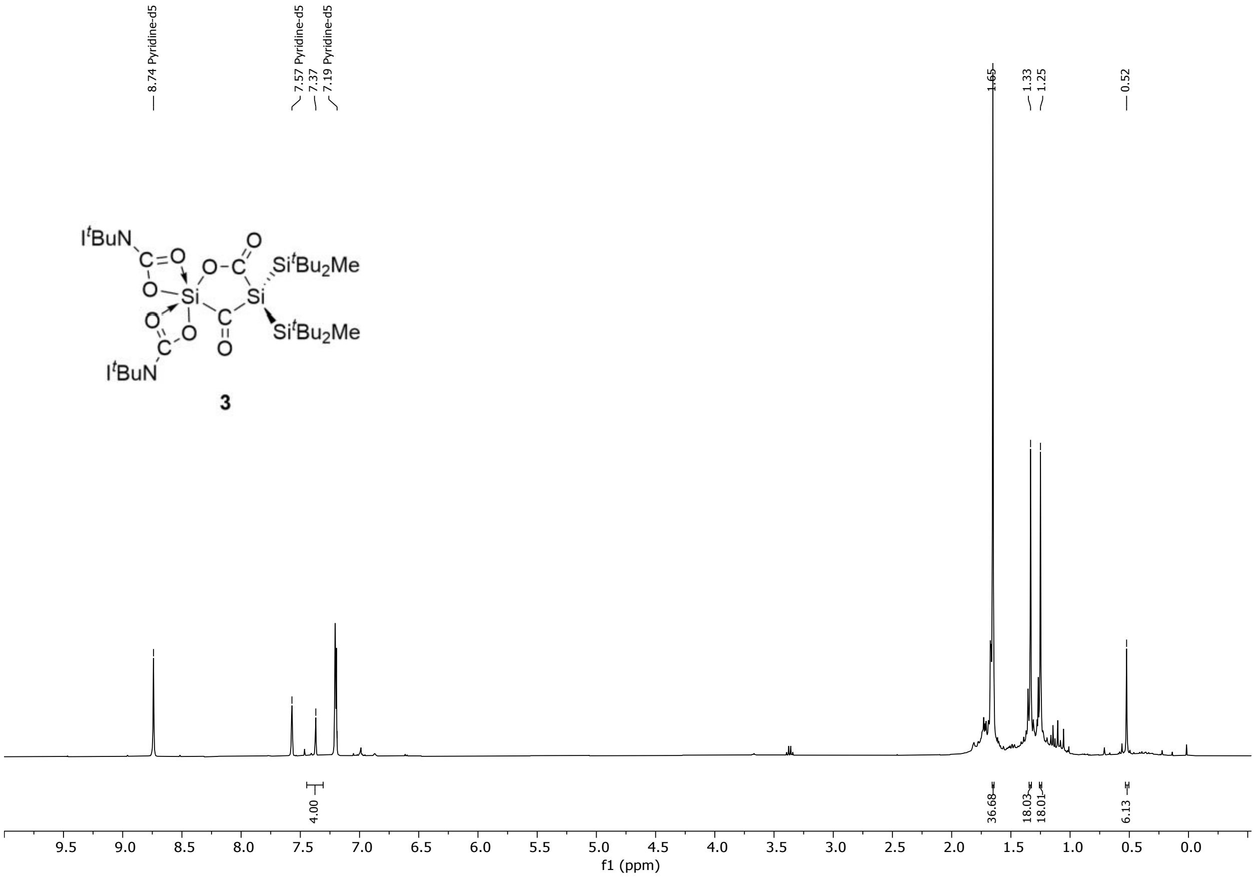


**Figure S25** $^1H$ NMR spectrum of **3** in pyridine-*d$_5$* at 300 K. Minor impurity $(^tBu_2MeSi)_2SiH_2$ at 3.36 ppm and 1.08 ppm.

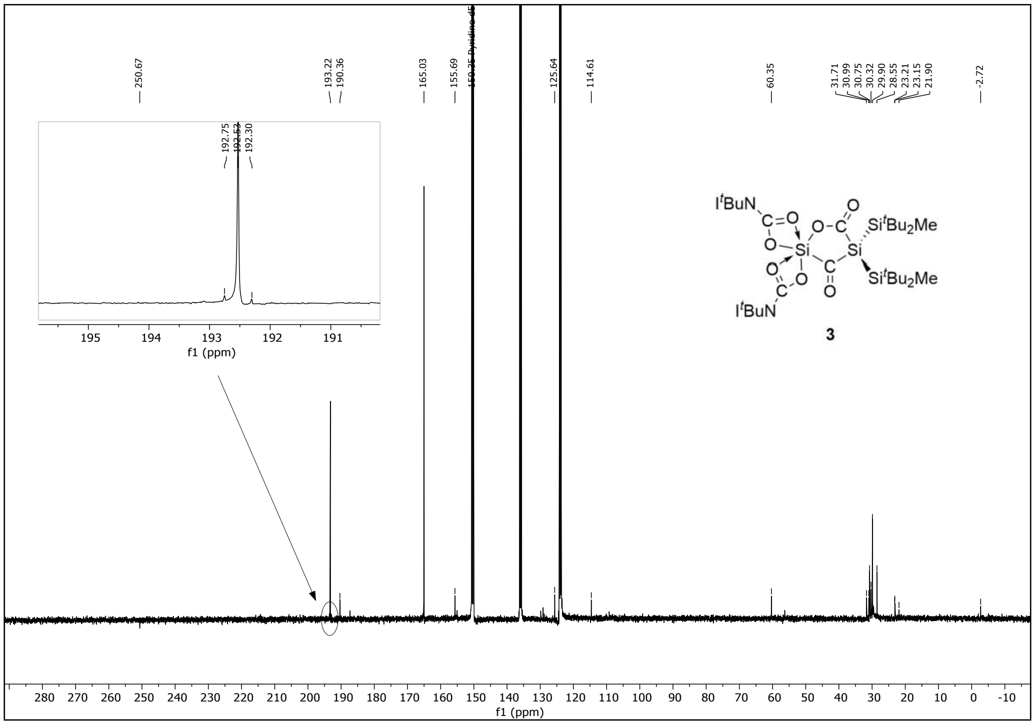

**Figure S26** $^{13}C$ NMR spectrum of **3** in pyridine-$d_5$ at 300 K with $^{13}CO_2$. Coupling constant is determined as δ = 192.5 ppm (t, $J$ = 28.1 Hz, Si<u>C</u>$O_2$Si).

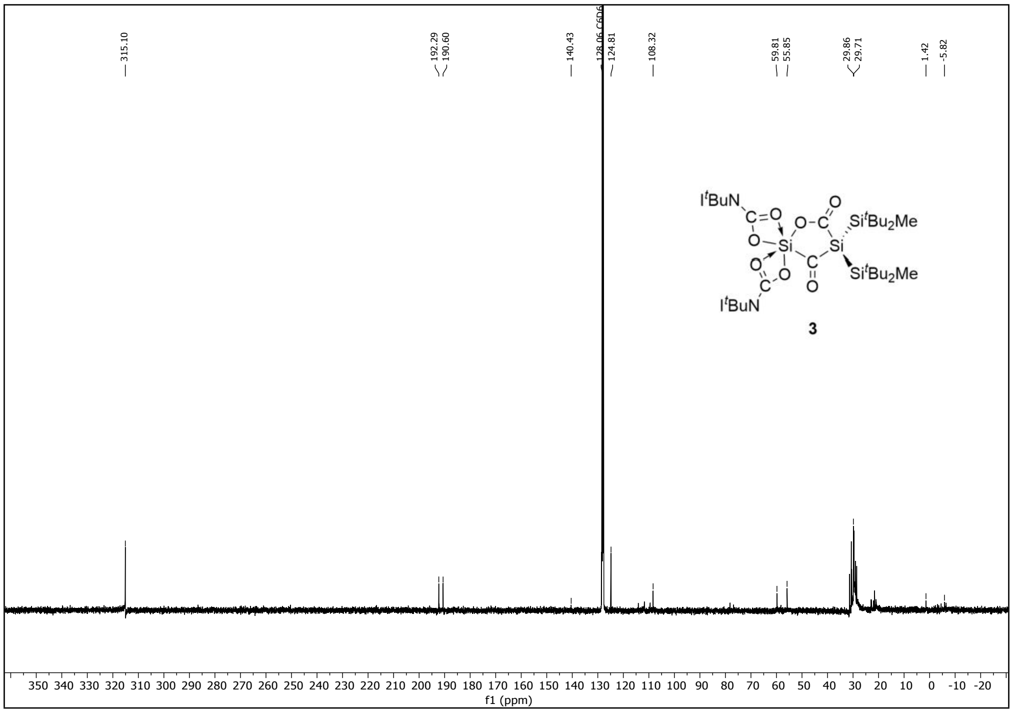

**Figure S27** $^{13}C$ NMR spectrum of **3** in benzene-$d_6$ at 300 K from labelled **2a**($^{13}CO$) with non-labelled $CO_2$.

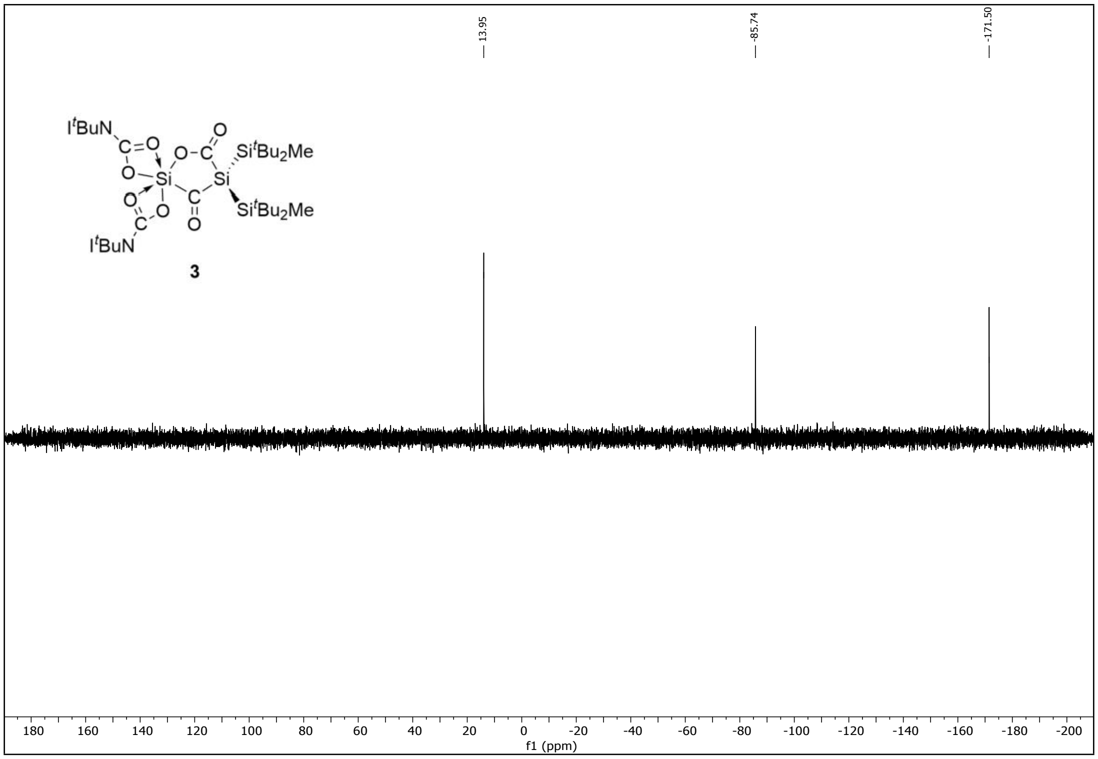


**Figure S28:** $^{29}$Si-ig NMR spectrum of **3** in pyridine-$d_5$ at 300 K.

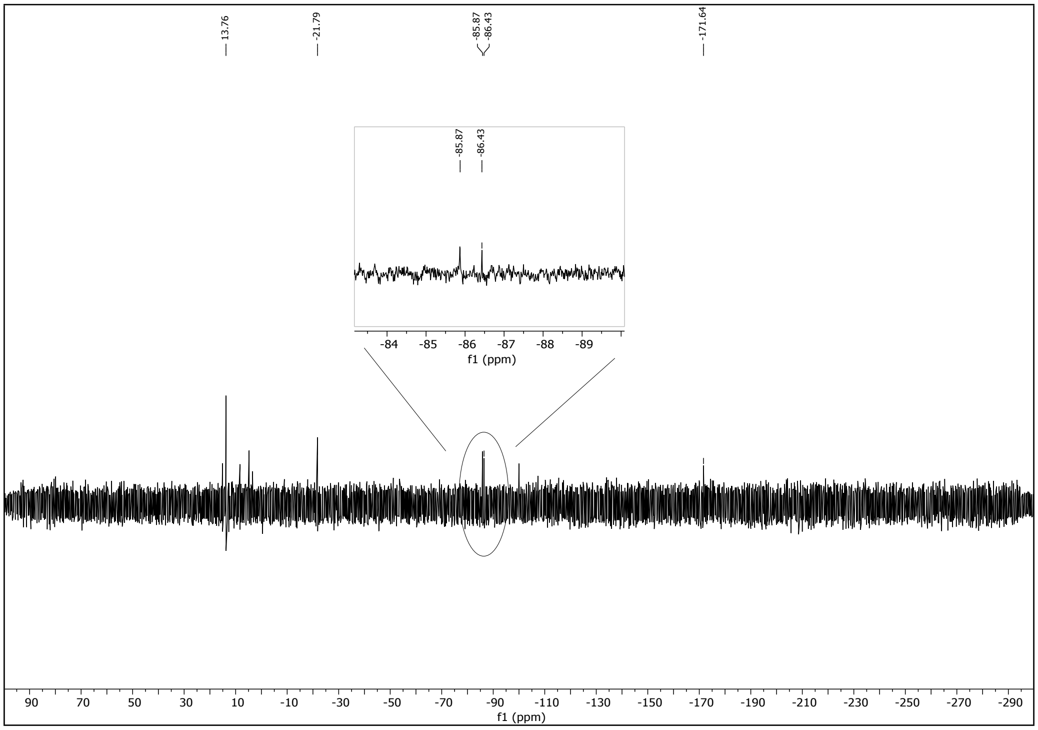


**Figure S29** $^{29}$Si NMR spectrum of **3** in $C_6D_6$ at 300 K with $^{13}CO_2$. Coupling constant is determined as δ = 86.2 ppm (d, *J* = 55.4 Hz, *<u>Si</u>*Si$_2$). An impurity of silicon grease at -21.8 ppm and an unknown impurity at -100.0 ppm can be detected.

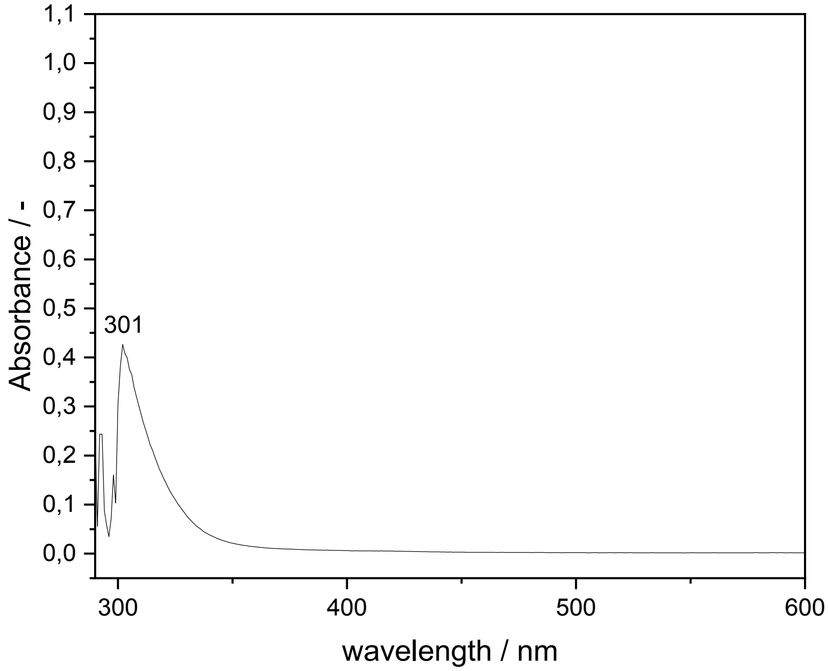


**Figure S30** UV-Vis spectrum of **3a** in pyridine ($3.04\times10^{-3}$ M), measured at room temperature ($\lambda_{max}$ = 301 nm).

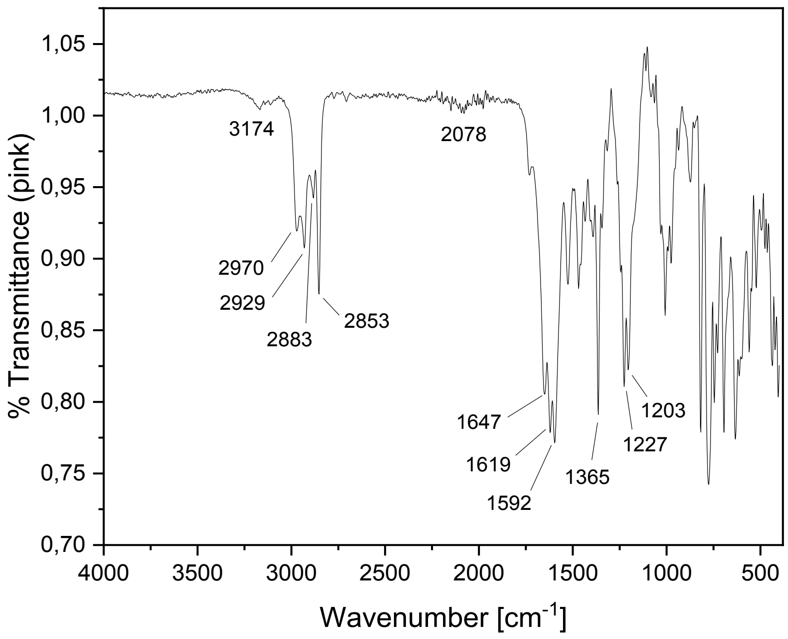


**Figure S31** Solid-state FT-IR spectrum of compound **3** at 298 K.

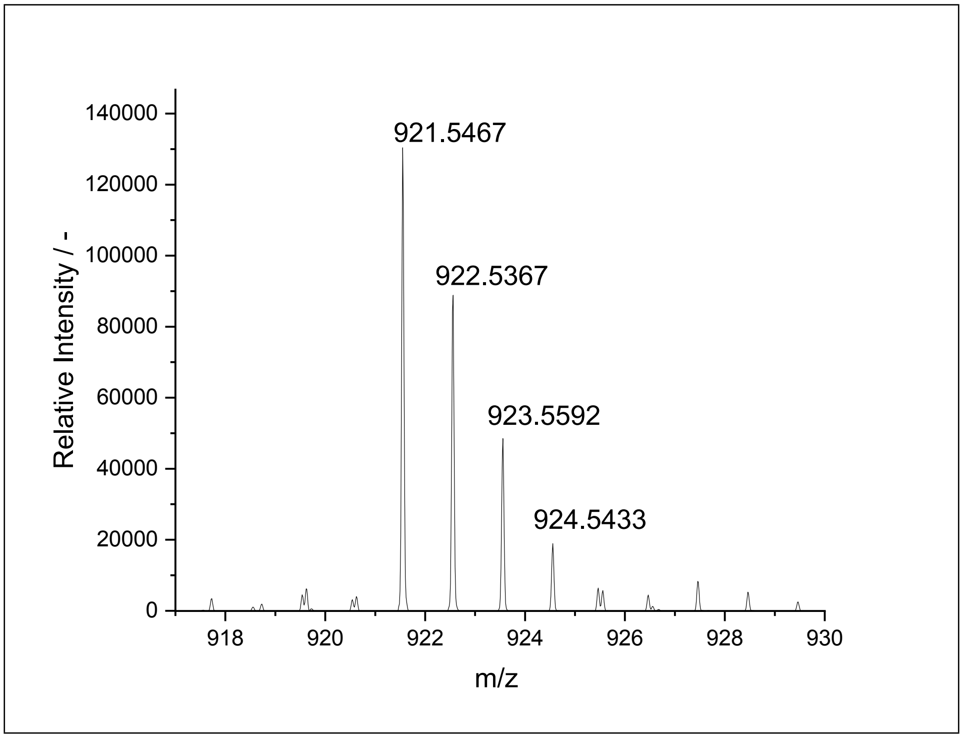


**Figure S32** Measured LIFDI-MS spectrum of **3.**

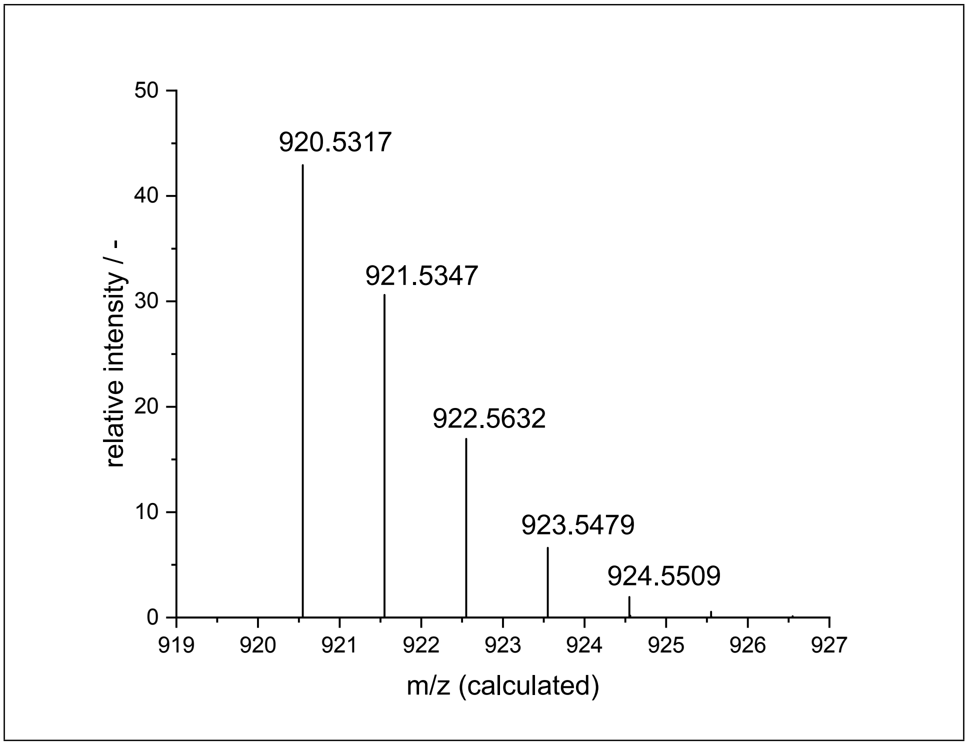


**Figure S33** Calculated MS spectrum of **3.**

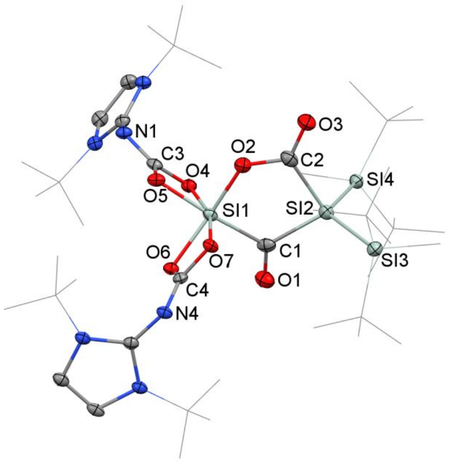


**Figure S34** SC-XRD structure of **3**, with thermal ellipsoids drawn at the 50% probability level. Hydrogen atoms are omitted for clarity, tBu- and Me-groups are simplified as wireframes. Selected bond lengths [Å] and angles [°]: bond lengths: C1-O1 1.203(3), C2-O2 1.313(4), C2-O3 1.217(4), Si1-C1 1.925, Si2-C1 1.992(3), Si1⋯Si2 3.239, Si2-Si3 2.420(3), Si2-Si4 2.384(8). Bond angles: Si1-C1-Si2 111.56°.

### 1.6 Double $CO_2$ insertion product: disila-bislactone 4

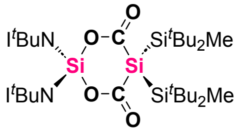


**4**
$Si_4C_{42}H_{82}N_6O_4$
847.50 g/mol

A solution of **3** (20.0 mg, µmol) in pyridine (0.2 mL) was left for several days at room temperature. The color of the solution changed from bright pink to pale yellow while crystals formed in a quantitative yield. These crystals were soluble in benzene-$d_6$ and pyridine-$d_5$ and were characterized *via* multinuclear NMR spectroscopy and SC-XRD, confirming the structure.

**$^1$H NMR (500 MHz, pyridine-d$_5$, 300 K):** δ [ppm] = 6.99 (s, 2H, C*H*-N), 6.61 (s, 2H, C*H*-N), 1.67 (s, 9H, N(C*H*$_3$)$_3$), 1.36 (s, 9H, N(C*H*$_3$)$_3$), 1.31 (s, 9H, N(C*H*$_3$)$_3$), 1.28 (s, 9H, N(C*H*$_3$)$_3$), 1.27 (s, 18H, SiC(C*H*$_3$)$_3$), 0.57 (s, 3H, Si(C*H*$_3$)), 0.50 (s, 3H, Si(C*H*$_3$)).
**$^{13}$C{$^1$H} NMR (126 MHz, benzene-d$_6$, 300 K):** δ [ppm] = 185.7 (C=O), 151.1 (C=N), 108.1 (CH-N), 107.6 (*C*H-N), 64.7 (N*C*(CH$_3$)$_3$, 55.8 (N*C*(CH$_3$)$_3$), 34.7 (Si*C*(CH$_3$)$_3$), 30.6 (NC(*C*H$_3$)$_3$), 29.7 (NC(*C*H$_3$)$_3$), 29.2 (NC(*C*H$_3$)$_3$), 28.8 (NC(*C*H$_3$)$_3$), 23.0 (SiC(*C*H$_3$)$_3$), 14.6 (SiC(*C*H$_3$)$_3$), 1.7 (Si*C*H$_3$), -2.0 (Si*C*H$_3$).
**$^{29}$Si{$^1$H} NMR (99 MHz, benzene-d$_6$, 300 K):** δ [ppm] = -12.8 (s, *Si*$^t$Bu$_2$Me), -79.0 (s, *Si*Si$_2$), -147.1 (s, N$_2$*Si*O$_2$).
**m.p.:** 86°C
**LIFDI-MS:** $Si_4C_{42}H_{82}N_6O_4$ calculated (found experimentally): 846.5317 (846.5403 m/z).

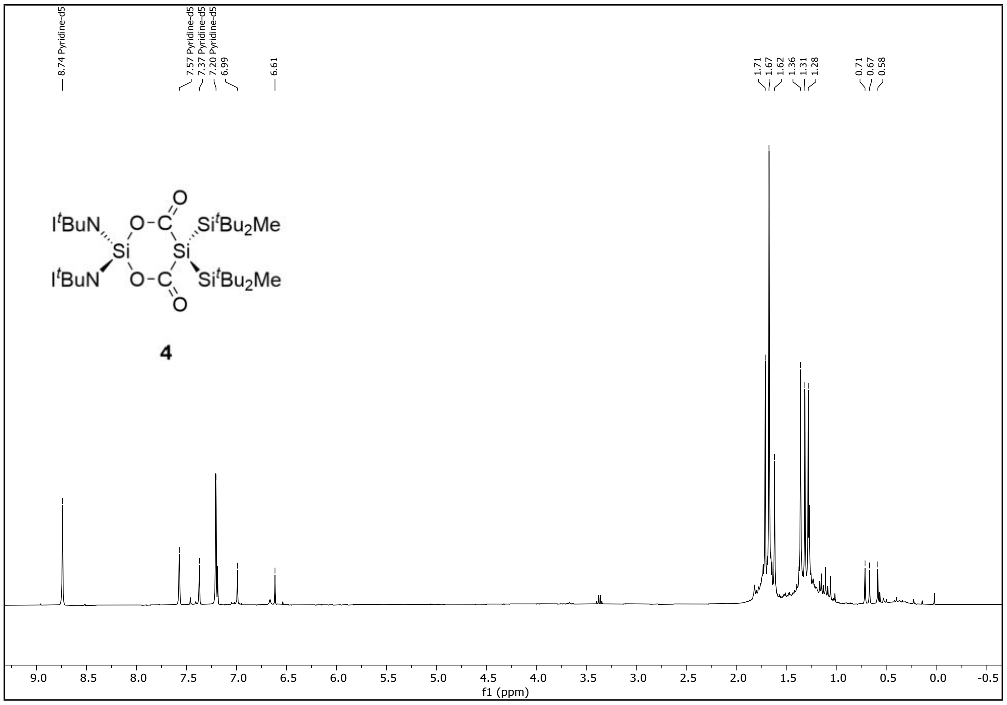


**Figure S35** $^1$H NMR spectrum of **4** in pyridine-d$_5$ at 300 K.

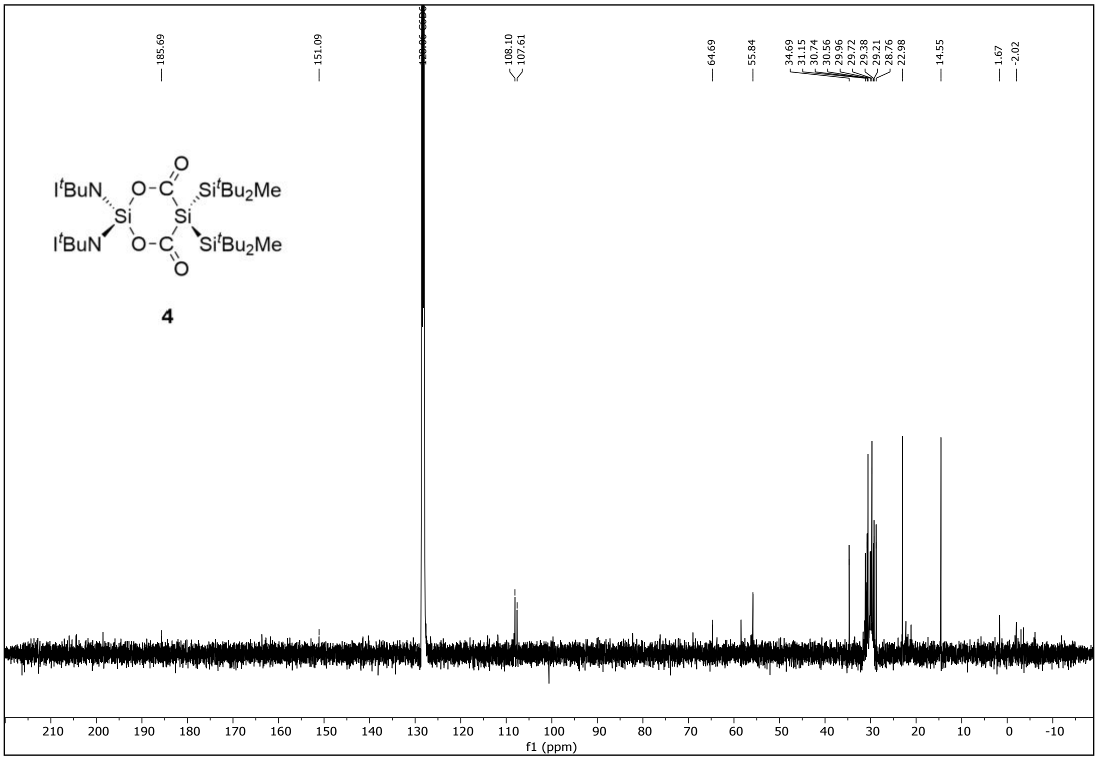


**Figure S36** $^{13}$C NMR spectrum of **4** in benzene-*d$_6$* at 300 K.

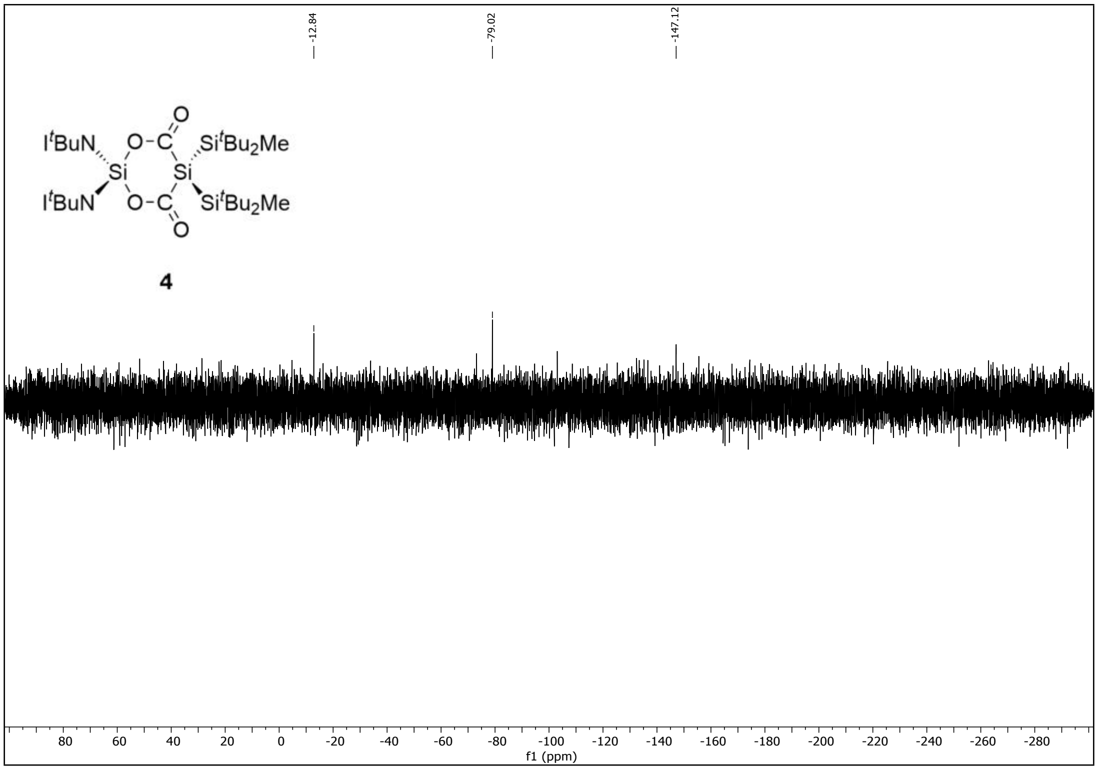


**Figure S37** $^{29}$Si NMR spectrum of **4** in benzene-$d_6$ at 300 K.

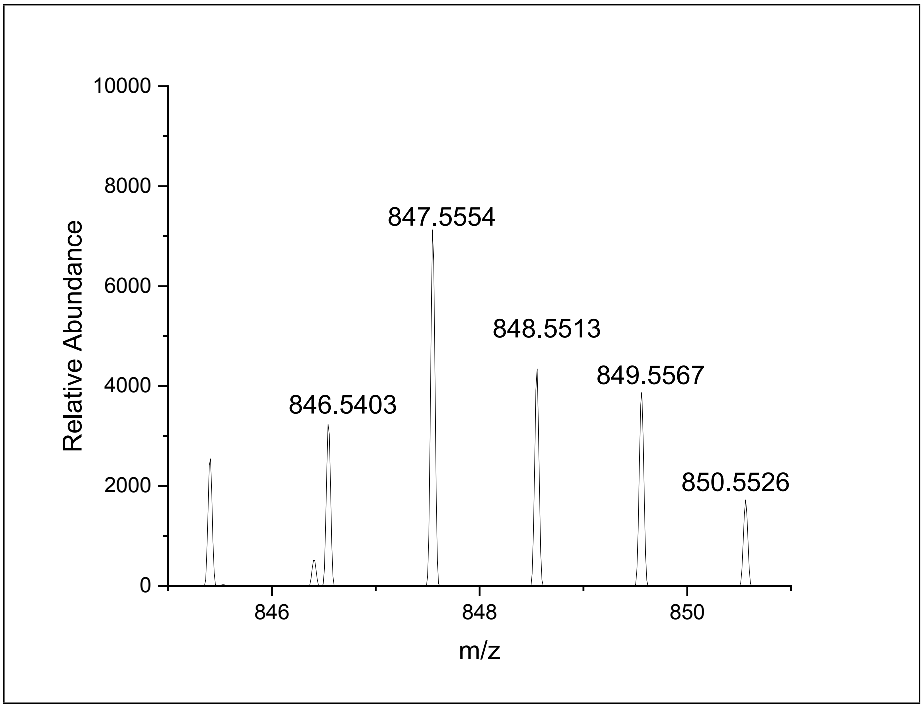


**Figure S38** Measured LIFDI-MS spectrum of **4.**

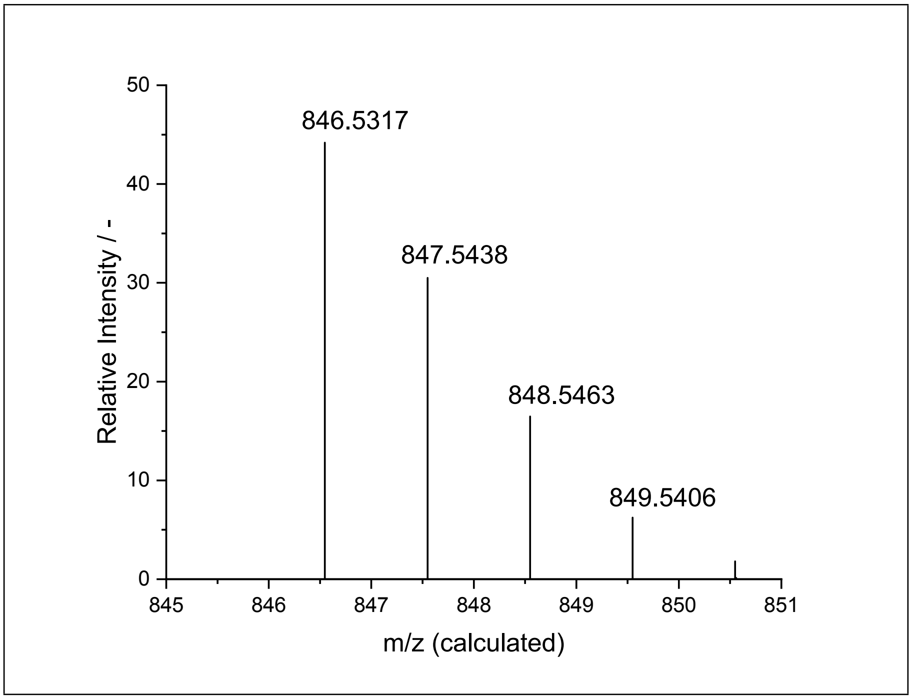


**Figure S39** Calculated LIFDI-MS spectrum of **4.**

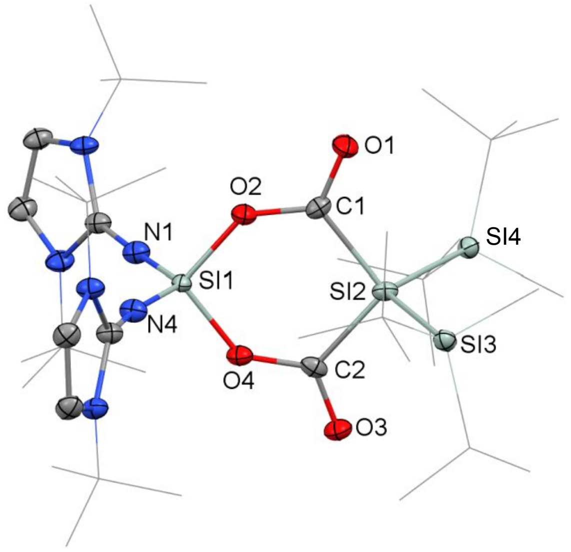


**Figure S40** SC-XRD structure of **4**, with thermal ellipsoids drawn at the 50% probability level. Hydrogen atoms are omitted for clarity, tBu- and Me-groups are simplified as wireframes. Selected bond lengths [Å] and angles [°]: bond lengths: C1-O1 1.234(4), C1-O2 1.302(4), Si2-C1 1.917(3), Si1-O2 1.682(3), Si1-N1 1.609(4), Si1-N4 1.614(3), Si2-Si3 2.411(1), Si2-Si4 2.433(7), Si1···Si2 3.547. Bond angles: O2-Si1-O4 102.7(1), C1-Si2-C2 101.9(1), N1-Si1-N4 119.0(2), Si3-Si2-Si4 117.44(5); twist N1/N4 vs Si3/Si4: 2.11°, twist N1/N4/Si3/Si4 vs $CO_2$: 87.89°.

## 1.7 Proposed mechanism for the formation of 4

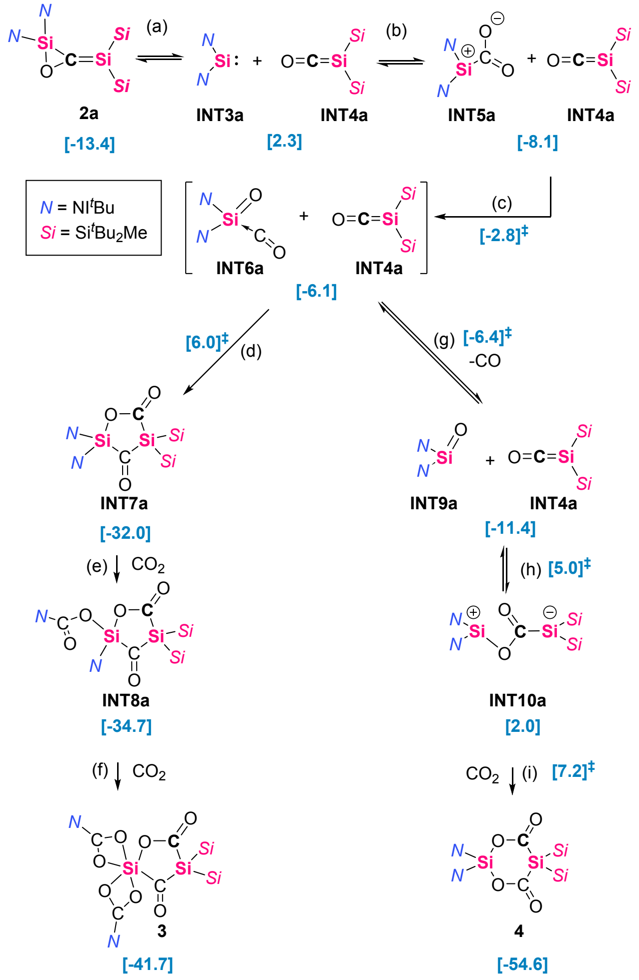


**Figure S41** Proposed mechanism for the formation of **3** and **4** from **2a**. Energies in blue are ΔG [kcal mol$^{-1}$].

To rationalize the formation of **3** and **4** from **2a** in the presence of $CO_2$, we propose a mechanism presented in Figure S41. According to this proposed mechanism [**INT6a+INT4a**] and **INT10a** are in thermodynamic equilibrium, and the formation of **INT10a** is kinetically preferred over the formation of **INT7a**. The conversion of[**INT6a+INT4a**] to **INT7a** via path (d) is irreversible and so is the conversion of **INT10a** to **4** via path (i). The ΔG‡ for these two processes (path (d) vs path (i)) is only 1.2 kcal mol$^{-1}$, and we think that it is reasonable to suggest that the outcome can be affected by the amount of $CO_2$ present in the reaction mixture. The modified Scheme 10 has been incorporated into an article. We also added the equilibrium arrows to emphasize that [**INT6a +**

**INT4a**], **INT9a + INT4a,** and **INT10a** are in thermodynamic equilibrium under the experimental conditions.

In the main text we state that **3** converts to **4** only when dissolved in pyridine. Experimentally, we tried to convert **3** to **4** using high $CO_2$ pressure (2.0 bar in benzene), but it didn't work. The reaction is calculated to be exergonic by 12.9 kcal $mol^{-1}$, which is a significant diving force. However, **3** does not convert to **4** spontaneously if left in benzene or heated up. Computationally, we considered numerous pathways for the transformation of **3** to **4**, but we couldn't find a reasonable pathway with barriers that can be overcome under the experimental conditions. Therefore, we assume that there is a complex mechanism in which pyridine plays a role, since experimental results suggest that.

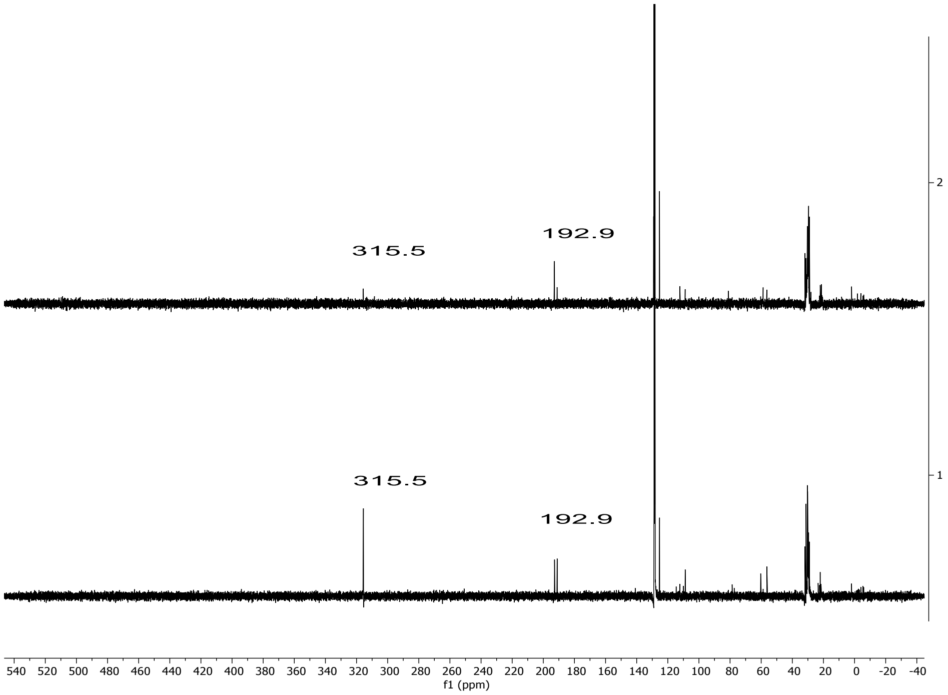


**Figure S42** $^{13}C$ NMR of reaction **2a** -> **4** in $C_6D_6$. The $Si_2C$=O disilyl ketone moiety is visible at 315.5 ppm (bottom), and its transfer to the $CO_2$-carbon of **4** is visible at 192.9 ppm (top).

## 1.8 Disilene $CO_2$ adduct 5

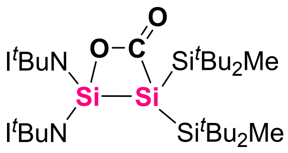


**5**

$Si_4C_{41}H_{82}N_6O_2$
M = 803,49 g/mol

Disilene **1a** (40.0 mg, 52.8 µmol, 1.0 eq) was dissolved in 0.4 mL of $C_6D_6$ in a pressurize-able *J-Young* NMR tube. The solution was freeze-pump-thawed-degassed three times, before addition of 1.0 bar of $CO_2$ at room temperature. After 10 minutes of reaction time, the deep red color vanished and gave a colorless solution. After removal of all volatiles, the residue was dried *in vacuo*, resulting in a white powder (37.5 mg, 94 % yield). Re-dissolving in toluene and storage at -35 °C yielded **5** as colorless crystals suitable for SC-XRD analysis

**$^1$H NMR (500 MHz, $C_6D_6$, 300 K):** δ [ppm] = 6.08 (s, 2H, NC*H*$^{CO2}$), 5.99 (s, 2H, NC*H*), 1.55 (s, 36H, NC(C*H*$_3$)$_3$), 1.35 (s, 36H, Si(C(C*H*$_3$)$_3$)$_2$CH$_3$), 0.67 (s, 6H, Si(C(CH$_3$)$_3$)$_2$C*H*$_3$).
**$^{13}$C{$^1$H} NMR (126 MHz, $C_6D_6$, 300 K):** δ [ppm] = 190.6 (O*C*=O), 139.4 (*C*=N), 108.7 (N*C*H), 107.6 (N*C*H), 55.8 (N*C*(CH$_3$)$_3$, 55.7 (N*C*(CH$_3$)$_3$, 31.2 (NC(*C*H$_3$)$_3$), 30.6 (NC(*C*H$_3$)$_3$), 30.4 (NC(*C*H$_3$)$_3$), 29.66 (NC(*C*H$_3$)$_3$), 23.6 (Si(*C*(CH$_3$)$_3$)$_2$(CH$_3$)), 22.5 (Si(*C*(CH$_3$)$_3$)$_2$(CH$_3$)), -2.0 (Si(C(CH$_3$)$_3$)$_2$(*C*H$_3$)).
**$^{29}$Si{$^1$H} NMR (99 MHz, $C_6D_6$, 300 K):** δ [ppm] = 16.4 (*Si*$^t$Bu$_2$Me), -57.3 (=*Si*Si$_2$), -87.5 (=*Si*N$_2$).

**m.p.:** 139 °C

**LIFDI-MS:** $Si_4C_{41}H_{82}N_6O_2$ calculated (found experimentally): 802.5573 (802.5405 m/z).

*

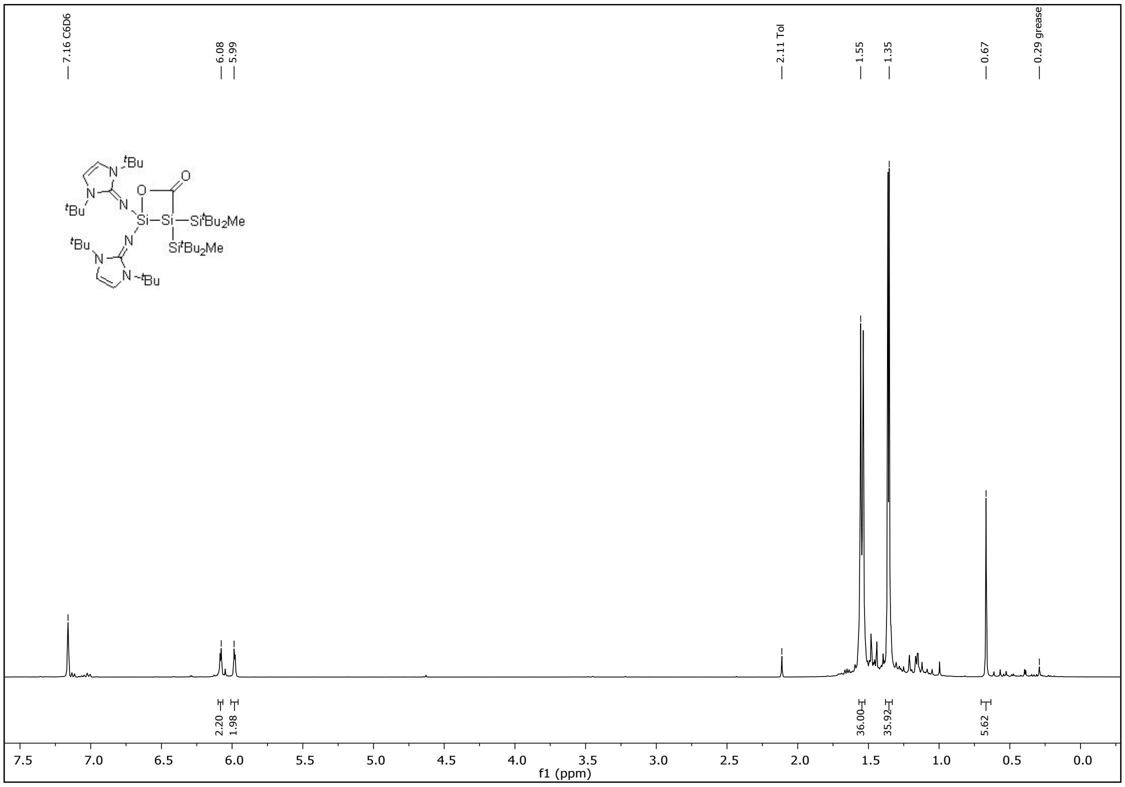

**Figure S43** $^1H$ NMR (400 MHz) spectrum of **5** in benzene-$d_6$ (300 K).

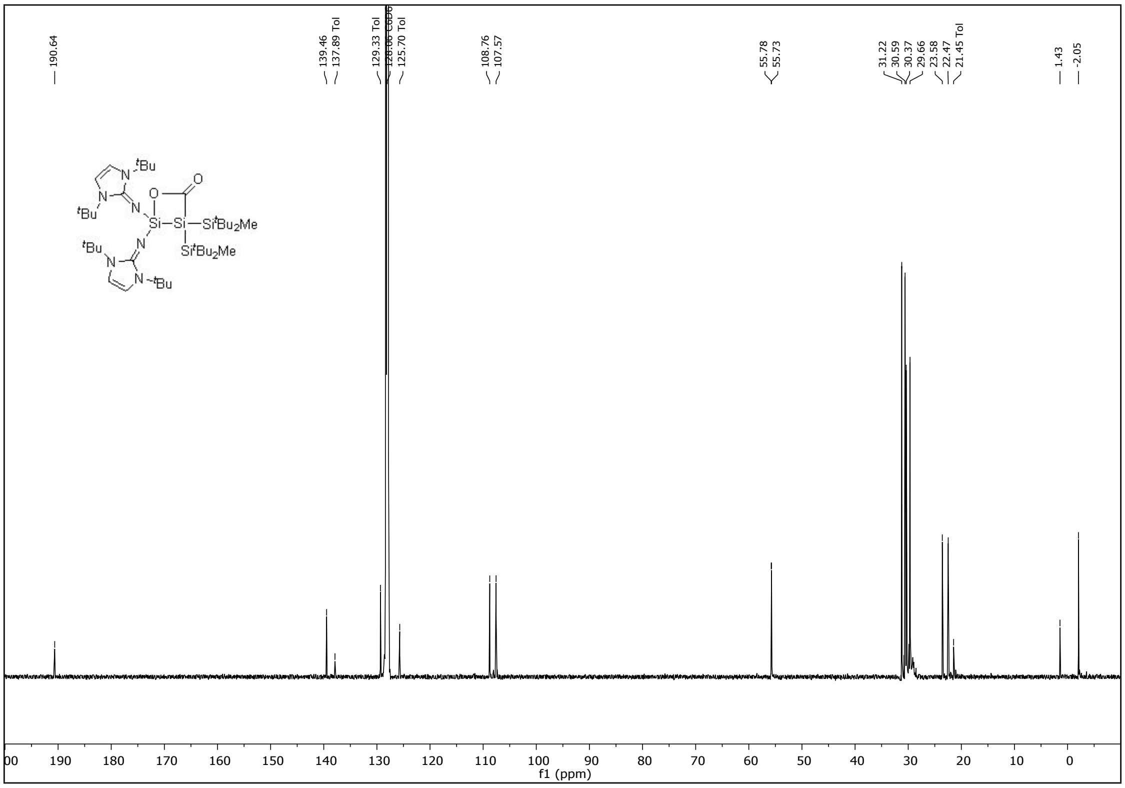

**Figure S44** $^{13}C$ NMR (126 MHz) spectrum of **5** in benzene-$d_6$ (300 K).

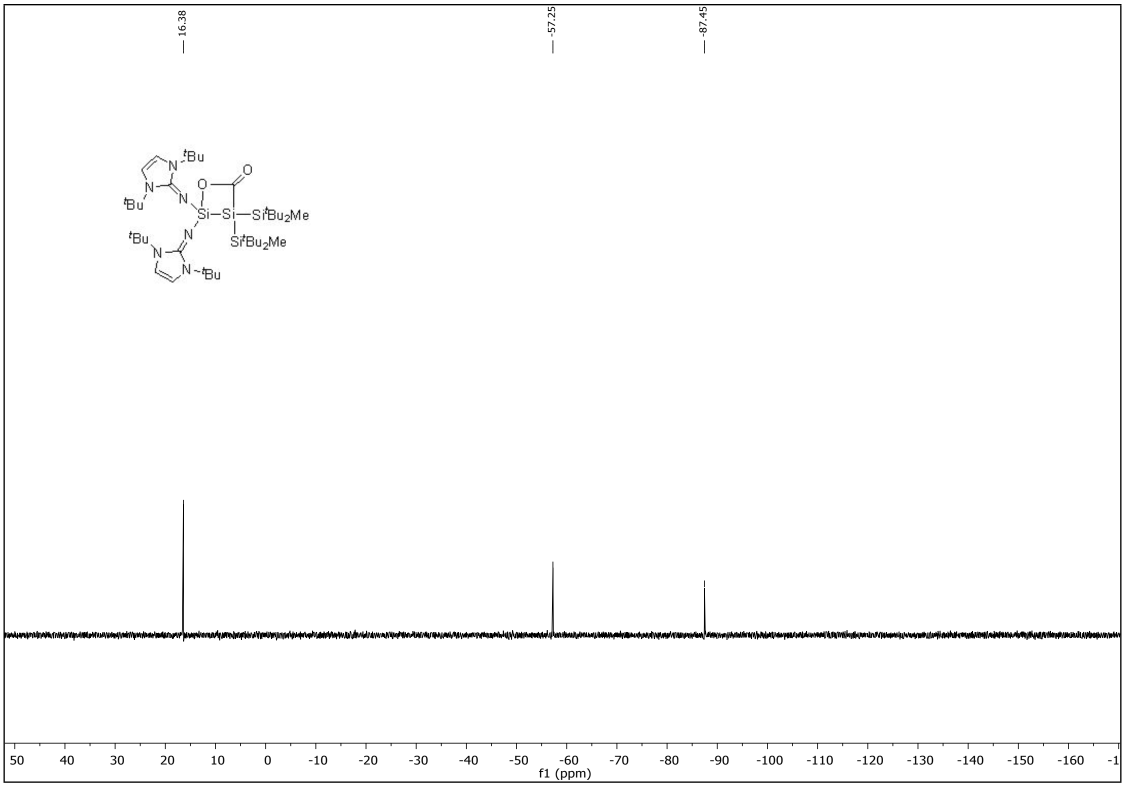


**Figure S45** $^{29}$Si NMR (99 MHz) spectrum of **5** in benzene-$d_6$ (300 K).

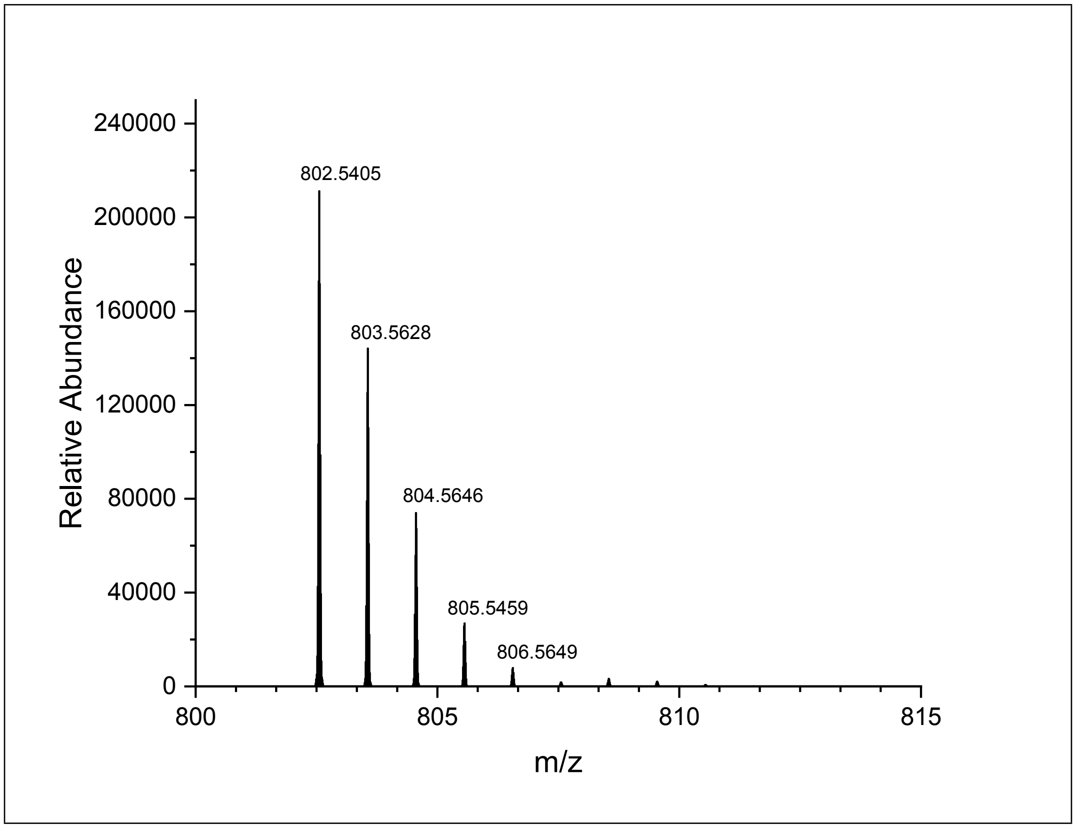


**Figure S46** Measured LIFDI-MS spectrum of **5.**

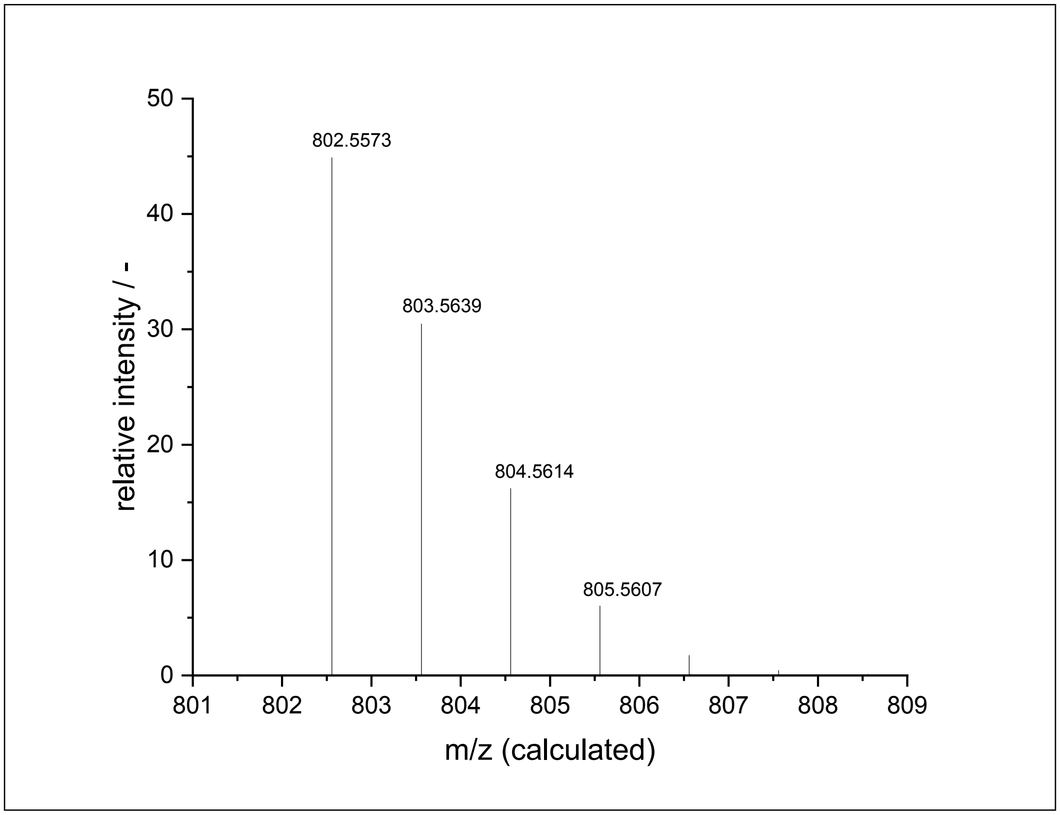


**Figure S47** Calculated mass spectrum of **5**.

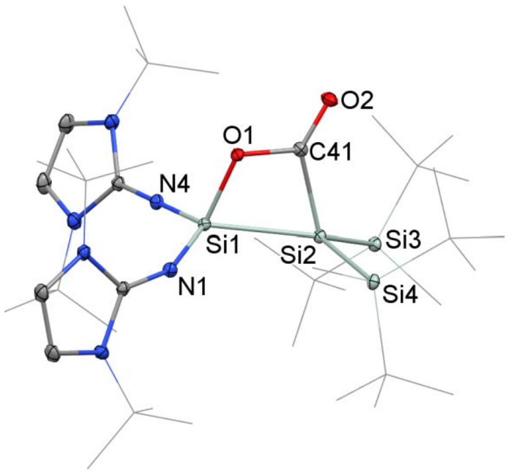


**Figure S48** SC-XRD structure of **5**, with thermal ellipsoids drawn at the 50% probability level. Hydrogen atoms are omitted for clarity, $^t$Bu- and Me-groups are simplified as wireframes. Selected bond lengths [Å] and angles [°]: bond lengths: Si1-Si2 2.4303(7), Si1-O1 1.744(1), Si1-N1 1.663(1), Si1-N4 1.646(1), Si2-Si3 2.4163(6), Si2-Si4 2.4248(7), Si2-C41 1.962(1), O1-C41 1.371(2), O2-C41 1.207(1). Bond angles: Si2-Si1-O1 76.53(4), Si1-Si2-C41 69.39(4), Si1-O1-C41 108.01(8), Si2-C41-O1 103.37(9), Si2-C41-O2

136.1(1), O1-C41-O2 120.5(1), N1-Si1-N4 115.28(7), Si3-Si2-Si4 117.38(2). Twist angle (O1-C41/Si1-Si2) 13.1°, twist angle (N1-S1-N4/Si3-Si2-Si4): 45.2°.

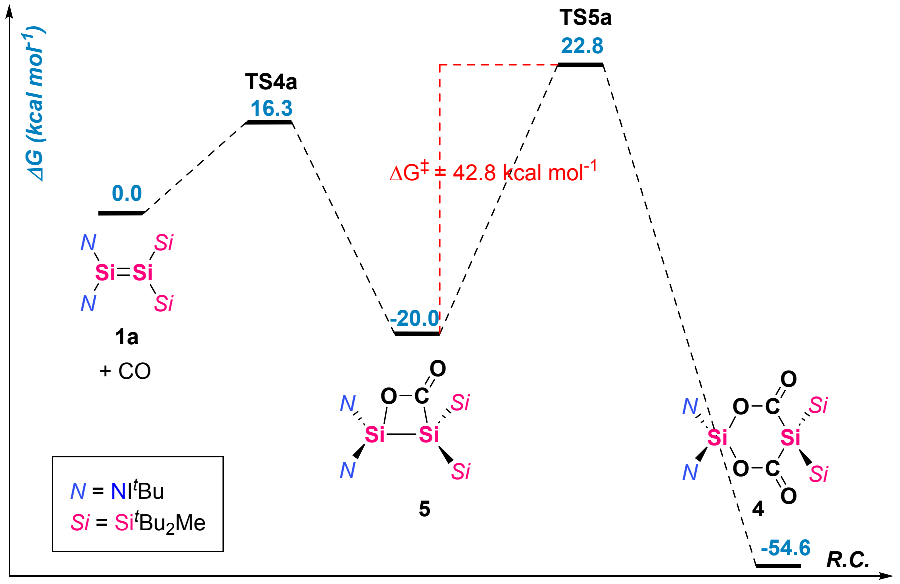


**Figure S49.** Calculated free energy diagram for the proposed reaction mechanism of **1a** with $CO_2$. Free energies are at the (SMD=Benzene)PW6B95-D4/def2-QZVPP//r$^2$SCAN-3c level of theory.

## 2. X-ray Crystallographic Data

### 2.1. General Information

The X-ray intensity data of **2a** were collected on an X-ray single crystal diffractometer equipped with a CCD detector (*Apex II CCD*), a fine-focus sealed tube and with MoKα radiation (λ = 0.71073 Å) and a *Triumph monochromator* by using the *APEX III* software package. The X-ray intensity data of **2b** were collected on an X-ray single crystal diffractometer equipped with a CMOS detector (*Bruker Photon-100*), a rotating anode (*Bruker IMS*) with MoKα radiation (λ = 0.71073 Å) and a *Helios* mirror optic by using the *APEX III* software package.[8] The measurements were performed on single crystals coated with the perfluorinated ether *Fomblin® Y*. The crystal was fixed on the top of a micro sampler, transferred to the diffractometer, and frozen under a stream of cold nitrogen (100 K). A matrix scan was used to determine the initial lattice parameters. Reflections were merged and corrected for Lorenz and polarization effects, scan speed, and background using *SAINT*.[9] Absorption corrections, including odd and even ordered spherical harmonics were performed using *SADABS*.[9] Space group assignments were based upon systematic absences, E statistics, and successful refinement of the structures. Structures were solved by direct methods with the aid of successive difference Fourier maps, and were refined against all data using the *APEX III* software in conjunction with *SHELXL-2014*[10] and *SHELXLE*.[11] All H atoms were placed in calculated positions and refined using a riding model, with methylene and aromatic C–H distances of 0.99 and 0.95 Å, respectively, and $U_{iso}(H) = 1.5 \cdot U_{eq}(C)$. Full-matrix least-squares refinements were carried out by minimizing $\Delta w(F_o^2 – F_c^2)$[9] with *SHELXL-97* weighting scheme.[12] Neutral atom scattering factors for all atoms and anomalous dispersion corrections for the non-hydrogen atoms were taken from International Tables for Crystallography.[13] Refinement of **2a** showed a whole-molecule rotational disorder around the central Si=C bond and was modeled in a disorder refinement, resulting in a high $R_1$ value. No residual electron density can be detected in the core structure and therefore the core structure can be described without reasonable doubt. The symmetry of **2b** was adjusted from *Pbcn* to *Pna*$2_1$, orthorhombic via ADDSYM in Platon.[14] **2b** was refined as an inversion twin with BASF 0.53852. Additionally, the $Si(Si^tBu_2Me)_2$ groups showed a rotational disorder around the central Si=C bond, and the disorder was modeled accordingly. Though **2b** shows one additional oxygen in the crystal structure, the presented structure was validated by additional analytical data (multinuclear NMR, LIFDI-MS spectroscopy, and FT-IR spectroscopy). For **3**, one of the $Si^tBu_2Me$ groups was modeled in a disorder refinement. For **4**, the symmetry was adjusted to P21/*c* via ADDSYM (Platon) and a $Si^tBu_2Me$ group was modeled in a disorder refinement. The final cif files

were generated via *Platon* function <table acc>[14] and finalcif.[15] The images of the crystal structures were generated by *Mercury*.[16] The CCDC numbers 2416183-2416187 contain the supplementary crystallographic data for the structures. The data can be obtained free of charge from the Cambridge Crystallographic Data Centre *via* https://www.ccdc.cam.ac.uk/structures/.

## 2.2 SC-XRD structures

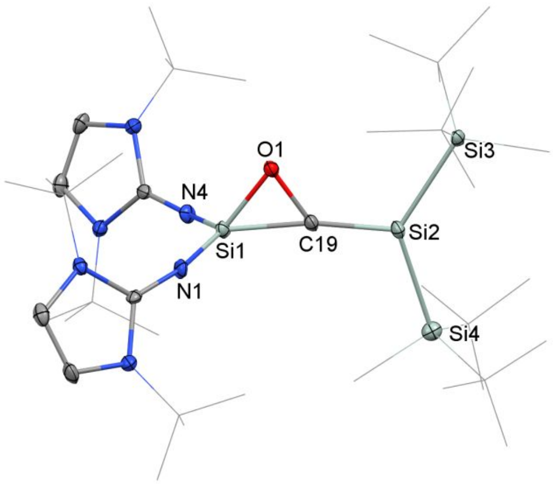


**Figure S50** SC-XRD structure of **2a**, with thermal ellipsoids drawn at the 50% probability level. Hydrogen atoms are omitted for clarity, *t*Bu- and Me-groups are simplified as wireframes. Selected bond lengths [Å] and angles [°]: bond legths: Si1-C19 1.799(11), C19-Si2 1.761(13), Si1-O1 1.700(10), O1-C19 1.427(14); bond angles: O1-Si1-C19 48.0(5), O1-C19-Si1 62.3(6), Si1-O1-C19 69.6(6), Si1-C19-Si2 168.9(14); twist angle: 89.96° (N1, N4 / Si3, Si4).

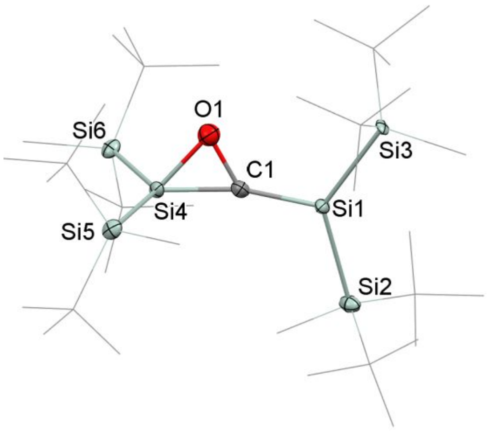


**Figure S51** SC-XRD structure of **2b**, with thermal ellipsoids drawn at the 50% probability level. Hydrogen atoms are omitted for clarity, *t*Bu- and Me-groups are simplified as wireframes. Selected bond lengths [Å] and angles [°]: bond lengths: Si4-C1 1.781(10), C1-Si1 1.786(10), Si4-O1 1.727(12), O1-C1 1.401(12); bond angles: O1-Si4-C1 47.0(4), O1-C1-Si4 64.5(6), Si4-O1-C1 68.5(6), Si4-C1-Si1 167.4(6); ligand twisting: 85.94° (Si5, Si6 / Si2, Si3).

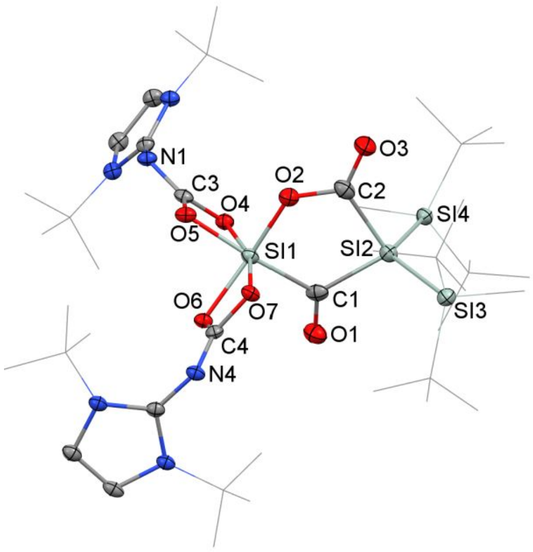


**Figure S52** SC-XRD structure of **3**, with thermal ellipsoids drawn at the 50% probability level. Hydrogen atoms are omitted for clarity, tBu- and Me-groups are simplified as wireframes. Selected bond lengths [Å] and angles [°]: bond lengths: C1-O1 1.203(3), C2-O2 1.313(4), C2-O3 1.217(4), Si1-C1 1.925, Si2-C1 1.992(3), Si1···Si2 3.239, Si2-Si3 2.420(3), Si2-Si4 2.384(8). Bond angles: Si1-C1-Si2 111.56°.

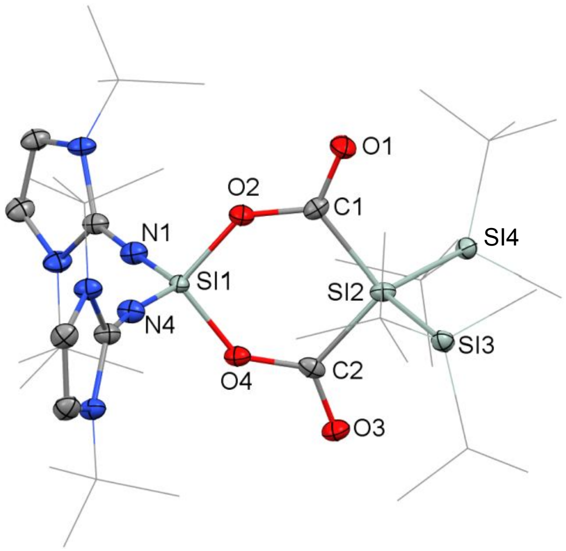


**Figure 53** SC-XRD structure of **4**, with thermal ellipsoids drawn at the 50% probability level. Hydrogen atoms are omitted for clarity, tBu- and Me-groups are simplified as wireframes. Selected bond lengths [Å] and angles [°]: bond lengths: C1-O1 1.234(4), C1-O2 1.302(4), Si2-C1 1.917(3), Si1-O2 1.682(3), Si1-N1 1.609(4), Si1-N4 1.614(3), Si2-Si3 2.411(1), Si2-Si4 2.433(7), Si1···Si2 3.547. Bond angles: O2-Si1-O4

102.7(1), C1-Si2-C2 101.9(1), N1-Si1-N4 119.0(2), Si3-Si2-Si4 117.44(5); twist N1/N4 vs Si3/Si4: 2.11°, twist N1/N4/Si3/Si4 vs $CO_2$: 87.89°.

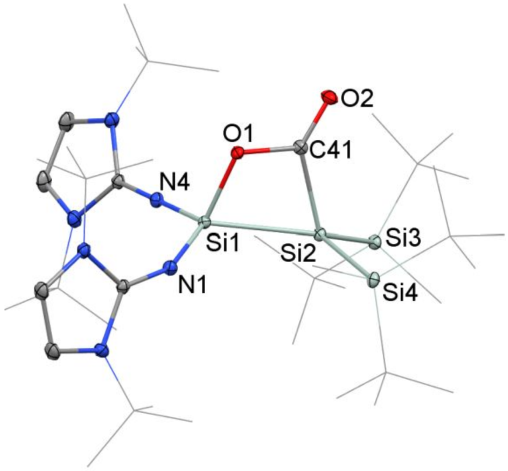


**Figure S54** SC-XRD structure of **5**, with thermal ellipsoids drawn at the 50% probability level. Hydrogen atoms are omitted for clarity, $^t$Bu- and Me-groups are simplified as wireframes. Selected bond lengths [Å] and angles [°]: bond lengths: Si1-Si2 2.4303(7), Si1-O1 1.744(1), Si1-N1 1.663(1), Si1-N4 1.646(1), Si2-Si3 2.4163(6), Si2-Si4 2.4248(7), Si2-C41 1.962(1), O1-C41 1.371(2), O2-C41 1.207(1). Bond angles: Si2-Si1-O1 76.53(4), Si1-Si2-C41 69.39(4), Si1-O1-C41 108.01(8), Si2-C41-O1 103.37(9), Si2-C41-O2 136.1(1), O1-C41-O2 120.5(1), N1-Si1-N4 115.28(7), Si3-Si2-Si4 117.38(2). Twist angle (O1-C41/Si1-Si2) 13.1°, twist angle (N1-S1-N4/Si3-Si2-Si4): 45.2°.

## 2.3 Crystal data and structural refinement parameters

**Table S1** Structural refinement parameters for the SC-XRD structures **2a** and **2b.**

| | **2a** | **2b** |
|---|---|---|
| CCDC Number | 2416185 | 2416184 |
| **Crystal Data** | | |
| Chemical formula | $C_{27.33}H_{54.67}N_4O_{0.67}Si_{2.67}$ | $C_{37}H_{84}OSi_6$ |
| $M_r$ | 524.99 | 729.58 |
| Crystal system, space group | Monoclinic, $P2_1/c$ | Orthorhombic, $Pna2_1$ |
| Temperature (K) | 100 | 100 |
| *a*, *b*, *c* (Å) | 15.021(3), 11.800(2), 27.186(5) | 23.3107 (12), 17.2938 (9), 11.7097 (6) |
| α, β, γ (°) | 90, 90.145(7), 90 | 90, 90, 90 |
| *V* (Å$^3$) | 4818.6(15) | 4720.5 (4) |
| *Z* | 6 | 4 |
| *F*(000) | 1736 | 1624 |
| Radiation type | Mo *Kα* | Mo *K*a |
| No. of reflections for cell measurement | 9611 | 9788 |
| θ range (°) for cell measurement | 2.71– 51.39 (0.82 Å) | 2.2748–25.6621 |
| μ (mm$^{-1}$) | 0.16 | 0.20 |
| Crystal shape | fragment | fragment |
| Colour | pale-yellow | clear yellow |
| Crystal size (mm) | 0.420 × 0.311 × 0.259 | 0.94 × 0.72 × 0.33 |
| **Data Collection** | | |
| Diffractometer | Apex II CCD | Bruker Photon CMOS IMS microsource |
| Radiation source | fine-focus sealed tube | fine-focus sealed tube |
| Detector resolution (p mm$^{-1}$) | 16 | 16 |
| Scan method | phi– and omega–rotation scans | phi– and omega–rotation scans |
| Absorption correction | Multi-scan | Multi-scan |
| $T_{min}$, $T_{max}$ | 0.6945, 0.7453 | 0.6675, 0.7453 |
| No. of measured, independent and observed [*I* > 2s(*I*)] reflections | 178988, 9176, 8632 | 106512, 10823, 9224 |
| $R_{int}$ | 0.052 | 0.052 |
| θ values (°) | $θ_{max}$ = 25.69, $θ_{min}$ = 1.36 | $θ_{max}$ = 27.51, $θ_{min}$ = 2.10 |
| $(\sin θ/λ)_{max}$ (Å$^{-1}$) | 0.610 | 0.650 |
| Range of *h*, *k*, *l* | *h* = -18→17, *k* = -14→14, *l* = -33→33 | *h* = -30→30, *k* = -20→22, *l* = -15→15 |
| **Refinement** | | |
| Refinement on | $F^2$ | $F^2$ |
| $R[F^2 > 2s(F^2)]$, $wR(F^2)$, *S* | 0.163, 0.334, 1.35 | 0.092, 0.274, 1.06 |
| No. of reflections | 9176 | 10823 |
| No. of parameters | 726 | 808 |
| No. of restraints | 663 | 1633 |

| | | |
|---|---|---|
| H-atom treatment | H-atom parameters constrained | H-atom parameters constrained |
| Weighting scheme | $\omega = 1/[\sigma^2(F_o^2) + 56.8129P]$ where $P = (F_o^2 + 2F_c^2)/3$ | $\omega = 1/[\sigma^2(F_o^2) + (0.1501P)^2 + 11.6774P]$ where $P = (F_o^2 + 2F_c^2)/3$ |
| $\Delta\rho_{max}$, $\Delta\rho_{min}$ (e Å$^{-3}$) | 0.63, –1.10 | 0.87, −1.39 |
| Absolute structure | – | Refined as an inversion twin |
| Absolute Structure parameter | – | 0.5 (4) |

**Table S2** Structural refinement parameters for the SC-XRD structures **3, 4** and **5.**

| | **3** | **4** | **5** |
|---|---|---|---|
| CCDC Number | 2416186 | 2416187 | 2416183 |
| **Crystal Data** | | | |
| Chemical formula | $C_{44}H_{81.88}O_7Si_4$, 2($C_6H_6$) | $C_{42}H_{82}N_6O_4Si_4$ | $C_{41}H_{82}N_6O_2Si_4$ |
| $M_r$ | 1075.61 | 847.50 | 803.48 |
| Crystal system, space group | orthorhombic, $Pbca$ | monoclinic, $P2_1/c$ | monoclinic, $P2_1/n$ |
| Temperature (K) | 100 | 100 | 100 |
| *a*, *b*, *c* (Å) | 18.6830(19), 18.3547(16), 35.749(4) | 12.0160(15), 17.864(2), 23.493(3) | 11.1353(11), 32.474(3), 13.2808(13) |
| α, β, γ (°) | 90, 90, 90 | 90, 102.426(4), 90 | 90, 90.835(3), 90 |
| $V$ (Å$^3$) | 12259(2) | 4924.7(10) | 4802.0(8) |
| $Z$ | 8 | 4 | 4 |
| $F$(000) | 4671 | 1856 | 1768 |
| Radiation type | Mo $K\alpha$ (λ=0.71073 Å) | Mo $K\alpha$ (λ=0.71073 Å) | Mo $K\alpha$ (λ=0.71073 Å) |
| θ range (°) for cell measurement | 3.31 to 50.80 (0.83 Å) | 4.15 to 52.83 (0.80 Å) | 3.87 to 50.70 (0.83 Å) |
| $\rho_{calc}$ [gcm$^{-3}$] | 1.166 | 1.143 | 1.111 |
| μ (mm$^{-1}$) | 0.149 | 0.164 | 0.162 |
| Crystal shape | fragment | needle | fragment |
| Colour | pink | colorless | colorless |
| Crystal size (mm) | 0.232×0.218×0.086 | 0.530×0.324×0.212 | 0.351×0.323×0.295 |
| Index ranges | −22 ≤ h ≤ 22<br>−22 ≤ k ≤ 22<br>−43 ≤ l ≤ 43 | −15 ≤ h ≤ 15<br>−22 ≤ k ≤ 22<br>−29 ≤ l ≤ 29 | −13 ≤ h ≤ 13<br>−39 ≤ k ≤ 39<br>−15 ≤ l ≤ 15 |
| Reflections collected | 279304 | 220305 | 71847 |
| Independent reflections | 11270<br>$R_{int}$ = 0.1072<br>$R_{sigma}$ = 0.0323 | 10050<br>$R_{int}$ = 0.0804<br>$R_{sigma}$ = 0.0277 | 8791<br>$R_{int}$ = 0.0383<br>$R_{sigma}$ = 0.0213 |
| Completeness to θ = 25.242° | 99.9 % | 99.4 % | 100.0 % |
| Data / Restraints / Parameters | 11270/313/880 | 10050/1251/639 | 8791/0/504 |
| Absorption correction $T_{min}/T_{max}$ (method) | 0.6773/0.7452 (multi-scan) | 0.69/0.7416 (multi-scan) | 0.698/0.7452 (multi-scan) |
| Goodness-of-fit on $F^2$ | 1.087 | 1.101 | 1.082 |
| Final $R$ indexes [$I$≥2σ($I$)] | $R_1$ = 0.0559<br>w$R_2$ = 0.1374 | $R_1$ = 0.0717<br>w$R_2$ = 0.1874 | $R_1$ = 0.0339<br>w$R_2$ = 0.0829 |
| Final $R$ indexes [all data] | $R_1$ = 0.0824<br>w$R_2$ = 0.1572 | $R_1$ = 0.0870<br>w$R_2$ = 0.1985 | $R_1$ = 0.0370<br>w$R_2$ = 0.0844 |
| Largest peak/hole [eÅ$^{-3}$] | 0.71/−0.34 | 1.15/−0.58 | 0.37/−0.25 |

## 3 Computational Details

Calculations were carried out using ORCA software.[17]

Geometry optimizations were carried using the r$^2$SCAN-3c composite method,[18] utilizing the regularized and restored SCAN functional,[19,20] geometrical counterpoise correction gCP,[21] the atom-pairwise dispersion correction based on tight binding partial charges (D4), [22–24] the def2-mTZVPP basis set and def2-mTZVPP/J auxiliary basis set.[18]

The optimized geometries were verified as minima or transition states by analytical frequency calculations. The transition states were additionally verified by IRC calculations. Single point calculations of the optimized geometries were carried out at the r$^2$SCAN-3c level in benzene using the SMD solvation module[25] to obtain electrostatic contribution and the cavity term to account for the solvent effects.

To get more accurate electronic energies for the mechanistic investigations, single point calculations of the r$^2$SCAN-3c optimized geometries were carried using the PW6B95 [26] functional, with D4 dispersion correction, the def2-QZVPP [27] basis set and def2/J[28] and def2-QZVPP/C[29] auxiliary basis sets. The level at which the free energies were obtained is denoted as (SMD=Benzene)PW6B95-D4/def2-QZVPP//r$^2$SCAN-3c. The summary of the thermochemistry results is presented in Table S1.

Properties calculations and NBO analysis were carried out at the PBE0[30] -D4/def2-TZVP[27]// r$^2$SCAN-3c level of theory. NBO analysis was carried out using NBO 7 software.[31]

**Table S3 Calculated energies ($E_h$). Thermochemistry at 298.15 K.**
$E_{PW6B95}$ - electronic energy at the PW6B95-D4/def2-QZVPP//r$^2$SCAN-3c level; G-$E_{el}$ - Gibbs energy minus the electronic energy at the r$^2$SCAN-3c//r$^2$SCAN-3c level; $G_{cds}$ - cavity term and $G_{enp}$ - electrostatic contribution at the r$^2$SCAN-3c(SMD=Beneze)//r$^2$SCAN-3c level; $G_{conc}$ - concentration-induced free-energy shift ($G_{conc}$ = $RT\ln(24.5)$); $G_{PW6B95}$ - free energy at the (SMD=Benzene)PW6B95-D4/def2-QZVPP/ //r$^2$SCAN-3c level ($G_{PW6B95}$ = $E_{PW6B95}$ + [G-$E_{el}$] + $G_{cds}$ + $G_{enp}$ + $G_{conc}$).

| FNA | Compound | $E_{PW6B95}$ | G-$E_{el}$(298.15K) | $G_{cds}$(Benzene) | $G_{enp}$(Benzene) | $G_{conc}$(298.15K) | $G_{PW6B95}$ |
|---|---|---|---|---|---|---|---|
| 605511 | CO | -113.50052 | -0.01413 | -0.00046 | 0.00476 | 0.00302 | -113.50733 |
| 3692037 | **1a** | -3064.75425 | 1.08364 | -0.01575 | -0.02199 | 0.00302 | -3063.70532 |
| 3891079 | **TS1a** | -3178.24533 | 1.08769 | -0.01624 | -0.02237 | 0.00302 | -3177.19323 |
| 3881988 | **INT1a** | -3178.24453 | 1.08891 | -0.01553 | -0.02295 | 0.00302 | -3177.19108 |
| 3889227 | **TS2a** | -3178.24386 | 1.08969 | -0.01537 | -0.02323 | 0.00302 | -3177.18975 |
| 3895740 | **INT2a** | -3178.24240 | 1.08895 | -0.01500 | -0.02396 | 0.00302 | -3177.18939 |
| 3946600 | **TS3a** | -3178.23521 | 1.08777 | -0.01717 | -0.02668 | 0.00302 | -3177.18827 |
| 3690991 | **2a** | -3178.27863 | 1.08613 | -0.01621 | -0.02826 | 0.00302 | -3177.23395 |

| | | | | | | | |
|---|---|---|---|---|---|---|---|
| 3666833 | **1b** | -3163.72131 | 1.04658 | -0.00813 | -0.01347 | 0.00302 | -3162.69331 |
| 3690085 | **TS1b** | -3277.19979 | 1.05283 | -0.00862 | -0.01365 | 0.00302 | -3276.16621 |
| 3690193 | **INT1b** | -3277.20519 | 1.05270 | -0.00971 | -0.01416 | 0.00302 | -3276.17335 |
| 3674620 | **TS2b** | -3277.20218 | 1.05365 | -0.01007 | -0.01428 | 0.00302 | -3276.16985 |
| 3680320 | **INT2b** | -3277.22927 | 1.05344 | -0.01011 | -0.01439 | 0.00302 | -3276.19732 |
| 602032 | **TS3b** | -3277.21813 | 1.05402 | -0.00981 | -0.01398 | 0.00302 | -3276.18488 |
| 3673290 | **INT3b** | -3277.22230 | 1.05389 | -0.00954 | -0.01414 | 0.00302 | -3276.18907 |
| 3665806 | **TS4b** | -3277.20491 | 1.05166 | -0.00941 | -0.01461 | 0.00302 | -3276.17425 |
| 3672061 | **2b** | -3277.26630 | 1.04941 | -0.00976 | -0.01443 | 0.00302 | -3276.23807 |
| *3927528* | **TS4a** | -3253.64047 | 1.09539 | -0.01728 | -0.02129 | 0.00302 | -3252.58062 |
| *600812* | **5** | -3253.69933 | 1.09741 | -0.01724 | -0.02236 | 0.00302 | -3252.63850 |
| *3928842* | **TS5a** | -3442.54288 | 1.10651 | -0.01756 | -0.02375 | 0.00302 | -3441.47466 |
| 3897680 | **INT3a** | -1482.84517 | 0.54241 | -0.01400 | -0.02209 | 0.00302 | -1482.33583 |
| 4031755 | **INT4a** | -1695.37551 | 0.51362 | -0.00572 | -0.00906 | 0.00302 | -1694.87365 |
| 1732464 | **INT5a** | -1671.77137 | 0.55399 | -0.01720 | -0.02158 | 0.00302 | -1671.25314 |
| 1836677 | **TS[INT5a_INT6a]** | -1671.76398 | 0.55311 | -0.01636 | -0.02046 | 0.00302 | -1671.24467 |
| 1836679 | **INT6a** | -1671.76782 | 0.55180 | -0.01583 | -0.02123 | 0.00302 | -1671.25006 |
| 1732805 | **INT7a** | -3367.22559 | 1.10035 | -0.01851 | -0.02420 | 0.00302 | -3366.16493 |
| 1720410 | **INT8a** | -3556.13892 | 1.11220 | -0.02252 | -0.02419 | 0.00302 | -3555.07041 |
| 1715506 | **3** | -3745.05575 | 1.12653 | -0.02893 | -0.02779 | 0.00302 | -3743.98292 |
| 1830838 | **INT9a** | -1558.26062 | 0.54670 | -0.01905 | -0.02115 | 0.00302 | -1557.75109 |
| 1983986 | **TS[INT9a+INT4a_INT10a]** | -3253.64960 | 1.09113 | -0.01926 | -0.02386 | 0.00302 | -3252.59856 |
| 1979336 | **INT10a** | -3253.65361 | 1.09149 | -0.02115 | -0.02264 | 0.00302 | -3252.60289 |
| 1991494 | **TS[INT10a_4]** | -3442.55432 | 1.10060 | -0.01971 | -0.02600 | 0.00302 | -3441.49640 |
| 3927564 | **4** | -3442.65977 | 1.10635 | -0.01828 | -0.02621 | 0.00302 | -3441.59490 |

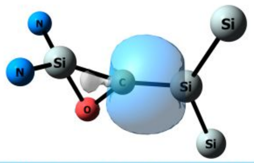


NBO 83. BD(1) Si2-C6 occ. 1.98
27.14% Si2 sp2.57
72.86% C6 sp0.97

| Acceptor | E(2) (kcal mol$^{-1}$) |
|---|---|
| 218. BD*(1) Si1-O5 | 3.9 |
| 219. BD*(1) Si1-C6 | 2.1 |
| 364. RY (6) Si1 | 1.1 |
| 228. BD*(1) Si4-C16 | 0.9 |
| 363. RY (5) Si1 | 0.9 |

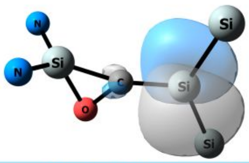


NBO 69. LP(1) Si2 occ. 0.97
sp99.99

| Acceptor | E(2) (kcal mol$^{-1}$) |
|---|---|
| 229. BD*(1) Si4-C20 | 15.0 |
| 226. BD*(1) Si3-C29 | 11.6 |
| 225. BD*(1) Si3-C25 | 10.3 |
| 228. BD*(1) Si4-C16 | 4.7 |
| 230. BD*(1) Si4-C21 | 2.6 |

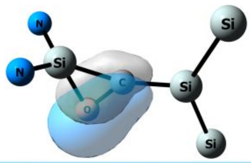


NBO 91. BD(2) O5-C6 occ. 1.94
84.36% O5 sp1.00
15.64% C6 sp99.99

| Acceptor | E(2) (kcal mol$^{-1}$) |
|---|---|
| 221. BD*(1) Si1-N8 | 7.8 |
| 220. BD*(1) Si1-N7 | 7.6 |
| 361. RY (3) Si1 | 3.9 |
| 232. BD*(2) O5-C6 | 0.8 |
| 360. RY (2) Si1 | 0.6 |

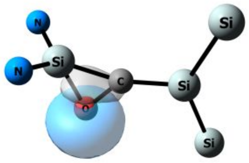


NBO 70. LP(1) O5 occ. 1.98
sp0.97

| Acceptor | E(2) (kcal mol$^{-1}$) |
|---|---|
| 219. BD*(1) Si1-C6 | 2.3 |
| 499. RY (3) C6 | 2.0 |
| 363. RY (5) Si1 | 1.5 |
| 359. RY (1) Si1 | 1.4 |
| 364. RY (6) Si1 | 0.7 |

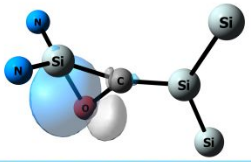


NBO 77. BD(1) Si1-O5 occ. 1.97
13.65% Si1 sp4.81d0.14
86.35% O5 sp2.97

| Acceptor | E(2) (kcal mol$^{-1}$) |
|---|---|
| 224. BD*(1) Si2-C6 | 9.1 |
| 498. RY (2) C6 | 3.9 |
| 231. BD*(1) O5-C6 | 1.8 |
| 368. RY (10) Si1 | 0.7 |
| 550. RY (2) N8 | 0.5 |

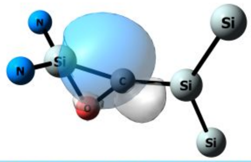


NBO 78. BD(1) Si1-C6 occ. 1.96
25.57% Si1 sp2.76
74.43% C6 sp2.30

| Acceptor | E(2) (kcal mol$^{-1}$) |
|---|---|
| 222. BD*(1) Si2-Si3 | 2.4 |
| 224. BD*(1) Si2-C6 | 2.3 |
| 472. RY (2) O5 | 2.2 |
| 387. RY (1) Si2 | 1.6 |
| 236. BD*(2) N8-C52 | 1.3 |

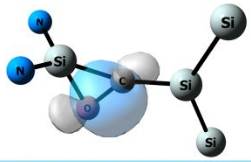


NBO 90. BD(1) O5-C6 occ. 1.98
67.25% O5 sp3.10
32.75% C6 sp4.28

| Acceptor | E(2) (kcal mol$^{-1}$) |
|---|---|
| 218. BD*(1) Si1-O5 | 2.7 |
| 220. BD*(1) Si1-N7 | 1.7 |
| 221. BD*(1) Si1-N8 | 1.5 |
| 219. BD*(1) Si1-C6 | 1.3 |
| 224. BD*(1) Si2-C6 | 1.1 |

**Figure S55** Selected NBO and the donor acceptor interactions in **2a**.

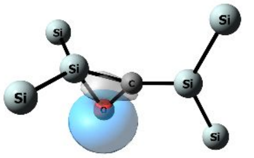

| NBO 69. LP(1) O7 occ. 1.97 sp0.85 | |
|---|---|
| Acceptor | E(2) (kcal mol-1) |
| 213. BD*(1) Si4-C8 | 4.5 |
| 414. RY (2) Si4 | 2.6 |
| 525. RY (3) C8 | 2.1 |
| 212. BD*(1) Si4-O7 | 0.8 |
| 523. RY (1) C8 | 0.6 |

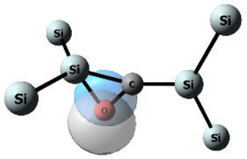

| NBO 70. LP(2) O7 occ. 1.80 sp1.00 | |
|---|---|
| Acceptor | E(2) (kcal mol-1) |
| 203. BD*(2) Si1-C8 | 33.4 |
| 524. RY (2) C8 | 3.3 |
| 413. RY (1) Si4 | 2.3 |
| 211. BD*(1) Si4-Si6 | 1.8 |
| 210. BD*(1) Si4-Si5 | 1.5 |

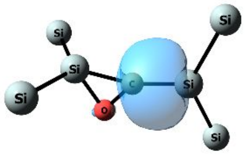

| NBO 73. BD(1) Si1-C8 occ. 1.97 26.28% Si1 sp2.52 73.72% C8 sp0.92 | |
|---|---|
| Acceptor | E(2) (kcal $mol^{-1}$) |
| 212. BD*(1) Si4-O7 | 6.8 |
| 213. BD*(1) Si4-C8 | 2.8 |
| 209. BD*(1) Si3-C26 | 0.6 |
| 414. RY (2) Si4 | 0.5 |

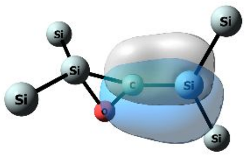

| NBO 74. BD(2) Si1-C8 occ. 1.91 44.75% Si1 sp99.99 55.25% C8 sp99.99 | |
|---|---|
| Acceptor | E(2) (kcal $mol^{-1}$) |
| 203. BD*(2) Si1-C8 | 4.4 |
| 204. BD*(1) Si2-C12 | 3.5 |
| 205. BD*(1) Si2-C15 | 2.8 |
| 208. BD*(1) Si3-C25 | 2.7 |
| 207. BD*(1) Si3-C21 | 2.4 |

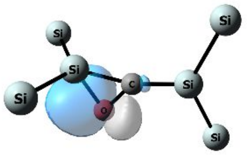

| NBO 83. BD(1) Si4-O7 occ. 1.96 15.22% Si4 sp10.83 84.78% O7 sp4.44 | |
|---|---|
| Acceptor | E(2) (kcal $mol^{-1}$) |
| 202. BD*(1) Si1-C8 | 10.1 |
| 523. RY (1) C8 | 3.9 |
| 220. BD*(1) O7-C8 | 2.0 |
| 213. BD*(1) Si4-C8 | 1.6 |
| 214. BD*(1) Si5-C37 | 1.0 |

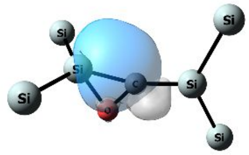

| NBO 84. BD(1) Si4-C8 occ. 1.96 30.18% Si4 sp3.97 69.82% C8 sp2.76 | |
|---|---|
| Acceptor | E(2) (kcal $mol^{-1}$) |
| 498. RY (2) O7 | 2.0 |
| 201. BD*(1) Si1-Si3 | 1.9 |
| 202. BD*(1) Si1-C8 | 1.8 |
| 329. RY (1) Si1 | 1.6 |
| 215. BD*(1) Si5-C41 | 1.2 |

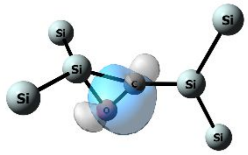

| NBO 91. BD(1) O7-C8 occ. 1.99 67.39% O7 sp2.61 32.61% C8 sp3.78 | |
|---|---|
| Acceptor | E(2) (kcal $mol^{-1}$) |
| 212. BD*(1) Si4-O7 | 2.3 |
| 202. BD*(1) Si1-C8 | 1.3 |
| 213. BD*(1) Si4-C8 | 0.5 |

**Figure S56** Selected NBO and the donor acceptor interactions in **2b**.

### Cartesian coordinates and energies of the optimized geometries at the r²SCAN-3c level

Calculated energies and coordinates of **1a**

Electronic energy ... -3059.63397744 Eh
Total Enthalpy ... -3058.39917425 Eh
Final Gibbs free energy ... -3058.55033292 Eh

CARTESIAN COORDINATES (ANGSTROEM)

| | | | |
|---|---|---|---|
| Si | 10.914082 | 9.075383 | 5.081379 |
| Si | 10.660059 | 6.861447 | 5.125540 |
| Si | 10.212064 | 5.979698 | 2.965678 |
| Si | 11.685860 | 6.041692 | 7.099506 |
| N | 10.919483 | 10.052866 | 6.425160 |
| N | 9.432769 | 10.532102 | 8.283698 |
| N | 11.568388 | 11.117077 | 8.454581 |
| N | 10.872430 | 10.057006 | 3.740973 |
| N | 10.252571 | 11.183946 | 1.738057 |
| N | 12.390386 | 10.623693 | 1.934083 |
| C | 10.651730 | 10.497361 | 7.595958 |
| C | 9.627247 | 11.134522 | 9.522249 |
| C | 10.919609 | 11.496477 | 9.622476 |
| C | 8.098574 | 10.149434 | 7.734219 |
| C | 8.016963 | 8.632867 | 7.555873 |
| C | 7.005292 | 10.586884 | 8.715357 |
| C | 7.864633 | 10.879686 | 6.406063 |
| C | 12.906653 | 11.616732 | 8.030615 |
| C | 13.579014 | 12.326530 | 9.209076 |
| C | 12.710421 | 12.626418 | 6.890532 |
| C | 13.803595 | 10.451026 | 7.601478 |
| C | 11.158662 | 10.546561 | 2.592725 |
| C | 10.922277 | 11.626007 | 0.604560 |
| C | 12.218434 | 11.280705 | 0.720359 |
| C | 8.883318 | 11.611937 | 2.140521 |
| C | 8.025346 | 10.390205 | 2.486129 |
| C | 8.219204 | 12.345285 | 0.971921 |
| C | 9.002195 | 12.576403 | 3.329076 |
| C | 13.705539 | 10.210225 | 2.498000 |
| C | 14.825093 | 10.628804 | 1.539874 |
| C | 13.743655 | 8.689094 | 2.654845 |
| C | 13.932526 | 10.920043 | 3.838004 |
| C | 10.307642 | 7.365830 | 1.674338 |
| C | 8.333613 | 5.444702 | 2.982514 |
| C | 7.492878 | 6.728732 | 3.094058 |
| C | 7.919887 | 4.712222 | 1.696390 |
| C | 7.982487 | 4.555177 | 4.180841 |
| C | 11.417523 | 4.594806 | 2.322357 |
| C | 11.237621 | 4.346589 | 0.813481 |
| C | 11.231407 | 3.268396 | 3.068902 |
| C | 12.860917 | 5.075161 | 2.535105 |
| C | 11.571879 | 7.418091 | 8.399105 |
| C | 10.647996 | 4.568743 | 7.832575 |

| | | | |
|---|---|---|---|
| C | 9.269299 | 5.132087 | 8.220464 |
| C | 10.436226 | 3.443936 | 6.812999 |
| C | 11.287799 | 3.971160 | 9.096135 |
| C | 13.589113 | 5.639219 | 6.953555 |
| C | 14.262189 | 6.697684 | 6.066825 |
| C | 14.264808 | 5.700700 | 8.336297 |
| C | 13.845710 | 4.253403 | 6.345303 |
| H | 8.846488 | 11.230908 | 10.255375 |
| H | 11.426556 | 11.950694 | 10.455469 |
| H | 7.034584 | 8.365043 | 7.151513 |
| H | 8.138852 | 8.126400 | 8.518992 |
| H | 8.773085 | 8.255408 | 6.859563 |
| H | 6.038650 | 10.329537 | 8.274107 |
| H | 7.017525 | 11.667777 | 8.891147 |
| H | 7.076172 | 10.062322 | 9.673682 |
| H | 6.861172 | 10.644425 | 6.037064 |
| H | 8.586310 | 10.577545 | 5.647583 |
| H | 7.940744 | 11.963825 | 6.544668 |
| H | 14.574139 | 12.646994 | 8.888425 |
| H | 13.704423 | 11.658259 | 10.067712 |
| H | 13.028232 | 13.219024 | 9.521967 |
| H | 13.683588 | 12.995569 | 6.550004 |
| H | 12.120154 | 13.478991 | 7.244099 |
| H | 12.194008 | 12.168699 | 6.044250 |
| H | 14.769306 | 10.842215 | 7.264645 |
| H | 13.353339 | 9.886724 | 6.785337 |
| H | 13.981985 | 9.774201 | 8.443715 |
| H | 10.427613 | 12.110761 | -0.218555 |
| H | 13.016541 | 11.423483 | 0.013771 |
| H | 7.925278 | 9.739522 | 1.611603 |
| H | 7.025093 | 10.721363 | 2.784363 |
| H | 8.457941 | 9.812403 | 3.302792 |
| H | 7.204473 | 12.616809 | 1.275727 |
| H | 8.141678 | 11.708510 | 0.084070 |
| H | 8.744749 | 13.269086 | 0.710362 |
| H | 8.003728 | 12.879179 | 3.661759 |
| H | 9.555450 | 13.474077 | 3.031335 |
| H | 9.521112 | 12.105647 | 4.166455 |
| H | 15.776754 | 10.324708 | 1.984326 |
| H | 14.856041 | 11.713275 | 1.391083 |
| H | 14.743374 | 10.131329 | 0.568035 |
| H | 14.689859 | 8.387575 | 3.116777 |
| H | 13.674907 | 8.211191 | 1.672292 |
| H | 12.929254 | 8.317714 | 3.284409 |
| H | 14.911658 | 10.637451 | 4.237779 |
| H | 13.172883 | 10.646666 | 4.569575 |
| H | 13.907970 | 12.007153 | 3.704554 |
| H | 9.798642 | 7.055828 | 0.753310 |
| H | 11.342091 | 7.614283 | 1.420209 |
| H | 9.830902 | 8.278232 | 2.034315 |
| H | 6.429431 | 6.467347 | 3.199225 |
| H | 7.592874 | 7.358848 | 2.204363 |
| H | 7.785998 | 7.321682 | 3.970110 |
| H | 6.829155 | 4.567337 | 1.689419 |
| H | 8.376695 | 3.719666 | 1.627101 |
| H | 8.179623 | 5.273025 | 0.791106 |
| H | 6.908163 | 4.317361 | 4.170378 |
| H | 8.203241 | 5.065897 | 5.124890 |
| H | 8.528610 | 3.606136 | 4.169271 |
| H | 11.980967 | 3.609194 | 0.475430 |
| H | 11.394564 | 5.259391 | 0.228819 |
| H | 10.249957 | 3.951119 | 0.563565 |
| H | 11.977575 | 2.534519 | 2.729112 |
| H | 10.241620 | 2.832896 | 2.894351 |
| H | 11.359268 | 3.390884 | 4.148852 |
| H | 13.568623 | 4.296954 | 2.211933 |
| H | 13.059846 | 5.303760 | 3.585407 |
| H | 13.076418 | 5.978059 | 1.954444 |
| H | 11.815860 | 7.019387 | 9.391158 |
| H | 10.570809 | 7.851517 | 8.443838 |
| H | 12.269998 | 8.229185 | 8.181938 |
| H | 8.610216 | 4.315286 | 8.549756 |
| H | 8.789769 | 5.638577 | 7.374376 |
| H | 9.344463 | 5.850718 | 9.044156 |
| H | 9.769721 | 2.673465 | 7.229328 |
| H | 11.373883 | 2.952229 | 6.536752 |
| H | 9.968976 | 3.823033 | 5.900786 |
| H | 10.599691 | 3.239831 | 9.545991 |
| H | 11.496952 | 4.731754 | 9.857034 |
| H | 12.220951 | 3.442285 | 8.876623 |
| H | 15.346487 | 6.513231 | 6.022664 |
| H | 14.110694 | 7.710166 | 6.460060 |
| H | 13.862861 | 6.687233 | 5.049342 |
| H | 15.332232 | 5.453323 | 8.235019 |
| H | 13.832163 | 4.994632 | 9.051141 |
| H | 14.201768 | 6.703100 | 8.773289 |
| H | 14.920600 | 4.117989 | 6.153447 |
| H | 13.322821 | 4.117134 | 5.392921 |
| H | 13.531586 | 3.449193 | 7.018731 |

---

Calculated energies and coordinates of **TS1a**

Electronic energy ... -3172.91957862 Eh
Total Enthalpy ... -3171.67561161 Eh
Final Gibbs free energy ... -3171.83188506 Eh

CARTESIAN COORDINATES (ANGSTROEM)

| | | | |
|---|---|---|---|
| Si | 11.155390 | 9.055501 | 5.234121 |
| Si | 11.087732 | 6.813447 | 5.119782 |
| Si | 10.360999 | 6.057250 | 2.987691 |
| Si | 11.912160 | 5.718982 | 7.074236 |
| N | 10.836823 | 9.882610 | 6.641036 |
| N | 9.028630 | 10.888786 | 7.890020 |
| N | 10.991297 | 11.907387 | 7.941579 |
| N | 11.361371 | 10.055262 | 3.918546 |
| N | 10.382146 | 11.411630 | 2.190166 |
| N | 12.425228 | 10.637575 | 1.824397 |
| C | 10.335622 | 10.799011 | 7.391772 |
| C | 8.912830 | 12.015802 | 8.690983 |
| C | 10.109168 | 12.633249 | 8.726727 |
| C | 7.951557 | 9.874467 | 7.707002 |
| C | 8.452555 | 8.509787 | 8.178756 |
| C | 6.730107 | 10.270977 | 8.541121 |
| C | 7.546175 | 9.817739 | 6.234845 |
| C | 12.441954 | 12.199209 | 7.800551 |
| C | 12.792005 | 13.471798 | 8.576799 |
| C | 12.762635 | 12.431471 | 6.320973 |
| C | 13.233973 | 11.030218 | 8.398350 |
| C | 11.386070 | 10.626345 | 2.769162 |
| C | 10.793025 | 11.850184 | 0.940043 |
| C | 12.041228 | 11.394800 | 0.726697 |
| C | 9.078466 | 11.753265 | 2.818865 |
| C | 8.228490 | 10.484453 | 2.895933 |
| C | 8.345634 | 12.781863 | 1.951226 |
| C | 9.319703 | 12.380763 | 4.198606 |
| C | 13.834245 | 10.223957 | 2.081392 |
| C | 14.667875 | 10.414987 | 0.809443 |
| C | 13.899679 | 8.741353 | 2.458289 |
| C | 14.397416 | 11.140872 | 3.172637 |
| C | 10.497100 | 7.473051 | 1.730263 |
| C | 8.445897 | 5.648381 | 3.033399 |

| | | | |
|---|---|---|---|
| C | 7.696249 | 6.977274 | 3.216640 |
| C | 7.964188 | 5.007962 | 1.721349 |
| C | 8.040337 | 4.734896 | 4.194272 |
| C | 11.430724 | 4.605626 | 2.257529 |
| C | 11.231667 | 4.463138 | 0.737335 |
| C | 11.107671 | 3.263908 | 2.928653 |
| C | 12.913862 | 4.929682 | 2.491409 |
| C | 12.165933 | 7.097723 | 8.357009 |
| C | 10.621340 | 4.461964 | 7.813252 |
| C | 9.369333 | 5.261842 | 8.200525 |
| C | 10.209405 | 3.398222 | 6.787760 |
| C | 11.134278 | 3.748318 | 9.074602 |
| C | 13.673489 | 4.890007 | 6.874011 |
| C | 14.591782 | 5.826781 | 6.077808 |
| C | 14.340449 | 4.692891 | 8.250738 |
| C | 13.617378 | 3.527636 | 6.169574 |
| H | 7.994708 | 12.304970 | 9.170126 |
| H | 10.385458 | 13.536635 | 9.240880 |
| H | 7.631295 | 7.787539 | 8.137137 |
| H | 8.812350 | 8.565295 | 9.212061 |
| H | 9.254989 | 8.143781 | 7.536003 |
| H | 5.962286 | 9.507284 | 8.391564 |
| H | 6.305897 | 11.230416 | 8.226223 |
| H | 6.958226 | 10.308110 | 9.611376 |
| H | 6.748084 | 9.080860 | 6.096940 |
| H | 8.391294 | 9.512370 | 5.616753 |
| H | 7.186210 | 10.794249 | 5.895842 |
| H | 13.860674 | 13.661452 | 8.442256 |
| H | 12.607572 | 13.364930 | 9.650655 |
| H | 12.250142 | 14.345644 | 8.199819 |
| H | 13.839999 | 12.582691 | 6.194873 |
| H | 12.242652 | 13.326065 | 5.960315 |
| H | 12.451405 | 11.583432 | 5.710996 |
| H | 14.307330 | 11.195020 | 8.257693 |
| H | 12.956833 | 10.089773 | 7.921283 |
| H | 13.031659 | 10.949989 | 9.472054 |
| H | 10.169380 | 12.433665 | 0.286514 |
| H | 12.664491 | 11.528341 | -0.139441 |
| H | 8.045526 | 10.095254 | 1.888871 |
| H | 7.263375 | 10.696812 | 3.366558 |
| H | 8.730322 | 9.709558 | 3.476008 |
| H | 7.410050 | 13.039558 | 2.455119 |
| H | 8.086915 | 12.382943 | 0.965266 |
| H | 8.925870 | 13.702499 | 1.829488 |
| H | 8.359933 | 12.673556 | 4.635931 |
| H | 9.939570 | 13.278404 | 4.098450 |
| H | 9.817733 | 11.689938 | 4.878274 |
| H | 15.673433 | 10.035066 | 1.010958 |
| H | 14.763871 | 11.467533 | 0.524769 |
| H | 14.260380 | 9.845064 | -0.032231 |
| H | 14.916190 | 8.491099 | 2.777492 |
| H | 13.663669 | 8.130639 | 1.581764 |
| H | 13.208597 | 8.470121 | 3.259854 |
| H | 15.430325 | 10.864188 | 3.406479 |
| H | 13.803761 | 11.067812 | 4.084004 |
| H | 14.379768 | 12.181949 | 2.831096 |
| H | 9.926435 | 7.229424 | 0.825952 |
| H | 11.534077 | 7.648194 | 1.434942 |
| H | 10.109343 | 8.411152 | 2.130923 |
| H | 6.618565 | 6.784794 | 3.328177 |
| H | 7.826427 | 7.641213 | 2.356558 |
| H | 8.041926 | 7.501250 | 4.116203 |
| H | 6.865992 | 4.938491 | 1.728479 |
| H | 8.350046 | 3.991433 | 1.595086 |
| H | 8.247342 | 5.593489 | 0.839232 |
| H | 6.950900 | 4.579131 | 4.183492 |
| H | 8.303570 | 5.187479 | 5.156118 |
| H | 8.511463 | 3.748760 | 4.136807 |
| H | 11.880783 | 3.659587 | 0.358381 |
| H | 11.504641 | 5.381177 | 0.205788 |
| H | 10.204466 | 4.208643 | 0.464543 |
| H | 11.808303 | 2.488215 | 2.584937 |
| H | 10.095883 | 2.920307 | 2.688693 |
| H | 11.190072 | 3.321178 | 4.019113 |
| H | 13.544419 | 4.128724 | 2.076865 |
| H | 13.143660 | 5.032800 | 3.554340 |
| H | 13.204584 | 5.867557 | 2.005005 |
| H | 12.256146 | 6.677635 | 9.366011 |
| H | 11.348856 | 7.822910 | 8.353165 |
| H | 13.089254 | 7.648449 | 8.144929 |
| H | 8.593489 | 4.582130 | 8.583674 |
| H | 8.960707 | 5.797601 | 7.337498 |
| H | 9.585020 | 5.997629 | 8.983456 |
| H | 9.361540 | 2.810226 | 7.170637 |
| H | 11.019594 | 2.699080 | 6.564301 |
| H | 9.892021 | 3.857526 | 5.849956 |
| H | 10.313002 | 3.173396 | 9.528098 |
| H | 11.501110 | 4.447953 | 9.834560 |
| H | 11.934938 | 3.038372 | 8.846935 |
| H | 15.554482 | 5.330894 | 5.883576 |
| H | 14.805822 | 6.732480 | 6.649671 |
| H | 14.157068 | 6.133935 | 5.120983 |
| H | 15.348582 | 4.276105 | 8.106376 |
| H | 13.792837 | 4.003238 | 8.897062 |
| H | 14.454456 | 5.640485 | 8.787432 |
| H | 14.638249 | 3.168196 | 5.972519 |
| H | 13.096963 | 3.574843 | 5.208082 |
| H | 13.121614 | 2.770609 | 6.785019 |
| C | 13.922459 | 9.248382 | 5.746641 |
| O | 15.016247 | 8.954799 | 5.627587 |

---

Calculated energies and coordinates of **INT1a**

Electronic energy ... -3172.92397505 Eh
Total Enthalpy ... -3171.67889702 Eh
Final Gibbs free energy ... -3171.83506355 Eh

CARTESIAN COORDINATES (ANGSTROEM)

| | | | |
|---|---|---|---|
| Si | 11.298212 | 9.101509 | 5.278155 |
| Si | 11.081565 | 6.836867 | 5.125171 |
| Si | 10.339285 | 6.050424 | 3.003204 |
| Si | 12.005269 | 5.733231 | 7.031883 |
| N | 10.688000 | 9.902992 | 6.606654 |
| N | 8.932215 | 10.871965 | 7.923678 |
| N | 10.895556 | 11.890769 | 7.977794 |
| N | 11.365646 | 10.028671 | 3.889207 |
| N | 10.481184 | 11.429732 | 2.158769 |
| N | 12.515261 | 10.608986 | 1.834010 |
| C | 10.229686 | 10.798480 | 7.402471 |
| C | 8.826680 | 11.969614 | 8.762927 |
| C | 10.023897 | 12.586589 | 8.802040 |
| C | 7.844846 | 9.885818 | 7.668629 |
| C | 8.323898 | 8.487155 | 8.062246 |
| C | 6.620264 | 10.248420 | 8.512569 |
| C | 7.461116 | 9.933930 | 6.188970 |
| C | 12.343429 | 12.192036 | 7.826316 |
| C | 12.698960 | 13.445187 | 8.631571 |
| C | 12.650850 | 12.467239 | 6.351007 |
| C | 13.151076 | 11.012701 | 8.380615 |
| C | 11.452302 | 10.614172 | 2.753699 |
| C | 10.937691 | 11.878382 | 0.927663 |

| | | | |
|---|---|---|---|
| C | 12.178015 | 11.392886 | 0.737746 |
| C | 9.168075 | 11.782905 | 2.759328 |
| C | 8.310878 | 10.519014 | 2.840953 |
| C | 8.455181 | 12.803738 | 1.866751 |
| C | 9.386794 | 12.418996 | 4.138594 |
| C | 13.903795 | 10.157500 | 2.127570 |
| C | 14.790803 | 10.375552 | 0.896732 |
| C | 13.921897 | 8.658236 | 2.446337 |
| C | 14.449186 | 11.022029 | 3.271483 |
| C | 10.477330 | 7.447518 | 1.725654 |
| C | 8.419708 | 5.682675 | 3.108798 |
| C | 7.707209 | 7.030815 | 3.302558 |
| C | 7.884943 | 5.037789 | 1.820258 |
| C | 8.033048 | 4.790131 | 4.292395 |
| C | 11.366299 | 4.571837 | 2.270845 |
| C | 11.107502 | 4.391760 | 0.763655 |
| C | 11.058689 | 3.251088 | 2.988148 |
| C | 12.858786 | 4.892349 | 2.441144 |
| C | 12.270421 | 7.125197 | 8.302632 |
| C | 10.736945 | 4.487736 | 7.829732 |
| C | 9.504611 | 5.299240 | 8.255449 |
| C | 10.278759 | 3.416729 | 6.831931 |
| C | 11.296287 | 3.784315 | 9.076578 |
| C | 13.763116 | 4.909340 | 6.789189 |
| C | 14.649952 | 5.821622 | 5.928424 |
| C | 14.479138 | 4.749227 | 8.145287 |
| C | 13.682686 | 3.528262 | 6.123891 |
| H | 7.916128 | 12.239340 | 9.267636 |
| H | 10.307040 | 13.470631 | 9.344955 |
| H | 7.497189 | 7.777157 | 7.957084 |
| H | 8.657558 | 8.474726 | 9.105533 |
| H | 9.142419 | 8.155177 | 7.420714 |
| H | 5.842988 | 9.506595 | 8.310588 |
| H | 6.214116 | 11.231261 | 8.251020 |
| H | 6.838259 | 10.218934 | 9.585217 |
| H | 6.681133 | 9.194340 | 5.980585 |
| H | 8.324855 | 9.704148 | 5.565631 |
| H | 7.083178 | 10.927072 | 5.923217 |
| H | 13.765616 | 13.641912 | 8.490965 |
| H | 12.526684 | 13.310109 | 9.704231 |
| H | 12.149964 | 14.326501 | 8.283548 |
| H | 13.724289 | 12.641677 | 6.223343 |
| H | 12.110868 | 13.358822 | 6.014468 |
| H | 12.355292 | 11.630093 | 5.717713 |
| H | 14.220052 | 11.175664 | 8.210684 |
| H | 12.860663 | 10.078700 | 7.899709 |
| H | 12.978281 | 10.910187 | 9.457373 |
| H | 10.346217 | 12.487777 | 0.267904 |
| H | 12.825533 | 11.520956 | -0.111391 |
| H | 8.147984 | 10.109751 | 1.838306 |
| H | 7.337387 | 10.747124 | 3.286707 |
| H | 8.798548 | 9.757769 | 3.450312 |
| H | 7.506123 | 13.062191 | 2.344135 |
| H | 8.224807 | 12.396691 | 0.876995 |
| H | 9.035483 | 13.725538 | 1.753896 |
| H | 8.420039 | 12.711384 | 4.560773 |
| H | 10.007336 | 13.316708 | 4.043343 |
| H | 9.872465 | 11.727532 | 4.827419 |
| H | 15.781178 | 9.971765 | 1.125610 |
| H | 14.913740 | 11.435278 | 0.651767 |
| H | 14.408560 | 9.840790 | 0.020987 |
| H | 14.907947 | 8.373319 | 2.826671 |
| H | 13.733276 | 8.087203 | 1.531878 |
| H | 13.171774 | 8.366457 | 3.184457 |
| H | 15.452597 | 10.690006 | 3.555341 |
| H | 13.806381 | 10.964796 | 4.148887 |
| H | 14.500307 | 12.069125 | 2.952699 |
| H | 9.856960 | 7.221319 | 0.849912 |
| H | 11.506352 | 7.580318 | 1.382130 |
| H | 10.147929 | 8.402582 | 2.137537 |
| H | 6.629629 | 6.865883 | 3.452888 |
| H | 7.823731 | 7.683609 | 2.432161 |
| H | 8.095305 | 7.555345 | 4.184590 |
| H | 6.786297 | 4.991233 | 1.861085 |
| H | 8.246654 | 4.012101 | 1.695750 |
| H | 8.153784 | 5.606294 | 0.922620 |
| H | 6.941020 | 4.655876 | 4.317075 |
| H | 8.334428 | 5.248001 | 5.240697 |
| H | 8.483349 | 3.794355 | 4.233385 |
| H | 11.747766 | 3.584348 | 0.377937 |
| H | 11.348372 | 5.298060 | 0.197558 |
| H | 10.072459 | 4.120901 | 0.541279 |
| H | 11.739288 | 2.460324 | 2.638773 |
| H | 10.035527 | 2.911125 | 2.795479 |
| H | 11.183277 | 3.337302 | 4.072311 |
| H | 13.469537 | 4.075700 | 2.027721 |
| H | 13.127812 | 5.023684 | 3.491912 |
| H | 13.136637 | 5.813120 | 1.915740 |
| H | 12.348913 | 6.715042 | 9.316683 |
| H | 11.450861 | 7.849890 | 8.286737 |
| H | 13.200783 | 7.667598 | 8.101418 |
| H | 8.744143 | 4.630461 | 8.685672 |
| H | 9.060142 | 5.817780 | 7.400018 |
| H | 9.754304 | 6.051090 | 9.012265 |
| H | 9.443018 | 2.838471 | 7.253957 |
| H | 11.074667 | 2.709644 | 6.582431 |
| H | 9.927638 | 3.869285 | 5.902911 |
| H | 10.494517 | 3.206757 | 9.560568 |
| H | 11.683619 | 4.490276 | 9.820157 |
| H | 12.093985 | 3.079307 | 8.825150 |
| H | 15.583493 | 5.299068 | 5.672194 |
| H | 14.925826 | 6.727662 | 6.472669 |
| H | 14.158693 | 6.130190 | 4.999141 |
| H | 15.485258 | 4.336952 | 7.976147 |
| H | 13.959644 | 4.069981 | 8.825171 |
| H | 14.603352 | 5.709326 | 8.656699 |
| H | 14.697311 | 3.155760 | 5.918980 |
| H | 13.146570 | 3.554681 | 5.170228 |
| H | 13.191973 | 2.790594 | 6.766244 |
| C | 13.217858 | 9.067729 | 5.641588 |
| O | 14.358967 | 8.921093 | 5.747964 |

---

Calculated energies and coordinates of **TS2a**

Electronic energy ... -3172.92388877 Eh
Total Enthalpy ... -3171.67973924 Eh
Final Gibbs free energy ... -3171.83419716 Eh

CARTESIAN COORDINATES (ANGSTROEM)

| | | | |
|---|---|---|---|
| Si | 0.287770 | 0.910703 | 0.205951 |
| Si | 0.290411 | -1.363985 | 0.001657 |
| Si | -0.648513 | -2.165783 | -2.032253 |
| Si | 1.102840 | -2.467379 | 1.960365 |
| N | -0.404542 | 1.715015 | 1.488995 |
| N | -2.125688 | 2.631008 | 2.876585 |
| N | -0.159299 | 3.645302 | 2.938841 |
| N | 0.381196 | 1.825707 | -1.189919 |
| N | -0.466520 | 3.246072 | -2.922506 |
| N | 1.571515 | 2.424630 | -3.218979 |
| C | -0.837164 | 2.577670 | 2.333096 |
| C | -2.215527 | 3.692668 | 3.762774 |

| | | | |
|---|---|---|---|
| C | -1.016098 | 4.305108 | 3.808405 |
| C | -3.227294 | 1.679021 | 2.564183 |
| C | -2.766570 | 0.249690 | 2.859315 |
| C | -4.441895 | 2.000063 | 3.438478 |
| C | -3.616444 | 1.832402 | 1.092326 |
| C | 1.287689 | 3.949953 | 2.779228 |
| C | 1.649634 | 5.184427 | 3.609928 |
| C | 1.586029 | 4.258533 | 1.308498 |
| C | 2.100051 | 2.757173 | 3.294237 |
| C | 0.493023 | 2.422580 | -2.317542 |
| C | 0.013186 | 3.706854 | -4.140123 |
| C | 1.255095 | 3.219742 | -4.313036 |
| C | -1.785795 | 3.604543 | -2.339407 |
| C | -2.647864 | 2.343809 | -2.262331 |
| C | -2.486173 | 4.623428 | -3.244036 |
| C | -1.582323 | 4.244406 | -0.959747 |
| C | 2.952668 | 1.957740 | -2.913110 |
| C | 3.861371 | 2.199347 | -4.123644 |
| C | 2.954053 | 0.451674 | -2.630488 |
| C | 3.485086 | 2.792340 | -1.741065 |
| C | -0.516181 | -0.785670 | -3.328973 |
| C | -2.570265 | -2.480860 | -1.853314 |
| C | -3.236628 | -1.112522 | -1.640894 |
| C | -3.165861 | -3.115097 | -3.120477 |
| C | -2.939915 | -3.357767 | -0.653433 |
| C | 0.325272 | -3.672298 | -2.779556 |
| C | 0.016841 | -3.854661 | -4.277260 |
| C | 0.014092 | -4.983650 | -2.046899 |
| C | 1.829161 | -3.382866 | -2.654120 |
| C | 1.365107 | -1.070139 | 3.225959 |
| C | -0.174378 | -3.695294 | 2.772447 |
| C | -1.395030 | -2.865033 | 3.195690 |
| C | -0.647140 | -4.774319 | 1.790620 |
| C | 0.383336 | -4.389992 | 4.025312 |
| C | 2.852722 | -3.306166 | 1.718244 |
| C | 3.747098 | -2.417412 | 0.840400 |
| C | 3.572227 | -3.455320 | 3.073635 |
| C | 2.752534 | -4.694445 | 1.070217 |
| H | -3.116645 | 3.940714 | 4.294860 |
| H | -0.721426 | 5.162931 | 4.386326 |
| H | -3.595557 | -0.442841 | 2.679404 |
| H | -2.458016 | 0.152876 | 3.905786 |
| H | -1.932361 | -0.036906 | 2.216296 |
| H | -5.228034 | 1.279198 | 3.198262 |
| H | -4.840436 | 3.000892 | 3.241624 |
| H | -4.215227 | 1.903047 | 4.505397 |
| H | -4.409721 | 1.121602 | 0.838637 |
| H | -2.757601 | 1.631357 | 0.452558 |
| H | -3.977074 | 2.847582 | 0.893415 |
| H | 2.712455 | 5.391404 | 3.456558 |
| H | 1.496920 | 5.021597 | 4.681572 |
| H | 1.089138 | 6.070426 | 3.293422 |
| H | 2.657025 | 4.448643 | 1.183425 |
| H | 1.035326 | 5.150222 | 0.990542 |
| H | 1.302227 | 3.431223 | 0.657498 |
| H | 3.168790 | 2.932785 | 3.135762 |
| H | 1.820137 | 1.841664 | 2.774304 |
| H | 1.922160 | 2.611686 | 4.365072 |
| H | -0.564674 | 4.324489 | -4.804159 |
| H | 1.917498 | 3.354495 | -5.149437 |
| H | -2.797559 | 1.927670 | -3.264259 |
| H | -3.626664 | 2.577780 | -1.831230 |
| H | -2.170550 | 1.585893 | -1.641035 |
| H | -3.442597 | 4.881398 | -2.781168 |
| H | -2.701123 | 4.214881 | -4.236726 |
| H | -1.905540 | 5.545841 | -3.349222 |
| H | -2.553226 | 4.542840 | -0.551542 |
| H | -0.956088 | 5.138607 | -1.050098 |
| H | -1.109928 | 3.552279 | -0.262240 |
| H | 4.846994 | 1.789754 | -3.884752 |
| H | 3.989887 | 3.263555 | -4.345503 |
| H | 3.495036 | 1.682290 | -5.016645 |
| H | 3.935888 | 0.147670 | -2.254015 |
| H | 2.760521 | -0.096050 | -3.558484 |
| H | 2.200886 | 0.150857 | -1.899088 |
| H | 4.478636 | 2.442189 | -1.444876 |
| H | 2.826293 | 2.726440 | -0.876470 |
| H | 3.555354 | 3.844660 | -2.038513 |
| H | -1.168860 | -1.001810 | -4.183505 |
| H | 0.506021 | -0.686037 | -3.703716 |
| H | -0.804642 | 0.182287 | -2.915825 |
| H | -4.312383 | -1.245731 | -1.450842 |
| H | -3.133473 | -0.467140 | -2.518198 |
| H | -2.803893 | -0.593931 | -0.775727 |
| H | -4.263300 | -3.134078 | -3.041701 |
| H | -2.833806 | -4.149743 | -3.254035 |
| H | -2.913750 | -2.555478 | -4.028392 |
| H | -4.033929 | -3.458062 | -0.587847 |
| H | -2.589723 | -2.907667 | 0.281451 |
| H | -2.523188 | -4.367104 | -0.725422 |
| H | 0.625622 | -4.679493 | -4.676516 |
| H | 0.261661 | -2.957848 | -4.856324 |
| H | -1.030873 | -4.101890 | -4.466334 |
| H | 0.655816 | -5.792736 | -2.426627 |
| H | -1.025727 | -5.296386 | -2.191047 |
| H | 0.192266 | -4.900748 | -0.970154 |
| H | 2.409122 | -4.208658 | -3.092865 |
| H | 2.133467 | -3.267420 | -1.610591 |
| H | 2.110828 | -2.462370 | -3.178471 |
| H | 1.448374 | -1.478174 | 4.240472 |
| H | 0.536197 | -0.355112 | 3.212048 |
| H | 2.289823 | -0.519718 | 3.023477 |
| H | -2.168492 | -3.521720 | 3.621176 |
| H | -1.829337 | -2.336942 | 2.341335 |
| H | -1.135318 | -2.119917 | 3.955551 |
| H | -1.485288 | -5.340107 | 2.224678 |
| H | 0.141709 | -5.491146 | 1.546557 |
| H | -0.998666 | -4.330930 | 0.857676 |
| H | -0.422959 | -4.952409 | 4.519587 |
| H | 0.782266 | -3.680109 | 4.758906 |
| H | 1.171608 | -5.107183 | 3.778485 |
| H | 4.682914 | -2.947888 | 0.610000 |
| H | 4.016687 | -1.494501 | 1.358990 |
| H | 3.265121 | -2.136237 | -0.102493 |
| H | 4.571489 | -3.884979 | 2.907418 |
| H | 3.045666 | -4.115306 | 3.767103 |
| H | 3.712456 | -2.488387 | 3.567951 |
| H | 3.761371 | -5.078983 | 0.859211 |
| H | 2.206709 | -4.673507 | 0.121359 |
| H | 2.261153 | -5.420027 | 1.725725 |
| C | 2.175569 | 0.745848 | 0.549676 |
| O | 3.325661 | 0.680891 | 0.684272 |

---

Calculated energies and coordinates of **INT2a**

Electronic energy ... -3172.92619953 Eh
Total Enthalpy ... -3171.68090902 Eh
Final Gibbs free energy ... -3171.83725273 Eh

CARTESIAN COORDINATES (ANGSTROEM)

| | | | |
|---|---|---|---|
| Si | 0.233474 | 0.958127 | 0.188771 |

| | | | |
|---|---|---|---|
| Si | 0.501176 | -1.362558 | -0.084596 |
| Si | -0.537758 | -2.300080 | -2.064161 |
| Si | 1.173435 | -2.487211 | 1.971907 |
| N | -0.547322 | 1.774882 | 1.413769 |
| N | -2.102276 | 2.695550 | 2.972813 |
| N | -0.067323 | 3.570071 | 2.979849 |
| N | 0.202334 | 1.771708 | -1.274373 |
| N | -0.574958 | 3.327794 | -2.919469 |
| N | 1.518749 | 2.630452 | -3.133817 |
| C | -0.851253 | 2.594326 | 2.350872 |
| C | -2.066450 | 3.696766 | 3.931545 |
| C | -0.829311 | 4.230409 | 3.935043 |
| C | -3.274874 | 1.829895 | 2.673173 |
| C | -2.890077 | 0.368116 | 2.908833 |
| C | -4.432786 | 2.198345 | 3.603516 |
| C | -3.714384 | 2.047155 | 1.221879 |
| C | 1.339724 | 3.905032 | 2.631208 |
| C | 1.843139 | 5.019034 | 3.554350 |
| C | 1.389505 | 4.413810 | 1.185459 |
| C | 2.226298 | 2.673265 | 2.835157 |
| C | 0.382433 | 2.495272 | -2.315654 |
| C | -0.029675 | 3.934851 | -4.040672 |
| C | 1.241416 | 3.512774 | -4.169370 |
| C | -1.921413 | 3.623356 | -2.366486 |
| C | -2.733582 | 2.327979 | -2.299678 |
| C | -2.650013 | 4.607187 | -3.287001 |
| C | -1.766023 | 4.270399 | -0.984484 |
| C | 2.884238 | 2.124476 | -2.819508 |
| C | 3.868684 | 2.586359 | -3.899875 |
| C | 2.880589 | 0.592476 | -2.817679 |
| C | 3.332949 | 2.727821 | -1.483763 |
| C | -0.393565 | -0.936485 | -3.375571 |
| C | -2.460344 | -2.555705 | -1.847483 |
| C | -3.084350 | -1.170450 | -1.611713 |
| C | -3.073233 | -3.151372 | -3.126871 |
| C | -2.859104 | -3.449179 | -0.669978 |
| C | 0.413247 | -3.827081 | -2.786859 |
| C | 0.131361 | -3.997099 | -4.291256 |
| C | 0.062978 | -5.135710 | -2.067803 |
| C | 1.917324 | -3.556906 | -2.621274 |
| C | 1.480617 | -1.072973 | 3.204820 |
| C | -0.229741 | -3.585530 | 2.751290 |
| C | -1.418796 | -2.651149 | 3.015650 |
| C | -0.687994 | -4.709941 | 1.813817 |
| C | 0.183278 | -4.210653 | 4.094343 |
| C | 2.856773 | -3.457057 | 1.802688 |
| C | 3.838613 | -2.707246 | 0.889887 |
| C | 3.527244 | -3.566380 | 3.186538 |
| C | 2.661932 | -4.874357 | 1.245735 |
| H | -2.911593 | 3.956273 | 4.544093 |
| H | -0.441235 | 5.021399 | 4.551710 |
| H | -3.748285 | -0.280108 | 2.702347 |
| H | -2.583292 | 0.215340 | 3.948985 |
| H | -2.067862 | 0.078763 | 2.253726 |
| H | -5.275608 | 1.542299 | 3.369182 |
| H | -4.765962 | 3.231412 | 3.458095 |
| H | -4.175714 | 2.045883 | 4.656870 |
| H | -4.573247 | 1.406165 | 0.996423 |
| H | -2.904932 | 1.797514 | 0.535805 |
| H | -4.005816 | 3.090562 | 1.061183 |
| H | 2.883074 | 5.229282 | 3.290090 |
| H | 1.822802 | 4.718267 | 4.607010 |
| H | 1.275326 | 5.947046 | 3.430885 |
| H | 2.422299 | 4.657240 | 0.916745 |
| H | 0.779906 | 5.318255 | 1.084145 |
| H | 1.019340 | 3.667814 | 0.479565 |
| H | 3.262403 | 2.917216 | 2.580418 |
| H | 1.913905 | 1.842570 | 2.204891 |
| H | 2.189819 | 2.347781 | 3.880346 |
| H | -0.585044 | 4.597080 | -4.680632 |
| H | 1.952787 | 3.754903 | -4.938549 |
| H | -2.860965 | 1.909973 | -3.304229 |
| H | -3.723737 | 2.528004 | -1.876859 |
| H | -2.228896 | 1.590100 | -1.677135 |
| H | -3.643254 | 4.788203 | -2.866893 |
| H | -2.785522 | 4.202000 | -4.295245 |
| H | -2.135718 | 5.571739 | -3.350038 |
| H | -2.751208 | 4.547772 | -0.596151 |
| H | -1.157742 | 5.178087 | -1.063147 |
| H | -1.293838 | 3.587022 | -0.276722 |
| H | 4.849796 | 2.171890 | -3.651369 |
| H | 3.967399 | 3.676141 | -3.934252 |
| H | 3.594751 | 2.213715 | -4.892401 |
| H | 3.842791 | 0.216236 | -2.456892 |
| H | 2.720823 | 0.226158 | -3.837623 |
| H | 2.098077 | 0.183094 | -2.178014 |
| H | 4.329231 | 2.359513 | -1.222160 |
| H | 2.657084 | 2.461769 | -0.673386 |
| H | 3.368039 | 3.819910 | -1.568746 |
| H | -1.075446 | -1.132764 | -4.211618 |
| H | 0.619805 | -0.880393 | -3.781335 |
| H | -0.629847 | 0.044187 | -2.955333 |
| H | -4.164106 | -1.274640 | -1.427310 |
| H | -2.958646 | -0.516110 | -2.478519 |
| H | -2.642157 | -0.671741 | -0.739452 |
| H | -4.170468 | -3.140236 | -3.045638 |
| H | -2.770418 | -4.193013 | -3.275946 |
| H | -2.806719 | -2.585713 | -4.026246 |
| H | -3.955113 | -3.537566 | -0.628505 |
| H | -2.528435 | -3.024042 | 0.281589 |
| H | -2.452019 | -4.461590 | -0.750499 |
| H | 0.722777 | -4.839295 | -4.679847 |
| H | 0.413560 | -3.107153 | -4.863173 |
| H | -0.920258 | -4.212469 | -4.500311 |
| H | 0.701962 | -5.952387 | -2.435052 |
| H | -0.977366 | -5.431477 | -2.241847 |
| H | 0.214485 | -5.061551 | -0.986122 |
| H | 2.500383 | -4.369934 | -3.078673 |
| H | 2.201213 | -3.493722 | -1.566835 |
| H | 2.221187 | -2.618230 | -3.100568 |
| H | 1.547308 | -1.460517 | 4.228296 |
| H | 0.676890 | -0.329619 | 3.173206 |
| H | 2.418668 | -0.554309 | 2.984770 |
| H | -2.272233 | -3.223832 | 3.407980 |
| H | -1.744477 | -2.146284 | 2.100692 |
| H | -1.167119 | -1.878968 | 3.749460 |
| H | -1.633848 | -5.142017 | 2.173251 |
| H | 0.041025 | -5.521400 | 1.752238 |
| H | -0.860051 | -4.339256 | 0.802181 |
| H | -0.689560 | -4.701775 | 4.550074 |
| H | 0.546675 | -3.464787 | 4.809960 |
| H | 0.955887 | -4.976588 | 3.974614 |
| H | 4.753781 | -3.306138 | 0.771878 |
| H | 4.121793 | -1.739327 | 1.307293 |
| H | 3.426648 | -2.515531 | -0.106124 |
| H | 4.471493 | -4.122098 | 3.087435 |
| H | 2.911551 | -4.094796 | 3.919663 |
| H | 3.769644 | -2.580630 | 3.596152 |
| H | 3.643426 | -5.337254 | 1.067335 |
| H | 2.121919 | -4.877196 | 0.292773 |
| H | 2.121566 | -5.520355 | 1.943957 |
| C | 1.978623 | 0.201615 | 0.250818 |

| | | | |
|---|---|---|---|
| O | 3.147979 | 0.233093 | 0.492639 |

---

Calculated energies and coordinates of **TS3a**

Electronic energy ... -3172.92120604 Eh
Total Enthalpy ... -3171.67773029 Eh
Final Gibbs free energy ... -3171.83343581 Eh

CARTESIAN COORDINATES (ANGSTROEM)

| | | | |
|---|---|---|---|
| Si | 0.201890 | 1.376852 | -0.125873 |
| Si | 0.299675 | -1.540019 | 0.224765 |
| Si | -0.641409 | -2.215406 | -1.871711 |
| Si | 1.183782 | -2.761817 | 2.116561 |
| N | -0.191249 | 2.237504 | 1.214451 |
| N | -1.852766 | 2.594361 | 2.925326 |
| N | 0.155501 | 3.475829 | 3.225937 |
| N | -0.092229 | 2.243656 | -1.503710 |
| N | -0.672590 | 3.361366 | -3.513452 |
| N | 1.472308 | 2.861448 | -3.274888 |
| C | -0.590580 | 2.699010 | 2.343176 |
| C | -1.854554 | 3.277739 | 4.135536 |
| C | -0.633799 | 3.819184 | 4.314507 |
| C | -2.993859 | 1.811700 | 2.377427 |
| C | -2.616739 | 0.327662 | 2.356883 |
| C | -4.214765 | 1.997628 | 3.283562 |
| C | -3.352664 | 2.328739 | 0.977878 |
| C | 1.555907 | 3.925966 | 2.990592 |
| C | 2.002583 | 4.809315 | 4.159016 |
| C | 1.604055 | 4.760552 | 1.703199 |
| C | 2.479668 | 2.706329 | 2.914816 |
| C | 0.229391 | 2.743313 | -2.647703 |
| C | 0.002479 | 3.829780 | -4.628511 |
| C | 1.306348 | 3.521224 | -4.486011 |
| C | -2.089708 | 3.672660 | -3.171624 |
| C | -2.857829 | 2.370498 | -2.930661 |
| C | -2.740804 | 4.414271 | -4.341630 |
| C | -2.115947 | 4.576689 | -1.931024 |
| C | 2.790244 | 2.383818 | -2.753690 |
| C | 3.907855 | 2.905320 | -3.664783 |
| C | 2.818853 | 0.854033 | -2.771236 |
| C | 3.041410 | 2.941497 | -1.345808 |
| C | -0.417510 | -0.862824 | -3.184098 |
| C | -2.567914 | -2.395556 | -1.652934 |
| C | -3.150036 | -0.979971 | -1.513702 |
| C | -3.226779 | -3.062922 | -2.870799 |
| C | -2.947863 | -3.187425 | -0.398047 |
| C | 0.234592 | -3.782560 | -2.611067 |
| C | -0.104948 | -3.979966 | -4.099128 |
| C | -0.125798 | -5.057544 | -1.839188 |
| C | 1.750796 | -3.557747 | -2.502566 |
| C | 1.469289 | -1.333499 | 3.339497 |
| C | -0.142135 | -3.905222 | 2.957503 |
| C | -1.299984 | -3.000106 | 3.413838 |
| C | -0.700860 | -4.963483 | 1.999354 |
| C | 0.415524 | -4.623183 | 4.197547 |
| C | 2.910038 | -3.621785 | 1.855630 |
| C | 3.783322 | -2.741037 | 0.947861 |
| C | 3.647459 | -3.757318 | 3.201542 |
| C | 2.780183 | -5.013068 | 1.221485 |
| H | -2.714634 | 3.329303 | 4.778929 |
| H | -0.273990 | 4.412336 | 5.136302 |
| H | -3.454383 | -0.261953 | 1.967640 |
| H | -2.393090 | -0.019572 | 3.371206 |
| H | -1.745001 | 0.132652 | 1.724395 |
| H | -5.046347 | 1.437951 | 2.847012 |
| H | -4.518664 | 3.047632 | 3.354026 |
| H | -4.043649 | 1.600660 | 4.289238 |
| H | -4.195245 | 1.750690 | 0.584679 |
| H | -2.517606 | 2.234321 | 0.283267 |
| H | -3.645243 | 3.383511 | 1.025042 |
| H | 3.027157 | 5.135571 | 3.961131 |
| H | 2.007735 | 4.262286 | 5.107603 |
| H | 1.381581 | 5.705730 | 4.259444 |
| H | 2.635682 | 5.074009 | 1.512395 |
| H | 0.982106 | 5.656436 | 1.807358 |
| H | 1.249434 | 4.187515 | 0.845265 |
| H | 3.513921 | 3.041462 | 2.782445 |
| H | 2.233873 | 2.040039 | 2.086933 |
| H | 2.421659 | 2.130876 | 3.845034 |
| H | -0.485580 | 4.325346 | -5.448930 |
| H | 2.117278 | 3.715468 | -5.164431 |
| H | -2.826740 | 1.737500 | -3.824111 |
| H | -3.904415 | 2.598366 | -2.702893 |
| H | -2.431257 | 1.817307 | -2.095428 |
| H | -3.783731 | 4.609858 | -4.078071 |
| H | -2.737254 | 3.814649 | -5.258178 |
| H | -2.263055 | 5.379516 | -4.537938 |
| H | -3.154156 | 4.807308 | -1.670170 |
| H | -1.593954 | 5.517439 | -2.137478 |
| H | -1.640100 | 4.087924 | -1.078444 |
| H | 4.859451 | 2.557696 | -3.254694 |
| H | 3.935034 | 3.999916 | -3.697174 |
| H | 3.831330 | 2.512161 | -4.683228 |
| H | 3.748614 | 0.494972 | -2.318887 |
| H | 2.757927 | 0.486508 | -3.801332 |
| H | 1.994454 | 0.431518 | -2.194277 |
| H | 4.083772 | 2.755276 | -1.070791 |
| H | 2.433614 | 2.455694 | -0.582819 |
| H | 2.857386 | 4.020405 | -1.319304 |
| H | -1.101820 | -1.053077 | -4.020498 |
| H | 0.600092 | -0.858632 | -3.583583 |
| H | -0.628383 | 0.135744 | -2.795402 |
| H | -4.223500 | -1.039370 | -1.278865 |
| H | -3.044389 | -0.410059 | -2.441062 |
| H | -2.657529 | -0.420927 | -0.707878 |
| H | -4.321403 | -3.018776 | -2.769013 |
| H | -2.952146 | -4.119000 | -2.958533 |
| H | -2.966624 | -2.563612 | -3.811224 |
| H | -4.041791 | -3.206460 | -0.279653 |
| H | -2.525629 | -2.728238 | 0.503134 |
| H | -2.605957 | -4.225901 | -0.445416 |
| H | 0.461848 | -4.837242 | -4.491584 |
| H | 0.168337 | -3.105949 | -4.700013 |
| H | -1.165401 | -4.186750 | -4.266390 |
| H | 0.464962 | -5.907870 | -2.211310 |
| H | -1.183436 | -5.321290 | -1.950921 |
| H | 0.083806 | -4.953454 | -0.769477 |
| H | 2.291394 | -4.405985 | -2.947960 |
| H | 2.064249 | -3.466365 | -1.459084 |
| H | 2.071430 | -2.647288 | -3.023967 |
| H | 1.558819 | -1.703610 | 4.368006 |
| H | 0.638513 | -0.619775 | 3.301389 |
| H | 2.378452 | -0.784205 | 3.082149 |
| H | -2.117867 | -3.612022 | 3.822595 |
| H | -1.704966 | -2.408463 | 2.584933 |
| H | -0.982078 | -2.301038 | 4.194845 |
| H | -1.497837 | -5.539792 | 2.493419 |
| H | 0.065950 | -5.673224 | 1.674202 |
| H | -1.131677 | -4.503287 | 1.108031 |
| H | -0.401967 | -5.139904 | 4.722574 |
| H | 0.877439 | -3.931005 | 4.910684 |

| | | | |
|---|---|---|---|
| H | 1.157925 | -5.381054 | 3.928475 |
| H | 4.766228 | -3.217094 | 0.812446 |
| H | 3.934741 | -1.743919 | 1.369868 |
| H | 3.338002 | -2.594333 | -0.041188 |
| H | 4.636604 | -4.208050 | 3.030465 |
| H | 3.116997 | -4.394337 | 3.914623 |
| H | 3.809209 | -2.782191 | 3.672332 |
| H | 3.779586 | -5.418792 | 1.004475 |
| H | 2.225675 | -4.986003 | 0.276899 |
| H | 2.278886 | -5.724227 | 1.886697 |
| C | 1.439355 | -0.051915 | 0.124652 |
| O | 2.494160 | 0.166981 | 0.751315 |

---

Calculated energies and coordinates of **2a**

Electronic energy ... -3172.95638105 Eh
Total Enthalpy ... -3171.71224393 Eh
Final Gibbs free energy ... -3171.87025010 Eh

CARTESIAN COORDINATES (ANGSTROEM)

| | | | |
|---|---|---|---|
| N | 4.672994 | 1.897905 | 12.966651 |
| N | 6.877178 | 1.021703 | 12.538566 |
| N | 5.148176 | 0.009723 | 11.594787 |
| N | 2.650424 | 3.740378 | 12.793451 |
| N | 2.415154 | 5.675055 | 11.368465 |
| N | 0.607167 | 4.906207 | 12.386226 |
| C | 5.486412 | 1.072074 | 12.434440 |
| C | 6.301681 | -0.659877 | 11.210633 |
| H | 6.296568 | -1.518610 | 10.562836 |
| C | 7.354861 | -0.042628 | 11.784317 |
| H | 8.399664 | -0.286278 | 11.707226 |
| C | 3.759642 | -0.322101 | 11.178309 |
| C | 3.137900 | 0.875590 | 10.445626 |
| H | 3.763379 | 1.167601 | 9.594742 |
| H | 3.018848 | 1.736192 | 11.106850 |
| H | 2.150664 | 0.591488 | 10.065624 |
| C | 2.935586 | -0.699755 | 12.415978 |
| H | 1.908398 | -0.934927 | 12.118163 |
| H | 2.910879 | 0.123834 | 13.130864 |
| H | 3.366376 | -1.578060 | 12.908489 |
| C | 3.794158 | -1.519204 | 10.224626 |
| H | 4.355284 | -1.298053 | 9.310512 |
| H | 2.766039 | -1.753591 | 9.935908 |
| H | 4.215601 | -2.410716 | 10.700725 |
| C | 7.703837 | 1.945351 | 13.362552 |
| C | 7.305913 | 1.809951 | 14.837334 |
| H | 6.264400 | 2.086761 | 15.006113 |
| H | 7.932772 | 2.471292 | 15.444137 |
| H | 7.457688 | 0.779457 | 15.177278 |
| C | 7.523590 | 3.384551 | 12.861036 |
| H | 7.813910 | 3.458956 | 11.807157 |
| H | 8.160141 | 4.054997 | 13.447509 |
| H | 6.493627 | 3.722598 | 12.972741 |
| C | 9.179911 | 1.563390 | 13.216746 |
| H | 9.378553 | 0.550957 | 13.583310 |
| H | 9.769432 | 2.256216 | 13.823078 |
| H | 9.525885 | 1.649049 | 12.181140 |
| C | 1.978317 | 4.691802 | 12.259269 |
| C | 1.335185 | 6.463892 | 10.989931 |
| H | 1.419960 | 7.286851 | 10.302722 |
| C | 0.233788 | 5.993839 | 11.608763 |
| H | -0.779141 | 6.349369 | 11.541397 |
| C | 3.826774 | 5.888350 | 10.949259 |
| C | 4.398532 | 4.598243 | 10.343629 |
| H | 3.769424 | 4.256839 | 9.514262 |
| H | 4.469114 | 3.792181 | 11.074642 |
| H | 5.401652 | 4.802344 | 9.954945 |
| C | 4.645228 | 6.358855 | 12.158671 |
| H | 5.696788 | 6.472059 | 11.873520 |
| H | 4.588716 | 5.657068 | 12.991908 |
| H | 4.276326 | 7.328169 | 12.511398 |
| C | 3.872765 | 6.977694 | 9.872662 |
| H | 3.289857 | 6.702226 | 8.987237 |
| H | 4.913869 | 7.102664 | 9.562969 |
| H | 3.524804 | 7.944785 | 10.249101 |
| C | -0.282629 | 4.142528 | 13.302154 |
| C | -0.274440 | 2.659473 | 12.907898 |
| H | -0.632927 | 2.537855 | 11.879710 |
| H | -0.939367 | 2.102090 | 13.575653 |
| H | 0.728474 | 2.237248 | 12.983088 |
| C | 0.187488 | 4.345449 | 14.748225 |
| H | 1.187077 | 3.939474 | 14.919089 |
| H | -0.507294 | 3.843247 | 15.430062 |
| H | 0.198786 | 5.412255 | 14.997870 |
| C | -1.713050 | 4.672945 | 13.169736 |
| H | -1.792388 | 5.720740 | 13.477657 |
| H | -2.355316 | 4.084805 | 13.830696 |
| H | -2.097214 | 4.565063 | 12.149740 |
| Si | 3.825799 | 3.069683 | 13.733095 |
| Si | 3.534434 | 3.079350 | 17.240401 |
| Si | 4.563610 | 4.683563 | 18.636962 |
| Si | 2.319700 | 1.090903 | 17.570303 |
| O | 4.649426 | 4.162217 | 14.784381 |
| C | 4.172953 | 4.288007 | 20.450177 |
| H | 4.717329 | 3.397382 | 20.782854 |
| H | 3.106184 | 4.103203 | 20.606011 |
| H | 4.465288 | 5.119899 | 21.102091 |
| C | 6.492625 | 4.616413 | 18.458050 |
| C | 7.175312 | 5.333258 | 19.636190 |
| H | 8.267544 | 5.271487 | 19.521062 |
| H | 6.921694 | 4.871323 | 20.596784 |
| H | 6.912012 | 6.393805 | 19.692201 |
| C | 6.929242 | 3.142540 | 18.482867 |
| H | 6.523088 | 2.596002 | 17.625078 |
| H | 6.603912 | 2.628788 | 19.395731 |
| H | 8.026994 | 3.076550 | 18.441904 |
| C | 6.970042 | 5.236201 | 17.137041 |
| H | 8.053323 | 5.076074 | 17.024261 |
| H | 6.798302 | 6.317664 | 17.109273 |
| H | 6.466437 | 4.791587 | 16.271415 |
| C | 3.755490 | 6.394222 | 18.218099 |
| C | 2.299874 | 6.359660 | 18.715678 |
| H | 1.734921 | 5.548887 | 18.238861 |
| H | 1.799553 | 7.305258 | 18.460352 |
| H | 2.232906 | 6.233944 | 19.802246 |
| C | 3.727038 | 6.663973 | 16.705662 |
| H | 4.726295 | 6.690422 | 16.262678 |
| H | 3.246134 | 7.635034 | 16.514307 |
| H | 3.160407 | 5.891872 | 16.174423 |
| C | 4.484112 | 7.548934 | 18.923830 |
| H | 5.493174 | 7.698698 | 18.526383 |
| H | 4.562807 | 7.394077 | 20.006723 |
| H | 3.930647 | 8.486558 | 18.766993 |
| C | 1.270123 | 0.855439 | 16.010697 |
| H | 0.427606 | 1.553074 | 15.990631 |
| H | 0.872668 | -0.164890 | 15.945192 |
| H | 1.873247 | 1.046669 | 15.118069 |
| C | 3.544472 | -0.408500 | 17.654855 |
| C | 4.682934 | -0.160248 | 18.654181 |
| H | 5.251194 | 0.737390 | 18.388416 |
| H | 5.380320 | -1.011056 | 18.647968 |

| | | | |
|---|---|---|---|
| H | 4.321506 | -0.037928 | 19.680464 |
| C | 4.162394 | -0.572032 | 16.254707 |
| H | 3.412174 | -0.862827 | 15.511749 |
| H | 4.931520 | -1.358035 | 16.277373 |
| H | 4.633506 | 0.355937 | 15.908207 |
| C | 2.826809 | -1.716781 | 18.024301 |
| H | 1.981666 | -1.926266 | 17.357931 |
| H | 2.454000 | -1.706054 | 19.053878 |
| H | 3.526762 | -2.561237 | 17.940197 |
| C | 1.104934 | 1.237264 | 19.067811 |
| C | 1.815467 | 1.046492 | 20.414806 |
| H | 2.672217 | 1.721574 | 20.522717 |
| H | 2.176252 | 0.021042 | 20.545772 |
| H | 1.121485 | 1.258170 | 21.241735 |
| C | -0.030013 | 0.205600 | 18.943190 |
| H | -0.612839 | 0.357211 | 18.028335 |
| H | -0.719507 | 0.305898 | 19.794419 |
| H | 0.339383 | -0.824391 | 18.939400 |
| C | 0.475829 | 2.640374 | 19.044419 |
| H | 0.014322 | 2.871097 | 18.076612 |
| H | 1.219868 | 3.418700 | 19.243628 |
| H | -0.305646 | 2.716116 | 19.815037 |
| C | 3.853295 | 3.228885 | 15.522325 |

Calculated energies and coordinates of **1b**

Electronic energy ... -3158.91334809 Eh
Total Enthalpy ... -3157.71824707 Eh
Final Gibbs free energy ... -3157.86677172 Eh

CARTESIAN COORDINATES (ANGSTROEM)

| | | | |
|---|---|---|---|
| Si | 16.796882 | 0.663155 | 6.385065 |
| Si | 14.679020 | 0.193827 | 6.973619 |
| Si | 17.781736 | 2.875012 | 6.556075 |
| Si | 18.235524 | -1.213478 | 5.824701 |
| Si | 12.743362 | 1.165291 | 5.873451 |
| Si | 14.323069 | -1.566647 | 8.612141 |
| C | 19.641232 | 2.539670 | 6.714234 |
| H | 20.092442 | 2.285837 | 5.750289 |
| H | 19.845685 | 1.721988 | 7.408237 |
| H | 20.152167 | 3.431373 | 7.095764 |
| C | 17.623081 | 4.181326 | 5.119189 |
| C | 17.812329 | 3.512888 | 3.748058 |
| H | 18.832025 | 3.138312 | 3.617553 |
| H | 17.638482 | 4.254531 | 2.954939 |
| H | 17.117202 | 2.683053 | 3.585539 |
| C | 18.725243 | 5.250163 | 5.254387 |
| H | 19.727016 | 4.816109 | 5.177650 |
| H | 18.666794 | 5.804504 | 6.194298 |
| H | 18.620586 | 5.977980 | 4.436579 |
| C | 16.259008 | 4.876614 | 5.122765 |
| H | 15.448645 | 4.151656 | 5.027466 |
| H | 16.187804 | 5.565430 | 4.268441 |
| H | 16.091554 | 5.464665 | 6.030473 |
| C | 17.219962 | 3.531229 | 8.303869 |
| C | 17.871011 | 4.885698 | 8.633024 |
| H | 17.493894 | 5.689587 | 7.993120 |
| H | 18.962953 | 4.858995 | 8.546141 |
| H | 17.632773 | 5.160286 | 9.670868 |
| C | 15.699974 | 3.682927 | 8.414801 |
| H | 15.322470 | 4.510270 | 7.807824 |
| H | 15.413041 | 3.882850 | 9.457670 |
| H | 15.185875 | 2.765724 | 8.100213 |
| C | 17.680254 | 2.511965 | 9.363639 |
| H | 18.770422 | 2.408453 | 9.390960 |
| H | 17.244133 | 1.521710 | 9.187893 |
| H | 17.354876 | 2.847212 | 10.359026 |
| C | 17.104350 | -2.729209 | 5.808080 |
| H | 16.881415 | -3.111342 | 6.803962 |
| H | 17.562109 | -3.546012 | 5.239177 |
| H | 16.155612 | -2.469814 | 5.332163 |
| C | 19.781523 | -1.619903 | 6.944314 |
| C | 19.514452 | -1.208244 | 8.400063 |
| H | 19.300272 | -0.138170 | 8.495185 |
| H | 20.396140 | -1.432857 | 9.017829 |
| H | 18.668180 | -1.752496 | 8.825612 |
| C | 21.077881 | -0.935234 | 6.484751 |
| H | 20.989667 | 0.153274 | 6.454219 |
| H | 21.402984 | -1.276578 | 5.497900 |
| H | 21.883819 | -1.178868 | 7.192029 |
| C | 20.018268 | -3.143299 | 6.921342 |
| H | 19.170390 | -3.695993 | 7.338060 |
| H | 20.901501 | -3.384778 | 7.530180 |
| H | 20.202287 | -3.522493 | 5.910520 |
| C | 18.689180 | -0.994394 | 3.941869 |
| C | 19.488321 | 0.284652 | 3.677216 |
| H | 20.479676 | 0.265369 | 4.137615 |
| H | 18.951609 | 1.155079 | 4.058093 |
| H | 19.626356 | 0.429680 | 2.595568 |
| C | 19.490213 | -2.200542 | 3.420728 |
| H | 20.470446 | -2.291839 | 3.897813 |
| H | 19.664863 | -2.079240 | 2.341576 |
| H | 18.955772 | -3.146799 | 3.555934 |
| C | 17.380663 | -0.914163 | 3.134345 |
| H | 16.740676 | -0.089108 | 3.468257 |
| H | 16.796613 | -1.837266 | 3.205120 |
| H | 17.613084 | -0.750527 | 2.072177 |
| C | 11.363975 | -0.101542 | 6.173873 |
| H | 10.912916 | 0.011834 | 7.164003 |
| H | 11.732859 | -1.125619 | 6.086476 |
| H | 10.569684 | 0.028562 | 5.429566 |
| C | 11.987494 | 2.878222 | 6.418037 |
| C | 12.782145 | 4.064551 | 5.865166 |
| H | 13.812100 | 4.049586 | 6.226516 |
| H | 12.328920 | 5.007439 | 6.204558 |
| H | 12.798600 | 4.086198 | 4.771220 |
| C | 11.948729 | 3.009092 | 7.949182 |
| H | 12.933037 | 2.876356 | 8.409896 |
| H | 11.257382 | 2.291928 | 8.401251 |
| H | 11.587740 | 4.012927 | 8.216298 |
| C | 10.530840 | 2.986139 | 5.923589 |
| H | 10.444882 | 2.947664 | 4.834824 |
| H | 10.115496 | 3.950188 | 6.251292 |
| H | 9.896121 | 2.199091 | 6.342572 |
| C | 13.144154 | 1.038700 | 3.967808 |
| C | 14.381824 | 1.845507 | 3.565421 |
| H | 15.238164 | 1.606801 | 4.208498 |
| H | 14.207280 | 2.923833 | 3.616781 |
| H | 14.669197 | 1.609184 | 2.530128 |
| C | 13.407787 | -0.443935 | 3.642086 |
| H | 14.249931 | -0.840669 | 4.220925 |
| H | 13.658749 | -0.546013 | 2.576418 |
| H | 12.530260 | -1.070002 | 3.836459 |
| C | 11.958517 | 1.509231 | 3.107615 |
| H | 11.791846 | 2.587577 | 3.191985 |
| H | 11.024287 | 0.995128 | 3.359123 |
| H | 12.173160 | 1.297357 | 2.050245 |
| C | 16.036450 | -2.040227 | 9.263038 |
| H | 16.643506 | -1.141751 | 9.398303 |
| H | 16.583103 | -2.714941 | 8.604778 |
| H | 15.944027 | -2.540450 | 10.233456 |

| | | | |
|---|---|---|---|
| C | 13.512345 | -0.686165 | 10.149905 |
| C | 12.144513 | -0.085777 | 9.816079 |
| H | 12.225576 | 0.586771 | 8.960780 |
| H | 11.769129 | 0.502724 | 10.666214 |
| H | 11.395583 | -0.846422 | 9.581560 |
| C | 14.436727 | 0.459490 | 10.600298 |
| H | 14.605342 | 1.189639 | 9.800108 |
| H | 15.414801 | 0.095368 | 10.931162 |
| H | 13.978256 | 0.989841 | 11.447494 |
| C | 13.356558 | -1.658062 | 11.332234 |
| H | 12.667643 | -2.479414 | 11.114494 |
| H | 12.948598 | -1.112846 | 12.195988 |
| H | 14.312055 | -2.090322 | 11.647054 |
| C | 13.397444 | -3.209262 | 8.094667 |
| C | 13.964936 | -4.375084 | 8.929405 |
| H | 13.835150 | -4.220418 | 10.005962 |
| H | 15.030769 | -4.534931 | 8.738924 |
| H | 13.438923 | -5.303445 | 8.663745 |
| C | 11.877514 | -3.170087 | 8.320868 |
| H | 11.613549 | -3.074806 | 9.377837 |
| H | 11.438488 | -4.112631 | 7.963246 |
| H | 11.390739 | -2.358007 | 7.775641 |
| C | 13.646844 | -3.529879 | 6.612482 |
| H | 14.704586 | -3.712676 | 6.410459 |
| H | 13.317869 | -2.720940 | 5.950653 |
| H | 13.096750 | -4.439959 | 6.332199 |

---

Calculated energies and coordinates of **TS1b**

Electronic energy ... -3272.19085538 Eh
Total Enthalpy ... -3270.98603510 Eh
Final Gibbs free energy ... -3271.13802632 Eh

CARTESIAN COORDINATES (ANGSTROEM)

| | | | |
|---|---|---|---|
| Si | 16.810888 | 0.455853 | 6.383602 |
| Si | 14.604025 | -0.099551 | 6.623088 |
| Si | 17.785166 | 2.700241 | 6.480349 |
| Si | 18.363801 | -1.371311 | 5.866608 |
| Si | 12.650054 | 1.177179 | 5.683329 |
| Si | 14.293692 | -1.663012 | 8.530602 |
| C | 19.648903 | 2.415931 | 6.650371 |
| H | 20.110115 | 2.143242 | 5.696991 |
| H | 19.883340 | 1.639145 | 7.379081 |
| H | 20.123200 | 3.342044 | 6.995275 |
| C | 17.623881 | 4.025845 | 5.058009 |
| C | 17.723834 | 3.366458 | 3.672676 |
| H | 18.730110 | 2.976117 | 3.491142 |
| H | 17.522290 | 4.115754 | 2.893463 |
| H | 17.016107 | 2.543969 | 3.536892 |
| C | 18.781980 | 5.040743 | 5.149619 |
| H | 19.754231 | 4.570579 | 4.974200 |
| H | 18.822858 | 5.554607 | 6.114190 |
| H | 18.645255 | 5.808513 | 4.374030 |
| C | 16.306320 | 4.804658 | 5.156176 |
| H | 15.443875 | 4.140874 | 5.219558 |
| H | 16.174029 | 5.434685 | 4.264671 |
| H | 16.289474 | 5.466211 | 6.028059 |
| C | 17.220280 | 3.367927 | 8.220758 |
| C | 17.856722 | 4.726443 | 8.558396 |
| H | 17.506239 | 5.525624 | 7.898343 |
| H | 18.950997 | 4.696670 | 8.511431 |
| H | 17.582780 | 5.011475 | 9.584778 |
| C | 15.697130 | 3.499768 | 8.300793 |
| H | 15.325293 | 4.342085 | 7.711503 |
| H | 15.378364 | 3.658349 | 9.341715 |
| H | 15.209590 | 2.586596 | 7.939879 |
| C | 17.676050 | 2.357652 | 9.289822 |
| H | 18.767441 | 2.281220 | 9.344591 |
| H | 17.271817 | 1.356487 | 9.097280 |
| H | 17.317437 | 2.680872 | 10.277876 |
| C | 17.445609 | -3.025736 | 5.941559 |
| H | 17.332871 | -3.430009 | 6.947938 |
| H | 18.011074 | -3.767882 | 5.367087 |
| H | 16.455772 | -2.953173 | 5.496652 |
| C | 19.936493 | -1.595018 | 7.014828 |
| C | 19.615602 | -1.144696 | 8.448951 |
| H | 19.330377 | -0.089452 | 8.499649 |
| H | 20.496146 | -1.289700 | 9.091819 |
| H | 18.794274 | -1.723509 | 8.881884 |
| C | 21.194245 | -0.854193 | 6.534058 |
| H | 21.049562 | 0.222211 | 6.438979 |
| H | 21.550791 | -1.232313 | 5.571337 |
| H | 22.003671 | -1.011530 | 7.261796 |
| C | 20.297904 | -3.094647 | 7.068685 |
| H | 19.506842 | -3.698317 | 7.522240 |
| H | 21.201408 | -3.222440 | 7.681935 |
| H | 20.513300 | -3.508621 | 6.077673 |
| C | 18.805674 | -1.185477 | 3.980211 |
| C | 19.497117 | 0.146398 | 3.677050 |
| H | 20.493164 | 0.211925 | 4.123853 |
| H | 18.897638 | 0.980554 | 4.048217 |
| H | 19.610143 | 0.276628 | 2.590668 |
| C | 19.704398 | -2.335244 | 3.492278 |
| H | 20.682911 | -2.340641 | 3.982319 |
| H | 19.883228 | -2.221557 | 2.413043 |
| H | 19.244231 | -3.317683 | 3.639563 |
| C | 17.492250 | -1.234368 | 3.179367 |
| H | 16.802159 | -0.438688 | 3.489338 |
| H | 16.975937 | -2.192211 | 3.292750 |
| H | 17.705496 | -1.095670 | 2.109627 |
| C | 11.250958 | -0.100188 | 5.768753 |
| H | 10.890539 | -0.251757 | 6.790694 |
| H | 11.571728 | -1.069289 | 5.382744 |
| H | 10.399174 | 0.239234 | 5.168976 |
| C | 11.910107 | 2.786231 | 6.538005 |
| C | 12.553214 | 4.092425 | 6.052050 |
| H | 13.633927 | 4.093381 | 6.213917 |
| H | 12.136667 | 4.935756 | 6.621674 |
| H | 12.363593 | 4.292479 | 4.994699 |
| C | 12.046184 | 2.763858 | 8.065540 |
| H | 13.080447 | 2.908469 | 8.381416 |
| H | 11.673919 | 1.837985 | 8.510841 |
| H | 11.457822 | 3.589853 | 8.491161 |
| C | 10.396839 | 2.842891 | 6.235815 |
| H | 10.161534 | 2.809884 | 5.169246 |
| H | 9.995963 | 3.786781 | 6.631953 |
| H | 9.855612 | 2.027490 | 6.725380 |
| C | 12.974607 | 1.499235 | 3.777601 |
| C | 14.183494 | 2.412248 | 3.555885 |
| H | 15.053971 | 2.041974 | 4.110058 |
| H | 13.992792 | 3.441535 | 3.862734 |
| H | 14.453925 | 2.433129 | 2.489991 |
| C | 13.267574 | 0.190986 | 3.029430 |
| H | 14.249138 | -0.206561 | 3.301271 |
| H | 13.300177 | 0.395348 | 1.949394 |
| H | 12.506431 | -0.578924 | 3.193675 |
| C | 11.736776 | 2.127415 | 3.108308 |
| H | 11.497241 | 3.118964 | 3.499092 |
| H | 10.847668 | 1.494372 | 3.204567 |
| H | 11.936711 | 2.245048 | 2.033609 |
| C | 16.040908 | -2.025196 | 9.147616 |

| | | | |
|---|---|---|---|
| H | 16.458849 | -1.153941 | 9.660904 |
| H | 16.712866 | -2.267176 | 8.326439 |
| H | 16.036153 | -2.866646 | 9.851351 |
| C | 13.463615 | -0.693245 | 9.996677 |
| C | 11.943727 | -0.581931 | 9.822781 |
| H | 11.675626 | -0.193887 | 8.835558 |
| H | 11.520638 | 0.099589 | 10.575082 |
| H | 11.447629 | -1.550277 | 9.941794 |
| C | 14.083589 | 0.709149 | 10.045589 |
| H | 13.950451 | 1.243178 | 9.105965 |
| H | 15.159091 | 0.670946 | 10.248856 |
| H | 13.612353 | 1.305019 | 10.841194 |
| C | 13.760789 | -1.350886 | 11.358372 |
| H | 13.312144 | -2.339501 | 11.468584 |
| H | 13.348034 | -0.713436 | 12.153960 |
| H | 14.836135 | -1.441337 | 11.542363 |
| C | 13.435817 | -3.380045 | 8.193838 |
| C | 13.094046 | -4.083776 | 9.520903 |
| H | 12.293381 | -3.579821 | 10.068887 |
| H | 13.962679 | -4.176095 | 10.182281 |
| H | 12.743878 | -5.102500 | 9.300016 |
| C | 12.141802 | -3.261745 | 7.377256 |
| H | 11.406661 | -2.605231 | 7.853194 |
| H | 11.680637 | -4.253910 | 7.266314 |
| H | 12.331143 | -2.874680 | 6.372234 |
| C | 14.412835 | -4.304118 | 7.448240 |
| H | 15.310516 | -4.507519 | 8.041664 |
| H | 14.733597 | -3.899398 | 6.488342 |
| H | 13.923672 | -5.268758 | 7.250495 |
| C | 13.973631 | -1.829257 | 4.992575 |
| O | 14.538991 | -2.607402 | 4.365189 |

---

Calculated energies and coordinates of **INT1b**

Electronic energy ... -3272.19818716 Eh
Total Enthalpy ... -3270.99226272 Eh
Final Gibbs free energy ... -3271.14549093 Eh

CARTESIAN COORDINATES (ANGSTROEM)

| | | | |
|---|---|---|---|
| Si | 17.024417 | 0.446125 | 6.750489 |
| Si | 14.672495 | 0.008791 | 6.719095 |
| Si | 17.920176 | 2.691062 | 6.692687 |
| Si | 18.326939 | -1.333792 | 5.746769 |
| Si | 12.699053 | 1.096907 | 5.469527 |
| Si | 14.197631 | -1.573488 | 8.697954 |
| C | 19.805975 | 2.495161 | 6.771205 |
| H | 20.234525 | 2.286519 | 5.785983 |
| H | 20.119618 | 1.703816 | 7.454900 |
| H | 20.246739 | 3.433027 | 7.131384 |
| C | 17.612034 | 3.961196 | 5.249035 |
| C | 17.808012 | 3.271619 | 3.890455 |
| H | 18.861972 | 3.036737 | 3.712492 |
| H | 17.484004 | 3.942524 | 3.081119 |
| H | 17.240417 | 2.339981 | 3.809228 |
| C | 18.596795 | 5.143524 | 5.297878 |
| H | 19.635124 | 4.810992 | 5.196388 |
| H | 18.516576 | 5.726560 | 6.217944 |
| H | 18.387693 | 5.822767 | 4.458026 |
| C | 16.180001 | 4.494526 | 5.326729 |
| H | 15.467767 | 3.666945 | 5.386462 |
| H | 15.933961 | 5.083227 | 4.430224 |
| H | 16.026363 | 5.140593 | 6.196882 |
| C | 17.406524 | 3.404439 | 8.434917 |
| C | 17.864215 | 4.853797 | 8.661327 |
| H | 17.336163 | 5.562394 | 8.016059 |
| H | 18.940872 | 4.979925 | 8.499766 |
| H | 17.654780 | 5.146147 | 9.700999 |
| C | 15.887825 | 3.329040 | 8.618968 |
| H | 15.352439 | 3.991906 | 7.932088 |
| H | 15.602771 | 3.609985 | 9.644210 |
| H | 15.542687 | 2.302789 | 8.451166 |
| C | 18.061950 | 2.531029 | 9.520722 |
| H | 19.154632 | 2.608867 | 9.502580 |
| H | 17.792492 | 1.474279 | 9.401661 |
| H | 17.723330 | 2.860155 | 10.514214 |
| C | 17.383808 | -2.979431 | 5.827552 |
| H | 17.096551 | -3.258254 | 6.843429 |
| H | 18.033741 | -3.777060 | 5.453287 |
| H | 16.486275 | -2.983732 | 5.209691 |
| C | 19.939507 | -1.658030 | 6.827644 |
| C | 19.695377 | -1.249823 | 8.289247 |
| H | 19.490955 | -0.179843 | 8.394592 |
| H | 20.583499 | -1.488482 | 8.893484 |
| H | 18.843610 | -1.781966 | 8.725956 |
| C | 21.186664 | -0.921277 | 6.316243 |
| H | 21.031221 | 0.153778 | 6.208422 |
| H | 21.520909 | -1.313446 | 5.350457 |
| H | 22.013528 | -1.066150 | 7.027194 |
| C | 20.274273 | -3.163419 | 6.827701 |
| H | 19.494572 | -3.760613 | 7.310051 |
| H | 21.206154 | -3.323872 | 7.389276 |
| H | 20.429440 | -3.560570 | 5.818613 |
| C | 18.702524 | -1.102361 | 3.852593 |
| C | 19.564767 | 0.135014 | 3.576271 |
| H | 20.608914 | -0.013188 | 3.860985 |
| H | 19.184427 | 1.002376 | 4.119541 |
| H | 19.545170 | 0.379788 | 2.504018 |
| C | 19.398628 | -2.338543 | 3.259672 |
| H | 20.371777 | -2.528264 | 3.725606 |
| H | 19.577790 | -2.182548 | 2.185537 |
| H | 18.790104 | -3.243576 | 3.359521 |
| C | 17.355503 | -0.912278 | 3.134411 |
| H | 16.822877 | -0.036094 | 3.523028 |
| H | 16.700160 | -1.783314 | 3.243904 |
| H | 17.521924 | -0.757860 | 2.058291 |
| C | 11.337603 | -0.213744 | 5.578995 |
| H | 11.063913 | -0.435973 | 6.614618 |
| H | 11.642580 | -1.151760 | 5.109170 |
| H | 10.435314 | 0.135172 | 5.065538 |
| C | 11.953732 | 2.705347 | 6.311504 |
| C | 12.502089 | 4.032055 | 5.767981 |
| H | 13.581149 | 4.118638 | 5.911138 |
| H | 12.033668 | 4.859759 | 6.319273 |
| H | 12.283214 | 4.186192 | 4.708703 |
| C | 12.210858 | 2.698180 | 7.819829 |
| H | 13.274440 | 2.816280 | 8.040970 |
| H | 11.853981 | 1.781798 | 8.296182 |
| H | 11.682088 | 3.538815 | 8.291811 |
| C | 10.422952 | 2.695354 | 6.108902 |
| H | 10.126392 | 2.657675 | 5.056676 |
| H | 10.008634 | 3.620593 | 6.533293 |
| H | 9.946327 | 1.856956 | 6.625627 |
| C | 13.052987 | 1.361307 | 3.569067 |
| C | 14.218586 | 2.319980 | 3.302575 |
| H | 15.131180 | 1.968168 | 3.792846 |
| H | 14.018669 | 3.336165 | 3.646913 |
| H | 14.418769 | 2.369490 | 2.222680 |
| C | 13.378488 | 0.045741 | 2.843115 |
| H | 14.381926 | -0.311697 | 3.081944 |
| H | 13.359353 | 0.230152 | 1.759959 |
| H | 12.660391 | -0.753679 | 3.050602 |

| | | | |
|---|---|---|---|
| C | 11.770661 | 1.926209 | 2.922102 |
| H | 11.484697 | 2.902341 | 3.320497 |
| H | 10.918669 | 1.246016 | 3.030695 |
| H | 11.948595 | 2.055690 | 1.845345 |
| C | 15.946362 | -1.936177 | 9.297838 |
| H | 16.384782 | -1.067669 | 9.797210 |
| H | 16.614283 | -2.189348 | 8.473607 |
| H | 15.927428 | -2.774386 | 10.005817 |
| C | 13.331236 | -0.630127 | 10.151115 |
| C | 11.813446 | -0.521585 | 9.949934 |
| H | 11.554889 | -0.119767 | 8.964580 |
| H | 11.375048 | 0.145675 | 10.705708 |
| H | 11.320639 | -1.494318 | 10.045985 |
| C | 13.947881 | 0.771200 | 10.250829 |
| H | 13.781588 | 1.355080 | 9.347566 |
| H | 15.028338 | 0.729998 | 10.427453 |
| H | 13.495192 | 1.322733 | 11.087550 |
| C | 13.607726 | -1.325577 | 11.500115 |
| H | 13.173905 | -2.324431 | 11.568710 |
| H | 13.165041 | -0.719286 | 12.303587 |
| H | 14.680274 | -1.401916 | 11.705544 |
| C | 13.368538 | -3.282946 | 8.272916 |
| C | 12.987446 | -4.020627 | 9.570982 |
| H | 12.167821 | -3.533327 | 10.105970 |
| H | 13.835367 | -4.128133 | 10.256175 |
| H | 12.648346 | -5.034027 | 9.312297 |
| C | 12.107068 | -3.158163 | 7.410347 |
| H | 11.347550 | -2.519103 | 7.872156 |
| H | 11.659407 | -4.151739 | 7.264895 |
| H | 12.332062 | -2.756205 | 6.420428 |
| C | 14.382536 | -4.175129 | 7.536886 |
| H | 15.266016 | -4.379967 | 8.150137 |
| H | 14.721244 | -3.744242 | 6.594233 |
| H | 13.912384 | -5.141148 | 7.304073 |
| C | 14.416916 | -1.305041 | 5.465343 |
| O | 14.360689 | -2.183531 | 4.711290 |

---

Calculated energies and coordinates of **TS2b**

Electronic energy ... -3272.19445996 Eh
Total Enthalpy ... -3270.98955538 Eh
Final Gibbs free energy ... -3271.14080509 Eh

CARTESIAN COORDINATES (ANGSTROEM)

| | | | |
|---|---|---|---|
| C | 18.259376 | 5.076032 | 6.363935 |
| C | 14.894276 | 10.827879 | 5.420557 |
| C | 12.831877 | 9.422650 | 5.433230 |
| C | 16.738021 | 9.181816 | 3.390696 |
| C | 14.554867 | 7.033057 | 2.918939 |
| C | 14.817110 | 7.554322 | 1.492830 |
| C | 13.045966 | 6.791630 | 3.071759 |
| C | 15.270313 | 5.683123 | 3.065804 |
| C | 14.092353 | 9.772297 | 4.633778 |
| C | 13.624435 | 10.427761 | 3.318936 |
| C | 13.257895 | 6.109021 | 6.795886 |
| C | 15.183602 | 4.587199 | 8.500804 |
| C | 15.168053 | 3.635419 | 7.284633 |
| C | 14.060300 | 4.111536 | 9.444853 |
| C | 16.509526 | 4.475333 | 9.267423 |
| C | 14.392387 | 7.642534 | 9.261381 |
| C | 12.879513 | 7.549951 | 9.546064 |
| C | 14.700806 | 9.092617 | 8.861619 |
| C | 15.134504 | 7.371302 | 10.581211 |
| C | 21.879590 | 6.111702 | 4.870883 |
| C | 19.932292 | 4.159305 | 3.554550 |
| C | 20.760732 | 3.879574 | 2.282787 |
| C | 18.474253 | 3.785567 | 3.246688 |
| C | 20.511735 | 3.232111 | 4.642425 |
| C | 20.176295 | 7.260220 | 2.604423 |
| C | 21.491237 | 7.156502 | 1.800898 |
| C | 19.008400 | 6.993385 | 1.640423 |
| C | 20.135345 | 8.698502 | 3.153193 |
| C | 18.446702 | 7.753491 | 9.178908 |
| C | 21.146428 | 6.742932 | 10.369094 |
| C | 21.213323 | 6.502384 | 8.845535 |
| C | 20.846722 | 5.031142 | 8.592661 |
| C | 22.663156 | 6.755440 | 8.407570 |
| C | 20.467437 | 9.467097 | 7.648870 |
| C | 21.571768 | 9.565246 | 6.590965 |
| C | 19.240583 | 10.250974 | 7.147254 |
| C | 20.969678 | 10.127314 | 8.945201 |
| O | 18.073407 | 3.948867 | 6.582010 |
| Si | 16.344070 | 7.323354 | 6.174707 |
| Si | 18.666241 | 6.811366 | 5.988937 |
| Si | 14.833824 | 6.373239 | 7.822042 |
| Si | 15.297667 | 8.280751 | 4.228050 |
| Si | 20.165234 | 6.015889 | 4.088792 |
| Si | 19.899839 | 7.651654 | 7.988683 |
| H | 14.222213 | 11.628795 | 5.761587 |
| H | 15.389191 | 10.401904 | 6.300140 |
| H | 15.665077 | 11.291898 | 4.797519 |
| H | 13.070216 | 9.026435 | 6.421576 |
| H | 12.230834 | 10.330952 | 5.586488 |
| H | 12.198518 | 8.694519 | 4.918150 |
| H | 17.468668 | 8.488823 | 2.982873 |
| H | 16.395906 | 9.815213 | 2.565227 |
| H | 17.253911 | 9.817689 | 4.116082 |
| H | 14.386157 | 6.854202 | 0.762121 |
| H | 14.365375 | 8.535403 | 1.313468 |
| H | 15.887551 | 7.635211 | 1.276463 |
| H | 12.456816 | 7.689098 | 2.860482 |
| H | 12.724901 | 6.018989 | 2.357701 |
| H | 12.781894 | 6.440215 | 4.072971 |
| H | 14.960966 | 4.998739 | 2.261427 |
| H | 16.357872 | 5.794249 | 3.012027 |
| H | 15.051133 | 5.210285 | 4.029123 |
| H | 13.049157 | 11.334903 | 3.556093 |
| H | 14.457080 | 10.733130 | 2.678024 |
| H | 12.968267 | 9.772713 | 2.738004 |
| H | 13.476438 | 5.430468 | 5.967233 |
| H | 12.467393 | 5.656719 | 7.405583 |
| H | 12.858535 | 7.029624 | 6.372299 |
| H | 15.623172 | 2.671960 | 7.550144 |
| H | 14.139441 | 3.439053 | 6.965723 |
| H | 15.706877 | 4.037106 | 6.421474 |
| H | 13.074930 | 4.157256 | 8.969740 |
| H | 14.240447 | 3.060607 | 9.715463 |
| H | 14.015735 | 4.682093 | 10.376481 |
| H | 16.744701 | 3.420341 | 9.468909 |
| H | 17.350016 | 4.895447 | 8.713988 |
| H | 16.458650 | 4.993365 | 10.229419 |
| H | 12.622437 | 8.230430 | 10.370940 |
| H | 12.279272 | 7.843647 | 8.678668 |
| H | 12.570202 | 6.542446 | 9.843750 |
| H | 14.160896 | 9.399457 | 7.963452 |
| H | 14.398765 | 9.770985 | 9.673348 |
| H | 15.766272 | 9.248786 | 8.666720 |
| H | 14.848706 | 8.138306 | 11.315952 |
| H | 14.878856 | 6.402229 | 11.016296 |
| H | 16.219470 | 7.420708 | 10.464898 |
| H | 21.925523 | 5.548901 | 5.804630 |

| | | | |
|---|---|---|---|
| H | 22.615143 | 5.672273 | 4.186047 |
| H | 22.182061 | 7.142681 | 5.074320 |
| H | 20.724538 | 2.800961 | 2.073855 |
| H | 20.367057 | 4.391205 | 1.401174 |
| H | 21.816100 | 4.149761 | 2.400140 |
| H | 18.097565 | 4.339112 | 2.381230 |
| H | 18.410324 | 2.715449 | 3.004456 |
| H | 17.793661 | 3.974145 | 4.079537 |
| H | 20.325145 | 2.187525 | 4.357285 |
| H | 21.596149 | 3.356373 | 4.730496 |
| H | 20.074940 | 3.378397 | 5.629745 |
| H | 21.617232 | 6.191878 | 1.305733 |
| H | 21.484103 | 7.928657 | 1.018292 |
| H | 22.369509 | 7.339447 | 2.428176 |
| H | 18.893501 | 7.830813 | 0.938204 |
| H | 19.183751 | 6.090915 | 1.047213 |
| H | 18.053078 | 6.856481 | 2.152716 |
| H | 21.105324 | 8.969912 | 3.581255 |
| H | 19.930449 | 9.407358 | 2.339158 |
| H | 19.372790 | 8.858158 | 3.919649 |
| H | 18.214445 | 6.768175 | 9.593063 |
| H | 18.644888 | 8.433234 | 10.015287 |
| H | 17.557437 | 8.103403 | 8.643652 |
| H | 20.177928 | 6.459418 | 10.790180 |
| H | 21.913735 | 6.128855 | 10.862357 |
| H | 21.339031 | 7.787157 | 10.636908 |
| H | 21.529180 | 4.374431 | 9.151071 |
| H | 19.828111 | 4.802311 | 8.928498 |
| H | 20.915062 | 4.757637 | 7.534932 |
| H | 23.008176 | 7.748756 | 8.712739 |
| H | 23.323899 | 6.021261 | 8.890536 |
| H | 22.807157 | 6.667342 | 7.329067 |
| H | 21.248921 | 9.127015 | 5.643811 |
| H | 21.813449 | 10.620549 | 6.398490 |
| H | 22.494232 | 9.066176 | 6.901897 |
| H | 19.526382 | 11.291505 | 6.935698 |
| H | 18.825437 | 9.826450 | 6.225075 |
| H | 18.432836 | 10.270793 | 7.886245 |
| H | 21.887607 | 9.661011 | 9.318066 |
| H | 21.197586 | 11.185287 | 8.749399 |
| H | 20.221901 | 10.099427 | 9.744688 |

Calculated energies and coordinates of **INT2b**

Electronic energy ... -3272.22098817 Eh
Total Enthalpy ... -3271.01521261 Eh
Final Gibbs free energy ... -3271.16755042 Eh

CARTESIAN COORDINATES (ANGSTROEM)

| | | | |
|---|---|---|---|
| C | 17.518232 | 5.381276 | 5.977500 |
| C | 15.319688 | 10.279354 | 4.603993 |
| C | 13.042263 | 9.374789 | 5.052285 |
| C | 16.560238 | 7.760950 | 2.895440 |
| C | 14.020882 | 6.100160 | 3.260234 |
| C | 14.215653 | 6.133538 | 1.729838 |
| C | 12.512756 | 6.211025 | 3.529158 |
| C | 14.530404 | 4.740062 | 3.764353 |
| C | 14.258189 | 9.272114 | 4.124143 |
| C | 13.805673 | 9.681811 | 2.709423 |
| C | 13.168345 | 6.358923 | 7.126477 |
| C | 15.051928 | 4.831333 | 8.890850 |
| C | 14.822134 | 3.735610 | 7.831511 |
| C | 13.988957 | 4.636990 | 9.990278 |
| C | 16.438489 | 4.621952 | 9.508291 |
| C | 14.581748 | 8.068538 | 9.270371 |
| C | 13.081377 | 8.181031 | 9.613428 |
| C | 15.004217 | 9.383400 | 8.595665 |
| C | 15.342996 | 7.940875 | 10.600277 |
| C | 21.863916 | 6.418405 | 4.859247 |
| C | 19.985837 | 4.942500 | 3.045764 |
| C | 21.049869 | 4.794280 | 1.940146 |
| C | 18.600648 | 4.745903 | 2.421148 |
| C | 20.223063 | 3.810749 | 4.064936 |
| C | 20.436279 | 8.194625 | 2.781262 |
| C | 21.936103 | 8.333902 | 2.445117 |
| C | 19.678704 | 8.105138 | 1.446189 |
| C | 20.001456 | 9.481652 | 3.500255 |
| C | 18.464730 | 7.668170 | 9.139778 |
| C | 20.815174 | 6.005245 | 10.248517 |
| C | 21.007818 | 6.023467 | 8.717498 |
| C | 20.499524 | 4.680291 | 8.169022 |
| C | 22.515533 | 6.144719 | 8.450258 |
| C | 20.763605 | 9.221410 | 7.961199 |
| C | 21.978151 | 9.357862 | 7.035720 |
| C | 19.699737 | 10.242707 | 7.517153 |
| C | 21.216616 | 9.584009 | 9.388567 |
| O | 17.521808 | 4.174390 | 5.955757 |
| Si | 16.283381 | 6.900795 | 6.083742 |
| Si | 18.744717 | 6.910954 | 5.923419 |
| Si | 14.816147 | 6.563097 | 8.041082 |
| Si | 15.097508 | 7.519742 | 4.051854 |
| Si | 20.212568 | 6.644664 | 3.956519 |
| Si | 19.927278 | 7.466337 | 7.975200 |
| H | 14.868702 | 11.277660 | 4.697926 |
| H | 15.732921 | 10.007043 | 5.582632 |
| H | 16.154070 | 10.362256 | 3.899751 |
| H | 13.313617 | 9.182997 | 6.092755 |
| H | 12.628258 | 10.392551 | 5.008843 |
| H | 12.242788 | 8.681450 | 4.775618 |
| H | 17.072700 | 6.812664 | 2.727542 |
| H | 16.261794 | 8.162615 | 1.921528 |
| H | 17.280942 | 8.443018 | 3.348735 |
| H | 13.584292 | 5.359790 | 1.270152 |
| H | 13.926633 | 7.095723 | 1.293062 |
| H | 15.250496 | 5.926633 | 1.440353 |
| H | 12.082608 | 7.106683 | 3.068917 |
| H | 12.001442 | 5.343854 | 3.086957 |
| H | 12.265680 | 6.227476 | 4.592875 |
| H | 13.999677 | 3.927165 | 3.248089 |
| H | 15.601255 | 4.607875 | 3.574642 |
| H | 14.379915 | 4.608523 | 4.840096 |
| H | 13.419723 | 10.711244 | 2.735619 |
| H | 14.625228 | 9.661049 | 1.984276 |
| H | 12.999382 | 9.043421 | 2.333280 |
| H | 13.261086 | 5.597534 | 6.350369 |
| H | 12.384625 | 6.032293 | 7.819669 |
| H | 12.829202 | 7.280314 | 6.652501 |
| H | 15.019882 | 2.752910 | 8.282740 |
| H | 13.786628 | 3.726017 | 7.477142 |
| H | 15.488579 | 3.837290 | 6.970719 |
| H | 12.969594 | 4.760688 | 9.609335 |
| H | 14.064646 | 3.611794 | 10.380461 |
| H | 14.121528 | 5.315607 | 10.837158 |
| H | 16.499115 | 3.619009 | 9.954522 |
| H | 17.222922 | 4.683936 | 8.747199 |
| H | 16.652218 | 5.346978 | 10.300592 |
| H | 12.936364 | 9.012039 | 10.318307 |
| H | 12.466005 | 8.386174 | 8.732322 |
| H | 12.696644 | 7.274354 | 10.092696 |
| H | 14.434566 | 9.577928 | 7.682456 |
| H | 14.824472 | 10.226899 | 9.277836 |

| | | | |
|---|---|---|---|
| H | 16.064469 | 9.392830 | 8.325413 |
| H | 15.140547 | 8.831528 | 11.212510 |
| H | 15.022298 | 7.073362 | 11.183077 |
| H | 16.422381 | 7.876758 | 10.465412 |
| H | 21.781512 | 5.626505 | 5.605339 |
| H | 22.647242 | 6.126169 | 4.150487 |
| H | 22.197651 | 7.323234 | 5.367429 |
| H | 20.978497 | 3.784363 | 1.511148 |
| H | 20.915341 | 5.503786 | 1.119268 |
| H | 22.068480 | 4.907275 | 2.326346 |
| H | 18.383335 | 5.494377 | 1.651933 |
| H | 18.545635 | 3.757244 | 1.943405 |
| H | 17.815352 | 4.779593 | 3.183156 |
| H | 20.031537 | 2.843786 | 3.578379 |
| H | 21.258773 | 3.794673 | 4.418359 |
| H | 19.556456 | 3.876616 | 4.929081 |
| H | 22.331639 | 7.448105 | 1.936492 |
| H | 22.073501 | 9.189602 | 1.768858 |
| H | 22.547816 | 8.515884 | 3.333898 |
| H | 19.864910 | 9.022346 | 0.868953 |
| H | 20.016663 | 7.266915 | 0.831217 |
| H | 18.600528 | 8.015634 | 1.576151 |
| H | 20.563301 | 9.644840 | 4.424305 |
| H | 20.181986 | 10.350259 | 2.850611 |
| H | 18.939285 | 9.476602 | 3.762561 |
| H | 17.949200 | 6.715789 | 9.272040 |
| H | 18.763819 | 8.032203 | 10.128218 |
| H | 17.745917 | 8.369072 | 8.712866 |
| H | 19.780079 | 5.791879 | 10.532373 |
| H | 21.444671 | 5.214287 | 10.680706 |
| H | 21.107995 | 6.951081 | 10.717327 |
| H | 21.033084 | 3.851500 | 8.656326 |
| H | 19.429560 | 4.539954 | 8.357388 |
| H | 20.646992 | 4.585130 | 7.088935 |
| H | 22.945648 | 7.025721 | 8.937903 |
| H | 23.028121 | 5.264463 | 8.864212 |
| H | 22.761177 | 6.194672 | 7.387290 |
| H | 21.705343 | 9.203720 | 5.989355 |
| H | 22.391624 | 10.373665 | 7.115135 |
| H | 22.778500 | 8.655481 | 7.286154 |
| H | 20.150271 | 11.243445 | 7.451231 |
| H | 19.281344 | 10.000020 | 6.532958 |
| H | 18.868906 | 10.304215 | 8.227685 |
| H | 22.022804 | 8.933762 | 9.744005 |
| H | 21.602854 | 10.613641 | 9.396122 |
| H | 20.397163 | 9.539884 | 10.112871 |

---

Calculated energies and coordinates of **TS3b**

Electronic energy ... -3272.21228940 Eh
Total Enthalpy ... -3271.00760119 Eh
Final Gibbs free energy ... -3271.15827147 Eh

CARTESIAN COORDINATES (ANGSTROEM)

| | | | |
|---|---|---|---|
| C | -0.249168 | -1.647109 | -0.272700 |
| C | -2.583145 | 3.824626 | -0.599587 |
| C | -4.694337 | 2.478814 | -0.512551 |
| C | -0.818270 | 2.123130 | -2.611278 |
| C | -3.084481 | 0.046956 | -3.097794 |
| C | -2.840006 | 0.584646 | -4.521201 |
| C | -4.593920 | -0.165991 | -2.909690 |
| C | -2.391078 | -1.319570 | -2.985042 |
| C | -3.450002 | 2.794643 | -1.351855 |
| C | -3.938998 | 3.464189 | -2.652797 |
| C | -4.168366 | -0.899392 | 0.808789 |
| C | -2.309151 | -2.533345 | 2.523211 |
| C | -2.668849 | -3.555063 | 1.424173 |
| C | -3.277214 | -2.818038 | 3.689869 |
| C | -0.875173 | -2.781183 | 3.008886 |
| C | -2.949345 | 0.612393 | 3.241011 |
| C | -4.470680 | 0.645177 | 3.498466 |
| C | -2.533458 | 2.000411 | 2.734399 |
| C | -2.264078 | 0.377200 | 4.599501 |
| C | 4.466005 | -0.698314 | -1.199918 |
| C | 2.646371 | -2.877102 | -2.306614 |
| C | 3.896608 | -3.322504 | -3.093314 |
| C | 1.415593 | -3.298619 | -3.126068 |
| C | 2.667821 | -3.639932 | -0.968528 |
| C | 2.653282 | 0.175329 | -3.491275 |
| C | 3.897078 | 0.020616 | -4.389112 |
| C | 1.419690 | -0.130533 | -4.357095 |
| C | 2.643835 | 1.637228 | -3.010276 |
| C | 0.943093 | 0.397517 | 3.248444 |
| C | 3.758221 | -0.347874 | 4.324547 |
| C | 3.791433 | -0.539628 | 2.793479 |
| C | 3.540490 | -2.026712 | 2.499360 |
| C | 5.200392 | -0.156190 | 2.318780 |
| C | 2.717398 | 2.367697 | 1.731214 |
| C | 3.694139 | 2.608480 | 0.574907 |
| C | 1.394119 | 3.082333 | 1.405035 |
| C | 3.296116 | 2.992559 | 3.012399 |
| O | -0.392479 | -2.791932 | -0.612558 |
| Si | -1.146228 | 0.043861 | -0.022430 |
| Si | 1.256273 | -0.367432 | -0.018118 |
| Si | -2.593609 | -0.727631 | 1.852253 |
| Si | -2.306208 | 1.280668 | -1.808854 |
| Si | 2.741188 | -0.971522 | -1.929614 |
| Si | 2.343112 | 0.484839 | 1.995512 |
| H | -3.218164 | 4.634425 | -0.213092 |
| H | -2.047181 | 3.388598 | 0.250899 |
| H | -1.838493 | 4.278359 | -1.260382 |
| H | -4.440485 | 2.101362 | 0.479791 |
| H | -5.279694 | 3.397858 | -0.365123 |
| H | -5.349000 | 1.748069 | -0.996787 |
| H | -0.143735 | 1.395183 | -3.056664 |
| H | -1.120182 | 2.821951 | -3.399061 |
| H | -0.247397 | 2.678803 | -1.861197 |
| H | -3.291866 | -0.102456 | -5.251086 |
| H | -3.283438 | 1.572737 | -4.681706 |
| H | -1.771881 | 0.652393 | -4.753121 |
| H | -5.169952 | 0.746835 | -3.089062 |
| H | -4.947652 | -0.918149 | -3.629481 |
| H | -4.840101 | -0.532505 | -1.908784 |
| H | -2.741632 | -1.983423 | -3.788595 |
| H | -1.302620 | -1.238702 | -3.079980 |
| H | -2.592793 | -1.813216 | -2.029137 |
| H | -4.459660 | 4.399452 | -2.400230 |
| H | -3.118550 | 3.723854 | -3.328861 |
| H | -4.648534 | 2.837217 | -3.201087 |
| H | -3.976418 | -1.560217 | -0.041239 |
| H | -4.972243 | -1.347700 | 1.405428 |
| H | -4.530417 | 0.052514 | 0.420821 |
| H | -2.405312 | -4.561187 | 1.779463 |
| H | -3.744489 | -3.555072 | 1.221901 |
| H | -2.142519 | -3.391623 | 0.484154 |
| H | -4.320605 | -2.618156 | 3.420164 |
| H | -3.212045 | -3.885167 | 3.946396 |
| H | -3.039753 | -2.254392 | 4.593353 |
| H | -0.759767 | -3.830129 | 3.316710 |
| H | -0.134556 | -2.581425 | 2.226949 |
| H | -0.636753 | -2.157067 | 3.876594 |

| | | | |
|---|---|---|---|
| H | -4.685028 | 1.379653 | 4.287948 |
| H | -5.038756 | 0.941326 | 2.611106 |
| H | -4.853192 | -0.322715 | 3.839295 |
| H | -3.005933 | 2.241463 | 1.780366 |
| H | -2.839848 | 2.770831 | 3.456652 |
| H | -1.451613 | 2.085368 | 2.594925 |
| H | -2.408894 | 1.269323 | 5.225586 |
| H | -2.703667 | -0.461721 | 5.142253 |
| H | -1.190107 | 0.206873 | 4.519906 |
| H | 4.660769 | -1.396237 | -0.382310 |
| H | 5.228563 | -0.871983 | -1.966607 |
| H | 4.597090 | 0.318317 | -0.821597 |
| H | 3.870298 | -4.416192 | -3.204703 |
| H | 3.930185 | -2.899574 | -4.101170 |
| H | 4.830979 | -3.070611 | -2.581553 |
| H | 1.484961 | -2.949412 | -4.159936 |
| H | 1.360253 | -4.396018 | -3.157100 |
| H | 0.475333 | -2.942416 | -2.703749 |
| H | 2.585613 | -4.718320 | -1.165508 |
| H | 3.612870 | -3.483172 | -0.436695 |
| H | 1.840427 | -3.364902 | -0.310539 |
| H | 3.994006 | -0.985107 | -4.805225 |
| H | 3.811216 | 0.716098 | -5.236752 |
| H | 4.823828 | 0.265965 | -3.861363 |
| H | 1.201105 | 0.711207 | -5.029400 |
| H | 1.592876 | -1.011529 | -4.980974 |
| H | 0.520911 | -0.333541 | -3.767950 |
| H | 3.608966 | 1.904086 | -2.565596 |
| H | 2.478802 | 2.312506 | -3.862112 |
| H | 1.865361 | 1.838137 | -2.270126 |
| H | 0.856306 | -0.610688 | 3.659735 |
| H | 1.095128 | 1.094369 | 4.080668 |
| H | -0.003595 | 0.635875 | 2.761201 |
| H | 2.830252 | -0.715893 | 4.770630 |
| H | 4.585690 | -0.916234 | 4.772987 |
| H | 3.883012 | 0.699233 | 4.619278 |
| H | 4.288297 | -2.646939 | 3.014685 |
| H | 2.549982 | -2.351148 | 2.842325 |
| H | 3.599724 | -2.244969 | 1.429615 |
| H | 5.465509 | 0.864310 | 2.613055 |
| H | 5.936435 | -0.827657 | 2.784104 |
| H | 5.323600 | -0.236124 | 1.237964 |
| H | 3.295133 | 2.210406 | -0.362521 |
| H | 3.855593 | 3.687157 | 0.433714 |
| H | 4.671676 | 2.150225 | 0.756596 |
| H | 1.585343 | 4.148189 | 1.213859 |
| H | 0.909404 | 2.661568 | 0.516499 |
| H | 0.682955 | 3.020629 | 2.236029 |
| H | 4.281438 | 2.587152 | 3.261972 |
| H | 3.415491 | 4.076475 | 2.869180 |
| H | 2.639123 | 2.849643 | 3.877441 |

Calculated energies and coordinates of **INT3b**

Electronic energy ... -3272.21513607 Eh
Total Enthalpy ... -3271.00939740 Eh
Final Gibbs free energy ... -3271.16125029 Eh

CARTESIAN COORDINATES (ANGSTROEM)

| | | | |
|---|---|---|---|
| C | 17.102626 | 5.497074 | 5.404599 |
| C | 15.179137 | 10.938783 | 5.750237 |
| C | 12.974838 | 9.725882 | 5.745424 |
| C | 16.683344 | 9.358442 | 3.332421 |
| C | 14.211033 | 7.507039 | 2.920822 |
| C | 14.400386 | 8.133282 | 1.525045 |
| C | 12.705064 | 7.435699 | 3.215665 |
| C | 14.745464 | 6.068904 | 2.872987 |
| C | 14.206641 | 10.093362 | 4.904408 |
| C | 13.718057 | 10.977046 | 3.739071 |
| C | 13.507325 | 5.948501 | 6.737825 |
| C | 15.536394 | 4.413497 | 8.427156 |
| C | 15.594881 | 3.363024 | 7.304247 |
| C | 14.399172 | 3.970083 | 9.374990 |
| C | 16.874202 | 4.363340 | 9.177017 |
| C | 14.624513 | 7.432556 | 9.238218 |
| C | 13.097096 | 7.358145 | 9.454292 |
| C | 14.963580 | 8.878825 | 8.851666 |
| C | 15.292154 | 7.157428 | 10.598588 |
| C | 21.953800 | 6.399846 | 4.688910 |
| C | 20.318063 | 3.882193 | 4.063015 |
| C | 21.762875 | 3.484830 | 3.693801 |
| C | 19.385968 | 3.247643 | 3.016913 |
| C | 19.996265 | 3.261410 | 5.431998 |
| C | 19.888392 | 6.678801 | 2.459980 |
| C | 20.875245 | 6.197641 | 1.382551 |
| C | 18.455367 | 6.404412 | 1.994865 |
| C | 20.082581 | 8.193500 | 2.635777 |
| C | 18.485775 | 7.583029 | 9.211158 |
| C | 21.395117 | 7.193689 | 10.209910 |
| C | 21.398895 | 6.877762 | 8.699512 |
| C | 21.312729 | 5.353926 | 8.535305 |
| C | 22.739848 | 7.366992 | 8.133111 |
| C | 20.020441 | 9.555091 | 7.436928 |
| C | 20.920193 | 9.776004 | 6.214462 |
| C | 18.632933 | 10.136882 | 7.122189 |
| C | 20.598325 | 10.337614 | 8.629974 |
| O | 16.987252 | 4.535047 | 4.696297 |
| Si | 16.387058 | 7.170668 | 5.896442 |
| Si | 18.753278 | 6.557006 | 5.984850 |
| Si | 15.081472 | 6.193464 | 7.772790 |
| Si | 15.204729 | 8.571633 | 4.218984 |
| Si | 20.225295 | 5.833992 | 4.160095 |
| Si | 19.819257 | 7.675210 | 7.886624 |
| H | 14.637548 | 11.768174 | 6.227012 |
| H | 15.663425 | 10.360260 | 6.542979 |
| H | 15.970419 | 11.374792 | 5.131819 |
| H | 13.203999 | 9.026356 | 6.551701 |
| H | 12.556652 | 10.630978 | 6.208926 |
| H | 12.186577 | 9.274485 | 5.138056 |
| H | 17.224059 | 8.637598 | 2.719452 |
| H | 16.355890 | 10.167126 | 2.669613 |
| H | 17.396322 | 9.776541 | 4.049850 |
| H | 13.819426 | 7.559212 | 0.788859 |
| H | 14.055966 | 9.172086 | 1.478610 |
| H | 15.447321 | 8.110186 | 1.206676 |
| H | 12.217396 | 8.412340 | 3.144369 |
| H | 12.224938 | 6.779532 | 2.475507 |
| H | 12.494560 | 7.017183 | 4.204911 |
| H | 14.214824 | 5.505189 | 2.091985 |
| H | 15.811596 | 6.024749 | 2.639598 |
| H | 14.603653 | 5.541473 | 3.820344 |
| H | 13.233937 | 11.876496 | 4.146970 |
| H | 14.537142 | 11.313949 | 3.095977 |
| H | 12.978891 | 10.467747 | 3.113190 |
| H | 13.719243 | 5.326778 | 5.863102 |
| H | 12.719618 | 5.457424 | 7.319672 |
| H | 13.105858 | 6.898314 | 6.375613 |
| H | 15.587840 | 2.363695 | 7.761825 |
| H | 14.741174 | 3.414168 | 6.621043 |
| H | 16.506294 | 3.441522 | 6.714328 |
| H | 13.441636 | 3.887308 | 8.849552 |

| | | | |
|---|---|---|---|
| H | 14.639411 | 2.972362 | 9.768195 |
| H | 14.260003 | 4.630310 | 10.232834 |
| H | 17.080213 | 3.330967 | 9.494426 |
| H | 17.697848 | 4.681650 | 8.526447 |
| H | 16.876529 | 4.990842 | 10.072225 |
| H | 12.821061 | 8.024648 | 10.283545 |
| H | 12.536231 | 7.683063 | 8.572581 |
| H | 12.758358 | 6.350312 | 9.716078 |
| H | 14.501379 | 9.167325 | 7.907748 |
| H | 14.590448 | 9.568304 | 9.622755 |
| H | 16.040741 | 9.040947 | 8.757419 |
| H | 14.955324 | 7.924694 | 11.310450 |
| H | 15.018104 | 6.189388 | 11.023152 |
| H | 16.380430 | 7.213440 | 10.554932 |
| H | 22.312713 | 5.822008 | 5.544513 |
| H | 22.669893 | 6.248453 | 3.873014 |
| H | 21.969281 | 7.457957 | 4.959985 |
| H | 21.816326 | 2.391393 | 3.593377 |
| H | 22.087483 | 3.916373 | 2.740490 |
| H | 22.485258 | 3.777992 | 4.461375 |
| H | 19.645539 | 3.546773 | 1.996720 |
| H | 19.493824 | 2.154235 | 3.066178 |
| H | 18.337037 | 3.484093 | 3.198324 |
| H | 20.122967 | 2.170201 | 5.378787 |
| H | 20.667693 | 3.626644 | 6.215881 |
| H | 18.966385 | 3.466213 | 5.737988 |
| H | 20.764306 | 5.132983 | 1.158428 |
| H | 20.692300 | 6.748868 | 0.448674 |
| H | 21.917309 | 6.379869 | 1.667746 |
| H | 18.215770 | 7.003817 | 1.103623 |
| H | 18.294523 | 5.353128 | 1.743625 |
| H | 17.740153 | 6.663033 | 2.780648 |
| H | 21.122929 | 8.444619 | 2.871060 |
| H | 19.818221 | 8.717497 | 1.705652 |
| H | 19.447800 | 8.595377 | 3.431975 |
| H | 18.523115 | 6.625373 | 9.735877 |
| H | 18.577543 | 8.384251 | 9.953433 |
| H | 17.505201 | 7.659061 | 8.738261 |
| H | 20.537082 | 6.753738 | 10.725605 |
| H | 22.302889 | 6.771666 | 10.664629 |
| H | 21.397100 | 8.269375 | 10.413331 |
| H | 22.105894 | 4.862651 | 9.117348 |
| H | 20.350681 | 4.952740 | 8.878607 |
| H | 21.436083 | 5.061701 | 7.489240 |
| H | 22.909694 | 8.426773 | 8.345255 |
| H | 23.560310 | 6.807860 | 8.605887 |
| H | 22.824314 | 7.222772 | 7.054817 |
| H | 20.518222 | 9.271245 | 5.329838 |
| H | 20.982015 | 10.849238 | 5.982953 |
| H | 21.940676 | 9.416588 | 6.379925 |
| H | 18.726703 | 11.196480 | 6.842675 |
| H | 18.152734 | 9.610016 | 6.291372 |
| H | 17.961057 | 10.085487 | 7.986160 |
| H | 21.628296 | 10.050585 | 8.860385 |
| H | 20.606488 | 11.410796 | 8.389383 |
| H | 19.998161 | 10.214424 | 9.538325 |

Calculated energies and coordinates of **TS4b**

Electronic energy ... -3272.20155117 Eh
Total Enthalpy ... -3270.99812424 Eh
Final Gibbs free energy ... -3271.14989265 Eh

CARTESIAN COORDINATES (ANGSTROEM)

| | | | |
|---|---|---|---|
| C | 17.288195 | 6.051287 | 5.279771 |
| C | 15.386148 | 10.869237 | 5.560861 |
| C | 13.085783 | 9.945594 | 5.741650 |
| C | 16.527866 | 8.847800 | 3.129064 |
| C | 13.812519 | 7.394165 | 3.050642 |
| C | 13.942900 | 7.894589 | 1.597687 |
| C | 12.334589 | 7.490256 | 3.454433 |
| C | 14.240274 | 5.918616 | 3.071261 |
| C | 14.279778 | 10.123481 | 4.794988 |
| C | 13.841560 | 10.989442 | 3.601643 |
| C | 13.104363 | 6.260864 | 6.996598 |
| C | 15.317591 | 4.494264 | 8.143237 |
| C | 15.171120 | 3.733124 | 6.812091 |
| C | 14.381473 | 3.819584 | 9.164883 |
| C | 16.768289 | 4.362829 | 8.624173 |
| C | 14.494348 | 7.370496 | 9.510152 |
| C | 13.024750 | 7.176060 | 9.940438 |
| C | 14.713033 | 8.868071 | 9.239989 |
| C | 15.371165 | 6.963823 | 10.705848 |
| C | 22.307653 | 6.234319 | 4.726129 |
| C | 20.515544 | 3.829391 | 4.098228 |
| C | 21.445465 | 3.328728 | 2.978098 |
| C | 19.122503 | 3.225863 | 3.877162 |
| C | 21.043551 | 3.287892 | 5.437689 |
| C | 20.345394 | 6.656300 | 2.427919 |
| C | 21.632858 | 6.541622 | 1.588716 |
| C | 19.178033 | 6.087392 | 1.608182 |
| C | 20.107600 | 8.152880 | 2.685680 |
| C | 18.365116 | 7.627957 | 9.091852 |
| C | 21.213100 | 7.249250 | 10.297665 |
| C | 21.298720 | 6.902940 | 8.797320 |
| C | 21.195231 | 5.376959 | 8.656529 |
| C | 22.675725 | 7.357142 | 8.291988 |
| C | 20.049918 | 9.565450 | 7.385643 |
| C | 21.015350 | 9.712728 | 6.202100 |
| C | 18.696604 | 10.170576 | 6.987002 |
| C | 20.585899 | 10.363714 | 8.586978 |
| O | 17.220255 | 5.521175 | 4.161907 |
| Si | 16.052180 | 7.261334 | 6.018868 |
| Si | 19.044761 | 6.511506 | 5.900255 |
| Si | 14.787897 | 6.336910 | 7.876054 |
| Si | 15.009262 | 8.441918 | 4.177199 |
| Si | 20.560483 | 5.778084 | 4.144909 |
| Si | 19.789481 | 7.704846 | 7.865871 |
| H | 15.003302 | 11.834214 | 5.923383 |
| H | 15.728020 | 10.299331 | 6.433302 |
| H | 16.259883 | 11.074555 | 4.933654 |
| H | 13.343155 | 9.296374 | 6.583055 |
| H | 12.786007 | 10.919070 | 6.156268 |
| H | 12.213845 | 9.516244 | 5.241978 |
| H | 16.965088 | 7.927292 | 2.730838 |
| H | 16.281134 | 9.498910 | 2.283469 |
| H | 17.294035 | 9.346195 | 3.729448 |
| H | 13.246522 | 7.331358 | 0.959755 |
| H | 13.698701 | 8.957175 | 1.491277 |
| H | 14.950693 | 7.736163 | 1.202324 |
| H | 11.934813 | 8.499808 | 3.315089 |
| H | 11.742055 | 6.817110 | 2.818178 |
| H | 12.160797 | 7.194550 | 4.493015 |
| H | 13.608325 | 5.343237 | 2.378968 |
| H | 15.284543 | 5.787194 | 2.776150 |
| H | 14.138641 | 5.469651 | 4.065967 |
| H | 13.501899 | 11.971396 | 3.962074 |
| H | 14.661059 | 11.165872 | 2.896199 |
| H | 13.008857 | 10.539490 | 3.051330 |
| H | 13.195283 | 5.832407 | 5.995807 |
| H | 12.415269 | 5.627719 | 7.569035 |

| | | | |
|---|---|---|---|
| H | 12.644671 | 7.247479 | 6.896400 |
| H | 15.485061 | 2.689639 | 6.957251 |
| H | 14.132851 | 3.714077 | 6.464185 |
| H | 15.790956 | 4.154579 | 6.015514 |
| H | 13.331020 | 3.870255 | 8.856569 |
| H | 14.643484 | 2.755004 | 9.246180 |
| H | 14.463394 | 4.250339 | 10.166392 |
| H | 17.039296 | 3.300414 | 8.705745 |
| H | 17.468038 | 4.825783 | 7.915951 |
| H | 16.918988 | 4.819018 | 9.607858 |
| H | 12.849561 | 7.724226 | 10.876784 |
| H | 12.317943 | 7.559086 | 9.198897 |
| H | 12.783818 | 6.123807 | 10.127916 |
| H | 14.044253 | 9.247057 | 8.461518 |
| H | 14.506600 | 9.444002 | 10.153523 |
| H | 15.740550 | 9.087738 | 8.932043 |
| H | 15.118799 | 7.603490 | 11.563672 |
| H | 15.200409 | 5.929874 | 11.017419 |
| H | 16.435487 | 7.089693 | 10.509987 |
| H | 22.513541 | 5.843336 | 5.724704 |
| H | 23.062464 | 5.815583 | 4.050943 |
| H | 22.449894 | 7.320066 | 4.752445 |
| H | 21.536096 | 2.234790 | 3.045738 |
| H | 21.045648 | 3.558764 | 1.985730 |
| H | 22.457170 | 3.744373 | 3.047494 |
| H | 18.646185 | 3.588479 | 2.963782 |
| H | 19.214651 | 2.131784 | 3.807050 |
| H | 18.448374 | 3.459351 | 4.705209 |
| H | 21.034195 | 2.188701 | 5.414952 |
| H | 22.072186 | 3.601629 | 5.644981 |
| H | 20.407775 | 3.600582 | 6.274649 |
| H | 21.892353 | 5.506731 | 1.349340 |
| H | 21.485022 | 7.071102 | 0.636345 |
| H | 22.491769 | 7.002284 | 2.087188 |
| H | 19.039343 | 6.690107 | 0.698700 |
| H | 19.376922 | 5.058798 | 1.288394 |
| H | 18.241870 | 6.081360 | 2.171333 |
| H | 20.977406 | 8.621138 | 3.160734 |
| H | 19.933786 | 8.676092 | 1.734011 |
| H | 19.237100 | 8.325225 | 3.324304 |
| H | 18.356125 | 6.658746 | 9.596702 |
| H | 18.443818 | 8.410787 | 9.855139 |
| H | 17.407840 | 7.743927 | 8.577695 |
| H | 20.316595 | 6.835268 | 10.768057 |
| H | 22.083259 | 6.819283 | 10.814055 |
| H | 21.224156 | 8.328373 | 10.481129 |
| H | 21.934341 | 4.884589 | 9.305214 |
| H | 20.203387 | 5.000396 | 8.938489 |
| H | 21.390090 | 5.057335 | 7.629096 |
| H | 22.860893 | 8.413882 | 8.506055 |
| H | 23.461764 | 6.781097 | 8.801418 |
| H | 22.802820 | 7.207021 | 7.218087 |
| H | 20.633880 | 9.187256 | 5.320215 |
| H | 21.126950 | 10.773397 | 5.934291 |
| H | 22.012214 | 9.320636 | 6.426582 |
| H | 18.829350 | 11.224042 | 6.700741 |
| H | 18.248096 | 9.642807 | 6.138984 |
| H | 17.976163 | 10.141921 | 7.813089 |
| H | 21.593596 | 10.057329 | 8.881117 |
| H | 20.635246 | 11.429587 | 8.321368 |
| H | 19.933660 | 10.279187 | 9.463686 |

Calculated energies and coordinates of **2b**

Electronic energy ... -3272.25671445 Eh

Total Enthalpy ... -3271.05317265 Eh
Final Gibbs free energy ... -3271.20730907 Eh

CARTESIAN COORDINATES (ANGSTROEM)

| | | | |
|---|---|---|---|
| C | 17.560035 | 6.836351 | 5.743927 |
| C | 15.448174 | 10.919764 | 5.980054 |
| C | 13.185316 | 9.922675 | 6.280285 |
| C | 16.474064 | 9.192331 | 3.359971 |
| C | 13.696129 | 7.897036 | 3.081449 |
| C | 13.738650 | 8.653490 | 1.741119 |
| C | 12.248252 | 7.870811 | 3.588966 |
| C | 14.150394 | 6.447849 | 2.833362 |
| C | 14.262160 | 10.273913 | 5.243840 |
| C | 13.689856 | 11.297609 | 4.249382 |
| C | 12.689653 | 5.861915 | 7.025901 |
| C | 15.020344 | 3.811259 | 7.018746 |
| C | 14.592496 | 3.488366 | 5.575307 |
| C | 14.286274 | 2.855148 | 7.973613 |
| C | 16.532848 | 3.563244 | 7.131487 |
| C | 14.845772 | 6.259688 | 9.190781 |
| C | 13.814313 | 5.677522 | 10.172299 |
| C | 14.700513 | 7.789975 | 9.216152 |
| C | 16.261007 | 5.889891 | 9.656530 |
| C | 22.480782 | 6.024144 | 4.991460 |
| C | 20.398509 | 3.777900 | 4.910664 |
| C | 21.587405 | 2.956636 | 4.378456 |
| C | 19.101967 | 3.254405 | 4.275431 |
| C | 20.324571 | 3.563417 | 6.431746 |
| C | 20.375224 | 6.215887 | 2.733590 |
| C | 21.155548 | 5.325675 | 1.752910 |
| C | 18.888081 | 6.198985 | 2.355360 |
| C | 20.877901 | 7.662654 | 2.588044 |
| C | 18.230220 | 9.065831 | 8.500110 |
| C | 20.895935 | 8.537235 | 10.367175 |
| C | 20.924053 | 7.692116 | 9.080833 |
| C | 20.316395 | 6.317837 | 9.406010 |
| C | 22.379956 | 7.489360 | 8.642693 |
| C | 20.639173 | 10.098782 | 6.890842 |
| C | 21.786495 | 9.745353 | 5.935012 |
| C | 19.530757 | 10.785960 | 6.074366 |
| C | 21.156324 | 11.092083 | 7.943777 |
| O | 16.819197 | 6.074247 | 4.855781 |
| Si | 15.734241 | 7.048879 | 5.854101 |
| Si | 19.294659 | 6.987792 | 5.992220 |
| Si | 14.536230 | 5.645951 | 7.387929 |
| Si | 14.925747 | 8.714678 | 4.330865 |
| Si | 20.667338 | 5.664420 | 4.565326 |
| Si | 19.855631 | 8.524703 | 7.701025 |
| H | 15.108501 | 11.809203 | 6.529710 |
| H | 15.892850 | 10.233756 | 6.710449 |
| H | 16.240228 | 11.234655 | 5.292248 |
| H | 13.557571 | 9.197226 | 7.010920 |
| H | 12.889818 | 10.824235 | 6.836440 |
| H | 12.282955 | 9.507556 | 5.822360 |
| H | 16.733960 | 8.409044 | 2.641820 |
| H | 16.315760 | 10.122540 | 2.801522 |
| H | 17.334758 | 9.323940 | 4.021859 |
| H | 13.028431 | 8.193421 | 1.038910 |
| H | 13.460871 | 9.707591 | 1.845540 |
| H | 14.730087 | 8.610459 | 1.279089 |
| H | 11.816784 | 8.874715 | 3.655702 |
| H | 11.621741 | 7.292362 | 2.894587 |
| H | 12.166222 | 7.401356 | 4.574911 |
| H | 13.549150 | 6.000625 | 2.028579 |
| H | 15.205020 | 6.380032 | 2.542520 |
| H | 14.014140 | 5.827314 | 3.726749 |

| | | | |
|---|---|---|---|
| H | 13.405294 | 12.215075 | 4.784567 |
| H | 14.419304 | 11.580624 | 3.481916 |
| H | 12.793491 | 10.923089 | 3.745177 |
| H | 12.450877 | 5.623414 | 5.985491 |
| H | 12.087904 | 5.203805 | 7.663017 |
| H | 12.365525 | 6.891818 | 7.212095 |
| H | 14.826757 | 2.437537 | 5.353476 |
| H | 13.516321 | 3.622609 | 5.419250 |
| H | 15.134059 | 4.102831 | 4.848201 |
| H | 13.199050 | 2.995410 | 7.956804 |
| H | 14.487235 | 1.815805 | 7.675878 |
| H | 14.631586 | 2.967746 | 9.006497 |
| H | 16.765600 | 2.546780 | 6.782354 |
| H | 17.103568 | 4.263330 | 6.517223 |
| H | 16.892777 | 3.646009 | 8.159981 |
| H | 14.018875 | 6.053252 | 11.185280 |
| H | 12.792661 | 5.975981 | 9.913248 |
| H | 13.846091 | 4.584901 | 10.216034 |
| H | 13.687134 | 8.107983 | 8.947435 |
| H | 14.902181 | 8.167506 | 10.229149 |
| H | 15.410035 | 8.284333 | 8.542310 |
| H | 16.499102 | 6.413899 | 10.594010 |
| H | 16.353507 | 4.816866 | 9.851011 |
| H | 17.017797 | 6.162714 | 8.911056 |
| H | 22.774379 | 5.513460 | 5.914391 |
| H | 23.138919 | 5.659005 | 4.194071 |
| H | 22.677087 | 7.091507 | 5.122735 |
| H | 21.418184 | 1.890594 | 4.589165 |
| H | 21.719925 | 3.058928 | 3.297228 |
| H | 22.528685 | 3.239372 | 4.861911 |
| H | 19.170162 | 3.231088 | 3.182709 |
| H | 18.911118 | 2.225338 | 4.613409 |
| H | 18.232909 | 3.863296 | 4.546524 |
| H | 20.239786 | 2.489399 | 6.653347 |
| H | 21.222469 | 3.932704 | 6.942963 |
| H | 19.456604 | 4.067007 | 6.869423 |
| H | 20.765078 | 4.303302 | 1.732158 |
| H | 21.062788 | 5.730493 | 0.734593 |
| H | 22.225085 | 5.279071 | 1.989422 |
| H | 18.762928 | 6.581138 | 1.331008 |
| H | 18.454273 | 5.196053 | 2.392394 |
| H | 18.304589 | 6.831553 | 3.029441 |
| H | 21.944101 | 7.762384 | 2.821432 |
| H | 20.732254 | 8.000818 | 1.551996 |
| H | 20.316087 | 8.345156 | 3.237649 |
| H | 17.879613 | 8.335186 | 9.234188 |
| H | 18.346535 | 10.028641 | 9.011730 |
| H | 17.454348 | 9.174000 | 7.738143 |
| H | 19.876730 | 8.663717 | 10.748426 |
| H | 21.479570 | 8.033060 | 11.151136 |
| H | 21.328512 | 9.531722 | 10.225434 |
| H | 20.875614 | 5.848678 | 10.228703 |
| H | 19.269726 | 6.395311 | 9.721654 |
| H | 20.351512 | 5.642354 | 8.545005 |
| H | 22.906274 | 8.440595 | 8.511484 |
| H | 22.926460 | 6.915579 | 9.405281 |
| H | 22.446034 | 6.933440 | 7.701063 |
| H | 21.447365 | 9.068765 | 5.143691 |
| H | 22.167733 | 10.655650 | 5.449545 |
| H | 22.626907 | 9.270506 | 6.451049 |
| H | 19.946649 | 11.645531 | 5.529429 |
| H | 19.093425 | 10.100329 | 5.338086 |
| H | 18.720630 | 11.156727 | 6.711868 |
| H | 22.028521 | 10.702464 | 8.478620 |
| H | 21.465590 | 12.026061 | 7.452425 |
| H | 20.390075 | 11.351408 | 8.683692 |

Calculated energies and coordinates of **TS4a**

Electronic energy ... -3248.19886678 Eh
Total Enthalpy ... -3246.94772409 Eh
Final Gibbs free energy ... -3247.10347285 Eh

CARTESIAN COORDINATES (ANGSTROEM)

| | | | |
|---|---|---|---|
| Si | 6.758825 | 11.611869 | 8.008991 |
| Si | 6.144544 | 12.361265 | 10.101271 |
| Si | 3.859825 | 13.171635 | 10.168996 |
| Si | 7.821041 | 13.933950 | 10.858558 |
| O | 8.194606 | 10.029632 | 11.039079 |
| O | 6.107960 | 9.637278 | 11.963268 |
| N | 8.217968 | 11.996891 | 7.295967 |
| N | 10.030857 | 10.856867 | 6.148582 |
| N | 9.332944 | 12.785045 | 5.312461 |
| N | 5.858012 | 10.452679 | 7.235056 |
| N | 5.169347 | 9.377254 | 5.178655 |
| N | 5.252929 | 8.132078 | 7.005863 |
| C | 9.094694 | 11.871627 | 6.348721 |
| C | 10.776099 | 11.133402 | 5.010230 |
| C | 10.364498 | 12.312863 | 4.513765 |
| C | 10.123855 | 9.564552 | 6.888477 |
| C | 11.436068 | 8.862038 | 6.518298 |
| C | 8.949415 | 8.686225 | 6.450916 |
| C | 10.134323 | 9.810786 | 8.398217 |
| C | 8.857205 | 14.200356 | 5.260529 |
| C | 7.330933 | 14.283409 | 5.364070 |
| C | 9.557299 | 14.949579 | 6.396672 |
| C | 9.257436 | 14.824575 | 3.918732 |
| C | 5.476898 | 9.431756 | 6.542023 |
| C | 4.797080 | 8.084550 | 4.839855 |
| C | 4.845883 | 7.329234 | 5.952667 |
| C | 5.303057 | 10.490447 | 4.198279 |
| C | 6.780603 | 10.868003 | 4.085543 |
| C | 4.449802 | 11.677622 | 4.650282 |
| C | 4.802002 | 10.030546 | 2.825768 |
| C | 5.279085 | 7.690777 | 8.433604 |
| C | 6.699605 | 7.789527 | 8.991300 |
| C | 4.307905 | 8.555994 | 9.231137 |
| C | 4.826902 | 6.230550 | 8.525242 |
| C | 3.883266 | 14.951650 | 10.835299 |
| C | 2.863148 | 12.179818 | 11.519843 |
| C | 3.542876 | 12.446806 | 12.873983 |
| C | 1.396461 | 12.628777 | 11.621351 |
| C | 2.889684 | 10.666083 | 11.294082 |
| C | 2.942912 | 13.301121 | 8.452379 |
| C | 1.664798 | 14.153183 | 8.568866 |
| C | 2.569840 | 11.918972 | 7.900892 |
| C | 3.852649 | 14.013110 | 7.440218 |
| C | 9.446608 | 13.138211 | 10.282450 |
| C | 7.930287 | 13.951957 | 12.807563 |
| C | 6.690398 | 14.545926 | 13.489616 |
| C | 9.167102 | 14.756277 | 13.248918 |
| C | 8.094954 | 12.516960 | 13.332688 |
| C | 7.808615 | 15.757038 | 10.122274 |
| C | 7.081722 | 15.714665 | 8.772071 |
| C | 7.123079 | 16.828705 | 10.983084 |
| C | 9.258300 | 16.225328 | 9.887581 |
| C | 7.061117 | 10.036795 | 11.385836 |
| H | 11.545996 | 10.480087 | 4.640970 |
| H | 10.722397 | 12.838535 | 3.646877 |
| H | 11.453489 | 8.521905 | 5.478150 |
| H | 11.528809 | 7.975838 | 7.151447 |

| | | | |
|---|---|---|---|
| H | 12.304978 | 9.501051 | 6.707007 |
| H | 8.981058 | 8.523966 | 5.367891 |
| H | 7.997329 | 9.154143 | 6.705643 |
| H | 9.003173 | 7.713765 | 6.948232 |
| H | 10.991674 | 10.431535 | 8.679207 |
| H | 10.215608 | 8.850994 | 8.917164 |
| H | 9.233415 | 10.305639 | 8.750537 |
| H | 7.032402 | 15.336096 | 5.341568 |
| H | 6.956891 | 13.848185 | 6.287818 |
| H | 6.860596 | 13.773513 | 4.518261 |
| H | 10.641287 | 14.945032 | 6.237894 |
| H | 9.344592 | 14.469523 | 7.352257 |
| H | 9.212244 | 15.986676 | 6.449163 |
| H | 8.863532 | 14.251044 | 3.072333 |
| H | 10.340535 | 14.934139 | 3.810827 |
| H | 8.824430 | 15.827499 | 3.874409 |
| H | 4.517359 | 7.795192 | 3.843027 |
| H | 4.617023 | 6.284849 | 6.065469 |
| H | 7.375950 | 10.004070 | 3.771668 |
| H | 7.155498 | 11.226071 | 5.042566 |
| H | 6.910149 | 11.662380 | 3.343404 |
| H | 4.542762 | 12.492346 | 3.924636 |
| H | 4.763207 | 12.045063 | 5.625811 |
| H | 3.395816 | 11.388683 | 4.713522 |
| H | 4.874265 | 10.880688 | 2.141914 |
| H | 3.753338 | 9.717312 | 2.855731 |
| H | 5.411890 | 9.221634 | 2.411048 |
| H | 7.369784 | 7.105644 | 8.461834 |
| H | 6.690708 | 7.515229 | 10.050476 |
| H | 7.093709 | 8.803141 | 8.914232 |
| H | 4.621976 | 9.600023 | 9.238026 |
| H | 4.276882 | 8.211475 | 10.267966 |
| H | 3.300041 | 8.491516 | 8.806588 |
| H | 3.797923 | 6.093614 | 8.177120 |
| H | 4.861056 | 5.941382 | 9.578893 |
| H | 5.491305 | 5.554294 | 7.976584 |
| H | 4.142331 | 15.675061 | 10.056877 |
| H | 4.601158 | 15.067023 | 11.650271 |
| H | 2.895721 | 15.225739 | 11.225098 |
| H | 4.592061 | 12.128377 | 12.860694 |
| H | 3.037389 | 11.867675 | 13.660495 |
| H | 3.497451 | 13.502761 | 13.161322 |
| H | 0.810394 | 12.295796 | 10.758776 |
| H | 1.285087 | 13.714870 | 11.713791 |
| H | 0.939696 | 12.176682 | 12.514274 |
| H | 2.424271 | 10.378566 | 10.346866 |
| H | 2.337711 | 10.159716 | 12.100618 |
| H | 3.914544 | 10.292651 | 11.308738 |
| H | 1.217900 | 14.272389 | 7.570495 |
| H | 1.876641 | 15.156375 | 8.953696 |
| H | 0.908953 | 13.697044 | 9.211424 |
| H | 2.096712 | 12.022183 | 6.912522 |
| H | 1.855284 | 11.405050 | 8.552151 |
| H | 3.452167 | 11.279898 | 7.784275 |
| H | 4.811280 | 13.496369 | 7.321627 |
| H | 4.072753 | 15.042811 | 7.742842 |
| H | 3.358751 | 14.055905 | 6.457745 |
| H | 9.507949 | 13.113794 | 9.190496 |
| H | 9.492742 | 12.100651 | 10.624227 |
| H | 10.321639 | 13.672900 | 10.667356 |
| H | 6.536697 | 15.600004 | 13.246276 |
| H | 6.797023 | 14.472348 | 14.582375 |
| H | 5.786914 | 13.994911 | 13.216410 |
| H | 10.095501 | 14.339227 | 12.844245 |
| H | 9.248335 | 14.728140 | 14.345811 |
| H | 9.110460 | 15.809189 | 12.956331 |
| H | 7.198219 | 11.918963 | 13.141587 |
| H | 8.248282 | 12.541883 | 14.421788 |
| H | 8.950705 | 12.001141 | 12.886542 |
| H | 7.217678 | 16.663664 | 8.229763 |
| H | 6.010543 | 15.549278 | 8.909411 |
| H | 7.443916 | 14.901429 | 8.139877 |
| H | 7.115184 | 17.781293 | 10.431613 |
| H | 7.649714 | 17.008774 | 11.925082 |
| H | 6.084383 | 16.581111 | 11.214114 |
| H | 9.808154 | 15.566836 | 9.211035 |
| H | 9.827881 | 16.286298 | 10.821285 |
| H | 9.249908 | 17.230905 | 9.440186 |

Calculated energies and coordinates of **5**

Electronic energy ... -3248.25453276 Eh
Total Enthalpy ... -3247.00244338 Eh
Final Gibbs free energy ... -3247.15711887 Eh

CARTESIAN COORDINATES (ANGSTROEM)

| | | | |
|---|---|---|---|
| Si | 7.065549 | 11.327500 | 8.089788 |
| Si | 6.066165 | 12.579048 | 9.905337 |
| Si | 3.734431 | 13.142872 | 10.269958 |
| Si | 7.760607 | 14.039512 | 10.862630 |
| O | 7.576852 | 10.447488 | 9.517695 |
| O | 6.648617 | 10.181369 | 11.551110 |
| N | 8.401382 | 11.923827 | 7.263436 |
| N | 10.178785 | 10.751154 | 6.077451 |
| N | 9.513495 | 12.707517 | 5.278868 |
| N | 6.086861 | 10.321640 | 7.195813 |
| N | 5.057465 | 9.499541 | 5.185068 |
| N | 4.963121 | 8.185447 | 6.965039 |
| C | 9.263411 | 11.786940 | 6.313324 |
| C | 10.917512 | 11.030284 | 4.936176 |
| C | 10.523614 | 12.224622 | 4.459784 |
| C | 10.404907 | 9.558073 | 6.940556 |
| C | 11.643515 | 8.797765 | 6.451577 |
| C | 9.197710 | 8.625960 | 6.832859 |
| C | 10.668132 | 10.009333 | 8.381922 |
| C | 9.067520 | 14.127908 | 5.267279 |
| C | 7.539696 | 14.215743 | 5.313953 |
| C | 9.728076 | 14.824762 | 6.460240 |
| C | 9.531856 | 14.807740 | 3.974791 |
| C | 5.448585 | 9.431267 | 6.531373 |
| C | 4.384655 | 8.341242 | 4.830167 |
| C | 4.325944 | 7.544192 | 5.913186 |
| C | 5.398093 | 10.599544 | 4.246291 |
| C | 6.921923 | 10.679510 | 4.115848 |
| C | 4.792578 | 11.909546 | 4.761123 |
| C | 4.800950 | 10.305070 | 2.866809 |
| C | 5.065818 | 7.620856 | 8.342443 |
| C | 6.533453 | 7.401304 | 8.715106 |
| C | 4.377095 | 8.567648 | 9.323766 |
| C | 4.348002 | 6.267751 | 8.395941 |
| C | 3.668827 | 14.970863 | 10.774606 |
| C | 2.887530 | 12.161564 | 11.719331 |
| C | 3.898074 | 11.935580 | 12.855033 |
| C | 1.711016 | 12.979272 | 12.283819 |
| C | 2.364401 | 10.789797 | 11.279361 |
| C | 2.806645 | 13.045274 | 8.571498 |
| C | 1.329671 | 13.448409 | 8.719960 |
| C | 2.872259 | 11.649846 | 7.937854 |
| C | 3.484549 | 14.039906 | 7.612035 |
| C | 9.374848 | 13.189925 | 10.351712 |
| C | 7.795745 | 14.120900 | 12.800887 |

| | | | |
|---|---|---|---|
| C | 6.544920 | 14.759043 | 13.417770 |
| C | 9.028144 | 14.925313 | 13.254846 |
| C | 7.921567 | 12.693275 | 13.358950 |
| C | 7.730043 | 15.796120 | 10.016622 |
| C | 7.061688 | 15.645020 | 8.642179 |
| C | 6.971603 | 16.876747 | 10.800052 |
| C | 9.172112 | 16.298652 | 9.813388 |
| C | 6.762924 | 10.846687 | 10.548804 |
| H | 11.653844 | 10.357592 | 4.534316 |
| H | 10.866196 | 12.743242 | 3.582161 |
| H | 11.509642 | 8.383561 | 5.446918 |
| H | 11.804794 | 7.957684 | 7.132546 |
| H | 12.543190 | 9.421422 | 6.471684 |
| H | 9.082120 | 8.272805 | 5.801784 |
| H | 8.273096 | 9.122855 | 7.125165 |
| H | 9.340338 | 7.759921 | 7.486163 |
| H | 11.568175 | 10.632836 | 8.421718 |
| H | 10.826690 | 9.128232 | 9.011392 |
| H | 9.833088 | 10.570090 | 8.795524 |
| H | 7.238350 | 15.266430 | 5.374458 |
| H | 7.134144 | 13.683667 | 6.172860 |
| H | 7.112876 | 13.787080 | 4.402680 |
| H | 10.818019 | 14.802431 | 6.351016 |
| H | 9.461563 | 14.316212 | 7.387284 |
| H | 9.402433 | 15.867503 | 6.528220 |
| H | 9.154882 | 14.290653 | 3.085612 |
| H | 10.621284 | 14.888982 | 3.912122 |
| H | 9.126721 | 15.823516 | 3.967352 |
| H | 3.989621 | 8.166335 | 3.845425 |
| H | 3.872947 | 6.573557 | 6.004595 |
| H | 7.315222 | 9.743418 | 3.704667 |
| H | 7.380303 | 10.857996 | 5.087134 |
| H | 7.202746 | 11.496735 | 3.443776 |
| H | 5.021082 | 12.722728 | 4.065225 |
| H | 5.185665 | 12.165498 | 5.744725 |
| H | 3.704655 | 11.814807 | 4.835073 |
| H | 5.056792 | 11.138104 | 2.206116 |
| H | 3.708900 | 10.230293 | 2.899298 |
| H | 5.213576 | 9.392925 | 2.423727 |
| H | 7.024969 | 6.776577 | 7.961455 |
| H | 6.586069 | 6.888860 | 9.681127 |
| H | 7.068279 | 8.343969 | 8.816755 |
| H | 4.793294 | 9.573370 | 9.258546 |
| H | 4.520861 | 8.208934 | 10.347605 |
| H | 3.304556 | 8.618326 | 9.108746 |
| H | 3.287013 | 6.350265 | 8.138570 |
| H | 4.409783 | 5.902046 | 9.424309 |
| H | 4.823187 | 5.521726 | 7.749970 |
| H | 4.207761 | 15.607920 | 10.069270 |
| H | 4.101352 | 15.133310 | 11.766472 |
| H | 2.630456 | 15.320166 | 10.808344 |
| H | 4.766189 | 11.352436 | 12.529894 |
| H | 3.413469 | 11.381966 | 13.672981 |
| H | 4.249458 | 12.881326 | 13.278276 |
| H | 0.948622 | 13.201255 | 11.529850 |
| H | 2.044612 | 13.927572 | 12.717032 |
| H | 1.224281 | 12.405331 | 13.085687 |
| H | 1.567799 | 10.866177 | 10.532357 |
| H | 1.949628 | 10.259610 | 12.148978 |
| H | 3.166149 | 10.170345 | 10.871135 |
| H | 0.857542 | 13.483898 | 7.727045 |
| H | 1.208259 | 14.438071 | 9.174403 |
| H | 0.766671 | 12.725840 | 9.319692 |
| H | 2.373604 | 11.665073 | 6.957429 |
| H | 2.368963 | 10.894838 | 8.546662 |
| H | 3.904124 | 11.322747 | 7.781465 |
| H | 4.550571 | 13.815259 | 7.480810 |
| H | 3.394516 | 15.074978 | 7.959193 |
| H | 3.011411 | 13.982726 | 6.620747 |
| H | 9.451263 | 13.113645 | 9.262703 |
| H | 9.414508 | 12.167278 | 10.739293 |
| H | 10.249405 | 13.733589 | 10.725344 |
| H | 6.451496 | 15.821894 | 13.183390 |
| H | 6.581218 | 14.662767 | 14.512738 |
| H | 5.637607 | 14.255820 | 13.076671 |
| H | 9.964360 | 14.482312 | 12.898849 |
| H | 9.070237 | 14.935443 | 14.353958 |
| H | 8.993889 | 15.967908 | 12.921804 |
| H | 7.050513 | 12.076783 | 13.120985 |
| H | 8.013822 | 12.738413 | 14.453904 |
| H | 8.806166 | 12.174139 | 12.976137 |
| H | 7.177228 | 16.568870 | 8.055063 |
| H | 5.989087 | 15.444985 | 8.739198 |
| H | 7.492831 | 14.821381 | 8.068191 |
| H | 6.914855 | 17.792359 | 10.192626 |
| H | 7.478682 | 17.139899 | 11.733300 |
| H | 5.948100 | 16.579326 | 11.041993 |
| H | 9.766232 | 15.625105 | 9.191313 |
| H | 9.700499 | 16.423924 | 10.764715 |
| H | 9.151147 | 17.279667 | 9.315520 |

---

Calculated energies and coordinates of **TS5a**

Electronic energy ... -3436.77847582 Eh
Total Enthalpy ... -3435.51071644 Eh
Final Gibbs free energy ... -3435.67196214 Eh

CARTESIAN COORDINATES (ANGSTROEM)

| | | | |
|---|---|---|---|
| Si | 1.056837 | 0.222040 | -0.100545 |
| Si | -1.807160 | 0.003800 | 0.174116 |
| Si | -2.719199 | 0.519214 | 2.451560 |
| Si | -3.317475 | -0.552175 | -1.726208 |
| O | 0.320674 | -2.360353 | -0.367583 |
| O | -1.700102 | -2.644011 | 0.656550 |
| O | 0.317421 | 1.733802 | 0.164027 |
| O | -1.578726 | 2.865576 | -0.184885 |
| N | 2.028300 | -0.273281 | 1.114654 |
| N | 3.654381 | 0.602459 | 2.667353 |
| N | 2.964348 | -1.477761 | 2.974377 |
| N | 1.702122 | 0.184958 | -1.598756 |
| N | 2.270277 | 1.525356 | -3.537985 |
| N | 3.012067 | -0.556761 | -3.467078 |
| C | -0.743974 | -2.107961 | 0.146475 |
| C | -1.050372 | 1.788054 | -0.039322 |
| C | 2.793525 | -0.366576 | 2.146907 |
| C | 4.302316 | 0.090635 | 3.782263 |
| H | 4.997598 | 0.660901 | 4.371404 |
| C | 3.881068 | -1.174053 | 3.968250 |
| H | 4.154086 | -1.866194 | 4.744182 |
| C | 2.397926 | -2.840925 | 2.744196 |
| C | 2.936502 | -3.805506 | 3.804551 |
| H | 2.484450 | -4.783617 | 3.618576 |
| H | 2.657290 | -3.503516 | 4.819255 |
| H | 4.023507 | -3.923868 | 3.744392 |
| C | 2.845492 | -3.336072 | 1.365950 |
| H | 2.434410 | -2.716881 | 0.571761 |
| H | 2.487585 | -4.359226 | 1.214299 |
| H | 3.939509 | -3.333483 | 1.303084 |
| C | 0.875562 | -2.786305 | 2.868607 |
| H | 0.464536 | -1.976553 | 2.268961 |
| H | 0.583412 | -2.627252 | 3.911480 |

| | | | |
|---|---|---|---|
| H | 0.435336 | -3.725756 | 2.520714 |
| C | 3.960290 | 1.925496 | 2.052583 |
| C | 5.093585 | 2.599105 | 2.834980 |
| H | 5.304225 | 3.558664 | 2.355182 |
| H | 6.015352 | 2.008445 | 2.819842 |
| H | 4.811339 | 2.807112 | 3.871998 |
| C | 2.726766 | 2.828486 | 2.129935 |
| H | 2.950343 | 3.795136 | 1.665963 |
| H | 2.454749 | 3.003096 | 3.176096 |
| H | 1.864824 | 2.403138 | 1.620199 |
| C | 4.446507 | 1.712299 | 0.612697 |
| H | 3.689367 | 1.243107 | -0.016720 |
| H | 5.339882 | 1.078073 | 0.607700 |
| H | 4.706943 | 2.680426 | 0.173876 |
| C | 2.256588 | 0.370081 | -2.746120 |
| C | 3.449612 | 0.015481 | -4.647231 |
| H | 4.035570 | -0.514283 | -5.376638 |
| C | 3.001130 | 1.285620 | -4.688719 |
| H | 3.143035 | 2.021667 | -5.459415 |
| C | 1.684756 | 2.853388 | -3.184773 |
| C | 2.344035 | 3.376383 | -1.904258 |
| H | 3.429631 | 3.436991 | -2.037660 |
| H | 2.120759 | 2.745691 | -1.046499 |
| H | 1.960447 | 4.377957 | -1.686089 |
| C | 0.171538 | 2.708183 | -3.040729 |
| H | -0.281721 | 2.503431 | -4.016045 |
| H | -0.261732 | 3.626307 | -2.633797 |
| H | -0.077402 | 1.893251 | -2.365142 |
| C | 1.967954 | 3.855970 | -4.307980 |
| H | 1.532377 | 3.541484 | -5.261806 |
| H | 3.039028 | 4.043104 | -4.438946 |
| H | 1.496694 | 4.802570 | -4.029704 |
| C | 3.395945 | -1.920153 | -2.998498 |
| C | 2.141152 | -2.788547 | -2.904666 |
| H | 1.688980 | -2.904716 | -3.895636 |
| H | 1.407426 | -2.355039 | -2.229845 |
| H | 2.406047 | -3.781133 | -2.525369 |
| C | 4.122460 | -1.797274 | -1.653759 |
| H | 4.420298 | -2.793767 | -1.313254 |
| H | 3.486049 | -1.346968 | -0.891590 |
| H | 5.024442 | -1.185190 | -1.767833 |
| C | 4.358519 | -2.554535 | -4.007525 |
| H | 3.895611 | -2.693640 | -4.989944 |
| H | 4.626306 | -3.545199 | -3.630316 |
| H | 5.283365 | -1.978521 | -4.118027 |
| C | -4.632507 | -1.807213 | -1.205705 |
| H | -5.131222 | -2.185510 | -2.106486 |
| H | -5.397682 | -1.367571 | -0.561143 |
| H | -4.193725 | -2.660069 | -0.682055 |
| C | -2.354853 | -1.436495 | -3.177650 |
| C | -3.205176 | -1.428545 | -4.462090 |
| H | -3.350871 | -0.421800 | -4.862769 |
| H | -4.189445 | -1.887506 | -4.315796 |
| H | -2.685110 | -2.014594 | -5.234118 |
| C | -2.093307 | -2.914229 | -2.836419 |
| H | -3.024197 | -3.471743 | -2.691241 |
| H | -1.476957 | -3.048946 | -1.947034 |
| H | -1.555981 | -3.382493 | -3.674383 |
| C | -1.019143 | -0.754110 | -3.482016 |
| H | -0.500543 | -1.276479 | -4.300533 |
| H | -0.354087 | -0.761400 | -2.615227 |
| H | -1.156258 | 0.285703 | -3.791907 |
| C | -4.277146 | 1.032499 | -2.312751 |
| C | -5.525533 | 0.659256 | -3.134076 |
| H | -6.074088 | 1.579777 | -3.381681 |
| H | -6.208412 | 0.016917 | -2.567987 |
| H | -5.290092 | 0.157353 | -4.075069 |
| C | -4.761205 | 1.774143 | -1.060901 |
| H | -5.396287 | 2.624827 | -1.350252 |
| H | -3.920428 | 2.169319 | -0.487492 |
| H | -5.360654 | 1.125852 | -0.411291 |
| C | -3.400954 | 1.995823 | -3.124849 |
| H | -3.063918 | 1.559490 | -4.070771 |
| H | -2.523605 | 2.312368 | -2.554144 |
| H | -3.975614 | 2.901690 | -3.368761 |
| C | -4.015164 | 1.881516 | 2.223635 |
| H | -3.638793 | 2.695034 | 1.598289 |
| H | -4.262556 | 2.299372 | 3.207117 |
| H | -4.940360 | 1.508472 | 1.778439 |
| C | -1.399968 | 1.336393 | 3.645593 |
| C | -1.985249 | 1.452021 | 5.065827 |
| H | -2.940175 | 1.988363 | 5.088615 |
| H | -1.279811 | 2.017839 | 5.692056 |
| H | -2.124807 | 0.475618 | 5.538048 |
| C | -1.062057 | 2.765705 | 3.184757 |
| H | -0.562984 | 2.792544 | 2.215426 |
| H | -0.379078 | 3.220898 | 3.917499 |
| H | -1.949253 | 3.403485 | 3.123710 |
| C | -0.099093 | 0.536790 | 3.738830 |
| H | 0.380374 | 0.438853 | 2.763451 |
| H | -0.265380 | -0.467883 | 4.136075 |
| H | 0.611173 | 1.044482 | 4.409920 |
| C | -3.651375 | -0.981183 | 3.260529 |
| C | -2.696387 | -2.018293 | 3.868207 |
| H | -1.978093 | -2.385894 | 3.130102 |
| H | -3.272112 | -2.882973 | 4.230310 |
| H | -2.143576 | -1.615471 | 4.723457 |
| C | -4.489222 | -1.664511 | 2.172463 |
| H | -5.188201 | -0.962495 | 1.702938 |
| H | -5.087677 | -2.473483 | 2.617087 |
| H | -3.859045 | -2.099114 | 1.397823 |
| C | -4.638484 | -0.505936 | 4.345148 |
| H | -5.373295 | 0.201603 | 3.946722 |
| H | -4.150886 | -0.040591 | 5.203797 |
| H | -5.196067 | -1.377359 | 4.718627 |

---

Calculated energies and coordinates of **INT3a**

Electronic energy ... -1480.13055372 Eh
Total Enthalpy ... -1479.49486583 Eh
Final Gibbs free energy ... -1479.58814655 Eh

CARTESIAN COORDINATES (ANGSTROEM)

| | | | |
|---|---|---|---|
| Si | 10.908520 | 9.419728 | 5.093420 |
| N | 10.564773 | 10.464509 | 6.379484 |
| N | 9.248259 | 10.706580 | 8.382589 |
| N | 11.396233 | 11.255009 | 8.486755 |
| N | 11.238104 | 10.480087 | 3.816818 |
| N | 10.397080 | 11.290053 | 1.720811 |
| N | 12.551345 | 10.765333 | 1.817442 |
| C | 10.419936 | 10.750546 | 7.618296 |
| C | 9.520135 | 11.162772 | 9.668048 |
| C | 10.823998 | 11.498951 | 9.730344 |
| C | 7.942200 | 10.196560 | 7.894709 |
| C | 8.073950 | 8.704939 | 7.557880 |
| C | 6.891026 | 10.363748 | 8.995608 |
| C | 7.495535 | 11.006335 | 6.669011 |
| C | 12.783311 | 11.596226 | 8.078892 |
| C | 13.507104 | 12.253747 | 9.257480 |
| C | 12.747417 | 12.591996 | 6.909650 |

| | | | |
|---|---|---|---|
| C | 13.538330 | 10.315402 | 7.697147 |
| C | 11.379316 | 10.785296 | 2.582337 |
| C | 10.966237 | 11.557637 | 0.480684 |
| C | 12.273905 | 11.235797 | 0.538325 |
| C | 9.006645 | 11.610689 | 2.133710 |
| C | 8.265863 | 10.317161 | 2.500123 |
| C | 8.274732 | 12.274684 | 0.963776 |
| C | 9.032596 | 12.592238 | 3.315103 |
| C | 13.862723 | 10.261912 | 2.297677 |
| C | 14.911618 | 10.455096 | 1.198816 |
| C | 13.746169 | 8.764277 | 2.613098 |
| C | 14.301646 | 11.058846 | 3.534432 |
| H | 8.776359 | 11.211310 | 10.443682 |
| H | 11.379475 | 11.879779 | 10.569303 |
| H | 7.104935 | 8.313990 | 7.229410 |
| H | 8.391758 | 8.144141 | 8.443450 |
| H | 8.802797 | 8.537001 | 6.761614 |
| H | 5.932025 | 10.010121 | 8.607077 |
| H | 6.767661 | 11.412617 | 9.285712 |
| H | 7.126975 | 9.768686 | 9.884023 |
| H | 6.524094 | 10.637694 | 6.322787 |
| H | 8.215486 | 10.917501 | 5.854751 |
| H | 7.392693 | 12.065366 | 6.929685 |
| H | 14.521838 | 12.505208 | 8.936879 |
| H | 13.588616 | 11.579981 | 10.117167 |
| H | 13.015900 | 13.180897 | 9.570229 |
| H | 13.772447 | 12.840708 | 6.614576 |
| H | 12.242486 | 13.514811 | 7.215940 |
| H | 12.222589 | 12.177352 | 6.046477 |
| H | 14.570016 | 10.564009 | 7.425794 |
| H | 13.069632 | 9.812589 | 6.848589 |
| H | 13.558409 | 9.619557 | 8.542562 |
| H | 10.406281 | 11.943609 | -0.352927 |
| H | 13.016941 | 11.303487 | -0.236598 |
| H | 8.252183 | 9.631661 | 1.646148 |
| H | 7.231924 | 10.551610 | 2.775424 |
| H | 8.740684 | 9.808911 | 3.341999 |
| H | 7.257274 | 12.510303 | 1.287794 |
| H | 8.200633 | 11.610973 | 0.095632 |
| H | 8.754919 | 13.211399 | 0.662617 |
| H | 8.005134 | 12.826364 | 3.613702 |
| H | 9.527428 | 13.524117 | 3.019951 |
| H | 9.562384 | 12.172746 | 4.172919 |
| H | 15.874337 | 10.105544 | 1.581829 |
| H | 15.024463 | 11.509119 | 0.923440 |
| H | 14.680954 | 9.870275 | 0.302263 |
| H | 14.718737 | 8.378504 | 2.937061 |
| H | 13.434971 | 8.213150 | 1.719132 |
| H | 13.018013 | 8.577629 | 3.405796 |
| H | 15.275590 | 10.693362 | 3.876939 |
| H | 13.581634 | 10.953552 | 4.346702 |
| H | 14.396372 | 12.121952 | 3.287674 |

Calculated energies and coordinates of **INT4a**

Electronic energy ... -1692.77182925 Eh
Total Enthalpy ... -1692.16549074 Eh
Final Gibbs free energy ... -1692.25820805 Eh

CARTESIAN COORDINATES (ANGSTROEM)

| | | | |
|---|---|---|---|
| C | 18.120524 | 6.274003 | 6.482782 |
| C | 22.503209 | 6.091939 | 4.895414 |
| C | 20.390826 | 3.856633 | 4.936910 |
| C | 21.541558 | 3.010688 | 4.355273 |
| C | 19.056823 | 3.333505 | 4.386444 |
| C | 20.403640 | 3.661475 | 6.460876 |
| C | 20.372666 | 6.192445 | 2.653376 |
| C | 21.090220 | 5.230077 | 1.692275 |
| C | 18.878035 | 6.223341 | 2.300428 |
| C | 20.939922 | 7.606692 | 2.436235 |
| C | 18.146227 | 9.078294 | 8.324395 |
| C | 20.733490 | 8.493695 | 10.333336 |
| C | 20.793605 | 7.674141 | 9.030522 |
| C | 20.117863 | 6.320128 | 9.297050 |
| C | 22.257893 | 7.418256 | 8.653270 |
| C | 20.680944 | 10.164851 | 6.904792 |
| C | 21.826997 | 9.811603 | 5.946340 |
| C | 19.622625 | 10.958592 | 6.119079 |
| C | 21.227864 | 11.066199 | 8.024360 |
| O | 17.246005 | 5.687099 | 6.973200 |
| Si | 19.310089 | 7.315941 | 5.657940 |
| Si | 20.685695 | 5.720829 | 4.507764 |
| Si | 19.830975 | 8.578174 | 7.620587 |
| H | 22.813700 | 5.594721 | 5.819711 |
| H | 23.146723 | 5.720944 | 4.088508 |
| H | 22.695147 | 7.161637 | 5.011757 |
| H | 21.375316 | 1.953814 | 4.608227 |
| H | 21.611612 | 3.078945 | 3.266661 |
| H | 22.511538 | 3.297083 | 4.775653 |
| H | 19.047234 | 3.314596 | 3.292111 |
| H | 18.888521 | 2.304141 | 4.733633 |
| H | 18.205232 | 3.936116 | 4.723082 |
| H | 20.384213 | 2.587217 | 6.693773 |
| H | 21.304103 | 4.080216 | 6.925641 |
| H | 19.532428 | 4.116851 | 6.938747 |
| H | 20.641082 | 4.231873 | 1.704417 |
| H | 21.008926 | 5.609583 | 0.663711 |
| H | 22.157991 | 5.128434 | 1.918823 |
| H | 18.750487 | 6.546633 | 1.257371 |
| H | 18.403451 | 5.242599 | 2.401507 |
| H | 18.332457 | 6.927871 | 2.938482 |
| H | 22.018861 | 7.656439 | 2.620267 |
| H | 20.765348 | 7.919476 | 1.396999 |
| H | 20.445112 | 8.343029 | 3.082293 |
| H | 17.691950 | 8.259354 | 8.891624 |
| H | 18.250467 | 9.934162 | 9.002145 |
| H | 17.449556 | 9.359949 | 7.529511 |
| H | 19.701697 | 8.651575 | 10.665958 |
| H | 21.253395 | 7.945231 | 11.131898 |
| H | 21.213700 | 9.471538 | 10.242361 |
| H | 20.611287 | 5.820941 | 10.143394 |
| H | 19.057579 | 6.426087 | 9.553667 |
| H | 20.188641 | 5.660370 | 8.429078 |
| H | 22.826582 | 8.347538 | 8.542057 |
| H | 22.750699 | 6.824295 | 9.436357 |
| H | 22.334914 | 6.858372 | 7.714367 |
| H | 21.462150 | 9.248389 | 5.078916 |
| H | 22.293394 | 10.730275 | 5.562485 |
| H | 22.609986 | 9.218946 | 6.430553 |
| H | 20.094640 | 11.825880 | 5.635803 |
| H | 19.158077 | 10.350273 | 5.333662 |
| H | 18.826906 | 11.334901 | 6.770547 |
| H | 22.075772 | 10.609078 | 8.544220 |
| H | 21.581517 | 12.013610 | 7.592889 |
| H | 20.461799 | 11.312960 | 8.768670 |

Calculated energies and coordinates of **INT5a**

Electronic energy ... -1668.72938386 Eh
Total Enthalpy ... -1668.07707829 Eh

Final Gibbs free energy ... -1668.17539131 Eh

CARTESIAN COORDINATES (ANGSTROEM)

| | | | |
|---|---|---|---|
| Si | 10.332143 | 5.566309 | 8.751101 |
| O | 9.135794 | 6.585717 | 8.044691 |
| O | 8.325841 | 7.396086 | 10.084833 |
| N | 11.874266 | 6.030192 | 8.434608 |
| N | 13.717020 | 7.077066 | 9.587867 |
| N | 14.176224 | 5.750477 | 7.877052 |
| N | 10.076216 | 3.946900 | 8.640620 |
| N | 8.920656 | 1.932801 | 8.103829 |
| N | 9.810166 | 2.065370 | 10.126181 |
| C | 9.070512 | 6.704200 | 9.453142 |
| C | 13.119918 | 6.253922 | 8.633759 |
| C | 15.094843 | 7.058852 | 9.410195 |
| H | 15.774897 | 7.615831 | 10.030050 |
| C | 15.374762 | 6.250001 | 8.367909 |
| H | 16.333061 | 6.002005 | 7.946857 |
| C | 14.024152 | 4.778617 | 6.759888 |
| C | 13.147139 | 5.390756 | 5.659973 |
| H | 13.606753 | 6.304774 | 5.269177 |
| H | 12.153443 | 5.634887 | 6.037485 |
| H | 13.046644 | 4.674991 | 4.837426 |
| C | 15.403425 | 4.470596 | 6.170772 |
| H | 15.268982 | 3.770663 | 5.341598 |
| H | 16.065506 | 3.994939 | 6.901895 |
| H | 15.888819 | 5.367985 | 5.772699 |
| C | 13.414887 | 3.477599 | 7.300254 |
| H | 13.338202 | 2.747200 | 6.487596 |
| H | 12.416926 | 3.641740 | 7.712009 |
| H | 14.056419 | 3.056883 | 8.082634 |
| C | 12.981297 | 7.899918 | 10.588964 |
| C | 12.047679 | 8.876470 | 9.861903 |
| H | 12.631448 | 9.569125 | 9.246569 |
| H | 11.480115 | 9.454939 | 10.597095 |
| H | 11.336419 | 8.357704 | 9.218166 |
| C | 13.988665 | 8.708210 | 11.412163 |
| H | 14.575122 | 9.390275 | 10.788110 |
| H | 14.666795 | 8.062557 | 11.980487 |
| H | 13.428834 | 9.315185 | 12.128646 |
| C | 12.206659 | 6.982799 | 11.544353 |
| H | 11.413325 | 6.437503 | 11.030977 |
| H | 11.735079 | 7.587293 | 12.325459 |
| H | 12.885053 | 6.264444 | 12.017407 |
| C | 9.633841 | 2.782964 | 8.943971 |
| C | 8.666093 | 0.739150 | 8.765275 |
| H | 8.125886 | -0.077169 | 8.319421 |
| C | 9.205182 | 0.819825 | 9.998629 |
| H | 9.202226 | 0.084450 | 10.783462 |
| C | 10.516029 | 2.570709 | 11.334816 |
| C | 10.566421 | 1.460336 | 12.388858 |
| H | 9.566348 | 1.163906 | 12.721147 |
| H | 11.102525 | 1.845153 | 13.260581 |
| H | 11.104420 | 0.577964 | 12.026652 |
| C | 9.742808 | 3.761065 | 11.915976 |
| H | 9.643054 | 4.577729 | 11.198212 |
| H | 10.265907 | 4.144604 | 12.798173 |
| H | 8.737040 | 3.450644 | 12.217648 |
| C | 11.959036 | 2.952779 | 10.973035 |
| H | 12.490509 | 2.085942 | 10.565436 |
| H | 12.479797 | 3.284898 | 11.876838 |
| H | 11.997929 | 3.756488 | 10.236604 |
| C | 8.484204 | 2.284589 | 6.723742 |
| C | 7.737253 | 1.093658 | 6.117272 |
| H | 7.430042 | 1.366282 | 5.104148 |
| H | 6.832408 | 0.846309 | 6.682191 |
| H | 8.372578 | 0.204353 | 6.044350 |
| C | 9.716855 | 2.583771 | 5.858972 |
| H | 10.383817 | 1.714904 | 5.832443 |
| H | 10.269436 | 3.440064 | 6.248135 |
| H | 9.396923 | 2.807392 | 4.836092 |
| C | 7.536195 | 3.489495 | 6.778804 |
| H | 7.180427 | 3.716976 | 5.768701 |
| H | 8.029273 | 4.378217 | 7.174772 |
| H | 6.667711 | 3.261912 | 7.406014 |

---

Calculated energies and coordinates of **TS[INT5a_INT6a]**

Electronic energy ... -1668.72265890 Eh
Total Enthalpy ... -1668.07196177 Eh
Final Gibbs free energy ... -1668.16954976 Eh

CARTESIAN COORDINATES (ANGSTROEM)

| | | | |
|---|---|---|---|
| Si | 10.242962 | 5.637678 | 8.459975 |
| O | 9.139555 | 6.428292 | 7.584823 |
| O | 8.261256 | 7.519749 | 9.787992 |
| N | 11.808862 | 6.125820 | 8.362170 |
| N | 13.676372 | 7.037255 | 9.565933 |
| N | 14.112567 | 5.697701 | 7.858027 |
| N | 10.076530 | 4.048193 | 8.837653 |
| N | 9.054794 | 1.987159 | 8.112025 |
| N | 9.888351 | 2.078763 | 10.161840 |
| C | 9.060921 | 6.784491 | 9.378970 |
| C | 13.061377 | 6.253776 | 8.587575 |
| C | 15.056006 | 6.943394 | 9.430573 |
| H | 15.746291 | 7.463788 | 10.070657 |
| C | 15.321680 | 6.125711 | 8.392165 |
| H | 16.276987 | 5.830392 | 7.995685 |
| C | 13.946790 | 4.721107 | 6.748312 |
| C | 13.108821 | 5.347923 | 5.625858 |
| H | 13.613048 | 6.233401 | 5.224490 |
| H | 12.121034 | 5.644945 | 5.980088 |
| H | 12.989371 | 4.620883 | 4.815749 |
| C | 15.323674 | 4.361691 | 6.182635 |
| H | 15.177495 | 3.665290 | 5.352426 |
| H | 15.956508 | 3.863949 | 6.924762 |
| H | 15.847134 | 5.240516 | 5.791429 |
| C | 13.291675 | 3.447162 | 7.298604 |
| H | 13.182670 | 2.712685 | 6.493617 |
| H | 12.304239 | 3.651894 | 7.717212 |
| H | 13.920129 | 3.010542 | 8.082712 |
| C | 12.952574 | 7.864816 | 10.568104 |
| C | 12.061637 | 8.883551 | 9.844721 |
| H | 12.675261 | 9.561119 | 9.241697 |
| H | 11.511077 | 9.476259 | 10.582040 |
| H | 11.343425 | 8.392609 | 9.187138 |
| C | 13.968244 | 8.625549 | 11.424876 |
| H | 14.585728 | 9.301572 | 10.824494 |
| H | 14.617229 | 7.948620 | 11.990451 |
| H | 13.414877 | 9.234205 | 12.145222 |
| C | 12.134919 | 6.947952 | 11.487367 |
| H | 11.390797 | 6.378783 | 10.928631 |
| H | 11.609985 | 7.549816 | 12.236203 |
| H | 12.796208 | 6.244734 | 12.005035 |
| C | 9.694360 | 2.841279 | 9.009923 |
| C | 8.863718 | 0.750217 | 8.716218 |
| H | 8.389692 | -0.078207 | 8.220481 |
| C | 9.371772 | 0.805315 | 9.964320 |
| H | 9.403907 | 0.033335 | 10.712811 |
| C | 10.551372 | 2.579195 | 11.394662 |
| C | 10.608002 | 1.451618 | 12.428925 |

| | | | |
|---|---|---|---|
| H | 9.608551 | 1.110961 | 12.718600 |
| H | 11.099661 | 1.837267 | 13.326118 |
| H | 11.191073 | 0.597229 | 12.069633 |
| C | 9.732800 | 3.739315 | 11.976633 |
| H | 9.644831 | 4.560684 | 11.263740 |
| H | 10.219474 | 4.115082 | 12.882832 |
| H | 8.725754 | 3.398841 | 12.239843 |
| C | 11.987674 | 3.013787 | 11.066308 |
| H | 12.554222 | 2.168670 | 10.660215 |
| H | 12.482450 | 3.352318 | 11.982599 |
| H | 12.002683 | 3.826522 | 10.338129 |
| C | 8.554509 | 2.381377 | 6.764120 |
| C | 7.900762 | 1.166004 | 6.099840 |
| H | 7.560613 | 1.466987 | 5.105507 |
| H | 7.024940 | 0.818350 | 6.657189 |
| H | 8.604594 | 0.336019 | 5.974555 |
| C | 9.728107 | 2.838132 | 5.887784 |
| H | 10.466411 | 2.035148 | 5.787322 |
| H | 10.221450 | 3.718230 | 6.301701 |
| H | 9.355114 | 3.096530 | 4.891744 |
| C | 7.504321 | 3.488573 | 6.915732 |
| H | 7.082410 | 3.723687 | 5.932925 |
| H | 7.930549 | 4.408665 | 7.319912 |
| H | 6.691440 | 3.148281 | 7.566514 |

---

Calculated energies and coordinates of **INT6a**

Electronic energy ... -1668.72597615 Eh
Total Enthalpy ... -1668.07467092 Eh
Final Gibbs free energy ... -1668.17417344 Eh

CARTESIAN COORDINATES (ANGSTROEM)

| | | | |
|---|---|---|---|
| Si | 10.393627 | 5.462713 | 8.258426 |
| O | 9.742155 | 6.095540 | 6.978826 |
| O | 8.290551 | 7.492986 | 9.305290 |
| N | 11.898539 | 5.896247 | 8.794779 |
| N | 13.785568 | 7.099669 | 9.603952 |
| N | 14.123507 | 5.790775 | 7.848631 |
| N | 9.946243 | 4.012072 | 8.907206 |
| N | 8.958501 | 1.935604 | 8.138905 |
| N | 9.714373 | 2.048818 | 10.217268 |
| C | 9.125135 | 6.729829 | 9.163003 |
| C | 13.130671 | 6.229095 | 8.728504 |
| C | 15.125572 | 7.190616 | 9.249891 |
| H | 15.834022 | 7.803702 | 9.778460 |
| C | 15.332158 | 6.390913 | 8.184763 |
| H | 16.247771 | 6.205690 | 7.651693 |
| C | 13.911335 | 4.835365 | 6.728817 |
| C | 12.960718 | 5.456012 | 5.696126 |
| H | 13.413280 | 6.355621 | 5.264718 |
| H | 11.991804 | 5.735170 | 6.117436 |
| H | 12.784701 | 4.738656 | 4.887171 |
| C | 15.252801 | 4.556173 | 6.043298 |
| H | 15.074067 | 3.849512 | 5.228211 |
| H | 15.978149 | 4.101956 | 6.726829 |
| H | 15.685234 | 5.461255 | 5.604656 |
| C | 13.375858 | 3.506286 | 7.282279 |
| H | 13.240403 | 2.799373 | 6.457412 |
| H | 12.418720 | 3.626041 | 7.792400 |
| H | 14.091664 | 3.078164 | 7.992705 |
| C | 13.108468 | 7.871584 | 10.677042 |
| C | 12.099831 | 8.835758 | 10.039003 |
| H | 12.617991 | 9.554252 | 9.395290 |
| H | 11.567147 | 9.388453 | 10.819829 |
| H | 11.371831 | 8.294812 | 9.431728 |
| C | 14.150364 | 8.685064 | 11.449521 |
| H | 14.648025 | 9.424305 | 10.813597 |
| H | 14.904983 | 8.041875 | 11.914532 |
| H | 13.636355 | 9.228659 | 12.247051 |
| C | 12.420019 | 6.909281 | 11.656501 |
| H | 11.656985 | 6.313667 | 11.153654 |
| H | 11.950937 | 7.486020 | 12.460491 |
| H | 13.155069 | 6.229589 | 12.101410 |
| C | 9.571913 | 2.799051 | 9.047976 |
| C | 8.746820 | 0.705780 | 8.748382 |
| H | 8.284683 | -0.126481 | 8.247861 |
| C | 9.206746 | 0.774668 | 10.015372 |
| H | 9.202351 | 0.012721 | 10.774898 |
| C | 10.380156 | 2.548182 | 11.449877 |
| C | 10.380802 | 1.438523 | 12.504568 |
| H | 9.365450 | 1.132623 | 12.778155 |
| H | 10.865323 | 1.825210 | 13.405155 |
| H | 10.945437 | 0.560641 | 12.173615 |
| C | 9.601982 | 3.750883 | 12.001020 |
| H | 9.580861 | 4.564516 | 11.274213 |
| H | 10.084651 | 4.109112 | 12.916480 |
| H | 8.572470 | 3.463619 | 12.240304 |
| C | 11.832770 | 2.930094 | 11.129736 |
| H | 12.373512 | 2.063112 | 10.734151 |
| H | 12.331443 | 3.256797 | 12.048653 |
| H | 11.879806 | 3.741314 | 10.400203 |
| C | 8.552413 | 2.293942 | 6.749048 |
| C | 7.860829 | 1.086966 | 6.107455 |
| H | 7.567099 | 1.367790 | 5.092803 |
| H | 6.952797 | 0.800618 | 6.648506 |
| H | 8.526447 | 0.220209 | 6.031598 |
| C | 9.797353 | 2.641065 | 5.922492 |
| H | 10.513424 | 1.812231 | 5.945592 |
| H | 10.278519 | 3.550171 | 6.285981 |
| H | 9.503150 | 2.822036 | 4.883729 |
| C | 7.565241 | 3.466886 | 6.786145 |
| H | 7.190316 | 3.649486 | 5.773752 |
| H | 8.042624 | 4.387948 | 7.125491 |
| H | 6.713760 | 3.226252 | 7.432254 |

---

Calculated energies and coordinates of **INT7a**

Electronic energy ... -3361.58298964 Eh
Total Enthalpy ... -3360.32104695 Eh
Final Gibbs free energy ... -3360.48264302 Eh

CARTESIAN COORDINATES (ANGSTROEM)

| | | | |
|---|---|---|---|
| Si | 11.236949 | 4.961331 | 10.779916 |
| Si | 11.662302 | 5.570755 | 13.888269 |
| Si | 10.167254 | 4.934294 | 15.706664 |
| C | 10.635902 | 5.844023 | 17.298849 |
| H | 10.741459 | 6.918335 | 17.132842 |
| H | 11.576093 | 5.472933 | 17.718539 |
| H | 9.854805 | 5.696242 | 18.054461 |
| C | 8.354318 | 5.465728 | 15.273500 |
| C | 7.950133 | 5.093741 | 13.838215 |
| H | 8.548214 | 5.614716 | 13.086272 |
| H | 6.901557 | 5.377043 | 13.666705 |
| H | 8.034789 | 4.018682 | 13.650127 |
| C | 7.355172 | 4.805785 | 16.242042 |
| H | 6.354693 | 5.225654 | 16.063041 |
| H | 7.603455 | 4.997758 | 17.292254 |
| H | 7.283414 | 3.723457 | 16.098100 |
| C | 8.234589 | 6.990265 | 15.456264 |
| H | 8.948589 | 7.544069 | 14.840947 |

| | | | |
|---|---|---|---|
| H | 8.378607 | 7.280002 | 16.503060 |
| H | 7.225067 | 7.312833 | 15.163029 |
| C | 10.366610 | 3.039355 | 16.071361 |
| C | 11.854375 | 2.661787 | 16.037398 |
| H | 12.252121 | 2.732019 | 15.020961 |
| H | 11.978161 | 1.618530 | 16.364091 |
| H | 12.462775 | 3.282663 | 16.704388 |
| C | 9.654185 | 2.170892 | 15.026930 |
| H | 10.020908 | 2.388463 | 14.019926 |
| H | 8.567514 | 2.300929 | 15.036507 |
| H | 9.859671 | 1.108624 | 15.227361 |
| C | 9.828526 | 2.704044 | 17.474950 |
| H | 8.764354 | 2.926490 | 17.590368 |
| H | 10.374201 | 3.240596 | 18.258449 |
| H | 9.961477 | 1.628554 | 17.661909 |
| Si | 13.838091 | 6.621862 | 14.088362 |
| C | 14.551690 | 6.618187 | 12.335959 |
| H | 13.856903 | 7.060191 | 11.619123 |
| H | 14.759432 | 5.601906 | 11.986827 |
| H | 15.487031 | 7.191010 | 12.302219 |
| C | 15.021882 | 5.525275 | 15.151098 |
| C | 14.953060 | 4.093034 | 14.594235 |
| H | 15.351908 | 4.034197 | 13.575852 |
| H | 13.935400 | 3.699801 | 14.566228 |
| H | 15.553805 | 3.420300 | 15.223414 |
| C | 14.598008 | 5.516427 | 16.624342 |
| H | 14.690852 | 6.505528 | 17.086128 |
| H | 15.225854 | 4.820643 | 17.199853 |
| H | 13.557945 | 5.194953 | 16.737125 |
| C | 16.489028 | 5.979828 | 15.046569 |
| H | 16.823199 | 6.036913 | 14.004606 |
| H | 17.129367 | 5.243148 | 15.553372 |
| H | 16.671064 | 6.948629 | 15.516691 |
| C | 13.662960 | 8.466565 | 14.664379 |
| C | 13.293117 | 9.345275 | 13.455958 |
| H | 14.090394 | 9.352870 | 12.704818 |
| H | 13.149439 | 10.381989 | 13.793496 |
| H | 12.362482 | 9.026226 | 12.980944 |
| C | 14.984831 | 9.002344 | 15.238991 |
| H | 15.816197 | 8.897868 | 14.532097 |
| H | 15.262953 | 8.507550 | 16.174642 |
| H | 14.873824 | 10.074526 | 15.457446 |
| C | 12.567366 | 8.607456 | 15.730088 |
| H | 11.584949 | 8.342757 | 15.322951 |
| H | 12.512774 | 9.649590 | 16.077689 |
| H | 12.765320 | 7.976187 | 16.603673 |
| O | 12.264598 | 3.059117 | 12.605060 |
| O | 10.476782 | 6.343620 | 11.459209 |
| O | 10.293706 | 7.904267 | 13.028622 |
| N | 12.502559 | 5.455867 | 9.841067 |
| C | 11.847780 | 4.203571 | 12.470893 |
| N | 13.898682 | 6.990487 | 8.602593 |
| N | 14.382914 | 4.829884 | 8.487936 |
| N | 10.059636 | 4.051614 | 10.060778 |
| N | 8.679024 | 3.999122 | 8.072659 |
| N | 8.970244 | 2.108832 | 9.185993 |
| C | 10.702150 | 6.803546 | 12.714638 |
| C | 13.474204 | 5.732062 | 9.060628 |
| C | 15.024794 | 6.831395 | 7.804903 |
| H | 15.542773 | 7.663329 | 7.358652 |
| C | 15.318039 | 5.520853 | 7.733333 |
| H | 16.130834 | 5.040861 | 7.217834 |
| C | 14.275160 | 3.346482 | 8.558284 |
| C | 12.932004 | 2.911393 | 7.957246 |
| H | 12.850039 | 3.262568 | 6.922618 |
| H | 12.088510 | 3.301964 | 8.528546 |
| H | 12.870535 | 1.818678 | 7.957553 |
| C | 15.400484 | 2.717433 | 7.730447 |
| H | 15.303797 | 1.630975 | 7.804872 |
| H | 16.390858 | 2.986793 | 8.112338 |
| H | 15.334322 | 2.985628 | 6.670834 |
| C | 14.427220 | 2.882378 | 10.009939 |
| H | 14.356499 | 1.790702 | 10.058350 |
| H | 13.651704 | 3.293454 | 10.652609 |
| H | 15.405043 | 3.182789 | 10.402223 |
| C | 13.279265 | 8.344277 | 8.735513 |
| C | 12.894036 | 8.824416 | 7.326645 |
| H | 13.763743 | 8.917270 | 6.669760 |
| H | 12.415039 | 9.806326 | 7.393109 |
| H | 12.190310 | 8.123646 | 6.867275 |
| C | 14.323239 | 9.289325 | 9.350135 |
| H | 15.207817 | 9.400107 | 8.716016 |
| H | 14.643697 | 8.919098 | 10.327781 |
| H | 13.881117 | 10.281399 | 9.484225 |
| C | 12.030354 | 8.357370 | 9.610122 |
| H | 11.235930 | 7.724989 | 9.213515 |
| H | 11.663452 | 9.388227 | 9.644928 |
| H | 12.229919 | 8.041779 | 10.632763 |
| C | 9.328689 | 3.460850 | 9.189027 |
| C | 7.971096 | 2.997712 | 7.421139 |
| H | 7.404216 | 3.166354 | 6.523078 |
| C | 8.142829 | 1.849322 | 8.103880 |
| H | 7.745258 | 0.873788 | 7.887548 |
| C | 9.292528 | 1.137203 | 10.267685 |
| C | 8.649581 | -0.214811 | 9.944242 |
| H | 7.557343 | -0.152628 | 9.899062 |
| H | 8.908675 | -0.910182 | 10.747136 |
| H | 9.027772 | -0.636050 | 9.006542 |
| C | 8.709437 | 1.656360 | 11.586019 |
| H | 9.148455 | 2.620029 | 11.847246 |
| H | 8.921718 | 0.943534 | 12.388473 |
| H | 7.623564 | 1.775620 | 11.501797 |
| C | 10.809295 | 0.944101 | 10.361823 |
| H | 11.198487 | 0.552925 | 9.415702 |
| H | 11.033992 | 0.223337 | 11.154779 |
| H | 11.320673 | 1.874170 | 10.608795 |
| C | 8.725089 | 5.422816 | 7.644025 |
| C | 7.791710 | 5.623274 | 6.445258 |
| H | 7.821476 | 6.681186 | 6.170573 |
| H | 6.754203 | 5.371889 | 6.688303 |
| H | 8.110186 | 5.046174 | 5.570881 |
| C | 10.152818 | 5.769819 | 7.208416 |
| H | 10.462471 | 5.129244 | 6.375073 |
| H | 10.865825 | 5.643855 | 8.025115 |
| H | 10.185145 | 6.811866 | 6.873156 |
| C | 8.239002 | 6.324842 | 8.785199 |
| H | 8.261479 | 7.369016 | 8.456219 |
| H | 8.857580 | 6.235199 | 9.677066 |
| H | 7.207478 | 6.071095 | 9.052184 |

---

Calculated energies and coordinates of **INT8a**

| | | |
|---|---|---|
| Electronic energy | ... | -3550.17292178 Eh |
| Total Enthalpy | ... | -3548.89375005 Eh |
| Final Gibbs free energy | ... | -3549.06072551 Eh |

CARTESIAN COORDINATES (ANGSTROEM)

| | | | |
|---|---|---|---|
| Si | 10.746211 | 4.924720 | 10.870983 |
| Si | 11.483703 | 5.600511 | 13.882977 |
| Si | 10.100723 | 4.938748 | 15.761402 |
| C | 10.654834 | 5.805633 | 17.348407 |

| | | | |
|---|---|---|---|
| H | 10.672379 | 6.891155 | 17.231470 |
| H | 11.656318 | 5.485125 | 17.655112 |
| H | 9.963438 | 5.567066 | 18.165503 |
| C | 8.277779 | 5.475884 | 15.400911 |
| C | 7.841930 | 5.141369 | 13.965816 |
| H | 8.426455 | 5.688703 | 13.221262 |
| H | 6.790928 | 5.430272 | 13.820755 |
| H | 7.926207 | 4.074644 | 13.735196 |
| C | 7.306689 | 4.791573 | 16.380230 |
| H | 6.300293 | 5.211717 | 16.239774 |
| H | 7.584415 | 4.959266 | 17.427476 |
| H | 7.234607 | 3.712682 | 16.212232 |
| C | 8.166141 | 6.996923 | 15.616183 |
| H | 8.864438 | 7.560286 | 14.990055 |
| H | 8.338991 | 7.268342 | 16.663556 |
| H | 7.150233 | 7.326808 | 15.355329 |
| C | 10.338845 | 3.038808 | 16.050397 |
| C | 11.835007 | 2.700240 | 15.961943 |
| H | 12.219547 | 2.854008 | 14.949457 |
| H | 11.989069 | 1.639744 | 16.210016 |
| H | 12.433383 | 3.287198 | 16.667955 |
| C | 9.596593 | 2.190491 | 15.008210 |
| H | 9.899427 | 2.445422 | 13.989033 |
| H | 8.509709 | 2.296235 | 15.083311 |
| H | 9.833633 | 1.127473 | 15.161514 |
| C | 9.852936 | 2.648009 | 17.458947 |
| H | 8.790961 | 2.856953 | 17.614934 |
| H | 10.419780 | 3.161705 | 18.242668 |
| H | 10.000649 | 1.567935 | 17.602002 |
| Si | 13.635115 | 6.705570 | 13.848155 |
| C | 13.987120 | 6.919015 | 12.003119 |
| H | 13.180513 | 7.469501 | 11.510069 |
| H | 14.087842 | 5.960223 | 11.485876 |
| H | 14.916827 | 7.481837 | 11.855955 |
| C | 14.994447 | 5.534625 | 14.566632 |
| C | 14.867408 | 4.170933 | 13.867741 |
| H | 15.031788 | 4.256894 | 12.786907 |
| H | 13.890514 | 3.704166 | 14.010319 |
| H | 15.627055 | 3.480834 | 14.263356 |
| C | 14.811073 | 5.341133 | 16.077640 |
| H | 14.957649 | 6.271955 | 16.635743 |
| H | 15.535485 | 4.607677 | 16.460387 |
| H | 13.808629 | 4.966105 | 16.310026 |
| C | 16.414388 | 6.055073 | 14.279122 |
| H | 16.568193 | 6.234841 | 13.208109 |
| H | 17.146961 | 5.296483 | 14.591399 |
| H | 16.650336 | 6.975901 | 14.816346 |
| C | 13.537189 | 8.473629 | 14.647438 |
| C | 13.048765 | 9.490439 | 13.599445 |
| H | 13.760263 | 9.593441 | 12.772589 |
| H | 12.954880 | 10.476837 | 14.076217 |
| H | 12.070953 | 9.230069 | 13.188208 |
| C | 14.911563 | 8.953038 | 15.144023 |
| H | 15.659853 | 8.961824 | 14.343428 |
| H | 15.295949 | 8.345781 | 15.969103 |
| H | 14.817802 | 9.984228 | 15.514302 |
| C | 12.558294 | 8.476344 | 15.829022 |
| H | 11.539986 | 8.246469 | 15.495484 |
| H | 12.538355 | 9.469694 | 16.300646 |
| H | 12.844221 | 7.748312 | 16.596622 |
| O | 11.996138 | 3.076112 | 12.555385 |
| O | 10.517656 | 6.526978 | 11.409075 |
| O | 10.137064 | 7.962354 | 13.067752 |
| N | 13.216579 | 4.831851 | 7.844775 |
| C | 11.547244 | 4.212777 | 12.479396 |
| N | 14.609672 | 6.683039 | 8.523832 |
| N | 15.275704 | 4.662056 | 9.087880 |
| N | 9.330201 | 4.221759 | 10.471139 |
| N | 7.730303 | 4.424012 | 8.685604 |
| N | 8.437467 | 2.398903 | 9.196430 |
| C | 10.570072 | 6.882579 | 12.726176 |
| C | 14.249123 | 5.356634 | 8.492182 |
| C | 15.858737 | 6.795181 | 9.112073 |
| H | 16.345888 | 7.738466 | 9.284521 |
| C | 16.267242 | 5.555575 | 9.460219 |
| H | 17.163695 | 5.259049 | 9.975634 |
| C | 15.394986 | 3.167855 | 9.133339 |
| C | 15.548846 | 2.656873 | 7.696238 |
| H | 16.444039 | 3.082406 | 7.229764 |
| H | 14.673753 | 2.920566 | 7.097336 |
| H | 15.650308 | 1.566914 | 7.709168 |
| C | 16.637250 | 2.799116 | 9.947759 |
| H | 16.694648 | 1.708919 | 9.999221 |
| H | 16.578234 | 3.177425 | 10.974338 |
| H | 17.561836 | 3.153341 | 9.480730 |
| C | 14.165563 | 2.552937 | 9.807245 |
| H | 14.345292 | 1.483482 | 9.956361 |
| H | 13.273666 | 2.660278 | 9.191294 |
| H | 13.971780 | 3.004597 | 10.784101 |
| C | 13.886661 | 7.794914 | 7.823575 |
| C | 14.046886 | 7.583371 | 6.313872 |
| H | 15.105329 | 7.595086 | 6.031986 |
| H | 13.535673 | 8.388739 | 5.776572 |
| H | 13.606810 | 6.628585 | 6.014196 |
| C | 14.521791 | 9.124403 | 8.239073 |
| H | 15.561837 | 9.213344 | 7.909817 |
| H | 14.467056 | 9.271907 | 9.323481 |
| H | 13.960668 | 9.931829 | 7.762261 |
| C | 12.404022 | 7.823677 | 8.212941 |
| H | 11.849688 | 7.001925 | 7.760021 |
| H | 11.969523 | 8.760146 | 7.849022 |
| H | 12.272940 | 7.781266 | 9.297313 |
| C | 8.592812 | 3.734841 | 9.522357 |
| C | 7.097428 | 3.522990 | 7.842745 |
| H | 6.398272 | 3.818652 | 7.081309 |
| C | 7.528418 | 2.284124 | 8.156579 |
| H | 7.261038 | 1.344081 | 7.708219 |
| C | 9.126399 | 1.263273 | 9.874389 |
| C | 8.652907 | -0.053098 | 9.252293 |
| H | 7.571081 | -0.189101 | 9.352731 |
| H | 9.140744 | -0.871937 | 9.787709 |
| H | 8.933229 | -0.132154 | 8.196546 |
| C | 8.754118 | 1.269937 | 11.361963 |
| H | 9.035896 | 2.216679 | 11.824368 |
| H | 9.284103 | 0.460893 | 11.874956 |
| H | 7.676451 | 1.119159 | 11.489200 |
| C | 10.642261 | 1.380308 | 9.679327 |
| H | 10.884092 | 1.521031 | 8.621722 |
| H | 11.122389 | 0.465243 | 10.042511 |
| H | 11.054893 | 2.213828 | 10.247782 |
| C | 7.500578 | 5.898902 | 8.697266 |
| C | 6.388942 | 6.237024 | 7.698637 |
| H | 6.207797 | 7.313822 | 7.749763 |
| H | 5.449852 | 5.729430 | 7.942733 |
| H | 6.674506 | 5.998499 | 6.668759 |
| C | 8.776660 | 6.630497 | 8.267726 |
| H | 9.140165 | 6.243054 | 7.311731 |
| H | 9.571081 | 6.521621 | 9.005210 |
| H | 8.560402 | 7.699671 | 8.168487 |
| C | 7.049055 | 6.334527 | 10.096979 |
| H | 6.855529 | 7.411807 | 10.094109 |
| H | 7.814838 | 6.125919 | 10.843779 |

| | | | |
|---|---|---|---|
| H | 6.126984 | 5.814758 | 10.379184 |
| O | 11.017077 | 4.218486 | 7.866398 |
| C | 12.015835 | 4.660007 | 8.409231 |
| O | 11.979902 | 5.052006 | 9.764296 |

---

Calculated energies and coordinates of **3**

Electronic energy ... -3738.76007608 Eh
Total Enthalpy ... -3737.46302385 Eh
Final Gibbs free energy ... -3737.63354470 Eh

CARTESIAN COORDINATES (ANGSTROEM)

| | | | |
|---|---|---|---|
| Si | 10.938649 | 5.457089 | 11.004049 |
| Si | 11.768659 | 5.890528 | 14.095720 |
| Si | 10.021263 | 5.620512 | 15.757258 |
| C | 10.705399 | 5.986582 | 17.487666 |
| H | 11.299045 | 6.904029 | 17.504778 |
| H | 11.331899 | 5.170607 | 17.859930 |
| H | 9.876445 | 6.109875 | 18.194988 |
| C | 8.581828 | 6.885793 | 15.466865 |
| C | 8.069068 | 6.924343 | 14.019265 |
| H | 8.816829 | 7.302763 | 13.320798 |
| H | 7.204154 | 7.601203 | 13.960839 |
| H | 7.746810 | 5.944230 | 13.657278 |
| C | 7.391154 | 6.548064 | 16.383680 |
| H | 6.653900 | 7.361915 | 16.324279 |
| H | 7.679690 | 6.448121 | 17.436499 |
| H | 6.883137 | 5.628914 | 16.075392 |
| C | 9.092135 | 8.287059 | 15.852119 |
| H | 9.964178 | 8.585998 | 15.262497 |
| H | 9.348908 | 8.348189 | 16.915797 |
| H | 8.299746 | 9.025489 | 15.661868 |
| C | 9.460741 | 3.764413 | 15.778959 |
| C | 10.706463 | 2.867590 | 15.714171 |
| H | 11.212146 | 2.939867 | 14.746892 |
| H | 10.411782 | 1.816175 | 15.854245 |
| H | 11.428503 | 3.108316 | 16.504495 |
| C | 8.557995 | 3.423672 | 14.586158 |
| H | 9.009468 | 3.709716 | 13.628998 |
| H | 7.583580 | 3.918282 | 14.652847 |
| H | 8.375319 | 2.339111 | 14.559306 |
| C | 8.719633 | 3.430103 | 17.087322 |
| H | 7.807642 | 4.016704 | 17.223143 |
| H | 9.356210 | 3.584778 | 17.965269 |
| H | 8.430376 | 2.368754 | 17.076304 |
| Si | 14.122374 | 6.232119 | 14.483631 |
| C | 14.880634 | 6.060474 | 12.754522 |
| H | 14.329672 | 6.649460 | 12.016128 |
| H | 14.849968 | 5.020472 | 12.414633 |
| H | 15.925760 | 6.394152 | 12.751357 |
| C | 14.866535 | 4.801813 | 15.553863 |
| C | 14.348748 | 3.467756 | 14.993724 |
| H | 14.664821 | 3.301506 | 13.959315 |
| H | 13.259069 | 3.407388 | 15.006626 |
| H | 14.739508 | 2.636891 | 15.600163 |
| C | 14.437262 | 4.911025 | 17.021958 |
| H | 14.813939 | 5.820176 | 17.502342 |
| H | 14.819692 | 4.052270 | 17.593275 |
| H | 13.346604 | 4.911567 | 17.115563 |
| C | 16.403240 | 4.765905 | 15.468697 |
| H | 16.748007 | 4.692039 | 14.431177 |
| H | 16.772673 | 3.875948 | 15.999425 |
| H | 16.877441 | 5.637945 | 15.924567 |
| C | 14.497959 | 8.021437 | 15.137539 |
| C | 14.470937 | 9.008446 | 13.956105 |
| H | 15.252981 | 8.779258 | 13.222934 |
| H | 14.657007 | 10.025983 | 14.330719 |
| H | 13.502367 | 9.016673 | 13.450818 |
| C | 15.890183 | 8.114239 | 15.784136 |
| H | 16.686023 | 7.800667 | 15.098177 |
| H | 15.968600 | 7.517072 | 16.697751 |
| H | 16.090140 | 9.160244 | 16.059989 |
| C | 13.443973 | 8.456161 | 16.166207 |
| H | 12.445947 | 8.494390 | 15.715643 |
| H | 13.677477 | 9.462817 | 16.543363 |
| H | 13.412872 | 7.778661 | 17.027291 |
| O | 12.084394 | 3.442507 | 12.618726 |
| O | 10.801523 | 7.033874 | 11.735843 |
| O | 11.276863 | 8.553657 | 13.278387 |
| O | 12.542211 | 5.765339 | 10.145650 |
| O | 10.609706 | 6.252805 | 9.347097 |
| O | 10.641175 | 3.779350 | 10.130632 |
| O | 9.210065 | 4.950994 | 11.231523 |
| N | 12.427840 | 6.780290 | 8.035359 |
| C | 11.687617 | 4.596888 | 12.601605 |
| N | 14.529292 | 7.934331 | 8.291937 |
| N | 14.596521 | 5.932010 | 7.381627 |
| N | 8.429078 | 3.015587 | 10.264955 |
| N | 8.452860 | 1.893831 | 8.118985 |
| N | 8.856022 | 0.670907 | 9.904041 |
| C | 11.243495 | 7.378049 | 12.954956 |
| C | 11.887847 | 6.291196 | 9.125964 |
| C | 9.399350 | 3.860721 | 10.517675 |
| C | 13.764141 | 6.853403 | 7.953803 |
| C | 15.843284 | 7.669760 | 7.945408 |
| H | 16.649304 | 8.356653 | 8.132561 |
| C | 15.885855 | 6.439271 | 7.386297 |
| H | 16.734108 | 5.893634 | 7.013308 |
| C | 14.168351 | 4.625464 | 6.775442 |
| C | 13.167613 | 4.912937 | 5.650448 |
| H | 13.624039 | 5.537669 | 4.875070 |
| H | 12.279394 | 5.417762 | 6.035622 |
| H | 12.864160 | 3.964537 | 5.195513 |
| C | 15.401514 | 3.934022 | 6.189232 |
| H | 15.073455 | 2.999101 | 5.728191 |
| H | 16.135096 | 3.681321 | 6.962013 |
| H | 15.882224 | 4.535458 | 5.410814 |
| C | 13.553563 | 3.721008 | 7.848196 |
| H | 13.402379 | 2.724290 | 7.420890 |
| H | 12.588285 | 4.082799 | 8.200599 |
| H | 14.215986 | 3.630836 | 8.713887 |
| C | 14.007953 | 9.245238 | 8.823327 |
| C | 13.061963 | 9.846597 | 7.778334 |
| H | 13.587738 | 10.021634 | 6.833281 |
| H | 12.687997 | 10.805347 | 8.150277 |
| H | 12.208901 | 9.189426 | 7.598778 |
| C | 15.195406 | 10.186306 | 9.037165 |
| H | 15.732221 | 10.398305 | 8.106584 |
| H | 15.894976 | 9.799010 | 9.785881 |
| H | 14.803914 | 11.134192 | 9.414048 |
| C | 13.297157 | 9.037890 | 10.163655 |
| H | 12.348161 | 8.511427 | 10.067636 |
| H | 13.079484 | 10.015533 | 10.604124 |
| H | 13.935161 | 8.489859 | 10.861808 |
| C | 8.626255 | 1.951453 | 9.478247 |
| C | 8.604859 | 0.578834 | 7.710097 |
| H | 8.531338 | 0.271078 | 6.682503 |
| C | 8.847828 | -0.172151 | 8.806906 |
| H | 9.020718 | -1.231091 | 8.878818 |
| C | 8.991625 | 0.237560 | 11.337961 |
| C | 9.220416 | -1.275041 | 11.372514 |

| | | | |
|---|---|---|---|
| H | 8.379587 | -1.833166 | 10.947393 |
| H | 9.316271 | -1.571935 | 12.419823 |
| H | 10.146532 | -1.561167 | 10.862770 |
| C | 7.685777 | 0.567398 | 12.069044 |
| H | 7.495263 | 1.642193 | 12.072347 |
| H | 7.763190 | 0.225360 | 13.105595 |
| H | 6.839873 | 0.054723 | 11.598121 |
| C | 10.190847 | 0.937766 | 11.977756 |
| H | 11.102624 | 0.775467 | 11.396173 |
| H | 10.344142 | 0.538042 | 12.985183 |
| H | 10.047000 | 2.011373 | 12.071056 |
| C | 8.116522 | 3.045464 | 7.210923 |
| C | 7.771580 | 2.487128 | 5.826814 |
| H | 7.468465 | 3.325923 | 5.195377 |
| H | 6.936613 | 1.779600 | 5.864864 |
| H | 8.631862 | 2.011286 | 5.344652 |
| C | 9.329689 | 3.970517 | 7.089526 |
| H | 10.206833 | 3.408715 | 6.749935 |
| H | 9.578789 | 4.462302 | 8.029518 |
| H | 9.112433 | 4.751497 | 6.353544 |
| C | 6.892934 | 3.790876 | 7.755443 |
| H | 6.626009 | 4.581338 | 7.047696 |
| H | 7.090927 | 4.246162 | 8.725792 |
| H | 6.039192 | 3.111830 | 7.853841 |

Calculated energies and coordinates of **INT9a**

Electronic energy ... -1555.42146322 Eh
Total Enthalpy ... -1554.78005069 Eh
Final Gibbs free energy ... -1554.87476064 Eh

CARTESIAN COORDINATES (ANGSTROEM)

| | | | |
|---|---|---|---|
| Si | 2.696760 | -0.122690 | -0.236791 |
| O | 1.168044 | -0.326025 | -0.123997 |
| N | 3.757406 | -0.088911 | 1.028221 |
| N | 4.061292 | 0.924008 | 3.172824 |
| N | 3.882964 | -1.279227 | 3.124795 |
| N | 3.532953 | 0.094492 | -1.645173 |
| N | 3.016135 | 1.236669 | -3.708083 |
| N | 3.704170 | -0.860854 | -3.832567 |
| C | 3.848891 | -0.149469 | 2.313297 |
| C | 4.206400 | 0.453142 | 4.470975 |
| H | 4.386923 | 1.096065 | 5.314291 |
| C | 4.095252 | -0.890612 | 4.441681 |
| H | 4.165853 | -1.589425 | 5.256249 |
| C | 3.610196 | -2.670313 | 2.664629 |
| C | 3.853789 | -3.638383 | 3.826669 |
| H | 3.684781 | -4.654870 | 3.461021 |
| H | 3.159964 | -3.470058 | 4.656419 |
| H | 4.883103 | -3.581864 | 4.197305 |
| C | 4.572430 | -3.041863 | 1.527307 |
| H | 4.441969 | -2.394592 | 0.660133 |
| H | 4.385703 | -4.077939 | 1.226975 |
| H | 5.611840 | -2.959880 | 1.863170 |
| C | 2.143605 | -2.776081 | 2.228248 |
| H | 1.884602 | -2.068492 | 1.435103 |
| H | 1.484372 | -2.576321 | 3.079940 |
| H | 1.940543 | -3.790226 | 1.867595 |
| C | 4.080881 | 2.350936 | 2.750204 |
| C | 4.396526 | 3.229734 | 3.964010 |
| H | 4.422931 | 4.271163 | 3.631902 |
| H | 5.374671 | 2.992317 | 4.395204 |
| H | 3.628798 | 3.149600 | 4.740492 |
| C | 2.697644 | 2.740424 | 2.212363 |
| H | 2.711222 | 3.787936 | 1.893534 |
| H | 1.938285 | 2.624057 | 2.992503 |
| H | 2.400016 | 2.125487 | 1.360452 |
| C | 5.177789 | 2.562443 | 1.697366 |
| H | 5.006150 | 1.945107 | 0.814495 |
| H | 6.157688 | 2.307812 | 2.115299 |
| H | 5.192440 | 3.616188 | 1.399662 |
| C | 3.389404 | 0.147370 | -2.926873 |
| C | 3.511965 | -0.394548 | -5.126184 |
| H | 3.698891 | -0.991276 | -6.001427 |
| C | 3.090514 | 0.884268 | -5.049976 |
| H | 2.856097 | 1.564288 | -5.849660 |
| C | 2.491191 | 2.528143 | -3.180284 |
| C | 3.501096 | 3.142221 | -2.200474 |
| H | 4.465732 | 3.302467 | -2.694013 |
| H | 3.662704 | 2.500892 | -1.334507 |
| H | 3.121118 | 4.110223 | -1.858224 |
| C | 1.130795 | 2.281903 | -2.515598 |
| H | 0.410650 | 1.922004 | -3.258401 |
| H | 0.751698 | 3.220213 | -2.096598 |
| H | 1.179025 | 1.539043 | -1.713939 |
| C | 2.305896 | 3.506210 | -4.344839 |
| H | 1.548667 | 3.162352 | -5.056563 |
| H | 3.245626 | 3.691876 | -4.876287 |
| H | 1.959054 | 4.458288 | -3.934171 |
| C | 4.116809 | -2.239377 | -3.450282 |
| C | 2.965466 | -2.924006 | -2.701904 |
| H | 2.078896 | -2.986797 | -3.341356 |
| H | 2.685509 | -2.383145 | -1.795473 |
| H | 3.263994 | -3.939356 | -2.420863 |
| C | 5.389963 | -2.178300 | -2.595293 |
| H | 5.686084 | -3.195300 | -2.317568 |
| H | 5.229766 | -1.593146 | -1.688852 |
| H | 6.207422 | -1.723111 | -3.164678 |
| C | 4.419585 | -3.042446 | -4.718626 |
| H | 3.535496 | -3.153388 | -5.354812 |
| H | 4.739071 | -4.044220 | -4.418836 |
| H | 5.231284 | -2.593208 | -5.300386 |

Calculated energies and coordinates of
**TS[INT9a+INT4a_INT10a]**

Electronic energy ... -3248.20764779 Eh
Total Enthalpy ... -3246.95911386 Eh
Final Gibbs free energy ... -3247.11651444 Eh

CARTESIAN COORDINATES (ANGSTROEM)

| | | | |
|---|---|---|---|
| Si | 2.002822 | -0.020871 | -0.642780 |
| Si | -2.208993 | 0.743981 | 0.735338 |
| Si | -3.272329 | 0.193313 | 2.796006 |
| Si | -3.180318 | -0.056002 | -1.283267 |
| O | 0.449462 | 0.249842 | -0.532504 |
| O | 0.253504 | 0.018196 | 2.010128 |
| N | 2.999282 | -0.419734 | 0.592299 |
| N | 3.172571 | 0.532518 | 2.796080 |
| N | 2.756430 | -1.628598 | 2.654276 |
| N | 2.783688 | 0.098657 | -2.070584 |
| N | 2.642266 | 1.071720 | -4.261734 |
| N | 2.923707 | -1.116294 | -4.131208 |
| C | -0.516515 | 0.187920 | 1.129148 |
| C | 2.945348 | -0.489966 | 1.896037 |
| C | 3.061542 | 0.031502 | 4.085434 |
| H | 3.168508 | 0.640311 | 4.965125 |
| C | 2.809386 | -1.290012 | 3.998738 |
| H | 2.661567 | -2.001734 | 4.791064 |
| C | 2.510100 | -2.998533 | 2.115027 |

| | | | |
|---|---|---|---|
| C | 2.431605 | -3.988040 | 3.280941 |
| H | 2.276211 | -4.987331 | 2.865455 |
| H | 1.585980 | -3.770883 | 3.941415 |
| H | 3.356962 | -4.009788 | 3.866153 |
| C | 3.678739 | -3.399276 | 1.205468 |
| H | 3.769949 | -2.719720 | 0.356900 |
| H | 3.510109 | -4.414206 | 0.830801 |
| H | 4.620660 | -3.388675 | 1.764383 |
| C | 1.176038 | -3.035139 | 1.363328 |
| H | 1.160244 | -2.333555 | 0.528952 |
| H | 0.349148 | -2.779959 | 2.031336 |
| H | 1.009548 | -4.042381 | 0.967028 |
| C | 3.449391 | 1.956548 | 2.443067 |
| C | 3.780710 | 2.728722 | 3.724458 |
| H | 4.033441 | 3.754960 | 3.445236 |
| H | 4.643283 | 2.301456 | 4.246285 |
| H | 2.926380 | 2.774415 | 4.407311 |
| C | 2.207697 | 2.593329 | 1.807764 |
| H | 2.406859 | 3.651966 | 1.611849 |
| H | 1.347628 | 2.511582 | 2.477817 |
| H | 1.929510 | 2.122355 | 0.862345 |
| C | 4.664153 | 2.025801 | 1.508054 |
| H | 4.489146 | 1.479350 | 0.580503 |
| H | 5.547766 | 1.603357 | 1.998395 |
| H | 4.870137 | 3.074173 | 1.269278 |
| C | 2.745505 | 0.025109 | -3.360851 |
| C | 2.910047 | -0.763293 | -5.474319 |
| H | 3.009421 | -1.478299 | -6.271562 |
| C | 2.736587 | 0.571795 | -5.553500 |
| H | 2.663249 | 1.191152 | -6.429613 |
| C | 2.462131 | 2.506081 | -3.889871 |
| C | 3.619093 | 2.951265 | -2.985560 |
| H | 4.578094 | 2.820858 | -3.498441 |
| H | 3.643922 | 2.382155 | -2.055616 |
| H | 3.496909 | 4.012288 | -2.746230 |
| C | 1.102476 | 2.680214 | -3.207308 |
| H | 0.301751 | 2.408145 | -3.901287 |
| H | 0.963457 | 3.727798 | -2.920902 |
| H | 0.999006 | 2.062654 | -2.312485 |
| C | 2.486621 | 3.358205 | -5.161890 |
| H | 1.660574 | 3.111197 | -5.836321 |
| H | 3.436244 | 3.265282 | -5.699338 |
| H | 2.369212 | 4.404808 | -4.868888 |
| C | 3.231535 | -2.472407 | -3.590147 |
| C | 2.032145 | -3.000618 | -2.796357 |
| H | 1.156894 | -3.104265 | -3.444071 |
| H | 1.761596 | -2.339885 | -1.970771 |
| H | 2.273507 | -3.982890 | -2.377484 |
| C | 4.495077 | -2.399512 | -2.721619 |
| H | 4.739768 | -3.403186 | -2.358883 |
| H | 4.355458 | -1.740078 | -1.863299 |
| H | 5.340256 | -2.030578 | -3.312554 |
| C | 3.492048 | -3.430014 | -4.756229 |
| H | 2.616113 | -3.532451 | -5.405127 |
| H | 3.711769 | -4.416952 | -4.340320 |
| H | 4.354603 | -3.122038 | -5.355960 |
| C | -4.808098 | -0.930020 | -0.842395 |
| H | -5.430091 | -1.060660 | -1.735953 |
| H | -5.388156 | -0.362183 | -0.112739 |
| H | -4.619582 | -1.921457 | -0.416968 |
| C | -2.199922 | -1.333139 | -2.373233 |
| C | -3.178219 | -2.101965 | -3.282793 |
| H | -3.744356 | -1.441381 | -3.947022 |
| H | -3.894938 | -2.696375 | -2.707294 |
| H | -2.609586 | -2.797816 | -3.917932 |
| C | -1.527326 | -2.354216 | -1.441936 |
| H | -2.253037 | -2.838042 | -0.776861 |
| H | -0.765733 | -1.874003 | -0.822403 |
| H | -1.048815 | -3.146086 | -2.039209 |
| C | -1.129684 | -0.680292 | -3.260369 |
| H | -0.556349 | -1.461668 | -3.782758 |
| H | -0.428998 | -0.082890 | -2.671048 |
| H | -1.577818 | -0.044503 | -4.031972 |
| C | -3.696335 | 1.564083 | -2.222242 |
| C | -4.377640 | 1.271741 | -3.568401 |
| H | -4.738383 | 2.209235 | -4.017495 |
| H | -5.244298 | 0.609945 | -3.456967 |
| H | -3.689273 | 0.812697 | -4.285879 |
| C | -4.697548 | 2.318523 | -1.329025 |
| H | -4.956920 | 3.282177 | -1.791640 |
| H | -4.272182 | 2.532370 | -0.339505 |
| H | -5.628209 | 1.757455 | -1.189350 |
| C | -2.487252 | 2.477254 | -2.462674 |
| H | -1.770750 | 2.018423 | -3.148072 |
| H | -1.960521 | 2.695533 | -1.525706 |
| H | -2.812999 | 3.430887 | -2.905130 |
| C | -5.153777 | 0.247510 | 2.522453 |
| H | -5.440582 | 1.065268 | 1.853213 |
| H | -5.678568 | 0.395032 | 3.474113 |
| H | -5.518827 | -0.686821 | 2.085816 |
| C | -2.882968 | 1.660422 | 4.009760 |
| C | -3.402017 | 1.395637 | 5.431202 |
| H | -4.471911 | 1.156232 | 5.442964 |
| H | -3.262043 | 2.293990 | 6.051177 |
| H | -2.865660 | 0.576881 | 5.921280 |
| C | -3.594508 | 2.911916 | 3.464484 |
| H | -3.295035 | 3.122029 | 2.430262 |
| H | -3.329720 | 3.786802 | 4.076692 |
| H | -4.684747 | 2.808028 | 3.490733 |
| C | -1.378102 | 1.963568 | 4.070302 |
| H | -1.000971 | 2.232560 | 3.077241 |
| H | -0.791950 | 1.112110 | 4.430925 |
| H | -1.195027 | 2.813464 | 4.745631 |
| C | -2.902689 | -1.569450 | 3.544335 |
| C | -1.558854 | -1.646357 | 4.286121 |
| H | -0.723039 | -1.322446 | 3.660446 |
| H | -1.370968 | -2.686119 | 4.598283 |
| H | -1.554464 | -1.032583 | 5.193162 |
| C | -2.871106 | -2.577285 | 2.384193 |
| H | -3.810989 | -2.576085 | 1.818536 |
| H | -2.718030 | -3.596480 | 2.771332 |
| H | -2.065669 | -2.345665 | 1.679931 |
| C | -4.019115 | -1.994964 | 4.515556 |
| H | -4.995593 | -2.043904 | 4.022847 |
| H | -4.108644 | -1.318821 | 5.371773 |
| H | -3.800392 | -2.998597 | 4.910675 |

Calculated energies and coordinates of **INT10a**

Electronic energy ... -3248.21021310 Eh
Total Enthalpy ... -3246.96072268 Eh
Final Gibbs free energy ... -3247.11872625 Eh

CARTESIAN COORDINATES (ANGSTROEM)

| | | | |
|---|---|---|---|
| Si | 2.108898 | -0.155048 | -0.509147 |
| Si | -2.073953 | 0.699682 | 0.544720 |
| Si | -3.089564 | 0.298966 | 2.650867 |
| Si | -3.175561 | -0.168923 | -1.368513 |
| O | 0.502488 | 0.060998 | -0.463427 |
| O | 0.313217 | 0.007803 | 1.831654 |

| | | | |
|---|---|---|---|
| N | 3.104829 | -0.551288 | 0.715110 |
| N | 3.080543 | 0.450302 | 2.900853 |
| N | 2.659578 | -1.706949 | 2.778902 |
| N | 2.813225 | 0.025858 | -1.958355 |
| N | 2.417539 | 1.192780 | -4.025047 |
| N | 2.761341 | -0.986870 | -4.124147 |
| C | -0.325945 | 0.139891 | 0.797636 |
| C | 2.928730 | -0.593741 | 2.020966 |
| C | 2.841375 | -0.010587 | 4.186759 |
| H | 2.854514 | 0.625334 | 5.053405 |
| C | 2.582902 | -1.332105 | 4.112135 |
| H | 2.336258 | -2.017522 | 4.902939 |
| C | 2.453808 | -3.091627 | 2.253905 |
| C | 2.244834 | -4.042512 | 3.435409 |
| H | 2.140164 | -5.055848 | 3.038972 |
| H | 1.329751 | -3.808636 | 3.989447 |
| H | 3.098837 | -4.039546 | 4.120692 |
| C | 3.706218 | -3.524628 | 1.480976 |
| H | 3.895981 | -2.859440 | 0.636712 |
| H | 3.560013 | -4.542167 | 1.104312 |
| H | 4.583724 | -3.520659 | 2.136325 |
| C | 1.205562 | -3.143289 | 1.367167 |
| H | 1.337667 | -2.569402 | 0.448728 |
| H | 0.335005 | -2.745876 | 1.894120 |
| H | 1.007078 | -4.182771 | 1.086103 |
| C | 3.457056 | 1.849640 | 2.534492 |
| C | 3.546666 | 2.685145 | 3.814848 |
| H | 3.865956 | 3.693303 | 3.538815 |
| H | 4.285637 | 2.284981 | 4.516386 |
| H | 2.575188 | 2.768487 | 4.313334 |
| C | 2.392170 | 2.486072 | 1.630533 |
| H | 2.578273 | 3.563057 | 1.567145 |
| H | 1.387562 | 2.315432 | 2.023683 |
| H | 2.433334 | 2.097806 | 0.608049 |
| C | 4.829173 | 1.831886 | 1.849750 |
| H | 4.810651 | 1.217311 | 0.946908 |
| H | 5.592633 | 1.438012 | 2.528975 |
| H | 5.106043 | 2.854558 | 1.573321 |
| C | 2.638295 | 0.074585 | -3.245720 |
| C | 2.588854 | -0.520686 | -5.420069 |
| H | 2.609635 | -1.161931 | -6.283230 |
| C | 2.380740 | 0.810751 | -5.359577 |
| H | 2.194477 | 1.501794 | -6.162234 |
| C | 2.329475 | 2.597132 | -3.515850 |
| C | 3.608302 | 2.932510 | -2.736555 |
| H | 4.489332 | 2.812864 | -3.376158 |
| H | 3.726684 | 2.292238 | -1.861338 |
| H | 3.560961 | 3.974467 | -2.405187 |
| C | 1.074656 | 2.755915 | -2.653788 |
| H | 0.177157 | 2.586020 | -3.254169 |
| H | 1.030913 | 3.775587 | -2.257983 |
| H | 1.043767 | 2.062718 | -1.812179 |
| C | 2.221025 | 3.551327 | -4.708310 |
| H | 1.311885 | 3.373693 | -5.291557 |
| H | 3.094460 | 3.492772 | -5.366298 |
| H | 2.164666 | 4.571201 | -4.318990 |
| C | 3.138776 | -2.380715 | -3.737133 |
| C | 2.014193 | -3.011140 | -2.910203 |
| H | 1.118456 | -3.151981 | -3.520775 |
| H | 1.737227 | -2.392933 | -2.053785 |
| H | 2.337145 | -3.987361 | -2.534730 |
| C | 4.462977 | -2.355094 | -2.961073 |
| H | 4.768087 | -3.384248 | -2.746964 |
| H | 4.369345 | -1.812910 | -2.019282 |
| H | 5.246957 | -1.881096 | -3.561161 |
| C | 3.333068 | -3.214911 | -5.005912 |

| | | | |
|---|---|---|---|
| H | 2.415186 | -3.283289 | -5.598707 |
| H | 3.603883 | -4.230263 | -4.704340 |
| H | 4.142332 | -2.825542 | -5.632193 |
| C | -4.754773 | -1.090864 | -0.849017 |
| H | -5.407504 | -1.276590 | -1.710343 |
| H | -5.328331 | -0.524536 | -0.112999 |
| H | -4.508453 | -2.058653 | -0.399342 |
| C | -2.195065 | -1.454258 | -2.456064 |
| C | -3.154952 | -2.233024 | -3.374560 |
| H | -3.708499 | -1.580265 | -4.055730 |
| H | -3.882916 | -2.816973 | -2.802178 |
| H | -2.579121 | -2.940441 | -3.991180 |
| C | -1.528166 | -2.476115 | -1.519825 |
| H | -2.260488 | -2.960114 | -0.862676 |
| H | -0.774204 | -2.005864 | -0.883927 |
| H | -1.044243 | -3.268260 | -2.112901 |
| C | -1.121616 | -0.791205 | -3.331087 |
| H | -0.530878 | -1.560963 | -3.851204 |
| H | -0.434292 | -0.179450 | -2.738279 |
| H | -1.569783 | -0.158992 | -4.105237 |
| C | -3.793080 | 1.386559 | -2.366966 |
| C | -4.540713 | 1.021205 | -3.657836 |
| H | -4.969883 | 1.926965 | -4.112501 |
| H | -5.367422 | 0.324852 | -3.475785 |
| H | -3.875331 | 0.570605 | -4.402334 |
| C | -4.761547 | 2.151044 | -1.446107 |
| H | -5.089209 | 3.079724 | -1.936606 |
| H | -4.276318 | 2.427965 | -0.500976 |
| H | -5.657963 | 1.565360 | -1.213935 |
| C | -2.636363 | 2.332081 | -2.718233 |
| H | -1.957348 | 1.881030 | -3.448387 |
| H | -2.054689 | 2.591502 | -1.824835 |
| H | -3.026659 | 3.262565 | -3.158337 |
| C | -4.980884 | 0.358430 | 2.434959 |
| H | -5.277571 | 1.121736 | 1.708066 |
| H | -5.474751 | 0.593567 | 3.385582 |
| H | -5.372596 | -0.603160 | 2.089761 |
| C | -2.665375 | 1.827475 | 3.779500 |
| C | -3.145201 | 1.646804 | 5.227197 |
| H | -4.215124 | 1.413326 | 5.282161 |
| H | -2.985763 | 2.578089 | 5.792016 |
| H | -2.598555 | 0.853733 | 5.747503 |
| C | -3.386024 | 3.050761 | 3.184574 |
| H | -3.115454 | 3.197486 | 2.131494 |
| H | -3.100149 | 3.957874 | 3.738205 |
| H | -4.475588 | 2.954808 | 3.247151 |
| C | -1.158791 | 2.125477 | 3.781659 |
| H | -0.812469 | 2.332882 | 2.762874 |
| H | -0.567330 | 1.288708 | 4.167051 |
| H | -0.952617 | 3.011630 | 4.402708 |
| C | -2.727693 | -1.422075 | 3.502155 |
| C | -1.365523 | -1.476132 | 4.211128 |
| H | -0.548804 | -1.195735 | 3.540903 |
| H | -1.184927 | -2.498898 | 4.581728 |
| H | -1.327608 | -0.809463 | 5.079666 |
| C | -2.741337 | -2.495735 | 2.402099 |
| H | -3.709031 | -2.535212 | 1.887085 |
| H | -2.557511 | -3.490108 | 2.838734 |
| H | -1.977899 | -2.296414 | 1.642640 |
| C | -3.823772 | -1.776539 | 4.522731 |
| H | -4.814192 | -1.831603 | 4.059395 |
| H | -3.877952 | -1.055713 | 5.344646 |
| H | -3.612603 | -2.762915 | 4.963851 |

Calculated energies and coordinates of **TS[INT10a_4]**

Electronic energy ... -3436.78744462 Eh
Total Enthalpy ... -3435.52235224 Eh
Final Gibbs free energy ... -3435.68684336 Eh

CARTESIAN COORDINATES (ANGSTROEM)

| | | | |
|---|---|---|---|
| Si | -1.720809 | 0.652600 | -0.258192 |
| Si | 2.171355 | -1.114913 | 0.626997 |
| Si | 4.294866 | -0.042393 | 0.455287 |
| Si | 1.878863 | -3.168123 | -0.566763 |
| O | -0.401365 | -0.281805 | -0.088427 |
| O | 1.078907 | 1.432292 | 0.177699 |
| O | -0.058935 | -0.480750 | 3.024236 |
| O | 1.694833 | -1.719648 | 3.905556 |
| N | -1.767981 | 2.249565 | -0.572519 |
| N | -0.540561 | 4.001149 | 0.536983 |
| N | -0.107156 | 3.665478 | -1.594333 |
| N | -3.071696 | -0.210192 | -0.248770 |
| N | -4.765692 | -1.343062 | 1.005939 |
| N | -5.230529 | -0.797726 | -1.084056 |
| C | 0.956104 | 0.217733 | 0.142396 |
| C | 0.918725 | -1.101561 | 3.269003 |
| C | -0.855310 | 3.205503 | -0.537354 |
| C | 0.444312 | 4.898743 | 0.155460 |
| H | 0.892766 | 5.603273 | 0.832321 |
| C | 0.706215 | 4.697291 | -1.151545 |
| H | 1.420898 | 5.195303 | -1.781430 |
| C | -0.193767 | 3.183575 | -3.007307 |
| C | 0.166242 | 1.697702 | -3.092361 |
| H | 0.282918 | 1.413778 | -4.143115 |
| H | -0.615204 | 1.063941 | -2.669534 |
| H | 1.102937 | 1.492715 | -2.568113 |
| C | 0.809403 | 3.965753 | -3.859249 |
| H | 0.720363 | 3.616028 | -4.890967 |
| H | 1.839865 | 3.786135 | -3.534961 |
| H | 0.604462 | 5.040973 | -3.855502 |
| C | -1.611702 | 3.444785 | -3.527931 |
| H | -2.352321 | 2.923200 | -2.918368 |
| H | -1.691052 | 3.085938 | -4.559420 |
| H | -1.835659 | 4.516798 | -3.515009 |
| C | -1.136405 | 3.908681 | 1.907127 |
| C | -0.603513 | 5.065651 | 2.757634 |
| H | 0.475912 | 4.988648 | 2.922425 |
| H | -1.088331 | 5.014283 | 3.735932 |
| H | -0.838838 | 6.040892 | 2.318774 |
| C | -0.723895 | 2.594281 | 2.570354 |
| H | -1.126900 | 1.722044 | 2.053997 |
| H | -1.102211 | 2.569550 | 3.597266 |
| H | 0.362956 | 2.483760 | 2.584748 |
| C | -2.661363 | 4.040607 | 1.803876 |
| H | -2.933526 | 5.000928 | 1.352864 |
| H | -3.091114 | 3.997062 | 2.809715 |
| H | -3.093117 | 3.239274 | 1.202292 |
| C | -4.246144 | -0.723582 | -0.115274 |
| C | -6.335636 | -1.451153 | -0.554852 |
| H | -7.232744 | -1.636889 | -1.118048 |
| C | -6.051338 | -1.781620 | 0.721969 |
| H | -6.665769 | -2.296415 | 1.439088 |
| C | -4.030945 | -1.551141 | 2.292558 |
| C | -3.709355 | -0.189696 | 2.919117 |
| H | -3.135376 | -0.338818 | 3.838128 |
| H | -4.631151 | 0.351595 | 3.158008 |
| H | -3.107229 | 0.428025 | 2.252071 |
| C | -2.763816 | -2.370187 | 2.025011 |
| H | -2.054236 | -1.843417 | 1.387343 |
| H | -3.022450 | -3.323586 | 1.552558 |
| H | -2.260769 | -2.578870 | 2.973677 |
| C | -4.926573 | -2.333135 | 3.256282 |
| H | -5.850486 | -1.794909 | 3.493470 |
| H | -4.374378 | -2.468594 | 4.189723 |
| H | -5.172195 | -3.327644 | 2.869907 |
| C | -5.126234 | -0.214635 | -2.452866 |
| C | -3.951548 | -0.860177 | -3.199268 |
| H | -3.000062 | -0.676408 | -2.698409 |
| H | -3.896936 | -0.447589 | -4.211640 |
| H | -4.094473 | -1.943014 | -3.274435 |
| C | -4.962144 | 1.306182 | -2.337429 |
| H | -5.820871 | 1.740651 | -1.814573 |
| H | -4.914867 | 1.742822 | -3.340533 |
| H | -4.053630 | 1.581252 | -1.797874 |
| C | -6.414871 | -0.515345 | -3.222444 |
| H | -6.316714 | -0.093570 | -4.226145 |
| H | -7.290108 | -0.053440 | -2.754345 |
| H | -6.585760 | -1.591373 | -3.330555 |
| C | 3.550933 | -3.677768 | -1.311164 |
| H | 3.815572 | -3.035300 | -2.157871 |
| H | 3.521642 | -4.712160 | -1.673022 |
| H | 4.356496 | -3.602897 | -0.577901 |
| C | 0.635685 | -3.181666 | -2.073160 |
| C | -0.832893 | -3.282977 | -1.635592 |
| H | -1.488866 | -3.157917 | -2.511762 |
| H | -1.098799 | -2.513661 | -0.904603 |
| H | -1.057546 | -4.263449 | -1.202241 |
| C | 0.943950 | -4.362913 | -3.012254 |
| H | 0.218838 | -4.369664 | -3.840411 |
| H | 0.875620 | -5.332248 | -2.509446 |
| H | 1.942670 | -4.282248 | -3.453097 |
| C | 0.818308 | -1.892306 | -2.886948 |
| H | 1.846698 | -1.782501 | -3.250135 |
| H | 0.578138 | -1.009079 | -2.291226 |
| H | 0.155950 | -1.907184 | -3.767525 |
| C | 1.475388 | -4.517017 | 0.773655 |
| C | 1.332365 | -5.927192 | 0.180655 |
| H | 1.221828 | -6.662931 | 0.991569 |
| H | 2.209240 | -6.221458 | -0.407396 |
| H | 0.447129 | -6.015979 | -0.458457 |
| C | 2.655740 | -4.525251 | 1.762836 |
| H | 2.815556 | -3.537190 | 2.210958 |
| H | 3.587909 | -4.843098 | 1.282515 |
| H | 2.450477 | -5.228063 | 2.583780 |
| C | 0.196964 | -4.182561 | 1.553851 |
| H | 0.213768 | -3.151874 | 1.922709 |
| H | 0.096032 | -4.849737 | 2.423020 |
| H | -0.699512 | -4.300455 | 0.938611 |
| C | 5.650192 | -1.376791 | 0.445151 |
| H | 6.616512 | -0.946273 | 0.734544 |
| H | 5.770295 | -1.821744 | -0.547243 |
| H | 5.421704 | -2.184161 | 1.148875 |
| C | 4.572059 | 0.958574 | 2.099048 |
| C | 3.350577 | 1.821922 | 2.446449 |
| H | 3.523330 | 2.350236 | 3.397041 |
| H | 2.455981 | 1.199143 | 2.560682 |
| H | 3.131395 | 2.564831 | 1.672663 |
| C | 5.818070 | 1.856095 | 2.045143 |
| H | 6.722369 | 1.293339 | 1.784980 |
| H | 5.990303 | 2.308397 | 3.033727 |
| H | 5.709021 | 2.675675 | 1.327612 |
| C | 4.774502 | -0.067578 | 3.228812 |
| H | 4.823731 | 0.450400 | 4.198325 |
| H | 5.708909 | -0.625578 | 3.105262 |
| H | 3.950532 | -0.786833 | 3.278629 |
| C | 4.603451 | 1.014921 | -1.158132 |

| | | | |
|---|---|---|---|
| C | 3.932346 | 2.396661 | -1.114315 |
| H | 4.365421 | 3.039209 | -0.340241 |
| H | 2.859174 | 2.322312 | -0.919267 |
| H | 4.081969 | 2.907987 | -2.079541 |
| C | 4.023672 | 0.237420 | -2.348685 |
| H | 2.950544 | 0.063055 | -2.222951 |
| H | 4.503082 | -0.742086 | -2.465582 |
| H | 4.181833 | 0.796867 | -3.284122 |
| C | 6.109719 | 1.209980 | -1.408690 |
| H | 6.597827 | 1.764450 | -0.601654 |
| H | 6.257272 | 1.782926 | -2.337001 |
| H | 6.633829 | 0.255876 | -1.526502 |

---

Calculated energies and coordinates of **4**

Electronic energy ... -3436.88950705 Eh
Total Enthalpy ... -3435.62102148 Eh
Final Gibbs free energy ... -3435.78315958 Eh

CARTESIAN COORDINATES (ANGSTROEM)

| | | | |
|---|---|---|---|
| Si | 5.075555 | 4.660146 | 7.124542 |
| Si | 1.554051 | 4.662312 | 7.543000 |
| Si | 0.599943 | 5.016090 | 9.763294 |
| Si | 0.102539 | 4.317563 | 5.606707 |
| O | 4.026245 | 3.328163 | 7.113306 |
| O | 2.423849 | 2.034124 | 7.932807 |
| O | 4.061185 | 5.995902 | 7.372906 |
| O | 2.306492 | 7.286078 | 6.957230 |
| N | 6.068164 | 4.557852 | 8.427274 |
| N | 8.072175 | 5.187347 | 9.578576 |
| N | 7.290894 | 3.150720 | 9.954895 |
| N | 5.742197 | 4.754323 | 5.628266 |
| N | 6.580947 | 6.155237 | 3.854627 |
| N | 7.392344 | 4.102567 | 4.021870 |
| C | 2.772032 | 3.146744 | 7.588521 |
| C | 2.728636 | 6.174665 | 7.210591 |
| C | 7.042994 | 4.314953 | 9.220739 |
| C | 8.919345 | 4.560505 | 10.481777 |
| H | 9.788474 | 5.035162 | 10.901327 |
| C | 8.438971 | 3.323268 | 10.715689 |
| H | 8.829876 | 2.564320 | 11.369705 |
| C | 6.429374 | 1.933279 | 9.968695 |
| C | 7.005324 | 0.915501 | 10.957129 |
| H | 6.348954 | 0.041146 | 10.956914 |
| H | 7.035898 | 1.306735 | 11.979265 |
| H | 8.006149 | 0.578113 | 10.667397 |
| C | 6.406899 | 1.297114 | 8.575130 |
| H | 5.999397 | 1.978314 | 7.829169 |
| H | 5.769405 | 0.407451 | 8.591915 |
| H | 7.418308 | 0.998782 | 8.280118 |
| C | 5.020868 | 2.315452 | 10.429604 |
| H | 4.580365 | 3.060670 | 9.768657 |
| H | 5.051163 | 2.719998 | 11.447330 |
| H | 4.378659 | 1.429788 | 10.420445 |
| C | 8.288842 | 6.533689 | 8.984512 |
| C | 9.483999 | 7.201237 | 9.671979 |
| H | 9.601987 | 8.202768 | 9.249478 |
| H | 10.418014 | 6.657058 | 9.497812 |
| H | 9.324637 | 7.311211 | 10.749825 |
| C | 7.051492 | 7.412540 | 9.210429 |
| H | 7.226074 | 8.398857 | 8.767844 |
| H | 6.871136 | 7.542741 | 10.282907 |
| H | 6.157186 | 6.983131 | 8.759295 |
| C | 8.609873 | 6.369888 | 7.493560 |
| H | 7.776105 | 5.919385 | 6.952235 |
| H | 9.494069 | 5.735640 | 7.368702 |
| H | 8.821508 | 7.348846 | 7.052000 |
| C | 6.496998 | 4.989338 | 4.622140 |
| C | 8.006388 | 4.721791 | 2.942332 |
| H | 8.740978 | 4.236689 | 2.324373 |
| C | 7.507187 | 5.969346 | 2.837134 |
| H | 7.743582 | 6.727549 | 2.111958 |
| C | 5.757237 | 7.383044 | 4.050688 |
| C | 6.077785 | 8.014874 | 5.409334 |
| H | 7.135519 | 8.293659 | 5.457770 |
| H | 5.843471 | 7.339443 | 6.231174 |
| H | 5.470612 | 8.915849 | 5.541285 |
| C | 4.274025 | 7.019430 | 3.944729 |
| H | 4.051847 | 6.613766 | 2.951613 |
| H | 3.664687 | 7.913687 | 4.106013 |
| H | 3.998627 | 6.281533 | 4.697003 |
| C | 6.090153 | 8.397572 | 2.952915 |
| H | 5.865712 | 8.010099 | 1.953714 |
| H | 7.136468 | 8.719018 | 2.991265 |
| H | 5.466473 | 9.280949 | 3.113843 |
| C | 7.702060 | 2.739234 | 4.528756 |
| C | 6.419410 | 1.898590 | 4.588793 |
| H | 5.983779 | 1.803558 | 3.588328 |
| H | 5.672728 | 2.337742 | 5.250375 |
| H | 6.663210 | 0.896165 | 4.955898 |
| C | 8.369648 | 2.861817 | 5.904095 |
| H | 8.634790 | 1.866536 | 6.274259 |
| H | 7.706384 | 3.335267 | 6.630242 |
| H | 9.285748 | 3.456979 | 5.824760 |
| C | 8.680127 | 2.054274 | 3.569053 |
| H | 8.265310 | 1.966494 | 2.559435 |
| H | 8.867278 | 1.043278 | 3.940954 |
| H | 9.643673 | 2.572108 | 3.521103 |
| C | -1.401917 | 3.321356 | 6.172886 |
| H | -2.006633 | 3.032885 | 5.304872 |
| H | -2.040271 | 3.904443 | 6.844913 |
| H | -1.109915 | 2.408142 | 6.696132 |
| C | 1.012749 | 3.250282 | 4.269508 |
| C | 0.207323 | 3.237344 | 2.956883 |
| H | 0.193914 | 4.215129 | 2.466074 |
| H | -0.828609 | 2.910961 | 3.104811 |
| H | 0.674827 | 2.528687 | 2.257794 |
| C | 1.115494 | 1.795300 | 4.761137 |
| H | 0.127638 | 1.337302 | 4.880567 |
| H | 1.647037 | 1.703775 | 5.710863 |
| H | 1.664323 | 1.201656 | 4.015763 |
| C | 2.421044 | 3.778707 | 3.965189 |
| H | 2.895338 | 3.153445 | 3.194221 |
| H | 3.076712 | 3.756637 | 4.840476 |
| H | 2.396822 | 4.804486 | 3.588031 |
| C | -0.566722 | 6.016379 | 4.954311 |
| C | -1.837795 | 5.793075 | 4.111010 |
| H | -2.208235 | 6.767784 | 3.762063 |
| H | -2.639564 | 5.331517 | 4.697080 |
| H | -1.663763 | 5.174203 | 3.226769 |
| C | -0.962953 | 6.891585 | 6.153303 |
| H | -1.428901 | 7.820110 | 5.791706 |
| H | -0.091268 | 7.170935 | 6.752312 |
| H | -1.694454 | 6.389520 | 6.795389 |
| C | 0.464381 | 6.782091 | 4.112711 |
| H | 0.711175 | 6.258314 | 3.183452 |
| H | 1.387272 | 6.962851 | 4.670182 |
| H | 0.052400 | 7.763222 | 3.834497 |
| C | -0.998821 | 6.007443 | 9.568946 |
| H | -0.841964 | 6.922230 | 8.993481 |
| H | -1.382760 | 6.291715 | 10.555908 |

| | | | |
|---|---|---|---|
| H | -1.775237 | 5.421106 | 9.066658 |
| C | 1.797217 | 6.089400 | 10.846300 |
| C | 1.327430 | 6.090415 | 12.312550 |
| H | 0.288676 | 6.424700 | 12.417662 |
| H | 1.953015 | 6.787788 | 12.888531 |
| H | 1.420367 | 5.106709 | 12.782151 |
| C | 1.766883 | 7.547872 | 10.355364 |
| H | 2.060227 | 7.653598 | 9.308149 |
| H | 2.467990 | 8.144178 | 10.956985 |
| H | 0.773506 | 7.994080 | 10.474425 |
| C | 3.242626 | 5.575461 | 10.802621 |
| H | 3.671952 | 5.612568 | 9.797202 |
| H | 3.322347 | 4.546918 | 11.164184 |
| H | 3.879817 | 6.200812 | 11.445411 |
| C | 0.111647 | 3.316918 | 10.558369 |
| C | 1.318961 | 2.562663 | 11.133085 |
| H | 2.085167 | 2.389917 | 10.372865 |
| H | 0.994317 | 1.578316 | 11.501153 |
| H | 1.774823 | 3.090594 | 11.976908 |
| C | -0.544126 | 2.435043 | 9.484234 |
| H | -1.420423 | 2.919216 | 9.039974 |
| H | -0.887175 | 1.494462 | 9.939976 |
| H | 0.164545 | 2.177782 | 8.691690 |
| C | -0.928679 | 3.531497 | 11.674834 |
| H | -1.843619 | 3.998216 | 11.295048 |
| H | -0.552564 | 4.142241 | 12.500015 |
| H | -1.209781 | 2.553465 | 12.091487 |

# 4 References

(1) Fulmer, G. R.; Miller, A. J. M.; Sherden, N. H.; Gottlieb, H. E.; Nudelman, A.; Stoltz, B. M.; Bercaw, J. E.; Goldberg, K. I. NMR Chemical Shifts of Trace Impurities: Common Laboratory Solvents, Organics, and Gases in Deuterated Solvents Relevant to the Organometallic Chemist. *Organometallics* **2010**, *29* (9), 2176–2179.

(2) Muhr, M.; Heiß, P.; Schütz, M.; Bühler, R.; Gemel, C.; Linden, M. H.; Linden, H. B.; Fischer, R. A. Enabling LIFDI-MS measurements of highly air sensitive organometallic compounds: a combined MS/glovebox technique. *Dalton Trans.* **2021**, *50* (26), 9031–9036.

(3) Holzner, R.; Porzelt, A.; Karaca, U. S.; Kiefer, F.; Frisch, P.; Wendel, D.; Holthausen, M. C.; Inoue, S. Imino(silyl)disilenes: application in versatile bond activation, reversible oxidation and thermal isomerization. *Dalton Trans.* **2021**, *50* (25), 8785–8793.

(4) Sekiguchi, A.; Inoue, S.; Ichinohe, M.; Arai, Y. Isolable anion radical of blue disilene (tBu2MeSi)2Si=Si(SiMetBu2)2 formed upon one-electron reduction: synthesis and characterization. *J. Am. Chem. Soc.* **2004**, *126* (31), 9626–9629.

(5) Gau, D.; Rodriguez, R.; Kato, T.; Saffon-Merceron, N.; Cózar, A. de; Cossío, F. P.; Baceiredo, A. Synthesis of a stable disilyne bisphosphine adduct and its non-metal-mediated CO2 reduction to CO. *Angew. Chem. Int. Ed.* **2011**, *50* (5), 1092–1096.

(6) EPFL. https://mstools.epfl.ch/info/.

(7) Wang, J.; Hwang, G. B.; Knapp, C. E.; Wilson, D. W. N. Reversible CO2 insertion into the silicon-nitrogen σ-bond of an N-heterocyclic iminosilane. *Chem. Commun.* **2024**, *60* (89), 13051–13054.

(8) Bruker. *APEX4*; Bruker AXS Inc., 2021.

(9) *7.56 a and SADABS Version 2008/1*, 2008.

(10) *SHELXL-2014: Program for Crystal Structure Refinement*, 2014.

(11) Sheldrick, G. M. Crystal structure refinement with \it SHELXL. *Acta Crystallogr. C* **2015**, *71* (1), 3–8.

(12) *SHELXL-97; University of Göttingen: Göttingen, Germany, 1997*, 2010.

(13) A. J. C. Wilson. *In International Tables for Crystallography*; Kluwer Academic Publishers, 1992.

(14) Spek, A. L. Single-crystal structure validation with the program PLATON. *J. Appl. Crystallogr.* **2003**, *36* (1), 7–13.

(15) D. Kratzert. *FinalCif*; D. Kratzert. https://dkratzert.de/finalcif.html.

(16) Macrae, C. F.; Bruno, I. J.; Chisholm, J. A.; Edgington, P. R.; McCabe, P.; Pidcock, E.; Rodriguez-Monge, L.; Taylor, R.; van de Streek, J.; Wood, P. A. Mercury CSD 2.0 – new features for the visualization and investigation of crystal structures. *J. Appl. Crystallogr.* **2008**, *41* (2), 466–470.

(17) Neese, F. Software update: The ORCA program system—Version 5.0. *WIREs Comput Mol Sci* **2022**, *12* (5).
(18) Grimme, S.; Hansen, A.; Ehlert, S.; Mewes, J.-M. r2SCAN-3c: A "Swiss army knife" composite electronic-structure method. *J. Chem. Phys.* **2021**, *154* (6), 64103.
(19) Furness, J. W.; Kaplan, A. D.; Ning, J.; Perdew, J. P.; Sun, J. Accurate and Numerically Efficient r2SCAN Meta-Generalized Gradient Approximation. *J. Phys. Chem. Lett.* **2020**, *11* (19), 8208–8215.
(20) Furness, J. W.; Kaplan, A. D.; Ning, J.; Perdew, J. P.; Sun, J. Correction to "Accurate and Numerically Efficient r2SCAN Meta-Generalized Gradient Approximation". *J. Phys. Chem. Lett.* **2020**, *11* (21), 9248.
(21) Kruse, H.; Grimme, S. A geometrical correction for the inter- and intra-molecular basis set superposition error in Hartree-Fock and density functional theory calculations for large systems. *J. Chem. Phys.* **2012**, *136* (15), 154101.
(22) Caldeweyher, E.; Bannwarth, C.; Grimme, S. Extension of the D3 dispersion coefficient model. *J. Phys. Chem.* **2017**, *147* (3), 34112.
(23) Caldeweyher, E.; Ehlert, S.; Hansen, A.; Neugebauer, H.; Spicher, S.; Bannwarth, C.; Grimme, S. A generally applicable atomic-charge dependent London dispersion correction. *J. Chem. Phys.* **2019**, *150* (15), 154122.
(24) Caldeweyher, E.; Mewes, J.-M.; Ehlert, S.; Grimme, S. Extension and evaluation of the D4 London-dispersion model for periodic systems. *Phys. Chem. Chem. Phys.* **2020**, *22* (16), 8499–8512.
(25) Marenich, A. V.; Cramer, C. J.; Truhlar, D. G. Universal solvation model based on solute electron density and on a continuum model of the solvent defined by the bulk dielectric constant and atomic surface tensions. *J. Phys. Chem. B* **2009**, *113* (18), 6378–6396.
(26) Zhao, Y.; Truhlar, D. G. Design of density functionals that are broadly accurate for thermochemistry, thermochemical kinetics, and nonbonded interactions. *J. Phys. Chem. A* **2005**, *109* (25), 5656–5667.
(27) Weigend, F.; Ahlrichs, R. Balanced basis sets of split valence, triple zeta valence and quadruple zeta valence quality for H to Rn: Design and assessment of accuracy. *Phys. Chem. Chem. Phys.* **2005**, *7* (18), 3297–3305.
(28) Weigend, F. Accurate Coulomb-fitting basis sets for H to Rn. *Phys. Chem. Chem. Phys.* **2006**, *8* (9), 1057–1065.
(29) Hellweg, A.; Hättig, C.; Höfener, S.; Klopper, W. Optimized accurate auxiliary basis sets for RI-MP2 and RI-CC2 calculations for the atoms Rb to Rn. *Theor. Chem. Acc.* **2007**, *117* (4), 587–597.
(30) Adamo, C.; Barone, V. Toward reliable density functional methods without adjustable parameters: The PBE0 model. *J. Chem. Phys.* **1999**, *110* (13), 6158–6170.

(31) E. D. Glendening, J, K. Badenhoop, A. E. Reed, J. E. Carpenter, J. A. Bohmann, C. M. Morales, P. Karafiloglou, C. R. Landis, and F. Weinhold. *NBO 7.0*; Theoretical Chemistry Institute, University of Wisconsin, Madison, 2018.
